# Supplementary material for: Divergent and gram-scale syntheses of (–)-veratramine and (–)-cyclopamine
Source: Nat Commun. 2024 Jun 22;15:5332. doi: 10.1038/s41467-024-49748-2 (PMC11193734; doi:10.1038/s41467-024-49748-2)
Supplement: Supplementary file 1 — Supplementary Information [file 41467_2024_49748_MOESM1_ESM.pdf]

Supplementary Information

Divergent and gram-scale syntheses of (–)-veratramine and (–)-cyclopamine

Wenlong Hou, Hao Lin, Yanru Wu, Chuang Li, Jiajun Chen, Xiao-Yu Liu,* and Yong Qin*

Key Laboratory of Drug-Targeting and Drug Delivery System of the Education Ministry and Sichuan Province, Sichuan Engineering Laboratory for Plant-Sourced Drug and Sichuan Research Center for Drug Precision Industrial Technology, West China School of Pharmacy, Sichuan University, Chengdu, 610041, China.

*Corresponding authors: xyliu@scu.edu.cn (X.-Y.L.); yongqin@scu.edu.cn (Y.Q.)

Table of Contents

1. **General Information**....................S2

2. **Experimental Procedures and Characterization Data**....................S3

3. **X-ray Crystallographic Data**....................S52

4. **NMR Spectra**....................S56

5. **References**....................S113

1. General Information

All reactions that require anhydrous conditions were performed in flame-dried glassware under Ar atmosphere and all reagents were purchased from commercial suppliers. Dry toluene was obtained according to *Purification of Laboratory Chemicals* (Peerrin, D. D.; Armarego, W. L. and Perrins, D. R., Pergamon Press: Oxford, 1980). Other dry solvents were purchased from Energy Chemical. Reactions were monitored by thin layer chromatography (TLC) supplied by Yantai Chemicals. Visualization was accomplished with UV light, exposure to iodine, stained with ethanolic solution of phosphomolybdic acid or basic solution of $KMnO_4$. The reaction products were purified by column chromatography on silica gel (200–300 meshes) from the Anhui Liangchen Silicon Material Company. ^{1}H NMR and ^{13}C NMR spectra were recorded on Varian INOVA-400/54 and Agilent DD2-600/54 instruments. Solvent signal was used as reference for ^{1}H NMR (CD_2Cl_2, 5.32 ppm, $CDCl_3$, 7.26 ppm, CD_3OD, 3.31 ppm, CD_3CN, 1.94 ppm, C_5D_5N, 7.20, 7.57, 8.72 ppm) and ^{13}C NMR (CD_2Cl_2, 53.8 ppm, $CDCl_3$, 77.0 ppm, CD_3OD, 49.0 ppm, CD_3CN, 1.32, 118.3 ppm, C_5D_5N, 123.4, 135.4, 149.8 ppm). The following abbreviations are used to explain the multiplicities: s = singlet, d = doublet, t = triplet, q = quartet, br = broad, m = multiplet, and coupling constants (J) are reported in Hertz (Hz). Infrared (IR) spectra were recorded on a Perkin Elmer Spectrum Two FT-IR spectrometer. High-resolution mass spectra (HRMS) were recorded on Bruker Apex IV FTMS or Thermo Scientific LTQ Orbitrap XL ESI mass spectrometers. LC-MS analysis was performed on HP Agilent 6420 Triple Quad LC/MS. The specific optical rotation was obtained from Rudolph Research Analytical Autopol VI automatic polarimeter.

2. Experimental Procedures and Characterization Data

2.1 Synthesis of (–)-veratramine and (–)-cyclopamine

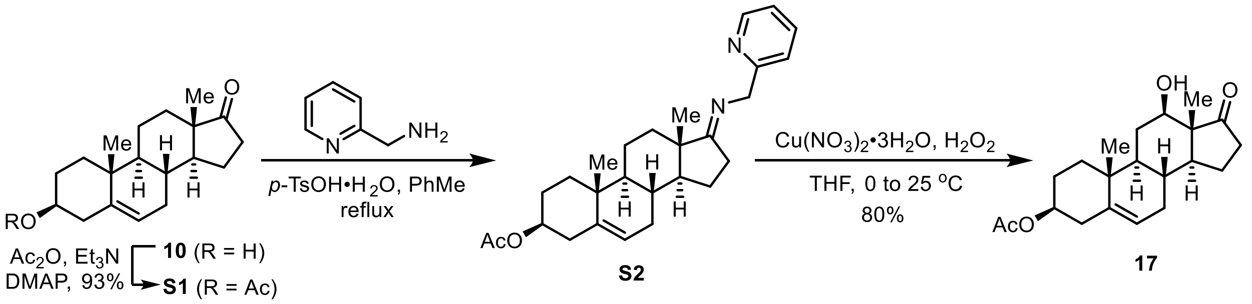

To a solution of dehydro-*epi*-androsterone (DHEA, **10**, 50.0 g, 174 mmol) in CH_2Cl_2 (578 mL) was added DMAP (2.12 g, 17.3 mmol) and Et_3N (72.3 mL, 520 mmol) at 0 ^oC, followed by addition of Ac_2O (24.4 mL, 260 mmol) 15 min later. The mixture was stirred at 25 ^oC for 6 h, quenched with $NaHCO_3$ solution (400 mL), extracted with CH_2Cl_2 (3 × 300 mL) and washed with brine (100 mL). The combined organic phases were dried over $MgSO_4$ and concentrated in *vacuo*. Purification of the residue via flash column chromatography on silica gel (petroleum ether / ethyl acetate = 10:1 to 2:1) gave **S1** (53.2 g, 93%) as a white solid.

A round bottle flask was charged with ketone **S1**[1] (50.0 g, 151.3 mmol), 2-picolylamine (34.3 mL, 333 mmol), *p*-TsOH·H_2O (50.0 mg, cat.) and toluene (500 mL). The reaction mixture was heated to reflux under a Dean-Stark apparatus overnight, and then it was diluted with ether (500 mL), sequentially washed with a saturated NH_4Cl solution (500 mL), a saturated $NaHCO_3$ solution (500 mL), and brine (500 mL). The remaining solution was dried over Na_2SO_4, filtered, and concentrated in *vacuo* to give crude imine **S2** as a yellow solid.

To a solution of the above crude imine in THF (250 mL) was added $Cu(NO_3)_2$·$3H_2O$ (40.2 g, 166 mmol). The heterogeneous mixture was stirred vigorously for 30 minutes during which a clear deep blue solution appeared, followed by the formation of a heavy pale blue precipitate. Hydrogen peroxide (30 wt % in water, 77.8 mL, 756 mmol) was then added to the reaction mixture slowly which resulted in the dissolution of the precipitate to give a blue green solution. The reaction mixture was then stirred at 25 ^oC for another 1.5 h. Then ethyl acetate (500 mL) and a saturated Na_4EDTA solution (500 mL) were added, and the reaction mixture was stirred overnight. The layers were separated, and the aqueous layer was extracted with EtOAc (3 × 500 mL). The combined

extracts were dried over Na_2SO_4, filtered, and concentrated in *vacuo*. Purification of the residue via flash column chromatography on silica gel (petroleum ether / ethyl acetate = 10:1 to 1:1) gave alcohol **17** (42.1 g, 80%) as a white solid. The spectroscopic data of **17** were identical with those reported in literature. [1]

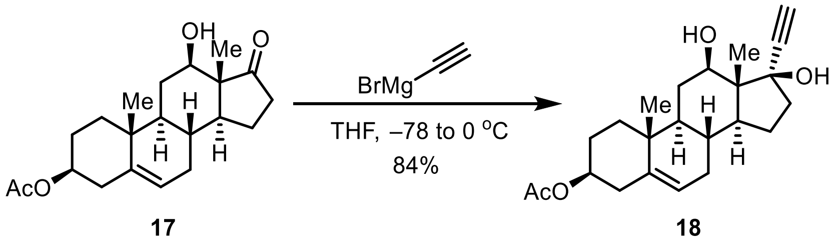

To a mixture of compound **17** (20.0 g, 57.8 mmol) in anhydrous THF (300 mL) were slowly added ethynylmagnesium bromide (0.5 M in THF, 576 mL, 289 mmol) under argon atmosphere at –78 ℃. After being stirred at –78 ℃ for 30 min and stirred at 0 ℃ for 12 h, the reaction was quenched by saturated NH_4Cl solution (800 mL) and extracted with EtOAc (3 × 500 mL). The combined organic phases were washed with brine (2 × 200 mL), dried over Na_2SO_4, filtered and concentrated in *vacuo*. The residue was purified via flash column chromatography on silica gel (petroleum ether / ethyl acetate = 5:1 to 1:1) to give alkyne **18** (18.1 g, 84%) as a white solid.

TLC (petroleum ether / ethyl acetate,1:1 v/v): R_f = 0.50.

OR: $[\alpha]_D^{25}$ = –96.4 (c = 1.67, $CHCl_3$).

IR (neat): ν_{max}: 3425, 3303, 2948, 1724, 1438, 1365, 1248, 1025, 803, 751 cm^{-1}.

^{1}H NMR (400 MHz, $CDCl_3$): δ 5.36 – 5.34 (m, 1H), 4.60 – 4.52 (m, 1H), 4.14 (dd, *J* = 11.2, 4.8 Hz, 1H), 2.92 (s, 1H), 2.61 (s, 1H), 2.41 – 2.19 (m, 4H), 2.03 – 1.95 (m, 5H), 1.89 – 1.79 (m, 2H), 1.77 – 1.68 (m, 2H), 1.62 – 1.33 (m, 6H), 1.16 – 1.04 (m, 2H), 1.02 (s, 3H), 0.89 (s, 3H).

^{13}C NMR (100 MHz, $CDCl_3$): δ 170.6, 139.5, 122.1, 86.9, 79.7, 74.5, 74.2, 73.7, 50.5, 49.4, 49.0, 38.5, 37.9, 36.9, 36.6, 31.5, 31.0, 29.8, 27.6, 22.9, 21.4, 19.2, 7.2.

HRMS (ESI): *m/z* calcd. for $C_{23}H_{33}O_4$ $[M+H]^+$ 373.2373, found 373.2374.

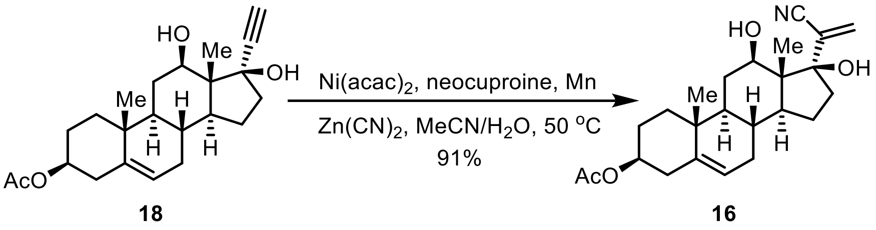

Ni(acac)$_2$ (1.24 g, 4.84 mmol), neocuproine (1.21 g, 5.81 mmol), Zn(CN)$_2$ (4.55 g, 38.7 mmol) and Mn (1.33 g, 24.2 mmol) were placed in a Schlenk flask with a rubber septum and the flask was evacuated and backfilled with argon three times. Degassed MeCN (193 mL) was added and the green-grey suspension was stirred at 25 ℃ for 20 min. Compound **18** (18.0 g, 48.4 mmol) was added, followed by degassed water (39 mL). The reaction mixture was heated to 50 ℃ and stirred for 16 h. After being cooled to 25 ℃, the mixture was diluted with water (200 mL) and MTBE (100 mL), followed by filtration over Celite®. The phases were separated and the aqueous phase was extracted with MTBE (3 × 200 mL). The combined organic phases were washed with water and brine, dried over Na$_2$SO$_4$ and concentrated under reduced pressure. The residue was subjected to flash column chromatography eluting with petroleum ether / ethyl acetate = 5:1 to 2:1 to produce compound **16** (17.6 g, 91%) as a white solid.

Note: Zn(CN)$_2$ is toxic and must be handled with extreme caution.

TLC (petroleum ether / ethyl acetate, 1:1 v/v): R_f = 0.45.

OR: $[\alpha]_D^{25}$ = –43.8 (c = 2.80, CHCl$_3$).

IR (neat): ν_{max}: 3455, 2947, 1719, 1375, 1365, 1250, 1066, 1027, 955, 746, 666 cm^{-1}.

^{1}H NMR (400 MHz, CDCl$_3$): δ 6.15 (s, 1H), 5.93 (s, 1H), 5.40 – 5.27 (m, 1H), 4.65 – 4.42 (m, 1H), 3.62 (dd, *J* = 11.2, 5.2 Hz, 1H), 2.60 (s, 2H), 2.35 – 2.22 (m, 2H), 2.09 – 1.93 (m, 6H), 1.87 – 1.77 (m, 2H), 1.75 – 1.65 (m, 2H), 1.61 – 1.41 (m, 5H), 1.33 – 1.21 (m, 1H), 1.14 – 1.03 (m, 2H), 1.02 (s, 3H), 0.99 (s, 3H).

^{13}C NMR (100 MHz, CDCl$_3$): δ 170.5, 139.4, 131.6, 129.5, 121.9, 118.6, 85.5, 73.6, 73.3, 51.2, 48.7, 48.4, 37.8, 36.8, 36.5, 35.9, 31.4, 31.0, 29.9, 27.5, 23.5, 21.3, 19.1, 9.0.

HRMS (ESI): *m/z* calcd. for $C_{24}H_{34}NO_4$ $[M+H]^+$ 400.2482, found 400.2485.

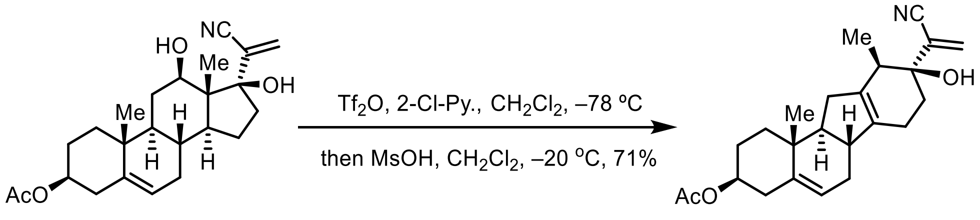

16 **19**

To a solution of compound **16** (12.0 g, 30.0 mmol) in CH_2Cl_2 (300 mL) was added 2-Cl-Py. (21.1 mL, 225 mmol) at –78 °C followed by addition of Tf_2O (15.2 mL, 90.0 mmol) 10 min later. The resultant mixture was stirred at –78 °C for another 2 h. After completion of the reaction (monitored by TLC), MsOH (29.4 mL, 450 mmol) was added to the mixture and stirred at –20 ℃ for 1 h. The reaction was quenched by saturated $NaHCO_3$ (250 mL) and extracted with EtOAc (3 × 250 mL). The combined organic phases were washed with brine (2 × 100 mL), dried over Na_2SO_4, filtered and concentrated in *vacuo*. The residue was purified via flash column chromatography on silica gel (petroleum ether / ethyl acetate = 30:1 to 5:1) to yield alcohol **19** (8.14 g, 71%) as a white foam.

TLC (petroleum ether / ethyl acetate, 2:1 v/v): R_f = 0.53.

OR: $[\alpha]_D^{25}$ = –59.1 (c = 1.67, $CHCl_3$).

IR (neat): ν_{max}: 3483, 2906, 2846, 2222, 1716, 1438, 1355, 1240, 1028, 962, 752 cm^{-1}.

^{1}H NMR (400 MHz, $CDCl_3$): δ 6.21 (d, *J* = 0.8 Hz, 1H), 6.06 (d, *J* = 0.4 Hz, 1H), 5.45 – 5.35 (m, 1H), 4.64 – 4.61 (m, 1H), 2.73 (d, *J* = 5.6 Hz, 1H), 2.46 (t, *J* = 8.4 Hz, 1H), 2.42 – 2.39 (m, 1H), 2.32 –2.27 (m, 1H),, 2.21 – 2.14 (m, 1H), 2.12 – 2.05 (m, 4H), 2.04 (s, 3H), 1.90 – 1.85 (m, 1H), 1.81 – 1.78 (m, 1H), 1.73 (t, *J* = 2.0 Hz, 1H), 1.68 – 1.58 (m, 2H), 1.57 (s, 2H), 1.29 – 1.22 (m, 1H), 1.06 (s, 3H), 0.92 (d, *J* = 4.8 Hz, 3H).

^{13}C NMR (100 MHz, $CDCl_3$): δ 170.5, 141.8, 136.0, 135.2, 129.9, 129.4, 123.0, 117.7, 74.6, 74.0, 56.1, 42.9, 37.9, 37.8, 37.6, 36.9, 33.9, 31.6, 30.2, 27.4, 21.4, 19.8, 19.3, 11.2.

HRMS (ESI): *m/z* calcd. for $C_{24}H_{32}NO_3$ $[M+H]^+$ 382.2377, found 382.2380.

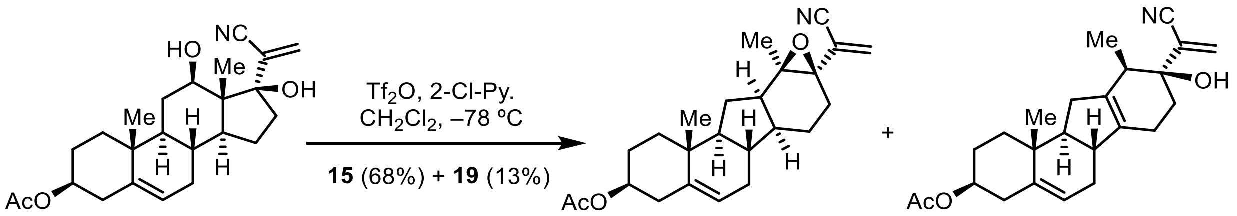

16 **15** **19**

To a solution of compound **16** (6.00 g, 15.0 mmol) in CH_2Cl_2 (750 mL) was added 2-Cl-Py. (10.6 mL, 113 mmol) at –78 °C followed by addition of Tf_2O (7.6 mL, 45.0 mmol) 10 min later. The resultant mixture was stirred at –78 °C for another 2 h before it was quenched by saturated aqueous $NaHCO_3$ (600 mL) and extracted with CH_2Cl_2 (3 × 300 mL). The combined organic layers were washed with brine (100 mL), dried over anhydrous Na_2SO_4, filtered and concentrated in *vacuo*. The residue was purified by column chromatography on silica gel (petroleum ether / ethyl acetate = 40:1 to 3:1 v/v) to furnish **15** (3.90 g, 68%) and **19** (745 mg, 13%) both as white foam.

Compound 15

Melting point: 132–134 ℃

TLC (petroleum ether / ethyl acetate, 5:1 v/v): R_f = 0.50.

OR: $[\alpha]_D^{25}$ = –58.9 (c = 1.80, $CHCl_3$).

IR (neat): ν_{max}: 3020, 2941, 2225, 1729, 1377, 1363, 1244, 1033, 750, 665 cm^{-1}.

^{1}H NMR (400 MHz, $CDCl_3$): δ 6.08 (s, 1H), 5.96 (s, 1H), 5.37 – 5.35 (m, 1H), 4.62 – 4.54 (m, 1H), 2.39 – 2.33 (m, 1H), 2.30 – 2.08 (m, 4H), 2.01 (s, 3H), 1.92 – 1.79 (m, 3H), 1.78 – 1.71 (m, 1H), 1.70 – 1.59 (m, 3H), 1.54 – 1.42 (m, 1H), 1.42 – 1.27 (m, 3H), 1.27 – 1.16 (m, 5H), 0.98 (s, 3H).

^{13}C NMR (100 MHz, $CDCl_3$): δ 170.4, 140.4, 131.4, 123.1, 122.6, 116.9, 73.8, 66.0, 65.8, 53.1, 44.3, 41.1, 40.2, 38.1, 37.5, 36.8, 32.0, 28.0, 27.4, 27.2, 24.6, 21.3, 18.6, 18.4.

HRMS (ESI): *m/z* calcd. for $C_{24}H_{32}NO_3$ $[M+H]^+$ 382.2377, found 382.2380.

*Conversion of **15** into **19***

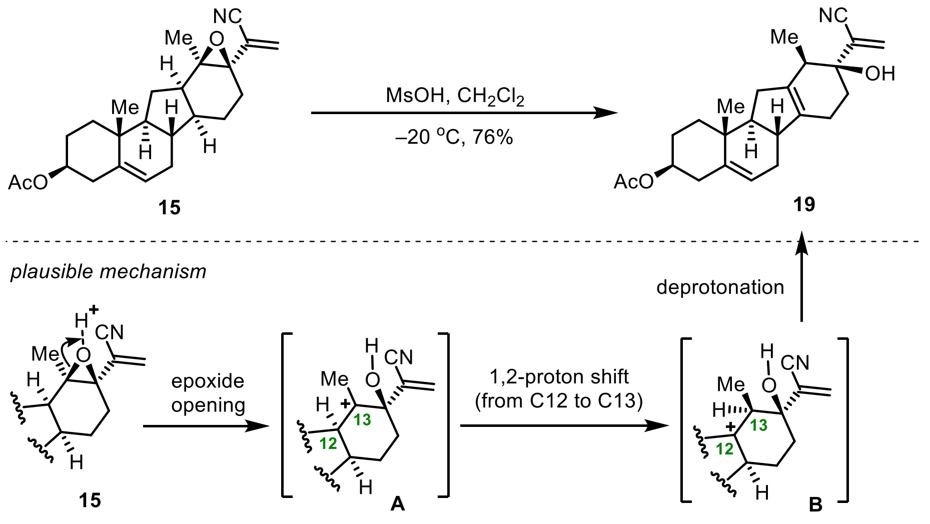

MsOH (51 μL, 0.078 mmol) was slowly added to a solution of epoxy **15** (100 mg, 0.026 mmol) in CH_2Cl_2 (2.5 mL) at –78 ℃ under argon atmosphere and stirred at –20 ℃ for 1 h. The reaction was quenched by saturated $NaHCO_3$ (5 mL) and extracted with CH_2Cl_2 (3 × 5 mL). The combined organic phases were washed with brine (2 × 5 mL), dried over anhydrous Na_2SO_4, filtered and concentrated in *vacuo*. The residue was purified via flash column chromatography on silica gel (petroleum ether / ethyl acetate = 40:1 to 10:1) to afford **19** (76 mg, 76%) as a white foam.

One-step protocol for the conversion of ***19*** *into* ***21***

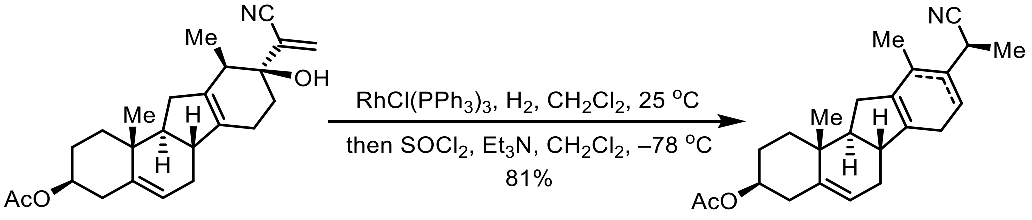

19 **21**

To a stirred solution of olefin **19** (10.0 g, 26.2 mmol) in CH_2Cl_2 (500 mL) was added $RhCl(PPh_3)_3$ (2.43 g, 2.62 mmol). The mixture was stirred under H_2 atmosphere at 25 ℃ for 24 h. After completion of the reaction (monitored by TLC), the mixture was cooled to –78 ℃, to which were sequentially added Et_3N (18.0 mL, 131 mmol) and $SOCl_2$ (4.76 mL, 66.1 mmol). The resulting mixture was stirred at –78 ℃ for 30 min, before being quenched by saturated aqueous $NaHCO_3$ solution at –78 ℃. The mixture was extracted with CH_2Cl_2 (3 × 300 mL), and the combined organic phases were washed with brine (2 × 100 mL), dried over Na_2SO_4, filtered and concentrated in *vacuo*. The crude product was purified by silica gel column chromatography (petroleum ether / ethyl acetate = 20:1 to 10:1) to deliver the product **21** as a mixture of olefins (7.76 g, 81%, 1.2:1) as a white foam.

TLC (petroleum ether / ethyl acetate, 6:1 v/v): R_f = 0.48.

^{1}H NMR (400 MHz, $CDCl_3$, all signals of the two isomers are listed): δ 6.06 – 5.99 (m, 1H), 5.48 – 5.41 (m, 2.2H), 4.68 – 4.57 (m, 2.2H), 3.86 – 3.77 (m, 1H), 3.41 – 3.32 (m, 1H), 2.88 – 2.79 (m, 1H), 2.71 – 2.63 (m, 2H), 2.52 – 2.35 (m, 6H), 2.35 – 2.31 (m, 1H), 2.31 – 2.26 (m, 3H), 2.26 – 2.19 (m, 4H), 2.19 – 2.12 (m, 3H), 2.04 (s, 7H), 2.00 – 1.91 (m, 1H), 1.91 – 1.84 (m, 3H), 1.78 – 1.73 (m, 5H), 1.70 – 1.58 (m, 6H), 1.43 (d, *J* = 7.2 Hz, 3H), 1.36 (d, *J* = 7.2 Hz, 3H), 1.32 – 1.20 (m, 3H), 1.11 – 1.03 (m, 10H).

^{13}**C NMR** (100 MHz, $CDCl_3$, all signals of the two isomers are listed): δ 170.5, 141.7, 141.7, 139.6, 136.3, 136.2, 135.7, 133.7, 127.7, 123.8, 123.1, 123.0, 122.3, 122.0, 121.8, 74.0, 73.9, 56.0, 55.8, 43.2, 42.8, 37.7, 37.7, 37.7, 36.8, 36.8, 33.1, 31.7, 30.6, 30.2, 29.9, 29.4, 27.9, 27.4, 25.6, 24.8, 22.1, 21.4, 19.4, 19.3, 18.7, 18.4, 17.2, 14.4.

HRMS (ESI): *m/z* calcd. for $C_{24}H_{32}NO_3$ $[M+H]^+$ 366.2428, found 366.2430.

*Two-step protocol for the conversion of **19** into **21***

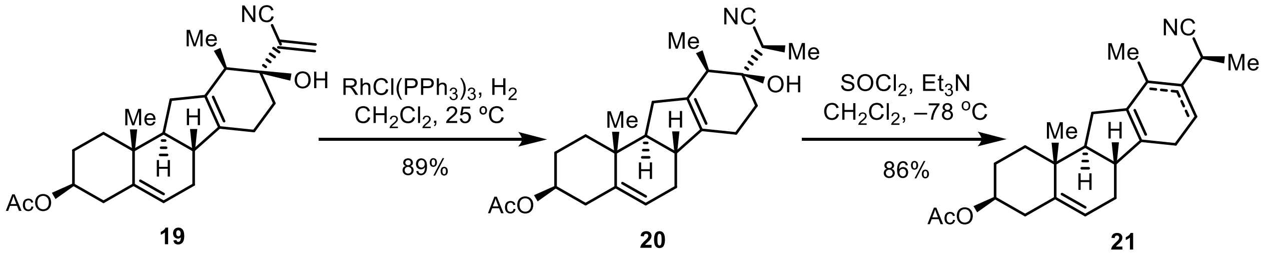

19 **20** **21**

To a stirred solution of olefin **19** (200 mg, 0.524 mmol) in CH_2Cl_2 (10 mL) was added $RhCl(PPh_3)_3$ (48.4 mg, 0.052 mmol). After the mixture was stirred under H_2 atmosphere at 25 ℃ for 24 h, it was concentrated in *vacuo*. The residue was purified via flash column chromatography on silica gel (petroleum ether / ethyl acetate = 10:1 to 1:1) to give compound **20** (180 mg, 89%) as a white foam.

To a solution of **20** (100 mg, 0.261 mmol) in CH_2Cl_2 (5 mL) were sequentially added Et_3N (0.18 mL, 1.30 mmol) and $SOCl_2$ (47 μL, 0.652 mmol) at –78 ℃. The resulting mixture was stirred at the same temperature for 30 min before it was quenched by saturated aqueous $NaHCO_3$ (5 mL) solution. The mixture was extracted with CH_2Cl_2 (3 × 5 mL), and the combined organic phases were washed with brine (2 × 5 mL), dried over Na_2SO_4, filtered and concentrated in *vacuo*. Purification of the crude product by silica gel column chromatography (petroleum ether / ethyl acetate = 20:1 to 10:1 v/v) yielded the product **21** as a mixture of olefins (81.9 mg, 86%, 1.5:1).

Compound 20

TLC (petroleum ether / ethyl acetate, 2:1 v/v): R_f = 0.51.

OR: $[\alpha]_D^{25}$ = –62.2 (c = 1.40, $CHCl_3$).

IR (neat) ν_{max}: 3484, 2916, 2848, 1731, 1439, 1375, 1246, 1212, 1143, 752 cm^{-1}.

^{1}**H NMR** (400 MHz, $CDCl_3$): δ 5.43 – 5.41 (m, 1H), 4.65 – 4.57 (m, 1H), 3.03 (q, *J* = 7.2 Hz, 1H), 2.57 – 2.48 (m, 1H), 2.43 – 2.36 (m, 2H), 2.33 – 2.23 (m, 1H), 2.21 – 2.12 (m, 2H), 2.03 (s, 4H),

2.00 – 1.81 (m, 5H), 1.78 – 1.63 (m, 4H), 1.63 – 1.53 (m, 1H), 1.32 (d, *J* = 7.2 Hz, 3H), 1.29 – 1.17 (m, 1H), 1.05 (s, 3H), 1.01 (d, *J* = 7.2 Hz, 3H).

^{13}C NMR (100 MHz, $CDCl_3$): δ 170.5, 141.7, 137.1, 135.4, 122.9, 121.9, 73.9, 73.6, 56.3, 42.9, 38.3, 37.7, 37.6, 36.8, 34.1, 32.1, 30.0, 27.6, 27.4, 21.4, 20.6, 19.3, 12.9, 12.8.

HRMS (ESI): *m/z* calcd. for $C_{24}H_{34}NO_3$ $[M+H]^+$ 384.2533, found 384.2535.

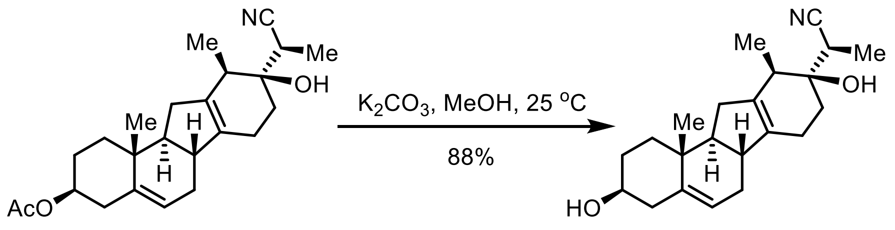

20 **20'**

To a solution of **20** (200 mg, 0.584 mmol) in MeOH (10 mL) was added K_2CO_3 (404 mg, 2.92 mmol) at 25 °C. After TLC indicated disappearance of the starting material, the reaction solution was concentrated in *vacuo*. The residue was purified via flash column chromatography on silica gel (petroleum ether / ethyl acetate = 4:1 to 2:1) to afford compound **20'** (157 mg, 88%) as a white solid.

Melting point: 183–185 ℃

TLC (petroleum ether / ethyl acetate, 2:1 v/v): R_f = 0.46.

OR: $[\alpha]_D^{25}$ = –97.0 (c = 0.53, $CHCl_3$).

IR (neat): ν_{max}: 3415, 2928, 2843, 2243, 1440, 1374, 1049, 981, 813, 749, 665, 541 cm^{-1}.

^{1}H NMR (400 MHz, $CDCl_3$): δ 5.41 – 5.39 (m, 1H), 3.53 (dd, *J* = 10.8, 4.4 Hz, 1H), 3.03 (q, *J* = 7.2 Hz, 1H), 2.52 (d, *J* = 7.6 Hz, 1H), 2.47 – 2.31 (m, 2H), 2.27 – 2.12 (m, 3H), 2.11 – 1.93 (m, 3H), 1.92 – 1.83 (m, 2H), 1.81 (s, 1H), 1.75 – 1.69 (m, 2H), 1.67 – 1.63 (m, 1H), 1.62 – 1.51 (m, 3H), 1.32 (d, *J* = 7.2 Hz, 3H), 1.18 (dd, *J* = 13.6, 4.0 Hz, 1H), 1.08 – 0.97 (m, 6H).

^{13}C NMR (100 MHz, $CDCl_3$): δ 142.9, 137.1, 135.5, 122.0, 121.9, 73.6, 71.9, 56.5, 43.0, 41.8, 38.4, 37.8, 36.7, 34.1, 32.2, 31.3, 30.0, 27.6, 20.7, 19.4, 13.0, 12.9.

HRMS (ESI): *m/z* calcd. for $C_{22}H_{32}NO_2$ $[M+H]^+$ 342.2428, found 342.2429.

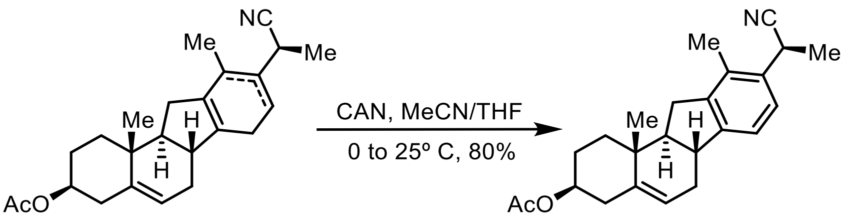

21 **22**

Ceric ammonium nitrate (26.1 g, 49.2 mmol) and **21** (6.00 g, 16.4 mmol) were placed in a Schlenk flask with a rubber septum and the flask was evacuated and backfilled with argon three times. MeCN/THF (4:1, 330 mL) was added and the mixture was stirred at 25 ℃ for 1 h. Water was added at 0 ℃ to quench the reaction, and the mixture was extracted with EtOAc (3 × 200 mL). The organic phases were combined, dried with anhydrous Na_2SO_4, filtered and concentrated. The crude product was purified by silica gel column chromatography (petroleum ether / ethyl acetate = 40:1 to 10:1) to yield **22** (4.83 g, 80%) as a white foam.

TLC (petroleum ether / ethyl acetate, 6:1 v/v): R_f = 0.46.

OR: $[\alpha]_D^{25}$ = –67.8 (c = 1.90, $CHCl_3$).

IR (neat): ν_{max}: 2936, 2241, 1728, 1438, 1375, 1364, 1240, 1028, 961, 812, 752, 666 cm^{-1}.

^{1}H NMR (400 MHz, $CDCl_3$): δ 7.29 (d, *J* = 7.6 Hz, 1H), 7.04 (d, *J* = 7.6 Hz, 1H), 5.54 – 5.51 (m, 1H), 4.72 – 4.60 (m, 1H), 4.06 (q, *J* = 7.2 Hz, 1H), 2.98 (td, *J* = 12.0, 5.6 Hz, 1H), 2.80 (dd, *J* = 14.8, 7.2 Hz, 1H), 2.65 – 2.57 (m, 2H), 2.49 – 2.43 (m, 1H), 2.41 – 2.31 (m, 1H), 2.28 (s, 3H), 2.08 – 1.99 (m, 4H), 1.97 – 1.80 (m, 3H), 1.76 – 1.65 (m, 1H), 1.60 (d, *J* = 7.2 Hz, 3H), 1.39 – 1.26 (m, 1H), 1.16 (s, 3H).

^{13}C NMR (100 MHz, $CDCl_3$): δ 170.4, 146.1, 143.6, 141.3, 133.2, 130.7, 125.0, 122.7, 122.1, 120.8, 73.7, 56.9, 41.1, 37.7 (×2), 36.9, 30.2, 30.1, 28.1, 27.4, 21.4, 20.3, 19.1, 15.3.

HRMS (ESI): *m/z* calcd. for $C_{24}H_{30}NO_2$ $[M+H]^+$ 364.2271, found 364.2273.

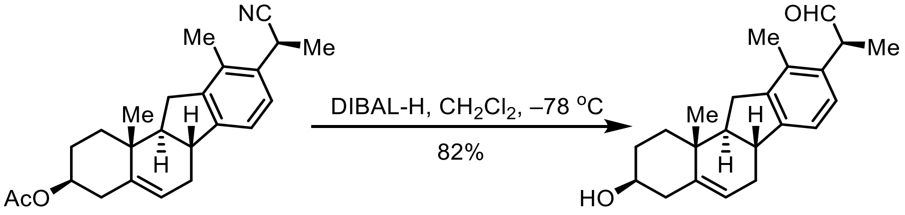

22 **23**

To a mixture of **22** (5.00 g, 13.8 mmol) in anhydrous CH_2Cl_2 (275 mL) were slowly added DIBAL-H (1.0 M in THF, 55 ml, 55.0 mmol) under argon atmosphere at –78 ℃. After being stirred at –

78 ℃ for 4 h, the reaction was quenched by dropwise addition of EtOAc (10 mL). The cooling bath was removed, and saturated Rochelle Salt solution (130 mL), H_2O (130 mL) and HCl (10 mL, 2.0 M in H_2O) were added. The mixture was stirred at 25 ℃ until two clear layers formed. The organic phase was separated and the aqueous phase was extracted with CH_2Cl_2 (3 × 200 mL). The combined organic phase was dried over anhydrous Na_2SO_4 and filtered. The solvents were removed under reduced pressure and the residue was purified by silica gel chromatography (petroleum ether / ethyl acetate = 8:1 to 1:1) to furnish aldehyde **23** (3.66 g, 82%) as a white foam.

TLC (petroleum ether / ethyl acetate, 2:1 v/v): R_f = 0.39.

OR: $[\alpha]_D^{25}$ = –14.3 (c = 0.55, $CHCl_3$).

IR (neat): ν_{max}: 3411, 2931, 1721, 1460, 1437, 1350, 1196, 1052, 1015, 810 cm^{-1}.

^{1}H NMR (400 MHz, CD_3CN): δ 9.61 (s, 1H), 6.99 (d, *J* = 7.6 Hz, 1H), 6.83 (d, *J* = 7.6 Hz, 1H), 5.49 – 5.46 (m, 1H), 3.90 (q, *J* = 6.8 Hz, 1H), 3.42 – 3.35 (m, 1H), 2.96 (td, *J* = 12.0, 5.6 Hz, 1H), 2.88 – 2.79 (m, 2H), 2.67 – 2.54 (m, 2H), 2.35 – 2.30 (m, 1H), 2.24 (s, 3H), 2.23 – 2.18 (m, 1H), 2.16 – 2.12 (m, 1H), 1.87 – 1.73 (m, 3H), 1.59 – 1.47 (m, 1H), 1.31 (d, *J* = 7.2 Hz, 3H), 1.26 – 1.22 (m, 1H), 1.14 (s, 3H).

^{13}C NMR (100 MHz, CD_3CN): δ 202.3, 146.2, 144.5, 144.1, 135.6, 133.4, 126.6, 121.8, 121.1, 71.8, 58.0, 49.4, 42.4, 41.8, 38.6, 37.5, 31.8, 30.8, 30.7, 19.4, 15.6, 14.7.

HRMS (ESI): *m/z* calcd. for $C_{22}H_{29}O_2$ $[M+H]^+$ 325.2162, found 325.2160.

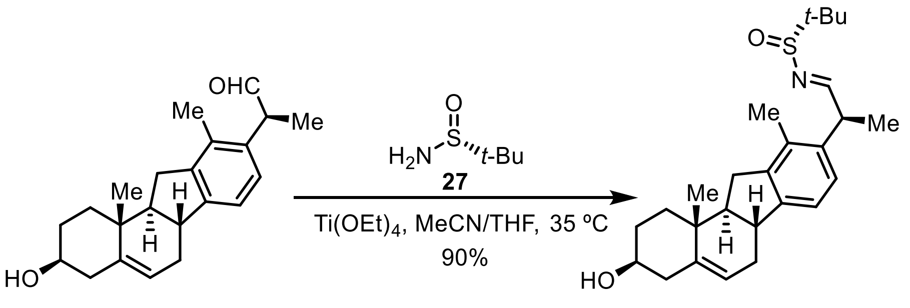

23 **12**

To a solution of **23** (3.00 g, 9.25 mmol) and (*R*)-*tert*-butanesulfinamide (**27**, 1.34 g, 11.1 mmol) in MeCN/THF (1:4, 185 mL) was added titanium ethoxide (9.7 ml, 46.2 mmol) under argon at 0 ℃. The reaction was stirred at 35 ℃ until aldehyde **23** was completely consumed as monitored by TLC. Then the reaction was poured into ice brine (200 mL) and stirred at 25 ℃ for 30 min. The white suspension was filtered through Celite®. The organic layer was separated and the aqueous phase

was extracted with EtOAc (3 × 150 mL), and the combined organics were washed with brine (80 mL), dried over Na_2SO_4, filtered and concentrated in *vacuo*. The crude product was purified by silica gel column chromatography (dichloromethane / ethyl acetate / ether = 80:1:1 to 20:1:1) to deliver the product **12** (3.55 g, 90%) as a white foam.

TLC (dichloromethane / ethyl acetate / ether, 20:1:1 v/v/v): R_f = 0.40.

OR: $[\alpha]_D^{25}$ = –170.9 (c = 1.00, $CHCl_3$).

IR (neat): ν_{max}: 3392, 2930, 1616, 1456, 1363, 1240, 1186, 1057, 1015, 810, 751, 665 cm^{-1}.

^{1}H NMR (400 MHz, $CDCl_3$): δ 8.09 (d, *J* = 4.0 Hz, 1H), 7.00 – 6.95 (m, 2H), 5.49 – 5.47 (m, 1H), 4.13 – 4.08 (m, 1H), 3.61 – 3.54 (m, 1H), 2.98 (td, *J* = 11.6, 5.2 Hz, 1H), 2.79 (dd, *J* = 14.8, 7.2 Hz, 1H), 2.68 – 2.53 (m, 2H), 2.45 – 2.40 (m, 1H), 2.30 – 2.22 (m, 4H), 2.07 – 1.96 (m, 1H), 1.90 – 1.80 (m, 2H), 1.75 (s, 1H), 1.67 – 1.55 (m, 1H), 1.49 (d, *J* = 6.8 Hz, 3H), 1.31 – 1.23 (m, 2H), 1.19 (s, 9H), 1.14 (s, 3H).

^{13}C NMR (100 MHz, $CDCl_3$): δ 171.0, 145.1, 143.4, 142.4, 136.9, 131.8, 125.4, 121.9, 120.5, 71.7, 57.0, 56.9, 41.9 (×2), 41.2, 38.0, 36.9, 31.3, 30.3 (×2), 22.4 (×3), 19.2, 17.7, 15.6.

HRMS (ESI): *m/z* calcd. for $C_{26}H_{38}NO_2S$ $[M+H]^+$ 428.2618, found 428.2616.

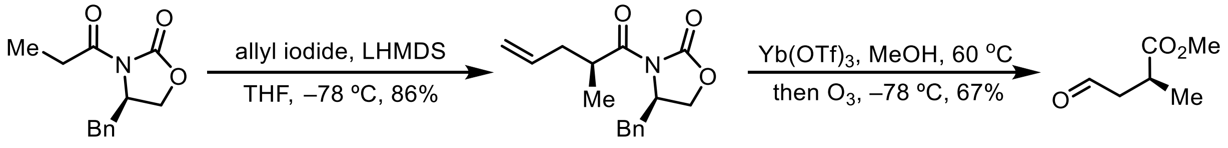

S3 **S4** **14**

To a solution of (*R*)-(–)-4-benzyl-3-propionyl-2-oxazolidinone **S3** (30 g, 128 mmol) in THF (300 mL) at –78 ℃ was added LHMDS (1.0 M in THF, 193 mL, 193 mmol). After the reaction mixture was stirred at the same temperature for 2 h, allyl iodide (36 mL, 386 mmol) was added and the resulting solution was stirred at –78 ℃ for 10 h. The reaction was allowed to warm to 25 °C and stirred for another 30 min before being quenched with saturated NH_4Cl solution (300 mL). The two phases were separated and the aqueous phase was extracted with EtOAc (3 × 250 mL). The combined organic phases were washed with brine, dried over Na_2SO_4 and concentrated in *vacuo*. The crude residue was purified by flash chromatography on silica gel (petroleum ether / ethyl acetate = 20:1 to 5:1) to give compound **S4**[2] (30.2 g, 86%) as a colorless oil.

Yb(OTf)$_3$ (6.92 g, 11.0 mmol) and **S4** (30.0 g, 110 mmol) were placed in a flask, to which was added anhydrous MeOH (548 mL) under argon. After the mixture was stirred at 60 ℃ for 30 min, it was cooled to –78 ℃. O_3 was bubbled through the stirred solution until a blue color persisted. The solution was purged until it became colorless, to which was added a solution of PPh_3 (57.6 g, 220 mmol) in CH_2Cl_2 (200 mL). The reaction was allowed to warm to 25 ℃ and stirred for 6 h, concentrated in *vacuo* at 20 ℃. The crude product was purified by silica gel column chromatography (petroleum ether / ethyl acetate = 100:1 to 10:1) to generate aldehyde **14** (9.57 g, 67%) as a colorless oil. The spectroscopic data of **14** were identical with those reported in literature.[3]

TLC (petroleum ether / ethyl acetate, 5:1 v/v): R_f = 0.42.

OR: $[\alpha]_D^{25}$ = +4.1 (c = 0.70, $CHCl_3$).

IR (neat): ν_{max}: 2955, 1730, 1436, 1379, 1196, 1174, 1137, 989, 772 cm^{-1}.

^{1}H NMR (400 MHz, $CDCl_3$): δ 9.76 (s, 1H), 3.69 (s, 3H), 3.04 – 2.86 (m, 2H), 2.54 (ddd, *J* = 17.6, 5.6, 0.8 Hz, 1H), 1.23 (d, *J* = 6.8 Hz, 3H).

^{13}C NMR (100 MHz, $CDCl_3$): δ 200.0, 175.6, 52.0, 46.9, 33.5, 17.0.

HRMS (ESI): *m/z* calcd. for $C_{23}H_{33}O_4$ $[M+H]^+$ 131.0703, found 131.0700.

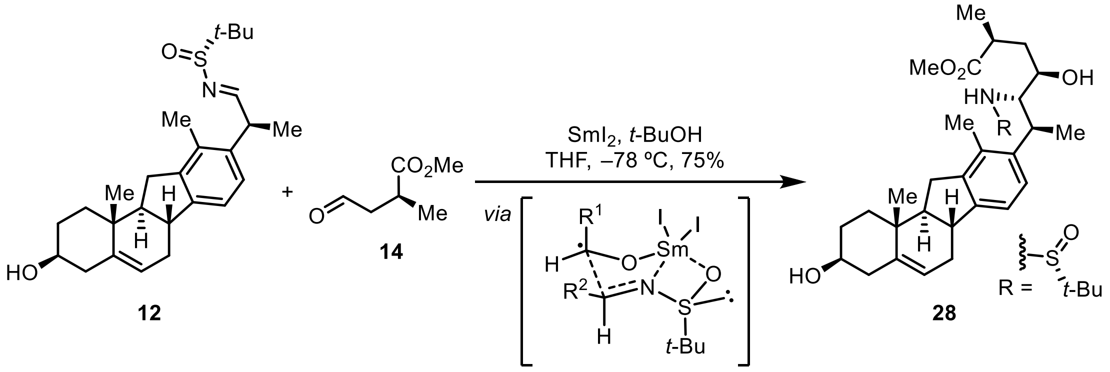

A mixture of **12** (3.50 g, 8.17 mmol), **14** (3.19 g, 24.6 mmol), and *t*-BuOH (3.13 mL, 32.7 mmol) in THF (250 mL) was added dropwise to a solution of SmI_2 (0.1 M in THF, 281 ml, 28.1 mmol) at –78 ℃ and stirred at the same temperature overnight. After the reaction was quenched with saturated aqueous $Na_2S_2O_3$ (250 mL), the resultant mixture was extracted with EtOAc (3 × 300 mL), and the combined organic phases were washed with brine (2 × 100 mL), dried over Na_2SO_4, filtered and concentrated in *vacuo*. The crude product was purified by silica gel column chromatography (dichloromethane / methanol = 100:1 to 20:1) to yield the product **28** (3.43 g, 75%) as a white foam.

Note: The SmI_2 solution (0.1 M in THF) employed in the reductive coupling could be purchased from a commercial source or prepared from Sm and I_2 using a well-known procedure.[4] *When purchasing on large scale (> 250 g), the price of samarium metal granules is around $0.15/gram from Huizhou Boguan Vacuum Applied Materials Co., Ltd. (http://www.bgvmat.com/, accessed on May 15, 2024).*

TLC (dichloromethane / methanol, 20:1 v/v): $R_f = 0.42$.

OR: $[\alpha]_D^{25} = -34.6$ (c = 0.43, $CHCl_3$).

IR (neat): ν_{max}: 3313, 2931, 1732, 1460, 1364, 1260, 1219, 1171, 1043, 771 cm^{-1}.

^{1}H NMR (400 MHz, $CDCl_3$) : δ 7.11 (d, *J* = 8.0 Hz, 1H), 7.00 (d, *J* = 7.6 Hz, 1H), 5.51 – 5.45 (m, 1H), 3.75 – 3.70 (m, 1H), 3.69 (s, 3H), 3.62 – 3.48 (m, 3H), 3.48 – 3.43 (m, 1H), 2.99 – 2.89 (m, 1H), 2.88 – 2.74 (m, 3H), 2.62 – 2.52 (m, 2H), 2.46 – 2.38 (m, 1H), 2.32 – 2.25 (m, 4H), 2.06 – 1.93 (m, 2H), 1.92 – 1.82 (m, 3H), 1.78 – 1.68 (m, 2H), 1.68 – 1.58 (m, 2H), 1.36 (d, *J* = 6.4 Hz, 3H), 1.22 (d, *J* = 6.8 Hz, 3H), 1.14 (s, 3H), 1.01 (s, 9H).

^{13}C NMR (100 MHz, $CDCl_3$): δ 177.6, 144.5, 143.3, 142.4, 139.1, 132.4, 125.7, 122.1, 120.5, 71.8, 70.5, 65.2, 57.0, 56.5, 51.9, 41.9, 41.3, 38.1, 36.9, 36.3, 36.0, 35.3, 31.4, 30.6, 30.4, 22.7 (×3), 19.6, 19.3, 18.4, 16.0.

HRMS (ESI): *m/z* calcd. for $C_{32}H_{50}NO_5S$ $[M+H]^+$ 560.3404, found 560.3405.

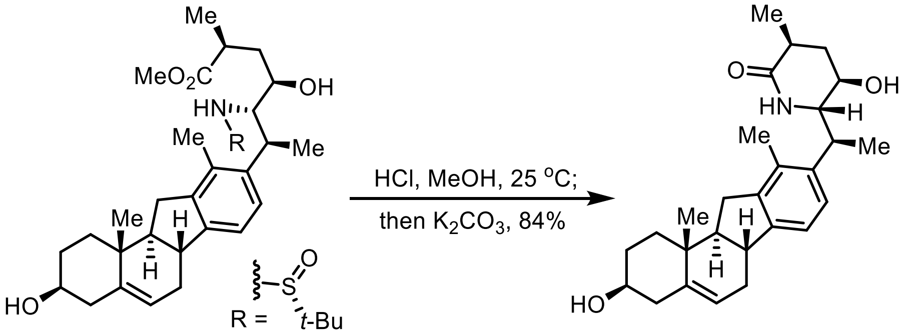

28 **29**

HCl (2.0 M in ether, 42.9 mL, 85.7 mmol) was added to a stirred solution of compound **28** (3.20 g, 5.72 mmol) in MeOH (100 mL) at 0 ℃. The mixture was stirred at 25 ℃ for 5 h and then concentrated under reduced pressure. The residue was dissolved in MeOH (100 mL), to which was added K_2CO_3 (3.95 g, 28.6 mmol) at 25 ℃. After been stirred overnight, the solvent was concentrated. Purification of the crude product by silica gel column chromatography

[dichloromethane (containing 0.5% ammonia) / methanol = 80:1 to 10:1] gave amide **29** (2.04 g, 84%) as a white solid.

TLC (dichloromethane / methanol, 20:1 v/v): R_f = 0.40.

OR: $[\alpha]_D^{25}$ = –12.9 (c =1.27, $CHCl_3$).

IR (neat): ν_{max}: 3533, 2931, 1631, 1455, 1255,1047,1034, 801, 748, 664 cm^{-1}.

^{1}H NMR (400 MHz, CD_3OD): δ 7.08 (d, *J* = 7.6 Hz, 1H), 6.95 (d, *J* = 8.0 Hz, 1H), 5.52 – 5.45 (m, 1H), 3.70 – 3.62 (m, 1H), 3.50 – 3.38 (m, 3H), 2.92 (td, *J* = 11.6, 5.2 Hz, 1H), 2.80 (dd, *J* = 14.8, 7.6 Hz, 1H), 2.66 – 2.54 (m, 2H), 2.42 – 2.33 (m, 1H), 2.30 – 2.20 (m, 4H), 2.17 – 2.07 (m, 1H), 2.05 – 1.71 (m, 5H), 1.68 – 1.54 (m, 2H), 1.34 (d, *J* = 6.4 Hz, 3H), 1.29 – 1.22 (m, 1H), 1.18 – 1.11 (m, 6H).

^{13}C NMR (100 MHz, CD_3OD): δ 177.5, 145.5, 144.0, 144.0, 139.9, 133.6, 126., 122.80, 121.2, 72.5, 68.5, 65.6, 58.7, 42.6, 42.4, 39.2, 38.7, 38.5, 38.0, 34.4, 32.0, 31.5, 31.4, 19.6, 19.4, 17.3, 16.0.

HRMS (ESI): *m/z* calcd. for $C_{27}H_{38}NO_3$ $[M+H]^+$ 424.2846, found 424.2847.

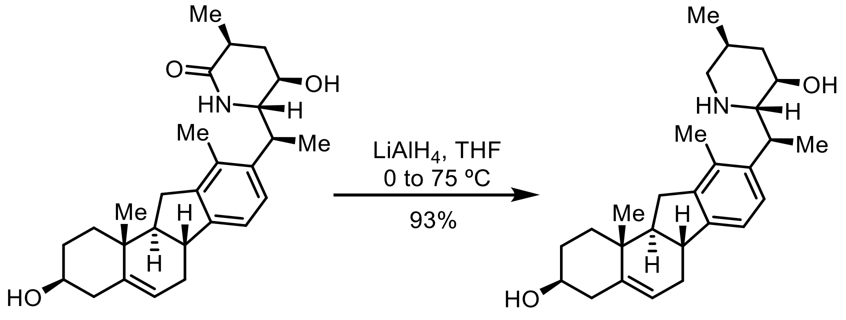

Lithium aluminum hydride (2.5 M in THF, 9.5 mL, 23.6 mmol) was added to a solution of **29** (2.00 g, 4.73 mmol) in anhydrous THF (95 mL) at 0 ℃. The resulting mixture was heated at 75 ℃ for 4 h. After the reaction was cooled to ambient temperature, it was quenched with saturated potassium sodium tartrate and potassium carbonate solution. The resultant mixture was extracted with EtOAc (5 × 100 mL), and the combined organic extracts were washed with water (50 mL) and brine (50 mL), dried over Na_2SO_4, filtered and concentrated in *vacuo*. The crude product was purified by silica gel column chromatography [dichloromethane (containing 0.5% ammonia) / methanol = 80:1 to 10:1] to afford (–)-veratramine (**1**, 1.79 g, 93%) as a white solid.

TLC (dichloromethane / methanol, 10:1 v/v): R_f = 0.46.

OR: $[\alpha]_D^{20}$ = –55.2 (c = 0.29, MeOH).

IR (neat): ν_{max}: 3314, 2929, 2904, 2843, 1646, 1456, 1266, 1032, 813, 629 cm^{-1}.

^{1}H NMR (400 MHz, C_5D_5N): δ 7.69 (d, *J* = 7.6 Hz, 1H), 7.11 (d, *J* = 7.6 Hz, 1H), 5.52 – 5.43 (m, 1H), 4.13 – 4.02 (m, 1H), 3.90 – 3.80 (m, 1H), 3.60 – 3.47 (m, 1H), 3.02 – 2.88 (m, 2H), 2.83 (dd, *J* = 8.8, 4.0 Hz, 1H), 2.81 – 2.74 (m, 1H), 2.74 – 2.68 (m, 1H), 2.66 – 2.59 (m, 1H), 2.58 (s, 3H), 2.56 – 2.52 (m, 1H), 2.52 – 2.42 (m, 1H), 2.29 – 2.22 (m, 1H), 2.22 – 2.15 (m, 1H), 2.15 – 1.99 (m, 2H), 1.94 – 1.85 (m, 1H), 1.85 – 1.80 (m, 1H), 1.80 – 1.75 (m, 1H), 1.63 (d, *J* = 7.2 Hz, 3H), 1.53 – 1.41 (m, 1H), 1.41 – 1.26 (m, 3H), 1.10 (s, 3H), 0.74 (d, *J* = 6.4 Hz, 3H).

^{13}C NMR (100 MHz, C_5D_5N): δ143.7, 143.6, 142.7, 141.2, 133.1, 126.7, 121.5, 119.9, 71.3, 70.7, 68.4, 57.5, 54.7, 45.3, 43.0, 41.5, 38.5, 37.2, 35.6, 32.6, 32.2, 30.8, 30.7, 21.1, 19.3, 19.1, 16.2.

HRMS (ESI): *m/z* calcd. for $C_{27}H_{40}NO_2$ $[M+H]^+$ 410.3054, found 410.3052.

Supplementary Table 1. Comparison of ^{1}H NMR spectroscopic data of natural and synthetic (–)-veratramine (1)

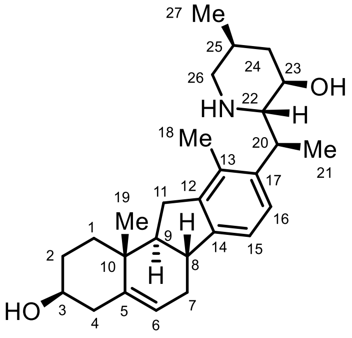

(–)-veratramine (**1**)

No.	**Natural**[6] δ ^{1}H [ppm; mult; *J* (Hz)] 400 MHz, C_5D_5N	**Gao's synthetic**[5] δ ^{1}H [ppm; mult; *J* (Hz)] 300 MHz, C_5D_5N	**Our synthetic** δ ^{1}H [ppm; mult; *J* (Hz)] 400 MHz, C_5D_5N
1	1.31 (1H, td, 13.5, 4.0)<br>1.80 (1H, br d, 13.5)	1.32 – 1.25 (1H, overlap)<br>1.86 – 1.80 (1H, overlap)	1.33 – 1.25 (1H, m)<br>1.85 – 1.80 (1H, m)
2	1.88 (1H, dddd, 13.5, 12.0, 11.0, 3.5)<br>2.12 (1H, br d, 12.0)	1.95 – 1.86 (1H, overlap)<br>2.15 – 2.07 (1H, overlap)	1.94 – 1.85 (1H, m)<br>2.15 – 2.06 (1H, m)
3	3.84 (1H, tt, 11.0, 4.0)	3.84 (1H, tt, 10.4, 4.5)	3.85 (1H, t, 12.4, 6.8)
4	2.60 (1H, br dd, 13.0, 11.0)<br>2.71 (1H, br dd, 13.0, 4.0)	2.65 – 2.59 (1H, overlap)<br>2.69 (1H, dd, 13.0, 4.0)	2.66 – 2.59 (1H, m)<br>2.68 (1H, m)
6	5.40 (1H, br d, 4.0)	5.52 – 5.44 (1H, m)	5.52 – 5.43 (1H, m)
7	2.02 (1H, br dd, 12.0)<br>2.56 (1H, m)	2.06 – 1.97 (1H, overlap)<br>2.56 – 2.52 (1H, overlap)	2.07 – 1.99 (1H, m)<br>2.56 – 2.52 (1H, m)
8	2.95 (1H, td, 12.0, 5.0)	3.03 – 2.87 (1H, overlap)	3.02 – 2.91 (1H, m)
9	1.80 (1H, td, 12.0, 7.0)	1.79 – 1.74 (1H, overlap)	1.80 – 1.75 (1H, m)
11	2.50 (1H, dd, 14.5, 12.0)<br>2.78 (1H, dd, 14.5, 7.0)	2.52 – 2.43 (1H, overlap)<br>2.80 – 2.74 (1H, overlap)	2.52 – 2.42 (1H, m)<br>2.81 – 2.74 (1H, m)
15	7.10 (1H, d, 7.5)	7.11 (1H, d, 7.8)	7.11 (1H, d, 7.6)
16	7.66 (1H, d, 7.5)	7.68 (1H, d, 7.8)	7.69 (1H, d, 7.6)
18	2.57 (3H, s)	2.57 (3H, s)	2.58 (3H, s)
19	1.11 (3H, s)	1.10 (3H, s)	1.10 (3H, s)
20	4.06 (1H, qd, 7.0, 4.0)	4.06 (1H, qd, 7.2, 4.1)	4.08 (1H, qd, 7.2, 4.0)
21	1.64 (3H, d, 7.0)	1.63 (3H, d, 7.2)	1.63 (3H, d, 7.2)
22	2.84 (1H, dd, 9.0, 4.0)	2.83 (1H, dd, 8.9, 3.9)	2.83 (1H, dd)
23	3.56 (1H, ddd, 11.0, 9.0, 4.5)	3.60 – 3.55 (1H, m)	3.60 – 3.47 (1H, m)
24	1.35 (1H, q, 11.0)<br>2.25 (1H, br d 11.0)	1.42 – 1.33 (1H, overlap)<br>2.31 – 2.23 (1H, overlap)	1.41 – 1.33 (1H, m)<br>2.29 – 2.22 (1H, m)
25	1.48 (1H, m)	1.54 – 1.41 (1H, m)	1.53 – 1.41 (1H, m)
26	2.20 (1H, t, 11.5)<br>2.99 (1H, br d, 11.5)	2.22 – 2.15 (1H, overlap)<br>3.03 – 2.87 (1H, overlap)	2.22 – 2.15 (1H, m)<br>3.02 – 2.91 (1H, m)
27	0.74 (3H, d, 6.5)	0.74 (3H, d, 6.3)	0.74 (3H, d, 6.4)

Supplementary Table 2. Comparison of ^{13}C NMR spectroscopic data of natural and synthetic (–)-veratramine (1)

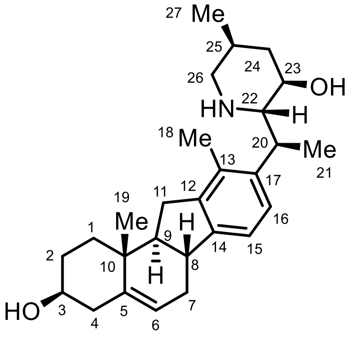

(–)-veratramine (**1**)

position	Natural[6] δ ^{13}C (ppm) 100 MHz, C_5D_5N	Gao's synthetic[5] δ ^{13}C (ppm) 125 MHz, C_5D_5N	Our synthetic δ ^{13}C (ppm) 100 MHz, C_5D_5N	Deviation Δδ (ppm)	
				Natural	Gao's synthetic
1	38.5	38.5	38.5	0	0
2	32.1	32.2	32.2	–0.1	0
3	71.3	71.2	71.3	0	–0.1
4	42.9	43.0	43.0	–0.1	0
5	142.7	142.7	142.7	0	0
6	121.5	121.5	121.5	0	0
7	30.8	30.8	30.8	0	0
8	41.5	41.5	41.5	0	0
9	57.5	57.5	57.5	0	0
10	37.2	37.2	37.2	0	0
11	30.7	30.7	30.7	0	0
12	133.1	133.1	133.1	0	0
13	143.7	143.6	143.6	0.1	0
14	143.7	143.7	143.7	0	0
15	119.9	119.9	119.9	0	0
16	126.7	126.7	126.7	0	0
17	141.2	141.2	141.2	0	0
18	16.1	16.1	16.2	–0.1	–0.1
19	19.3	19.3	19.3	0	0
20	35.7	35.6	35.6	0.1	0
21	21.1	21.1	21.1	0	0
22	68.3	68.4	68.4	–0.1	0
23	70.7	70.6	70.7	0	–0.1
24	45.2	45.3	45.3	–0.1	0
25	32.5	32.6	32.6	–0.1	0
26	54.6	54.6	54.7	–0.1	–0.1
27	19.0	19.1	19.1	–0.1	0

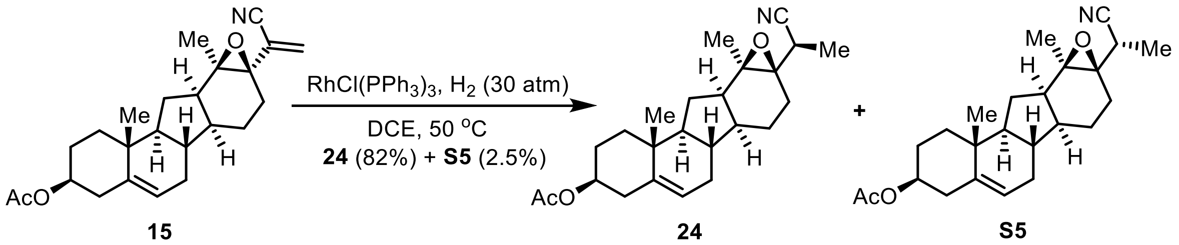

To a stirred solution of olefin **15** (8.00 g, 21.0 mmol) in DCE (175 mL) was added $RhCl(PPh_3)_3$ (1.94 g, 2.10 mmol). After the mixture was stirred under H_2 (30 atm) at 50 ℃ for 10 h, it was concentrated in *vacuo*. The residue was purified via flash column chromatography on silica gel (petroleum ether / ethyl acetate = 10:1 to 2:1) to give compounds **24** (6.60 g, 82%) and **S5** (201 mg, 2.5%) both as a white solid.

Compound 24

TLC (petroleum ether / ethyl acetate, 3:1 v/v): R_f = 0.45.

OR: $[\alpha]_D^{25}$ = –30.1 (c = 0.74, $CHCl_3$).

IR (neat): ν_{max}: 3017, 2940, 2858, 2240, 1990, 1730, 1648, 1448, 1373, 1245, 1032, 756 cm^{-1}.

^{1}H NMR (400 MHz, $CDCl_3$): δ 5.40 – 5.33 (m, 1H), 4.65 – 4.53 (m, 1H), 2.81 (d, *J* = 7.6 Hz, 1H), 2.40 – 2.33 (m, 1H), 2.32 – 2.22 (m, 1H), 2.22 – 2.18 (m, 1H), 2.18 – 2.14 (m, 1H), 2.14 – 2.08 (m, 1H), 2.03 (s, 3H), 1.91 – 1.75 (m, 4H), 1.72 – 1.60 (m, 3H), 1.45 – 1.42 (m, 1H), 1.42 – 1.40 (m, 1H), 1.39 – 1.35 (m, 4H), 1.33 (s, 3H), 1.27 – 1.13 (m, 3H), 0.99 (s, 3H).

^{13}C NMR (100 MHz, $CDCl_3$): δ 170.5, 140.4, 123.1, 120.5, 73.9, 64.9, 64.7, 53.2, 43.9, 41.8, 40.8, 38.1, 37.5, 36.8, 32.1, 31.1, 27.9, 27.4, 24.4, 23.6, 21.4, 18.7, 18.5, 14.8.

HRMS (ESI): *m/z* calcd. for $C_{24}H_{34}NO_3$ $[M+H]^+$ 384.2533, found 384.2535.

Compound S5

TLC (petroleum ether / ethyl acetate, 5:1 v/v): R_f = 0.46.

OR: $[\alpha]_D^{25}$ = –72.4 (c = 0.69, $CHCl_3$).

IR (neat): ν_{max}: 3023, 2941, 2242, 1728, 1452, 1371, 1244, 1030, 905, 810, 752 cm^{-1}.

^{1}H NMR (400 MHz, $CDCl_3$): δ 5.46 – 5.28 (m, 1H), 4.68 – 4.52 (m, 1H), 2.78 (q, *J* = 7.2 Hz, 1H), 2.42 – 2.32 (m, 1H), 2.32 – 2.26 (m, 1H), 2.26 – 2.20 (m, 1H), 2.20 – 2.13 (m, 1H), 2.02 (s, 3H), 2.01 – 1.94 (m, 1H), 1.94 – 1.82 (m, 3H), 1.82 – 1.75 (m, 1H), 1.74 – 1.60 (m, 3H), 1.53 – 1.45 (m, 1H), 1.42 (d, *J* = 7.2 Hz, 3H), 1.40 (s, 3H), 1.38 – 1.16 (m, 5H), 0.98 (s, 3H).

^{13}C NMR (100 MHz, $CDCl_3$): δ 170.5, 140.5, 123.2, 120.3, 73.9, 65.4, 64.9, 53.2, 43.9, 41.6, 40.4, 38.1, 37.5, 36.8, 32.1, 28.1, 27.8, 27.5, 24.5, 22.7, 21.4, 19.1, 18.5, 13.4.

HRMS (ESI): *m/z* calcd. for $C_{24}H_{34}NO_3$ $[M+H]^+$ 384.2533, found 384.2537.

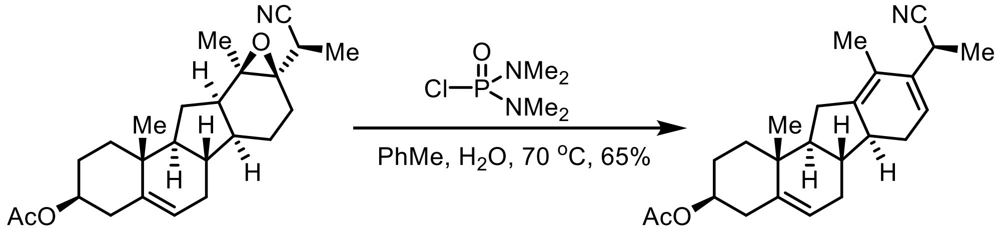

24 **25**

To a solution of **24** (4.50 g, 11.7 mmol) in PhMe/tetramethyldiamidophosphoric acid chloride (1:1, 90 mL) was added H_2O (4.5 mL) under argon atmosphere at 25 ℃. After being stirred at 70 ℃ for 10 h, the reaction mixture was diluted with water (200 mL) and extracted with ether (3 × 100 mL). The organic phases were combined, dried with anhydrous $MgSO_4$, filtered and concentrated. The residue was purified via flash column chromatography on silica gel (petroleum ether / dichloromethane = 3:1 to 1:4) to afford compound **25** (2.79 g, 65%) as a white solid.

TLC (petroleum ether / dichloromethane, 2:1 v/v): R_f = 0.43.

OR: $[\alpha]_D^{25}$ = +24.4 (c = 0.73, $CHCl_3$).

IR (neat): ν_{max}: 3020, 2925, 2236, 1731, 1655, 1444, 1371, 1244, 1088, 1033, 964, 766 cm^{-1}.

^{1}H NMR (400 MHz, $CDCl_3$): δ 5.69 (s, 1H), 5.49 – 5.42 (m, 1H), 4.67 – 4.58 (m, 1H), 3.83 (d, *J* = 7.2 Hz, 1H), 2.44 – 2.37 (m, 2H), 2.31 – 2.23 (m, 2H), 2.21 – 2.13 (m, 3H), 2.12 – 2.05 (m, 1H), 2.04 (s, 3H), 1.94 – 1.88 (m, 2H), 1.84 (s, 3H), 1.78 – 1.59 (m, 3H), 1.40 (d, *J* = 7.2 Hz, 3H), 1.34 – 1.26 (m, 2H), 1.00 (s, 3H).

^{13}C NMR (100 MHz, $CDCl_3$): δ 170.5, 146.0, 140.5, 130.3, 127.0, 123.4, 123.3, 121.5, 73.9, 59.7, 47.9, 45.2, 38.0, 37.7, 37.1, 29.7, 28.5, 27.5, 27.5, 26.8, 21.4, 19.1, 17.2, 13.7.

HRMS (ESI): *m/z* calcd. for $C_{24}H_{32}NO_2$ $[M+H]^+$ 366.2428, found 366.2426.

Conversion of ***25*** *into* ***22*** *(unoptimized) to confirm the C20 configuration*

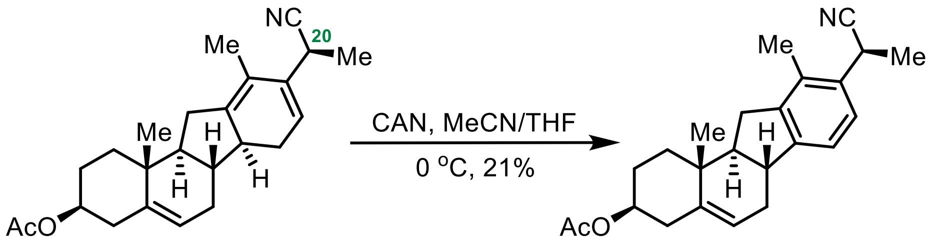

25 **22**

Following the procedure for the conversion of **21** into **22**, compound **22** (6.2 mg, 21%) was prepared from **25** (30 mg, 0.082 mmol) and purified through flash column chromatography (petroleum ether / ethyl acetate = 40:1 to 10:1 v/v).

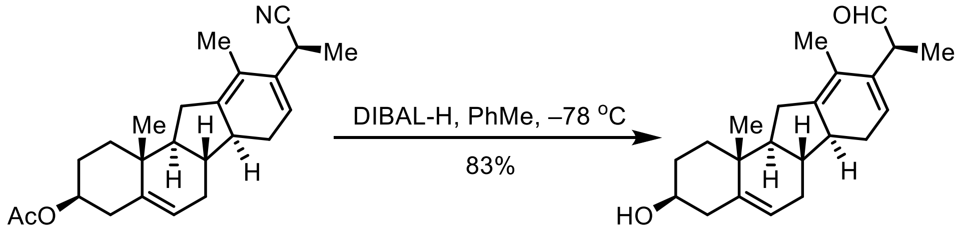

25 **26**

To a mixture of **25** (6.00 g, 16.4 mmol) in anhydrous PhMe (328 mL) were slowly added DIBAL-H (1.0 M in THF, 65.7 mL, 65.7 mmol) under argon atmosphere at –78 ℃. After being stirred at –78 ℃ for 4 h, the reaction was quenched by dropwise addition of HOAc/H_2O (1:4, 164 mL). After removal of the cooling bath, ethyl acetate (300 mL) and H_2O (600 mL) were added. The mixture was stirred at 25 ℃ until two clear layers formed. The organic phase was separated and the aqueous phase was extracted with ethyl acetate (3 × 300 mL). The combined organic phase was dried over anhydrous $MgSO_4$ and filtered. The solvents were removed under reduced pressure and the residue was purified by silica gel chromatography (petroleum ether / ethyl acetate = 8:1 to 2:1) to furnish aldehyde **26** (4.45 g, 83%) as a white foam.

TLC (petroleum ether / ethyl acetate, 2:1 v/v): R_f = 0.47.

OR: $[\alpha]_D^{25}$ = +132.7 (c = 0.26, $CHCl_3$).

IR (neat): ν_{max}: 3364, 2925, 2833, 2716, 1721, 1450, 1378, 1282, 1216, 1051, 818, 757 cm^{-1}.

^{1}H NMR (400 MHz, $CDCl_3$): δ 9.56 (s, 1H), 5.66 (s, 1H), 5.45 – 5.38 (m, 1H), 3.62 – 3.48 (m, 2H), 2.41 – 2.34 (m, 1H), 2.24 – 2.10 (m, 5H), 2.05 – 1.99 (m, 1H), 1.99 – 1.91 (m, 2H), 1.91 – 1.83 (m,

5H), 1.67 – 1.62 (m, 1H), 1.62 – 1.56 (m, 2H), 1.30 – 1.21 (m, 2H), 1.19 (d, *J* = 6.8 Hz, 3H), 0.99 (s, 3H).

^{13}C NMR (100 MHz, $CDCl_3$): δ 201.2, 146.5, 141.6, 131.5, 128.3, 122.5, 122.3, 71.9, 59.7, 50.4, 48.1, 45.3, 41.8, 38.3, 37.0, 31.3, 29.8, 27.8, 27.7, 19.2, 14.0, 11.2.

HRMS (ESI): *m/z* calcd. for $C_{22}H_{31}O_2$ $[M+H]^+$ 327.2319, found 327.2321.

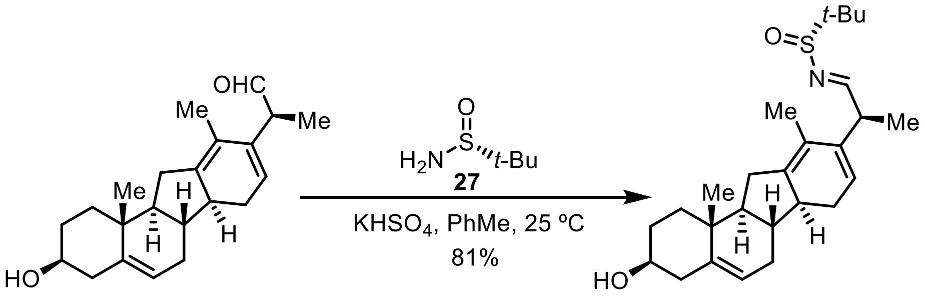

26 **13**

$KHSO_4$ (7.01 g, 51.5 mmol), **27** (2.34 g, 19.3 mmol), and **26** (4.20 g, 12.9 mmol) were placed in a flask with a rubber septum, which was evacuated and backfilled with argon three times. PhMe (250 mL) was added and the mixture was stirred at 25 ℃ for 10 h. The reaction was quenched by saturated NH_4Cl solution (300 mL) and extracted with EtOAc (3 × 200 mL). The combined organic phases were washed with brine (2 × 100 mL), dried over Na_2SO_4, filtered and concentrated in *vacuo*. The residue was purified via flash column chromatography on silica gel (petroleum ether / ethyl acetate = 8:1 to 1:1) to give *N*-sulfinyl imine **13** (4.48 g, 81%) as a light yellow solid.

TLC (petroleum ether / ethyl acetate, 2:1 v/v): R_f = 0.45.

OR: $[\alpha]_D^{25}$ = –74.2 (c = 0.94, $CHCl_3$).

IR (neat): ν_{max}: 3402, 2972, 2924, 1735, 1612, 1453, 1365, 1282, 1214, 1055, 815, 751 cm^{-1}.

^{1}H NMR (400 MHz, $CDCl_3$): δ 7.93 (d, *J* = 4.0 Hz, 1H), 5.61 (s, 1H), 5.46 – 5.38 (m, 1H), 3.89 – 3.80 (m, 1H), 3.59 – 3.49 (m, 1H), 2.41 – 2.32 (m, 1H), 2.22 – 2.11 (m, 5H), 2.09 – 1.94 (m, 3H), 1.93 – 1.87 (m, 2H), 1.86 (s, 3H), 1.70 – 1.65 (m, 1H), 1.65 – 1.57 (m, 2H), 1.27 (d, *J* = 6.8 Hz, 3H), 1.24 – 1.20 (m, 2H), 1.18 (s, 9H), 0.98 (s, 3H).

^{13}C NMR (100 MHz, $CDCl_3$): δ 169.8, 146.7, 141.7, 133.7, 126.9, 122.5, 121.8, 71.9, 59.7, 56.8, 48.1, 45.3, 42.7, 41.9, 38.3, 37.0, 31.4, 29.8, 27.8, 26.5, 22.4 (× 3), 19.1, 14.1, 13.7.

HRMS (ESI): *m/z* calcd. for $C_{26}H_{40}NO_2S$ $[M+H]^+$ 430.2774, found 430.2777.

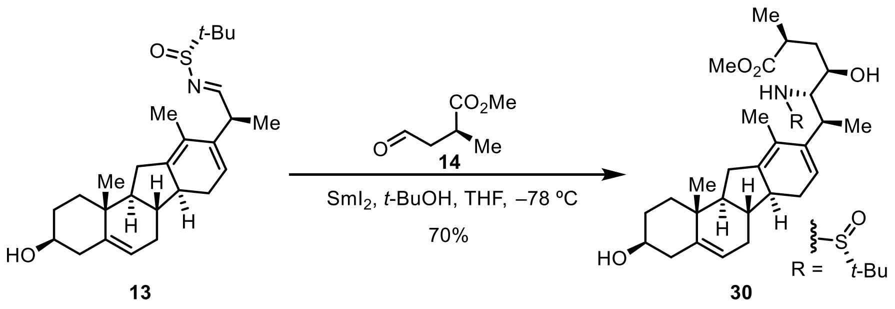

A mixture of **13** (4.20 g, 9.79 mmol), **14** (3.82 g, 29.4 mmol), and *t*-BuOH (3.75 mL, 39.2 mmol) in THF (200 mL) was added dropwise to a solution of SmI_2 (0.1 M in THF, 392 mL, 39.2 mmol) at –78 ℃ and stirred at the same temperature overnight. After the reaction was quenched with saturated aqueous $Na_2S_2O_3$ (500 mL) and H_2O (500 mL), the resultant mixture was extracted with EtOAc (3 × 300 mL), and the combined organic phases were washed with brine (2 × 100 mL), dried over Na_2SO_4, filtered and concentrated in *vacuo*. The crude product was purified by silica gel column chromatography (dichloromethane / methanol = 100:1 to 20:1) to give compounds **30** (3.84 g, 70%) and **30'** (121 mg, 2.2%) both as a white foam.

Compound 30

TLC (dichloromethane / methanol, 20:1 v/v): R_f = 0.43.

OR: $[\alpha]_D^{25}$ = –54.7 (c = 0.99, $CHCl_3$).

IR (neat): ν_{max}: 3380, 3255, 3015, 2837, 1994, 1726, 1663, 1455, 1287, 1215, 1026, 744 cm^{-1}.

^{1}H NMR (400 MHz, $CDCl_3$): δ 5.61 (s, 1H), 5.45 – 5.36 (m, 1H), 3.91 – 3.83 (m, 1H), 3.69 (s, 3H), 3.59 – 3.51 (m, 1H), 3.48 (s, 1H), 3.43 – 3.36 (m, 1H), 3.01 – 2.91 (m, 1H), 2.91 – 2.81 (m, 1H), 2.41 – 2.32 (m, 2H), 2.24 – 2.07 (m, 6H), 2.06 – 2.01 (m, 1H), 1.99 – 1.92 (m, 1H), 1.92 – 1.85 (m, 3H), 1.84 (s, 3H), 1.71 – 1.66 (m, 2H), 1.65 – 1.59 (m, 2H), 1.59 – 1.53 (m, 1H), 1.37 – 1.31 (m, 1H), 1.23 (d, *J* = 7.2 Hz, 3H), 1.12 (s, 9H), 1.09 (d, *J* = 6.4 Hz, 3H), 0.93 (s, 3H).

^{13}C NMR (100 MHz, $CDCl_3$): δ 177.3, 146.6, 141.5, 137.0, 127.3, 122.7, 122.1, 71.9, 71.1, 59.8, 59.5, 55.7, 51.7, 47.9, 45.5, 41.9, 38.3, 37.0, 36.3, 36.2, 35.1, 31.3, 29.7, 27.8, 24.8, 22.6 (×3), 18.8, 18.4, 16.1, 13.8.

HRMS (ESI): *m/z* calcd. for $C_{32}H_{52}NO_5S$ $[M+H]^+$ 562.3561, found 562.3563.

*Note: a byproduct (compound **30’**) was isolated in 2.2% yield in the above reductive coupling, which was confirmed to be the C20-epimer of **30**, through the conversion shown below starting*

from compounds ***S5*** *(the C20-epimer of* ***24****). Formation of* ***30'*** *could be due to partial epimerization of the C20 stereocenter at the aldehyde (in* ***26****) or imine (in* ***13****) stage.*

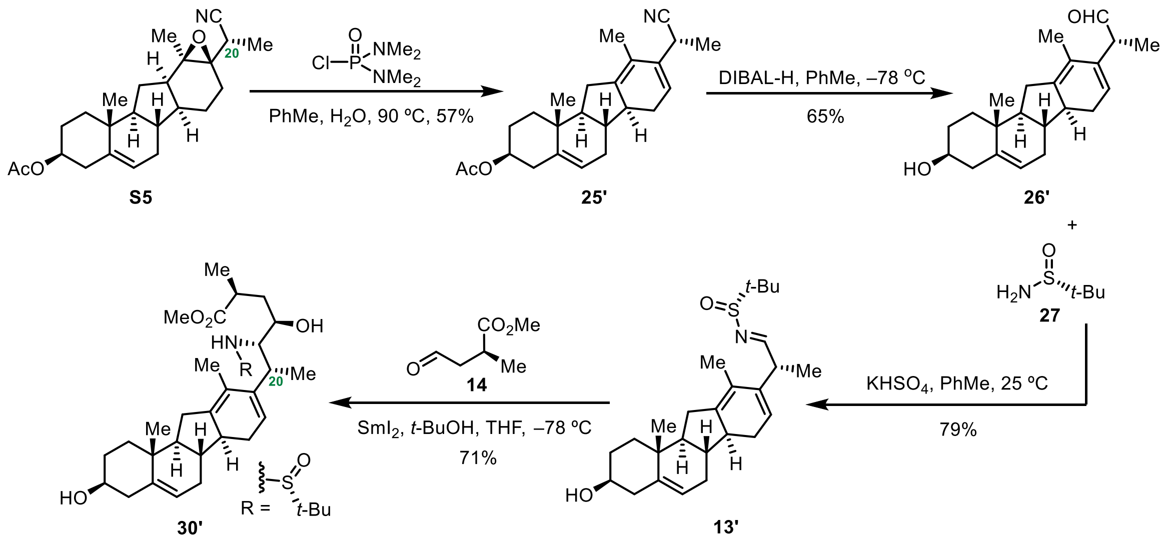

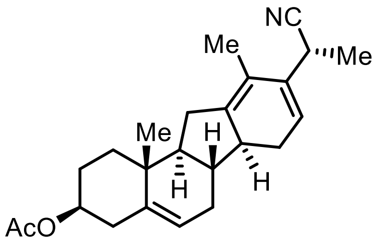

25'

Following the procedure for the conversion of **24** into **25**, compound **25'** (136 mg, 57%) was prepared starting from **S5** (250 mg, 0.652 mmol) and purified through flash column chromatography (petroleum ether / ethyl acetate = 60:1 to 8:1 v/v).

TLC (petroleum ether / ethyl acetate, 5:1 v/v): R_f = 0.52.

OR: $[\alpha]_D^{25}$ = –17.9 (c = 0.42, $CHCl_3$).

IR (neat) ν_{max}: 2925, 2237, 2032, 1731, 1442, 1372, 1244, 1084, 1030, 805, 752 cm^{-1}.

^{1}H NMR (400 MHz, $CDCl_3$): δ 5.66 (s, 1H), 5.49 – 5.43 (m, 1H), 4.68 – 4.57 (m, 1H), 3.81 (q, *J* = 7.2 Hz, 1H), 2.44 – 2.37 (m, 2H), 2.35 – 2.24 (m, 2H), 2.22 – 2.14 (m, 3H), 2.13 – 2.07 (m, 1H), 2.04 (s, 3H), 1.94 – 1.87 (m, 2H), 1.79 (s, 3H), 1.71 – 1.64 (m, 1H), 1.63 – 1.57 (m, 2H), 1.40 (d, *J* = 7.2 Hz, 3H), 1.32 – 1.27 (m, 1H), 1.26 – 1.21 (m, 1H), 0.99 (s, 3H).

^{13}C NMR (100 MHz, $CDCl_3$): δ 169.5, 144.9, 139.4, 129.4, 125.6, 122.5, 121.9, 120.2, 72.9, 58.6, 46.7, 44.3, 37.0, 36.7, 36.1, 28.7, 27.2, 26.9, 26.5, 26.0, 20.4, 18.1, 16.6, 12.3.

HRMS (ESI): *m/z* calcd. for $C_{24}H_{32}NO_2$ $[M+H]^+$ 366.2428, found 366.2423.

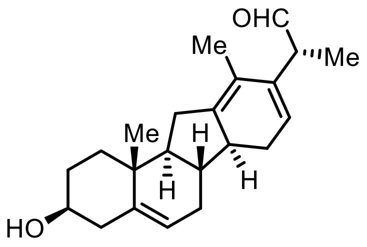

26'

Following the procedure for the conversion of **25** into **26**, compound **26'** (58 mg, 65%) was prepared starting from **25'** (100 mg, 0.306 mmol) and purified through flash column chromatography (petroleum ether / ethyl acetate = 8:1 to 2:1 v/v).

TLC (petroleum ether / ethyl acetate, 3:1 v/v): R_f = 0.40.

OR: $[\alpha]_D^{25}$ = –154 (c = 0.59, $CHCl_3$).

IR (neat) ν_{max}: 3389, 2924, 2713, 1721, 1451, 1378, 1261, 1210, 1081, 1051, 864, 810, 754 cm^{-1}.

^{1}H NMR (400 MHz, $CDCl_3$): δ 9.61 (s, 1H), 5.69 – 5.62 (m, 1H), 5.45 – 5.39 (m, 1H), 3.63 – 3.49 (m, 2H), 2.42 – 2.33 (m, 1H), 2.26 – 2.09 (m, 5H), 2.09 – 2.00 (m, 2H), 2.00 – 1.94 (m, 1H), 1.94 – 1.88 (m, 2H), 1.87 (s, 4H), 1.67 – 1.57 (m, 3H), 1.29 – 1.25 (m, 1H), 1.24 – 1.20 (m, 1H), 1.19 (d, J = 6.4 Hz, 3H), 0.99 (s, 3H).

^{13}C NMR (100 MHz, $CDCl_3$): δ 202.2, 146.3, 141.6, 132.4, 127.9, 122.5, 122.2, 71.9, 59.7, 50.3, 48.1, 45.3, 41.8, 38.3, 37.0, 31.3, 29.7, 28.1, 27.1, 19.2, 13.9, 11.6.

HRMS (ESI): *m/z* calcd. for $C_{22}H_{31}O_2$ $[M+H]^+$ 327.2319, found 327.2314.

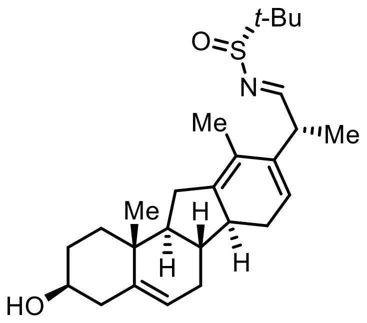

13'

Following the procedure for the conversion of **26** into **13**, compound **13'** (30 mg, 79%) was prepared starting from **26'** (29 mg, 0.089 mmol) and purified through flash column chromatography (petroleum ether / ethyl acetate = 8:1 to 1:1 v/v).

TLC (petroleum ether / ethyl acetate, 2:1 v/v): R_f = 0.45.

OR: $[\alpha]_D^{25}$ = –168 (c = 1.02, $CHCl_3$).

IR (neat) ν_{max}: 3416, 2965, 2925, 1612, 1453, 1366, 1260, 1178, 1056, 872, 807, 748cm^{-1}.

^{1}H NMR (400 MHz, $CDCl_3$): δ 7.99 (d, J = 3.6 Hz, 1H), 5.66 – 5.58 (m, 1H), 5.45 – 5.37 (m, 1H), 3.89 – 3.80 (m, 1H), 3.59 – 3.48 (m, 1H), 2.41 – 2.32 (m, 1H), 2.21 – 2.09 (m, 5H), 2.08 – 1.92 (m, 3H), 1.89 – 1.84 (m, 4H), 1.78 (s, 1H), 1.64 – 1.52 (m, 2H), 1.26 (d, J = 7.2 Hz, 3H), 1.25 – 1.22 (m, 1H), 1.20 (s, 9H), 1.19 – 1.16 (m, 1H), 0.98 (s, 3H).

^{13}C NMR (100 MHz, $CDCl_3$): δ 171.1, 146.5, 141.7, 134.8, 126.4, 122.5, 121.8, 71.8, 59.6, 56.7, 48.0, 45.3, 42.8, 41.8, 38.3, 37.0, 31.3, 29.7, 28.2, 26.7, 22.5 (×3), 19.2, 14.6, 13.7.

HRMS (ESI): *m/z* calcd. for $C_{26}H_{40}NO_2S$ $[M+H]^+$ 430.2774, found 430.2772.

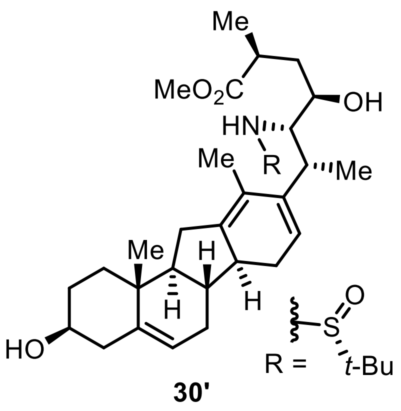

Following the procedure for the conversion of **13** into **30**, compound **30'** (14 mg, 71%) was prepared starting from **13'** (15 mg, 0.035 mmol) and purified through flash column chromatography (dichloromethane / acetone = 8:1 to 1:1 v/v).

TLC (dichloromethane / acetone, 5:1 v/v): R_f = 0.45.

OR: $[\alpha]_D^{25}$ = +17.2 (c = 0.36, $CHCl_3$).

IR (neat) ν_{max}: 3304, 2925, 1720, 1456, 1369, 1259, 1199, 1084, 1017, 869, 799 cm^{-1}.

^{1}**H NMR** (400 MHz, $CDCl_3$): δ 5.58 (s, 1H), 5.45 – 5.39 (m, 1H), 3.70 – 3.64 (m, 1H), 3.60 – 3.42 (m, 6H), 3.17 (d, *J* = 8.0 Hz, 1H), 2.79 – 2.63 (m, 3H), 2.42 – 2.33 (m, 1H), 2.33 – 2.24 (m, 1H), 2.24 – 2.11 (m, 3H), 2.11 – 1.94 (m, 4H), 1.94 – 1.82 (m, 3H), 1.79 (s, 3H), 1.62 – 1.53 (m, 4H), 1.28 (s, 9H), 1.23 – 1.18 (m, 2H), 1.18 – 1.13 (m, 6H), 0.98 (s, 3H).

^{13}**C NMR** (100 MHz, $CDCl_3$): δ 177.0, 146.7, 141.6, 136.9, 125.0, 122.5, 121.2, 71.9, 70.1, 65.0, 59.6, 57.1, 51.5, 48.4, 45.2, 41.9, 38.3, 37.8, 37.0, 35.9, 35.1, 31.3, 29.8, 28.1, 25.0, 23.3 (×3), 19.2, 17.6, 16.8, 13.5.

HRMS (ESI): *m/z* calcd. for $C_{32}H_{52}NO_5S$ $[M+H]^+$ 562.3561, found 562.3556.

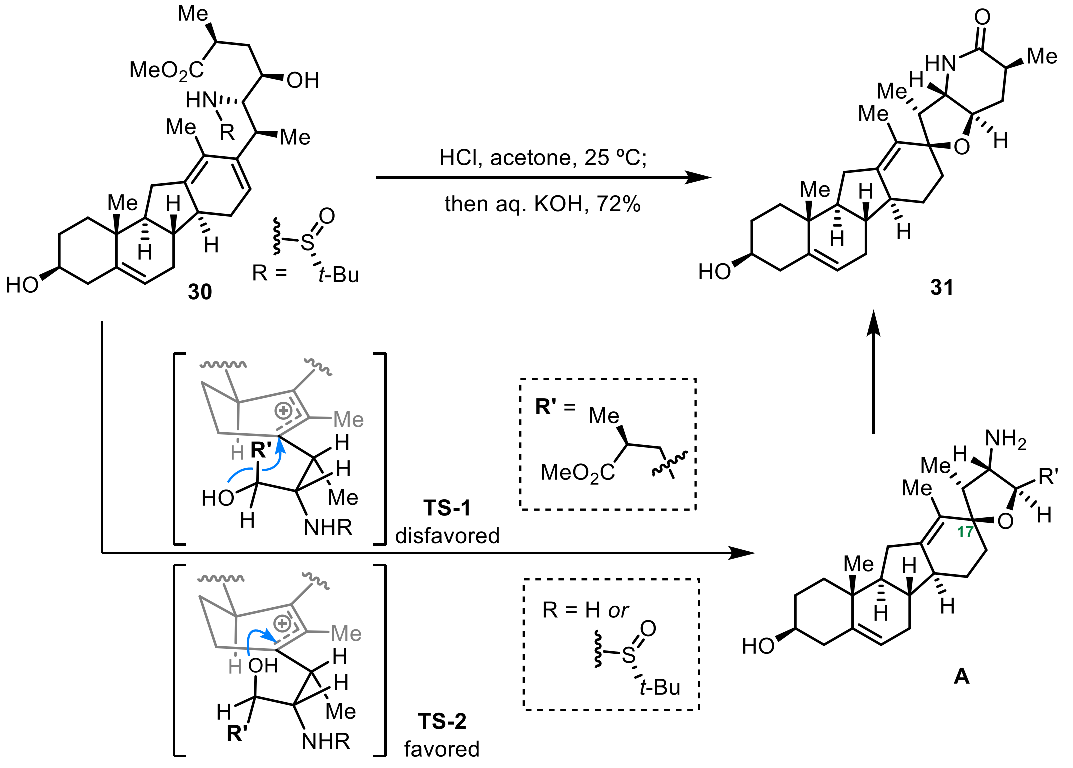

HCl (4.0 M in MeOH, 13.4 mL, 53.5 mmol) was added to a stirred solution of compound **30** (3.00 g, 5.34 mmol) in acetone (110 mL) at 0 ℃. The mixture was stirred at 25 ℃ for 2 h. After completion of the reaction (monitored by LC-MS), the mixture was cooled to 0 ℃, to which was added aqueous KOH (1.3 M in H_2O, 62 mL, 80.1 mmol). The resulting mixture was stirred at 25 ℃ for 4 h, before being quenched by saturated aqueous NH_4Cl solution at 0 ℃. The mixture was extracted with CH_2Cl_2 (3 × 100 mL), and the combined organic phases were washed with brine (50 mL), dried over Na_2SO_4, filtered and concentrated in *vacuo*. Purification of the crude product by silica gel column chromatography [dichloromethane (containing 0.5% ammonia) / methanol = 80:1 to 10:1] gave lactam **31** (1.64 g, 72%) as a white solid.

TLC (dichloromethane / methanol, 20:1 v/v): R_f = 0.40.

OR: $[\alpha]_D^{25}$ = –36.4 (c = 0.45, $CHCl_3$).

IR (neat): ν_{max}: 3390, 3289, 3075, 2928, 1748, 1655, 1459, 1389, 1273, 1044, 810, 751 cm^{-1}.

^{1}H NMR (400 MHz, $CDCl_3$): δ 5.88 (s, 1H), 5.41 – 5.32 (m, 1H), 3.68 – 3.59 (m, 1H), 3.59 – 3.47 (m, ^{2H}), 2.56 – 2.42 (m, 2H), 2.42 – 2.33 (m, 2H), 2.30 – 2.14 (m, 4H), 1.95 (dd, *J* = 14.8, 3.2 Hz, 1H), 1.87 – 1.80 (m, 2H), 1.77 – 1.66 (m, 3H), 1.66 – 1.63 (m, 3H), 1.62 (s, 1H), 1.58 – 1.49 (m, 3H), 1.46 – 1.37 (m, 1H), 1.33 (d, *J* = 7.2 Hz, 3H), 1.31 – 1.30 (m, 1H), 1.27 (d, *J* = 5.2 Hz, 1H), 1.19 (dd, *J* = 13.2, 3.6 Hz, 1H), 1.00 – 0.87 (m, 6H).

^{13}C NMR (100 MHz, $CDCl_3$): δ 175.8, 144.0, 141.6, 125.5, 121.8, 87.6, 73.1, 71.8, 60.9, 52.0, 49.2, 41.8, 41.6, 39.8, 38.1, 36.5, 35.1, 34.4, 31.8, 31.3, 31.0, 29.0, 24.5, 18.7, 18.3, 13.2, 10.5.

HRMS (ESI): *m/z* calcd. for $C_{27}H_{40}NO_3$ $[M+H]^+$ 426.3003, found 426.3006.

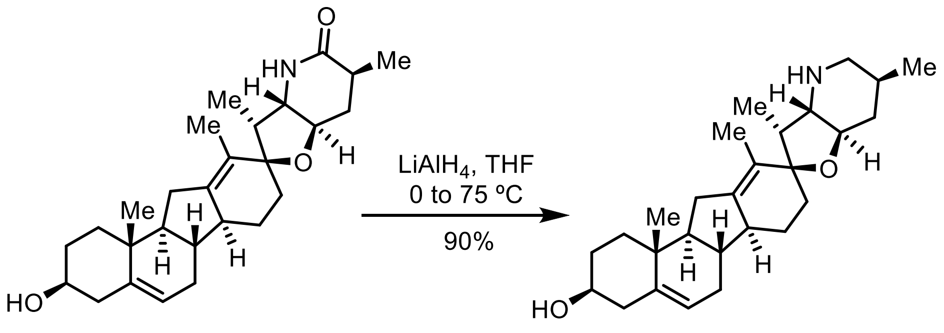

31 (–)-cyclopamine (**4**)

Lithium aluminum hydride (2.5 M in THF, 7.5 mL, 18.8 mmol) was added to a solution of **31** (1.60 g, 3.76 mmol) in anhydrous THF (75 mL) at 0 ℃. The resulting mixture was heated at 75 ℃ for 4 h. After the reaction was cooled to ambient temperature, it was quenched with saturated potassium

sodium tartrate and potassium carbonate solution. The resultant mixture was extracted with CH_2Cl_2 (5 × 100 mL), and the combined organic extracts were washed with brine (50 mL), dried over Na_2SO_4, filtered and concentrated in *vacuo*. The crude product was purified by silica gel column chromatography [dichloromethane (containing 0.5% ammonia) / methanol = 80:1 to 10:1] to afford (–)-cyclopamine (**4**, 1.40 g, 90%) as a white solid.

TLC (dichloromethane / methanol, 10:1 v/v): R_f = 0.45.

OR: $[\alpha]_D^{25}$ = –66.3 (c = 1.20, $CHCl_3$).

IR (neat): ν_{max}: 3344, 2926, 1457, 1377, 1259, 1208, 1117, 1040, 984, 923, 808, 749 cm^{-1}.

^{1}H NMR (400 MHz, CD_2Cl_2): δ 5.37 (dt, *J* = 4.8, 2.0 Hz, 1H), 3.47 (tt, *J* = 11.2, 4.4 Hz, 1H), 3.15 (td, *J* = 10.4, 4.0 Hz, 1H), 3.00 (dd, *J* = 12.8, 4.4 Hz, 1H), 2.59 (t, *J* = 9.2 Hz, 1H), 2.43 – 2.36 (m, 1H), 2.36 – 2.30 (m, 1H), 2.28 – 2.20 (m, 3H), 2.20 – 2.04 (m, 3H), 1.84 (ddd, *J* = 13.6, 4.0, 2.4 Hz, 1H), 1.81 – 1.75 (m, 2H), 1.75 – 1.65 (m, 3H), 1.61 (s, 3H), 1.57 – 1.55 (m, 1H), 1.54 – 1.52 (m, 1H), 1.49 – 1.46 (m, 1H), 1.44 – 1.36 (m, 1H), 1.32 – 1.25 (m, 1H), 1.25 – 1.16 (m, 2H), 1.09 (q, *J* = 11.2 Hz, 1H), 0.96 (s, 3H), 0.91 (d, *J* = 6.8 Hz, 3H), 0.88 (d, *J* = 7.6 Hz, 3H).

^{13}C NMR (100 MHz, CD_2Cl_2): δ 142.6, 142.3, 127.4, 122.0, 85.4, 75.9, 72.1, 66.9, 55.2, 52.5, 49.5, 42.3 (×2), 40.3, 39.5, 38.6, 36.9, 32.3, 32.0, 31.9, 31.5, 29.2, 25.1, 19.1, 18.8, 13.2, 10.8.

HRMS (ESI): *m/z* calcd. for $C_{27}H_{42}NO_2$ $[M+H]^+$ 412.3210, found 412.3211.

Supplementary Table 3. Comparison of ^{1}H NMR spectroscopic data of synthetic (–)-cyclopamine (4).

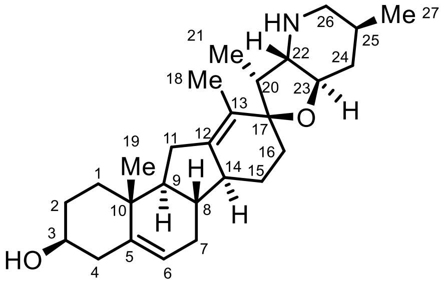

(–)-cyclopamine (**4**)

No.	**Gao's synthetic**[5] δ ^{1}H [ppm; mult; *J* (Hz)] 500 MHz, CD_2Cl_2	**Baran's synthetic**[7] δ ^{1}H [ppm; mult; *J* (Hz)] 600 MHz, CD_2Cl_2	**Our synthetic** δ ^{1}H [ppm; mult; *J* (Hz)] 400 MHz, CD_2Cl_2
1	1.23 – 1.18 (1H, overlap)<br>1.76 – 1.74 (1H, overlap)	1.26 – 1.15 (1H, m)<br>1.75 – 1.65 (1H, m)	1.25 – 1.16 (1H, m)<br>1.75 – 1.65 (1H, m),
2	1.56 – 1.52 (1H, overlap)<br>1.84 – 1.82 (1H, overlap)	1.60 – 1.44 (1H, m)<br>1.82 – 1.75 (1H, m)	1.57 – 1.55 (1H, m)<br>1.81 – 1.75 (1H, m),
3	3.50 (1H, tt, 11.1, 4.6)	3.47 (1H, tt, 11.1, 4.6)	3.47 (1H, tt, 11.2, 4.4)
4	2.23–2.18 (1H, overlap)<br>2.37 (1H, ddd, 13.0, 4.8, 2.3)	2.20 – 2.07 (1H, m)<br>2.34 (1H, ddd, 13.1, 4.8, 2.3)	2.20 – 2.04 (1H, m)<br>2.36 – 2.30 (1H, m)
6	5.40 (1H, dt, 4.7, 2.0)	5.37 (1H, dt, 4.6, 2.0)	5.37 (1H, dt, 4.8, 2.0)
7	1.79 – 1.76 (1H, overlap)<br>2.32 – 2.23 (1H, overlap)	1.75 – 1.65 (1H, m)<br>2.31 – 2.20 (1H, m)	1.75 – 1.65 (1H, m)<br>2.28 – 2.20 (1H, m)
8	1.33 – 1.29 (1H, overlap)	1.31 – 1.27 (1H, m)	1.32 – 1.25 (1H, m)
9	1.43 (1H, td, 11.3, 8.7)	1.41 (1H, td, 11.4, 8.7)	1.44 – 1.36 (1H, m)
11	2.18 – 2.13 (1H, overlap)<br>2.32 – 2.23 (1H, overlap)	2.20 – 2.07 (1H, m)<br>2.31 – 2.20 (1H, m)	2.20 – 2.04 (1H, m)<br>2.28 – 2.20 (1H, m)
14	1.73 – 1.71 (1H, m)	1.75 – 1.65 (1H, m)	1.75 – 1.65 (1H, m)
15	1.27 – 1.23 (1 H, m)<br>1.82 – 1.79 (1H, overlap)	1.26 – 1.15 (1H, m)<br>1.82 – 1.75 (1H, m)	1.25 – 1.16 (1H, m)<br>1.81 – 1.75 (1H, m)
16	1.51 – 1.46 (1H, overlap)<br>1.87 (1 H, ddd, 13.5, 4.0, 2.3)	1.60 – 1.44 (1H, m)<br>1.85 (1H, ddd, 13.7, 4.1, 2.4)	1.49 – 1.46 (1H, m),<br>1.84 (1H, ddd, 13.6, 4.0, 2.4)
18	1.64 (3H, s)	1.61 (3H, s)	1.61 (3H, s)
19	0.99 (3H, s)	0.96 (3H, s)	0.96 (3H, s)
20	2.42. (1H, p, 7.7)	2.41 (1H, p, 7.7)	2.43 – 2.36 (1H, m)
21	0.93 (3H, d, 7.6)	0.91 (3H, d, 7.6)	0.88 (3H, d, 7.6)
22	2.63 (1H, t, 9.2)	2.62 (1H, t, 9.2)	2.59 (1H, t, 9.2)
23	3.20 (1H, td, 10.3, 3.8)	3.19 (1H, td, 10.3, 3.8)	3.15 (1H, td,10.4,4.0)
24	1.14 (1H, q, 11.4)<br>2.13 – 2.09 (1H, overlap)	1.11 (1H, q, 11.4)<br>2.20 – 2.07 (1H, m)	1.09 (1H, q, 11.2)<br>2.20 – 2.04 (1H, m)
25	1.61 – 1.58 (1H, overlap)	1.60 – 1.44 (1H, m)	1.54 – 1.52 (1H, m)
26	2.32 – 2.23 (1H, overlap)<br>3.05 (1H, dd, 12.7, 4.2)	2.31 – 2.20 (1H, m)<br>3.04 (1H, dd, 12.6, 3.8)	2.28 – 2.20 (1H, m)<br>3.00 (1H, dd, 12.8, 4.4)
27	0.94 (3H, d, 6.6)	0.92 (3H, d, 6.7)	0.91 (3H d, 6.8)

Supplementary Table 4. Comparison of ^{13}C NMR spectroscopic data of synthetic (–)-cyclopamine (4).

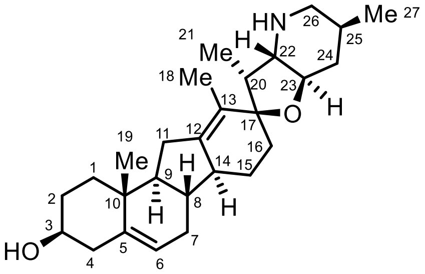

(–)-cyclopamine (**4**)

position	Gao's synthetic[5] δ ^{13}C (ppm) 125 MHz, CD_2Cl_2	Baran's synthetic[7] δ ^{13}C (ppm) 125 MHz, CD_2Cl_2	Our synthetic δ ^{13}C (ppm) 100 MHz, CD_2Cl_2	Deviation Δδ (ppm)	
				Gao's synthetic	Baran's synthetic
1	38.2	38.6	38.6	–0.4	0
2	31.5	31.8	32.0	–0.5	–0.2
3	71.7	72.1	72.1	–0.4	0
4	41.9	42.3	42.3	–0.4	0
5	141.9	142.2	142.3	–0.4	–0.1
6	121.7	122.2	122.0	–0.3	0.2
7	31.1	31.5	31.5	–0.4	0
8	41.9	42.3	42.3	–0.4	0
9	52.1	52.5	52.5	–0.4	0
10	36.5	36.9	36.9	–0.4	0
11	28.8	29.2	29.2	–0.4	0
12	142.3	142.7	142.6	–0.3	0.1
13	126.9	127.3	127.4	–0.5	–0.1
14	49.1	49.5	49.5	–0.4	0
15	24.7	25.0	25.1	–0.4	–0.1
16	31.8	31.9	32.3	–0.5	–0.4
17	85.1	85.5	85.4	–0.3	0.1
18	12.8	13.2	13.2	–0.4	0
19	18.4	18.8	18.8	–0.4	0
20	39.8	40.1	40.3	–0.5	–0.2
21	10.5	10.9	10.8	–0.3	0.1
22	66.3	66.6	66.9	–0.6	–0.3
23	75.4	75.7	75.9	–0.5	–0.2
24	39.1	39.4	39.5	–0.4	–0.1
25	31.5	31.9	31.9	–0.4	0
26	54.7	54.9	55.2	–0.5	–0.3
27	18.7	19.0	19.1	–0.4	–0.1

2.2 Preparation of substrates for the biomimetic rearrangement reaction

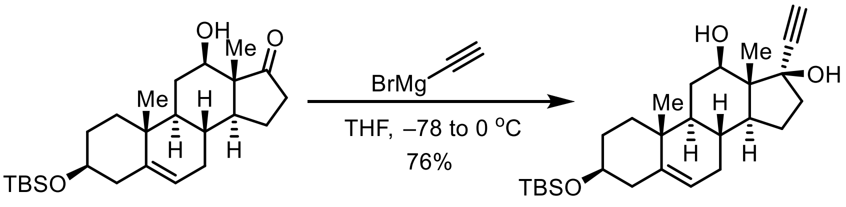

S6 **32b**

Following the procedure for the conversion of **17** into **18**, substrate **32b** (403 mg, 76%) was prepared starting from **S6**[1] (500 mg, 1.19 mmol) and purified through flash column chromatography (petroleum ether / ethyl acetate = 20:1 to 1:1 v/v).

TLC (petroleum ether / ethyl acetate, 2:1 v/v): R_f = 0.41.

OR: $[\alpha]_D^{25}$ = –79.0 (c = 1.67, $CHCl_3$).

IR (neat) ν_{max}: 3499, 3414, 3275, 2928, 1471, 1254, 1138, 1022, 835,612 cm^{-1}.

^{1}H NMR (400 MHz, $CDCl_3$) δ 5.33 – 5.28 (m, 1H), 4.15 (dd, *J* = 12.0, 4.0 Hz, 1H), 3.51 – 3.41 (m, 1H), 2.61 (s, 1H), 2.60 (s, 1H), 2.34 – 2.14 (m, 3H), 2.06 – 1.95 (m, 2H), 1.90 (s, 1H), 1.83 – 1.77 (m, 1H), 1.76 – 1.69 (m, 3H), 1.59 – 1.39 (m, 6H), 1.13 – 1.04 (m, 2H), 1.03 (s, 3H), 0.91 (s, 3H), 0.88 (s, 9H), 0.05 (s, 6H).

^{13}C NMR (100 MHz, $CDCl_3$) δ 141.5, 120.8, 87.1, 80.0, 74.9, 74.3, 72.6, 50.7, 49.6, 49.3, 42.8, 38.6, 37.5, 36.8, 32.1, 31.7, 31.2, 30.1, 26.0 (×3), 23.1, 19.5, 18.3, 7.4, –4.4 (×2).

HRMS (ESI): *m/z* calcd. for $C_{27}H_{45}O_3Si$ $[M+H]^+$ 445.3132, found 445.3134.

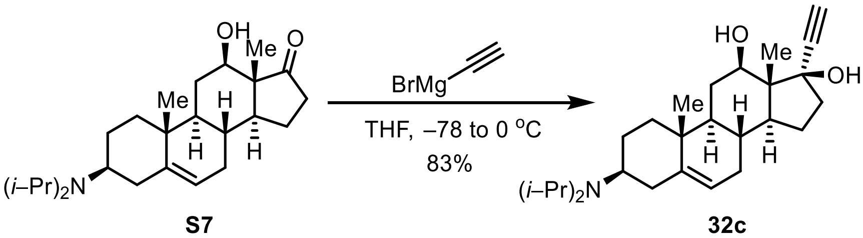

S7 **32c**

Following the procedure for the conversion of **17** into **18**, substrate **32c** (266 mg, 83%) was prepared starting from **S7**[1] (300 mg, 0.774 mmol) and purified through flash column chromatography (petroleum ether / ethyl acetate = 50:1 to 5:1 v/v).

TLC (dichloromethane / methanol, 10:1 v/v): R_f = 0.36;

OR: $[\alpha]_D^{25}$ = –57.4 (c = 0.77, $CHCl_3$).

IR (neat) ν_{max}: 3305, 3264, 2957, 2929, 1435, 1218, 1063, 1043,1025,771 cm^{-1};

^{1}H NMR (400 MHz, $CDCl_3$): δ 5.28 – 5.23 (m, 1H), 4.16 (dd, *J* = 11.2, 4.4 Hz, 1H), 3.20 – 3.08 (m, 2H), 2.66 (s, 1H), 2.62 (s, 1H), 2.55 (tt, *J* = 12.0, 4.0 Hz, 1H), 2.39 – 2.23 (m, 2H), 2.07 – 1.86 (m, 4H), 1.86 – 1.70 (m, 3H), 1.69 – 1.52 (m, 2H), 1.51 – 1.39 (m, 5H), 1.17 – 1.05 (m, 2H), 1.02 – 0.95 (m, 15H), 0.91 (s, 3H).

^{13}C NMR (100 MHz, $CDCl_3$): δ 143.7, 119.2, 87.1, 80.0, 75.0, 74.3, 54.7, 50.7, 49.7, 49.6, 44.7 (× 2), 40.1, 39.1, 38.7, 36.9, 31.8, 31.2, 30.2, 29.4, 23.3 (× 2), 23.2 (× 2), 23.2, 19.6, 7.4.

HRMS (ESI): *m/z* calcd. for $C_{27}H_{44}NO_2$ $[M+H]^+$ 414.3367, found 414.3365.

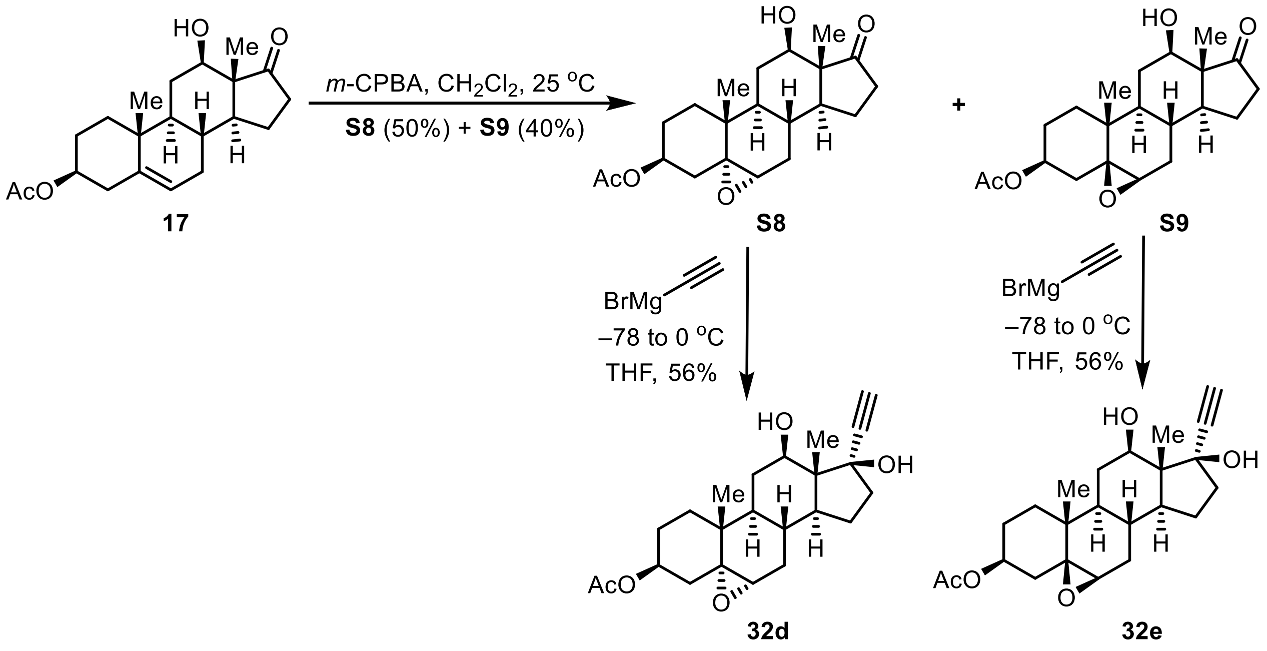

To a stirred solution of **17** (500 mg, 1.40 mmol) in CH_2Cl_2 (140 mL) was added *m*-CPBA (747 mg, 4.30 mmol) at 25 ℃. The reaction mixture was stirred for 2 h. The mixture was quenched with saturated aqueous Na_2SO_3 (70 mL) and extracted with CH_2Cl_2 (3 × 200 mL). The combined organic layers were washed with brine, dried over Na_2SO_4 and concentrated in *vacuo*. The residue was purified by flash column chromatography on silica gel (dichloromethane / acetone = 40:1 to 8:1 v/v) to furnish compounds **S8** (260 mg, 50%) and **S9** (209 mg, 40%) as white foam. *Note: the stereochemistries of both **S8** and **S9** were verified through X-ray crystallographic analysis of **32d** (vide infra).*

Following the procedure for the conversion of **17** into **18**, substrate **32d** (121 mg, 56%) was prepared starting from **S8** (200 mg, 0.551 mmol) and purified through flash column chromatography (petroleum ether / ethyl acetate = 8:1 to 2:1 v/v).

Following the procedure for the conversion of **17** into **18**, substrate **32e** (120 mg, 56%) was prepared starting from **S9** (200 mg, 0.551 mmol) and purified through flash column chromatography (petroleum ether / ethyl acetate = 8:1 to 2:1 v/v).

Compound S8

TLC (dichloromethane / acetone, 8:1 v/v): R_f = 0.56.

OR: $[\alpha]_D^{25}$ = –25.9 (c = 1.00, $CHCl_3$).

IR (neat) ν_{max}: 3529, 2953, 1726, 1242, 1155, 1066, 1028, 770 cm^{-1}.

^{1}H NMR (400 MHz, $CDCl_3$): δ 4.98 – 4.86 (m, 1H), 3.73 (dd, *J* = 10.8, 4.4 Hz, 1H), 3.00 (s, 1H), 2.92 (d, *J* = 4.4 Hz, 1H), 2.43 (dd, *J* = 19.2, 8.8 Hz, 1H), 2.19 – 2.07 (m, 2H), 2.04 (dd, *J* = 8.8, 4.0 Hz, 1H), 1.99 (s, 3H), 1.97 – 1.90 (m, 1H), 1.68 – 1.63 (m, 2H), 1.62 – 1.56 (m, 2H), 1.55 (d, *J* = 3.6 Hz, 1H), 1.53 – 1.48 (m, 1H), 1.48 – 1.38 (m, 2H), 1.34 (ddd, *J* = 12.8, 5.2, 2.0 Hz, 1H), 1.29 – 1.14 (m, 3H), 1.09 (s, 3H), 0.87 (s, 3H).

^{13}C NMR (150 MHz, $CDCl_3$): δ 222.2, 170.2, 72.3, 71.1, 65.2, 58.5, 51.5, 49.6, 41.5, 36.0, 35.7, 35.3, 32.2, 28.6, 27.8, 27.4, 27.2, 21.6, 21.4, 15.8, 8.1.

HRMS (ESI): m/z calcd. for $C_{21}H_{31}O_5$ $[M+H]^+$ 363.2166, found 363.2168.

Compound S9

TLC (dichloromethane / acetone, 8:1 v/v): R_f = 0.46.

OR: $[\alpha]_D^{25}$ = +12.0 (c = 1.00, $CHCl_3$).

IR (neat) ν_{max}: 3552, 2959, 1726, 1247, 1215, 1050, 1032, 748 cm^{-1}.

^{1}H NMR (400 MHz, $CDCl_3$): δ 4.79 – 4.69 (m, 1H), 3.69 (dd, *J* = 11.2, 4.4 Hz, 1H), 3.13 (d, *J* = 2.8 Hz, 1H), 3.02 (s, 1H), 2.51 – 2.40 (m, 1H), 2.24 – 2.16 (m, 1H), 2.14 – 2.05 (m, 2H), 2.01 (s, 3H), 1.98 – 1.93 (m, 1H), 1.87 – 1.78 (m, 1H), 1.69 – 1.60 (m, 3H), 1.52 – 1.42 (m, 2H), 1.41 – 1.35 (m, 1H), 1.35 – 1.29 (m, 1H), 1.29 – 1.25 (m, 1H), 1.23 (s, 1H), 1.16 – 1.07 (m, 1H), 1.02 (s, 3H), 0.90 (s, 3H), 0.82 – 0.73 (m, 1H).

^{13}C NMR (150 MHz, $CDCl_3$): δ 222.5, 170.6, 72.5, 71.1, 63.2, 62.5, 51.4, 49.9, 49.0, 37.9, 36.7, 35.7, 35.3, 31.1, 28.9, 28.5, 27.2, 21.7, 21.4, 17.1, 8.0.

HRMS (ESI): *m/z* calcd. for $C_{21}H_{31}O_5$ $[M+H]^+$ 363.2166, found 363.2167.

Compound 32d

Melting point: 248–250 ℃

TLC (petroleum ether / acetone, 2:1 v/v): R_f = 0.40.

OR: $[\alpha]_D^{25}$ = –6.8 (c = 1.5, $CHCl_3$).

IR (neat) ν_{max}: 3441, 2949, 1728, 1219, 1157, 1128, 1034, 771 cm^{-1}.

^{1}H NMR (400 MHz, $CDCl_3$): δ 4.99 – 4.87 (m, 1H), 4.11 (dd, *J* = 11.2, 4.8 Hz, 1H), 2.89 (d, *J* = 4.4 Hz, 1H), 2.61 (s, 1H), 2.55 (s, 1H), 2.32 – 2.21 (m, 1H), 2.15 (t, *J* = 12.4 Hz, 1H), 2.01 (s, 3H), 2.00 – 1.87 (m, 3H), 1.74 – 1.65 (m, 3H), 1.65 – 1.57 (m, 2H), 1.53 – 1.45 (m, 2H), 1.45 – 1.34 (m, 4H), 1.35 – 1.30 (m, 1H), 1.29 – 1.22 (m, 1H), 1.10 (s, 3H), 0.84 (s, 3H).

^{13}C NMR (100 MHz, $CDCl_3$): δ 170.4, 86.7, 79.7, 74.5, 74.4, 71.2, 65.3, 58.9, 50.6, 49.6, 41.5, 38.4, 36.0, 35.2, 32.2, 29.7, 29.6, 27.9, 27.2, 22.9, 21.4, 15.9, 7.3.

HRMS (ESI): *m/z* calcd. for $C_{23}H_{33}O_5$ $[M+H]^+$ 389.2323, found 389.2325.

Compound 32e

TLC (petroleum ether / acetone, 2:1 v/v): R_f = 0.40.

OR: $[\alpha]_D^{25}$ = –26.6 (c = 1.7, $CHCl_3$).

IR (neat) ν_{max}: 3449, 3303, 2951, 1726, 1217, 1244, 1140, 1078 cm^{-1}.

^{1}H NMR (400 MHz, $CDCl_3$): δ 4.79 – 4.69 (m, 1H), 4.05 (dd, *J* = 11.2, 4.4 Hz, 1H), 3.10 (d, *J* = 2.8 Hz, 1H), 2.62 (s, 1H), 2.53 (s, 1H), 2.32 – 2.22 (m, 1H), 2.15 – 2.05 (m, 2H), 2.03 (s, 3H), 2.01 – 1.93 (m, 2H), 1.92 – 1.79 (m, 2H), 1.75 – 1.66 (m, 1H), 1.65 – 1.57 (m, 1H), 1.56 – 1.45 (m, 3H), 1.45 – 1.39 (m, 2H), 1.34 – 1.26 (m, 2H), 1.22 – 1.16 (m, 1H), 1.03 (s, 3H), 0.87 (s, 3H), 0.82 – 0.73 (m, 1H).

^{13}C NMR (100 MHz, $CDCl_3$): δ 170.7, 86.8, 79.9, 74.6, 74.5, 71.2, 63.4, 62.4, 50.5, 49.8, 48.8, 38.5, 37.9, 36.7, 35.2, 31.7, 30.6, 29.4, 27.2, 23.0, 21.4, 17.2, 7.2.

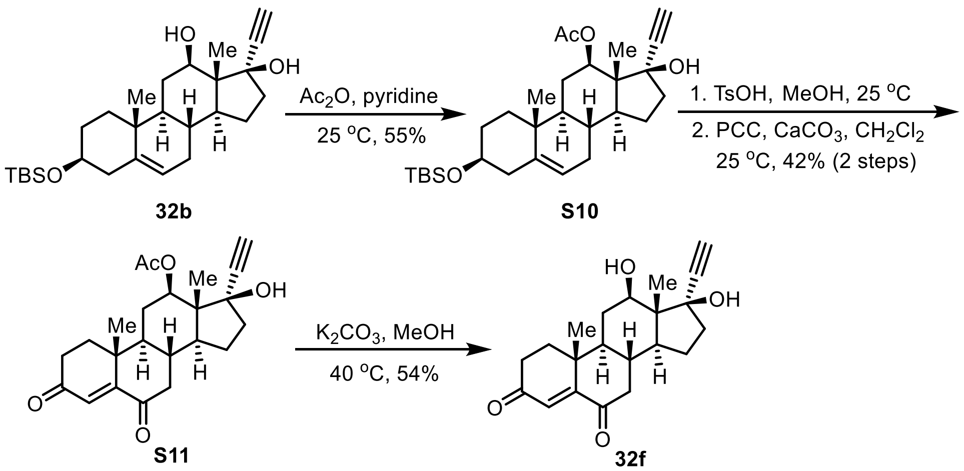

To a stirred solution of **32b** (1.00 g, 2.25 mmol) in anhydrous pyridine (22 mL) was added acetic anhydride (2.1 mL, 22.3 mmol). The mixture was stirred at 25 ℃ for 12 h before being quenched with saturated aqueous NH_4Cl (40 mL) and extracted with EtOAc (3 × 30 mL). The combined organic layers were washed with brine, dried over Na_2SO_4 and concentrated in *vacuo*. The residue was purified by flash column chromatography on silica gel (petroleum ether / ethyl acetate = 15:1 to 3:1 v/v) to furnish compound **S10** (600 mg, 55%) as a white solid.

To a stirred solution of **S10** (300 mg, 0.620 mmol) in methanol (12 ml) was added *p*-TsOH (318 mg, 1.85 mmol). After being stirred at 25 ℃ for 30 min, the reaction was quenched with saturated aqueous $NaHCO_3$ (6 mL) and extracted with EtOAc (3 × 20 mL). The combined organic layers were washed with brine, dried over Na_2SO_4 and concentrated in *vacuo*. The residue was dissolved in anhydrous CH_2Cl_2 (6.2 mL), to which were added pyridinium chlorochromate (PCC, 799 mg, 3.70 mmol) and $CaCO_3$ (371 mg, 3.70 mmol). The resultant mixture was stirred at 25 ℃ for 5 h, and then filtered through a pad of silica gel, which was rinsed with EtOAc repeatedly. Afterwards, the filtrate was concentrated in *vacuo*. The residue was purified by flash column chromatography on silica gel (dichloromethane / acetone = 40:1 to 15:1 v/v) to furnish compound **S11** (100 mg, 42%) as a white solid.

To a solution of **S11** (100 mg, 0.270 mmol) in MeOH (2.7 mL) was added of K_2CO_3 (112 mg, 0.810 mmol), the resulting mixture was stirred at 40 ℃ for 3 h. The mixture was quenched with saturated aqueous NH_4Cl (5 mL) and extracted with EtOAc (3 × 10 mL). The combined organic layers were washed with brine, dried over Na_2SO_4 and concentrated in *vacuo*. The residue was purified by flash column chromatography on silica gel (dichloromethane / acetone = 20:1 to 8:1 v/v) to furnish compound **32f** (48 mg, 54%) as a white solid.

Compound S10

TLC (petroleum ether / ethyl acetate, 3:1 v/v): R_f = 0.47.

OR: $[\alpha]_D^{25}$ = –85.9 (c = 0.90, $CHCl_3$).

IR (neat) ν_{max}: 3488, 3306, 2953, 2930, 1726, 1249, 1088, 772 cm^{-1}.

^{1}H NMR (400 MHz, $CDCl_3$): δ 5.31 (d, *J* = 5.2 Hz, 1H), 5.18 (dd, *J* = 11.2, 4.8 Hz, 1H), 3.51 – 3.41 (m, 1H), 2.60 (s, 1H), 2.35 – 2.26 (m, 1H), 2.26 – 2.15 (m, 2H), 2.06 (s, 3H), 2.03 – 1.95 (m,

3H), 1.94 (s, 1H), 1.79 – 1.67 (m, 3H), 1.59 – 1.47 (m, 4H), 1.47 – 1.36 (m, 2H), 1.22 – 1.13 (m, 1H), 1.12 – 1.03 (m, 1H), 1.01 (s, 3H), 0.98 (s, 3H), 0.88 (s, 9H), 0.05 (s, 6H).

^{13}C NMR (100 MHz, $CDCl_3$) δ 170.4, 141.4, 120.7, 86.2, 79.5, 78.2, 74.5, 72.4, 50.1, 49.9, 48.8, 42.7, 38.4, 37.3, 36.9, 31.9, 31.9, 31.1, 26.5, 26.0(×3), 23.0, 21.5, 19.4, 18.3, 8.6, −4.5(×2).

HRMS (ESI): *m/z* calcd. for $C_{29}H_{47}O_4Si$ $[M+H]^+$ 487.3238, found 487.3241.

Compound S11

TLC (dichloromethane / acetone, 8:1 v/v): R_f = 0.50.

OR: $[\alpha]_D^{25}$ = −78.2 (c = 0.50, $CHCl_3$).

IR (neat) ν_{max}: 3458, 3264, 2958, 1728, 1685, 1245, 1052, 751 cm^{-1}.

^{1}H NMR (400 MHz, $CDCl_3$): δ 6.19 (s, 1H), 5.24 (dd, *J* = 10.8, 4.8 Hz, 1H), 2.68 (dd, *J* = 16.2, 4.2 Hz, 1H), 2.64 (s, 1H), 2.52 – 2.46 (m, 2H), 2.37 – 2.30 (m, 1H), 2.13 – 2.08 (m, 3H), 2.07 (s, 3H), 2.06 – 2.01 (m, 3H), 1.99 – 1.91 (m, 2H), 1.80 – 1.73 (m, 1H), 1.72 – 1.66 (m, 1H), 1.65 – 1.59 (m, 1H), 1.50 – 1.43 (m, 2H), 1.25 – 1.21 (m, 1H), 1.18 (s, 3H), 1.02 (s, 3H).

^{13}C NMR (100 MHz, $CDCl_3$): δ 200.9, 199.0, 170.4, 159.5, 126.3, 85.6, 79.0, 76.9, 75.1, 49.9, 49.7, 49.0, 45.6, 39.4, 38.2, 35.5, 33.8, 33.8, 26.1, 22.6, 21.4, 17.7, 8.6.

HRMS (ESI): *m/z* calcd. for $C_{23}H_{29}O_5$ $[M+H]^+$ 385.2010, found 385.2015.

Compound 32f

TLC (dichloromethane / acetone, 4:1 v/v): R_f = 0.49.

OR: $[\alpha]_D^{25}$ = –40.0 (c = 0.13, $CHCl_3$).

IR (neat) ν_{max}: 3451, 3298, 2954, 2917, 1730, 1690, 1249, 1220, 1056, 771 cm^{-1}.

^{1}H NMR (400 MHz, CD_3OD): δ 6.08 (s, 1H), 4.20 (dd, *J* = 10.8, 5.2 Hz, 1H), 3.04 (s, 1H), 2.66 – 2.57 (m, 2H), 2.46 – 2.36 (m, 1H), 2.31 – 2.20 (m, 2H), 2.20 – 2.10 (m, 2H), 2.04 – 1.93 (m, 3H), 1.89 – 1.82 (m, 1H), 1.77 – 1.71 (m, 1H), 1.71 – 1.66 (m, 1H), 1.66 – 1.54 (m, 2H), 1.54 – 1.45 (m, 2H), 1.22 (s, 3H), 0.93 (s, 3H).

^{13}C NMR (100 MHz, CD_3OD): δ 203.1, 201.7, 162.6, 126.3, 87.7, 80.2, 75.8, 74.7, 52.1, 50.7, 50.5, 46.5, 40.6, 39.8, 36.5, 35.0, 34.7, 30.5, 23.3, 17.7, 7.8.

HRMS (ESI): *m/z* calcd. for $C_{21}H_{27}O_4$ $[M+H]^+$ 343.1904, found 343.1906.

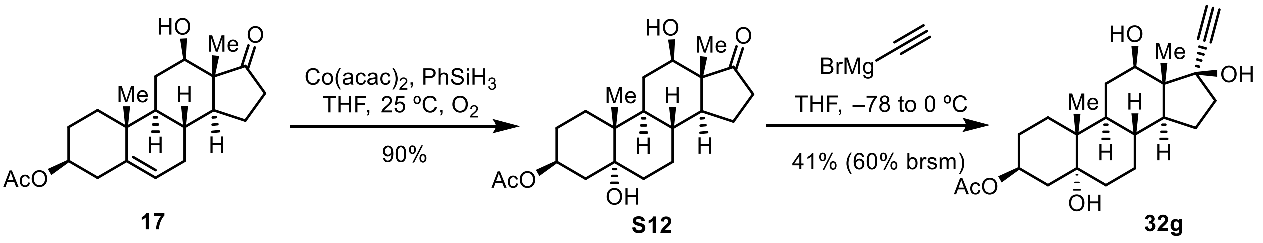

To a stirred solution of **17** (500 mg, 1.40 mmol) and Co(acac)$_2$ (103 mg, 0.29 mmol) in THF (29 mL) was added $PhSiH_3$ (0.390 mL, 3.20 mmol). The resultant mixture was stirred under an O_2 atmosphere (1 atm) at 25 °C for 5 h. Removal of solvent under reduced pressure afforded the crude residue, which was purified by flash column chromatography on silica gel (petroleum ether / acetone = 15:1 to 4:1 v/v) to furnish compound **S12** (475 mg, 90%) as a white solid.

Following the procedure for the conversion of **17** into **18**, substrate **32g** [92.0 mg, 41%, 60% yield based on recovery of **S12** (65.3 mg)] was prepared starting from **S12** (200 mg, 0.559 mmol) and purified through flash column chromatography (petroleum ether / ethyl acetate = 8:1 to 2:1 v/v).

Compound S12

TLC (petroleum ether / acetone, 2:1 v/v): R_f = 0.43.

OR: $[\alpha]_D^{25}$ = +28.9 (c = 1.60, $CHCl_3$).

IR (neat) ν_{max}: 3452, 2943, 1722, 1247, 1219, 1027, 770, 749 cm^{-1}.

^{1}H NMR (400 MHz, $CDCl_3$): δ 5.18 – 5.06 (m, 1H), 3.77 (dd, *J* = 10.8, 3.6 Hz, 1H), 3.01 (s, 1H), 2.42 (dd, *J* = 19.6, 8.8 Hz, 1H), 2.14 – 2.02 (m, 1H), 1.99 (s, 3H), 1.97 – 1.90 (m, 1H), 1.88 – 1.80 (m, 1H), 1.75 – 1.62 (m, 5H), 1.65 – 1.60 (m, 3H), 1.61 – 1.50 (m, 5H), 1.47 – 1.36 (m, 2H), 1.34 – 1.18 (m, 3H), 0.99 (s, 3H), 0.91 (s, 3H).

^{13}C NMR (150 MHz, $CDCl_3$): δ 222.9, 170.8, 74.6, 72.8, 70.7, 51.8, 48.9, 43.7, 40.1, 38.9, 35.8, 34.4, 33.5, 30.5, 28.3, 26.7, 24.3, 21.6, 21.5, 15.9, 8.3.

HRMS (ESI): *m/z* calcd. for $C_{21}H_{33}O_5$ $[M+H]^+$ 365.2323, found 365.2324.

Compound 32g

TLC (petroleum ether / acetone, 2:1 v/v): R_f = 0.35.

OR: $[\alpha]_D^{25}$ = –8.3 (c = 0.4, $CHCl_3$).

IR (neat) ν_{max}: 3415, 2942, 1715, 1370, 1250, 1219, 1031, 772 cm^{-1}.

^{1}H NMR (400 MHz, C_5D_5N): δ 6.26 (s, 1H), 5.79 – 5.67 (m, 1H), 5.62 (s, 1H), 5.18 (s, 1H), 4.60 (dd, *J* = 11.2, 4.8 Hz, 1H), 3.35 (s, 1H), 2.52 – 2.43 (m, 1H), 2.29 (td, *J* = 12.4, 4.0 Hz, 1H), 2.15 –

2.06 (m, 1H), 2.02 (s, 3H), 2.01 – 1.90 (m, 4H), 1.90 – 1.83 (m, 1H), 1.80 – 1.64 (m, 6H), 1.55 – 1.41 (m, 5H), 1.30 (s, 3H), 1.05 (s, 3H).

^{13}C NMR (100 MHz, C_5D_5N): δ 170.2, 89.3, 79.6, 74.6, 74.5, 73.5, 71.3, 51.5, 49.4, 44.0, 40.5, 39.7, 39.1, 35.0, 34.5, 31.0, 30.9, 27.2, 25.6, 23.2, 21.2, 15.7, 8.4.

HRMS (ESI): *m/z* calcd. for $C_{23}H_{35}O_5$ $[M+H]^+$ 391.2479, found 391.2480.

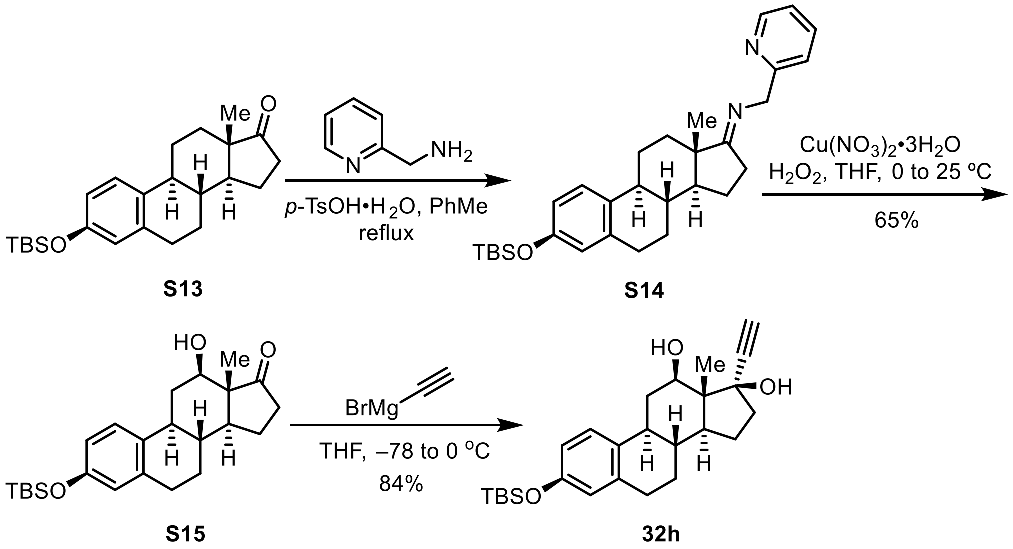

Following the procedure for the conversion of **S1** into **17**, alcohol **S15** (2.70 g, 65%) was prepared starting from **S13** (4.00 g, 10.4 mmol) and purified through flash column chromatography (petroleum ether / ethyl acetate = 10:1 to 1:1 v/v).

Following the procedure for the conversion of **17** into **18**, substrate **32h** (270 mg, 84%) was prepared starting from **S15** (300 mg, 0.749 mmol) and purified through flash column chromatography (petroleum ether / ethyl acetate = 10:1 to 1:1 v/v).

Compound S15

TLC (petroleum ether / ethyl acetate, 5:1 v/v): Rf = 0.28.

OR: $[\alpha]_D^{25}$ = +70.6 (c = 1.01, $CHCl_3$).

IR (neat): ν_{max}: 3564, 2928, 1729, 1608, 1571, 1496, 1471, 1416, 956, 669 cm^{-1}.

^{1}H NMR (400 MHz, $CDCl_3$): δ 7.10 (d, *J* = 8.4 Hz, 1H), 6.63 (dd, *J* = 8.4, 2.4 Hz, 1H), 6.59 – 6.55 (m, 1H), 3.97 (dd, *J* = 11.2, 4.4 Hz, 1H), 3.11 – 3.03 (m, 1H), 2.86 (dd, *J* = 9.2, 4.4 Hz, 2H), 2.58 – 2.45 (m, 2H), 2.41 – 2.29 (m, 1H), 2.22 – 1.96 (m, 3H), 1.80 – 1.66 (m, 1H), 1.64 – 1.36 (m, 4H), 0.99 (s, 3H), 0.98 (s, 9H), 0.19 (s, 6H).

^{13}C NMR (100 MHz, $CDCl_3$): δ 222.8, 153.7, 137.4, 131.7, 126.0, 120.1, 117.5, 72.8, 52.0, 48.5, 42.4, 37.3, 35.9, 33.0, 29.4, 26.3, 25.8 (× 3), 21.5, 18.2, 8.4, –4.2 (× 2).

HRMS (ESI): *m/z* calcd. for $C_{24}H_{37}O_3Si$ $[M+H]^+$ 401.2506, found 401.2507.

Compound 32h

TLC (petroleum ether / ethyl acetate, 2:1 v/v): R_f = 0.30.

OR: $[\alpha]_D^{25}$ = –4.1 (c = 1.07, $CHCl_3$).

IR (neat) ν_{max}: 3467, 3292, 3265, 2929, 1609, 1570, 1495, 1471, 1417, 750 cm^{-1}.

^{1}H NMR (400 MHz, $CDCl_3$): δ 7.09 (d, *J* = 8.4 Hz, 1H), 6.62 (dd, *J* = 8.4, 2.8 Hz, 1H), 6.59 – 6.54 (m, 1H), 4.32 (dd, *J* = 11.2, 4.8 Hz, 1H), 2.87 – 2.77 (m, 2H), 2.65 (s, 1H), 2.46 (dt, *J* = 12.4, 4.8 Hz, 1H), 2.39 – 2.27 (m, 2H), 2.11 – 1.99 (m, 1H), 1.93 – 1.76 (m, 2H), 1.70 – 1.26 (m, 7H), 0.97 (s, 9H), 0.94 (s, 3H), 0.19 (s, 6H).

^{13}C NMR (100 MHz, $CDCl_3$): δ 153.6, 137.8, 132.0, 126.0, 120.1, 117.4, 87.0, 80.0, 75.0, 74.5, 51.1, 48.4, 42.4, 38.6, 38.5, 35.0, 29.6, 27.0, 25.8 (× 3), 22.8, 18.3, 7.5, –4.2 (× 2).

HRMS (ESI): *m/z* calcd. for $C_{26}H_{39}O_3Si$ $[M+H]^+$ 427.2663, found 427.2667.

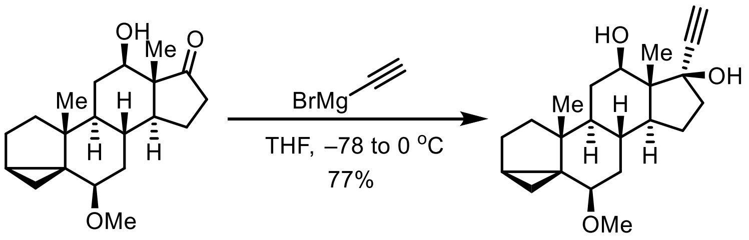

S16 **32i**

Following the procedure for the conversion of **17** into **18**, substrate **32i** (250 mg, 77%) was prepared starting from **S16** (300 mg, 0.942 mmol) and purified through flash column chromatography (petroleum ether / ethyl acetate = 6:1 to 1:1 v/v).

TLC (petroleum ether / ethyl acetate, 2:1 v/v): R_f = 0.30.

OR: $[\alpha]_D^{25}$ = –3.6 (c = 0.41, $CHCl_3$).

IR (neat) ν_{max}: 3403, 3304, 2954, 1151, 1134, 1080, 1065, 1050, 1028, 665 cm^{-1}.

^{1}H NMR (400 MHz, $CDCl_3$): δ 4.15 – 4.09 (m, 1H), 3.32 (s, 3H), 2.80 – 2.76 (m, 1H), 2.63 (s, 1H), 2.57 (s, 1H), 2.33 – 2.23 (m, 1H), 2.07 – 1.98 (m, 1H), 1.88 (dt, *J* = 13.2, 2.8 Hz, 1H), 1.84 – 1.73 (m, 3H), 1.70 – 1.63 (m, 2H), 1.58 – 1.48 (m, 3H), 1.47 – 1.38 (m, 2H), 1.10 – 1.02 (m, 1H), 1.05 (s, 3H), 0.98 – 0.85 (m, 3H), 0.95 (s, 3H), 0.67 (t, *J* = 4.4 Hz, 1H), 0.47 (dd, *J* = 8.0, 5.2 Hz, 1H).

^{13}**C NMR** (100 MHz, $CDCl_3$): δ 87.2, 82.0, 80.0, 75.1, 74.3, 56.7, 51.0, 49.2, 46.6, 43.5, 38.6, 35.1, 34.3, 33.4, 31.6, 30.2, 24.9, 23.0, 21.5, 19.4, 13.2, 7.6.

HRMS (ESI): *m/z* calcd. for $C_{22}H_{33}O_3$ $[M+H]^+$ 345.2424, found 345.2427.

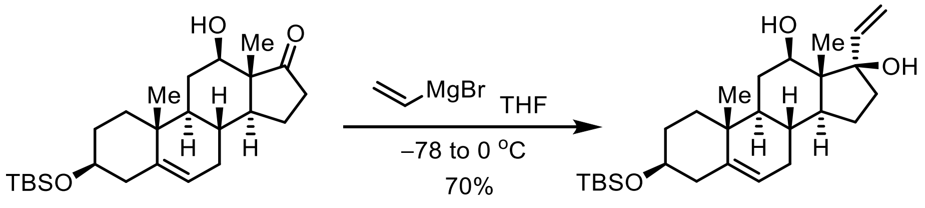

S6 **32j**

To a solution of **S6** (400 mg, 0.955 mmol) in anhydrous THF (20 mL) was slowly added vinylmagnesium bromide (1.0 M in THF, 9.6 mL, 9.55 mmol) under argon atmosphere at –78 ℃. The reaction was stirred at –78 ℃ for 30 min and stirred at 0 ℃ for 12 h, before it was quenched by saturated aqueous NH_4Cl (30 mL). The aqueous phase was extracted with EtOAc (3 × 30 mL). The organic layer was combined, washed with brine (30 mL), dried over anhydrous $MgSO_4$, filtered, and concentrated to dryness. Purification of crude product by flash column chromatography (petroleum ether / ethyl acetate = 20:1 to 3:1 v/v) yielded **32j** (302 mg, 70%) as a white solid.

TLC (petroleum ether / acetone, 4:1 v/v): R_f = 0.48.

OR: $[\alpha]_D^{25}$ = –54.4 (c = 0.81, $CHCl_3$).

IR (neat) ν_{max}: 3414, 2964, 2931, 2905, 2852, 1219, 1093, 887, 835, 772 cm^{-1}.

^{1}**H NMR** (400 MHz, $CDCl_3$): δ 6.16 (dd, *J* = 17.2, 10.8 Hz, 1H), 5.30 (s, 1H), 5.30 – 5.14 (m, 2H), 3.65 (dd, *J* = 11.2, 4.8 Hz, 1H), 3.50 – 3.40 (m, 1H), 2.30 – 2.12 (m, 2H), 2.08 – 1.82 (m, 5H), 1.80 – 1.63 (m, 5H), 1.57 – 1.40 (m, 5H), 1.26 – 1.17 (m, 1H), 1.02 (s, 4H), 0.97 (s, 3H), 0.87 (s, 9H), 0.04 (s, 6H).

^{13}**C NMR** (100 MHz, $CDCl_3$): δ 142.5, 141.6, 120.8, 112.7, 84.6, 74.1, 72.5, 50.5, 49.5, 49.0, 42.7, 37.4, 36.8, 35.9, 32.0, 31.7, 31.3, 29.6, 26.0 (× 3), 23.6, 19.5, 18.3, 8.6, –4.4 (× 2).

HRMS (ESI): *m/z* calcd. for $C_{27}H_{47}O_3Si$ $[M+H]^+$ 447.3289, found 447.3290.

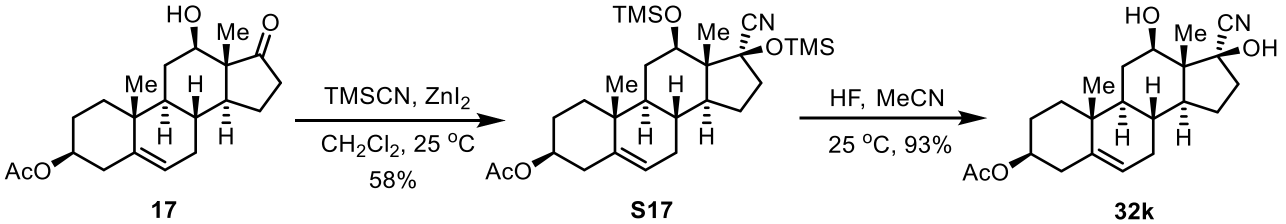

17 **S17** **32k**

A round-bottom flask was charged with compound **17** (300 mg, 0.866mmol), zinc iodide (55.2 mg, 0.173mmol), and anhydrous CH_2Cl_2 (3 mL) was added under argon at 25 ^oC, followed by dropwise addition of trimethylsilyl cyanide (1.2 mL, 9.01mmol). After the reaction was stirred at 25 ^oC for 12 h, the organic solvent was concentrated under reduced pressure. Purification of the crude product via flash column chromatography (petroleum ether / ethyl acetate= 50:1 to 5:1 v/v) yielded the product **S17** (260 mg, 58%) as a white solid.

To a solution of compound **S17** (150 mg, 0.289mmol) in anhydrous acetonitrile (3 mL) was slowly added 40% hydrofluoric acid aqueous solution (60 μL, 1.34 mmol) at 25 ^oC. The reaction was stirred at 25 ^oC for 1 h, and then the organic solvent was concentrated under reduced pressure. Flash column chromatography of the residue on silica gel (petroleum ether / ethyl acetate = 10:1 to 1:1 v/v) gave diol **32k** (100 mg, 93%) as a white foam.

Compound S17

TLC (petroleum ether / ethyl acetate, 5:1 v/v): R_f = 0.58.

OR: $[\alpha]_D^{25}$ = –66.4 (c = 0.73, $CHCl_3$).

IR (neat): ν_{max}: 3020, 2958, 1728, 1438, 1365, 1251, 1214, 843, 749, 667 cm^{-1}.

^{1}H NMR (400 MHz, $CDCl_3$): δ 5.41 – 5.33 (m, 1H), 4.65 – 4.54 (m, 1H), 4.00 (dd, *J* = 10.8, 4.8 Hz, 1H), 2.45 – 2.24 (m, 3H), 2.03 (s, 3H), 2.01 – 1.89 (m, 2H), 1.88 – 1.63 (m, 4H), 1.59 – 1.37 (m, 5H), 1.36 – 1.28 (m, 1H), 1.21 – 1.07 (m, 2H), 1.03 (s, 3H), 0.85 (s, 3H), 0.22 (s, 9H), 0.15 (s, 9H).

^{13}C NMR (100 MHz, $CDCl_3$): δ 170.6, 139.7, 122.5, 122.1, 81.8, 75.2, 73.8, 52.1, 48.6, 48.4, 38.3, 38.1, 37.0, 36.6, 31.7, 31.6, 31.0, 27.7, 23.0, 21.5, 19.3, 7.4, 1.6 (×3), 1.0 (×3).

HRMS (ESI): *m/z* calcd. for $C_{28}H_{48}NO_4Si_2$ $[M+H]^+$ 518.3116, found 518.3119.

Compound 32k

TLC (petroleum ether / ethyl acetate, 1:1 v/v): R_f = 0.46.

OR: $[\alpha]_D^{25}$ = –70.9 (c = 0.90, $CHCl_3$).

IR (neat): ν_{max}: 3462, 3019, 2955, 1724, 1439, 1366, 1256, 1030, 748, 667 cm^{-1}.

^{1}H NMR (400 MHz, $CDCl_3$): δ 5.42 – 5.36 (m, 1H), 4.64 – 4.54 (m, 1H), 4.08 (dd, *J* = 11.2, 4.8 Hz, 1H), 2.53 – 2.44 (m, 1H), 2.41 – 2.26 (m, 2H), 2.10 – 1.97 (m, 5H), 1.92 – 1.74 (m, 4H), 1.65 – 1.46 (m, 5H), 1.43 – 1.32 (m, 1H), 1.21 – 1.10 (m, 2H), 1.05 (s, 3H), 0.92 (s, 3H).

^{13}C NMR (100 MHz, $CDCl_3$): δ 170.7, 139.6, 122.0 (× 2), 81.2, 75.3, 73.7, 50.6, 49.7, 48.9, 37.9, 37.0, 36.8, 36.2, 31.6, 31.0, 30.5, 27.7, 23.1, 21.5, 19.4, 7.1.

HRMS (ESI): *m/z* calcd. for $C_{22}H_{32}NO_4$ $[M+H]^+$ 374.2326, found 374.2325.

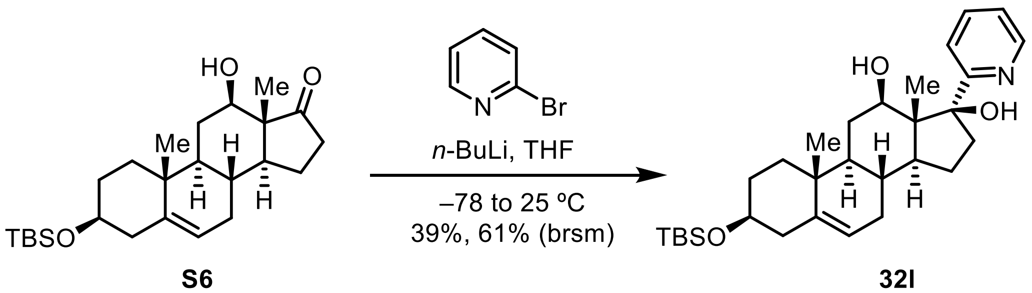

To a solution of 2-bromopyridine (683 μL, 7.17 mmol) in THF (24 mL) was slowly added *n*-BuLi (2.9 mL, 7.17 mmol) under argon atmosphere at –78 ℃. After being stirred at –78 ℃ for 2 h, to the mixture was added dropwise a solution of ketone **S6** (300 mg, 0.717 mmol) in THF (2 mL). The reaction mixture was stirred at 25 ^{o}C for 24 h and quenched by saturated NH_4Cl solution (25 mL) and extracted with EtOAc (3 × 25 mL). The organic extracts were combined, washed with brine (25 mL), dried over anhydrous Na_2SO_4, filtered, and concentrated to dryness. The residue was purified via flash column chromatography on silica gel (petroleum ether / ethyl acetate = 5/1 to 1/1 v/v) to give **32l** (140 mg, 39%) as a white solid, together with recovered **S6** (106 mg).

TLC (petroleum ether / ethyl acetate, 2:1 v/v): R_f = 0.24.

OR: $[\alpha]_D^{25}$ = –21.9 (c = 0.77, $CHCl_3$).

IR (neat) ν_{max}: 3489, 2929, 2855, 1591, 1466, 1435, 1369, 1091, 1060, 772 cm^{-1}.

^{1}H NMR (400 MHz, $CDCl_3$): δ 8.61 – 8.54 (m, 1H), 7.79 – 7.71 (m, 1H), 7.31 – 7.26 (m, 2H), 5.30 – 5.25 (m, 1H), 4.24 (s, 1H), 3.44 – 3.33 (m, 1H), 3.28 (s, 1H), 2.42 (dd, *J* = 10.0, 6.0 Hz, 1H), 2.31 – 2.18 (m, 3H), 2.18 – 2.10 (m, 1H), 2.09 – 2.00 (m, 1H), 1.90 – 1.77 (m, 1H), 1.77 – 1.56 (m, 4H), 1.55 – 1.42 (m, 5H), 1.12 (s, 3H), 1.10 – 1.03 (m, 1H), 1.00 (s, 3H), 0.93 – 0.88 (m, 1H), 0.86 (s, 9H), 0.02 (s, 6H).

^{13}C NMR (100 MHz, $CDCl_3$): δ 164.8, 148.2, 141.8, 137.0, 122.6, 121.3, 120.6, 86.8, 74.8, 72.6, 52.5, 49.3, 49.3, 42.8, 37.3, 36.8, 35.4, 32.1, 32.0, 31.4, 28.4, 26.0 (× 3), 23.6, 19.4, 18.3, 9.4, –4.4 (× 2).

HRMS (ESI): *m/z* calcd. for $C_{30}H_{48}NO_3Si$ $[M+H]^+$ 498.3398, found 498.3394.

2.3 The biomimetic rearrangement reaction

Supplementary Table 5. Screening of conditions for the biomimetic rearrangement reaction[a]

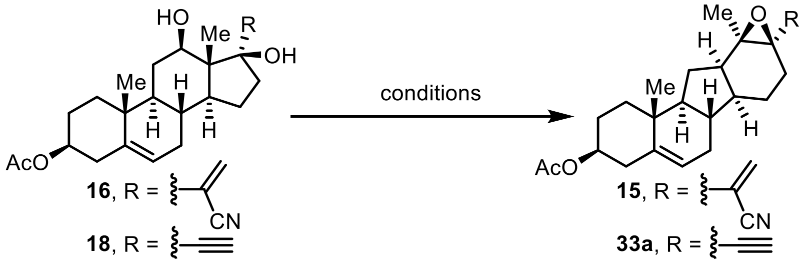

Entry	Substrate	Activator	Base	Solvent	Tem. (℃)	Conv (%)	Yield (%)
1	**16**	Tf_2O	Lutidine	CH_2Cl_2	–78 to 0	trace	trace
2	**16**	Tf_2O	Piperidine	CH_2Cl_2	–78 to 0	trace	trace
3	**16**	Tf_2O	DIPEA	CH_2Cl_2	–78 to 0	trace	trace
4	**16**	Tf_2O	Pyridine	CH_2Cl_2	–78 to 0	trace	trace
5[b]	**16**	Tf_2O	2-F-Py.	CH_2Cl_2	–78	90	39
6[b]	**16**	Tf_2O	2-Br-Py.	CH_2Cl_2	–78	90	52
7[b]	**16**	Tf_2O	2-Cl-Py.	CH_2Cl_2	–78	95	66
8	**16**	MsCl	2-Cl-Py.	CH_2Cl_2	–78 to 0	trace	trace
9	**16**	TsCl	2-Cl-Py.	CH_2Cl_2	–78 to 0	trace	trace
10	**16**	Tf_2NPh	2-Cl-Py.	CH_2Cl_2	–78 to 0	trace	trace
11[b]	**16**	Tf_2O	2-Cl-Py.	THF	–78	83	12
12[b]	**16**	Tf_2O	2-Cl-Py.	PhMe	–78	90	31
13[b]	**16**	Tf_2O	2-Cl-Py.	Hexane	–78	90	25
14	**18**	Tf_2O	2-Cl-Py.	CH_2Cl_2	–78	95	69
15	**18**	Tf_2O	Pyridine	CH_2Cl_2	–78 to 0	95	84

[a]Unless otherwise noted, all reactions were carried out with substrate (1.0 equiv.), activator (3.0 equiv.), base (7.5 equiv.) in dry solvent (c = 0.05 M) at indicated temperature for 2 h. The conversions and yields were calculated based on the isolated material. [b]Formation of different ratios of homoallylic alcohol **19** was observed.

General procedure A: To a solution of substrate (1.0 equiv.) in anhydrous CH_2Cl_2 (0.05 M) was added pyridine (7.5 equiv.) at –78 ℃. After stirring for 10 min, Tf_2O (3.0 equiv.) was added at the same temperature. The mixture was stirred at –78 ℃ for 30 min before being warmed to 0 ℃ and stirred for 2 h. The mixture was quenched with saturated aqueous $NaHCO_3$ and extracted with

CH_2Cl_2. The combined organic layers were washed with brine, dried over Na_2SO_4 and concentrated in *vacuo*. The residue was purified by flash column chromatography on silica gel to furnish the corresponding product.

General procedure B: To a solution of substrate (1.0 equiv.) in anhydrous CH_2Cl_2 (0.05 M) was added 2-chloropyridine (7.5 equiv.) at –78 ℃. After stirring for 10 min, Tf_2O (3.0 equiv.) was added at the same temperature. The reaction mixture was kept stirring at –78 ℃ for 2 h and quenched with saturated aqueous $NaHCO_3$ and extracted with CH_2Cl_2. The combined organic layers were washed with brine, dried over Na_2SO_4 and concentrated in *vacuo*. The residue was purified by flash column chromatography on silica gel to furnish the corresponding product.

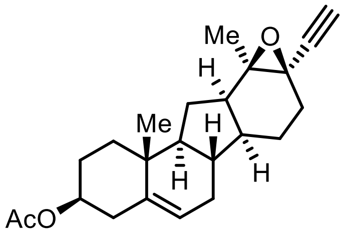

33a

Prepared according to general procedure A using **18** (32.0 mg, 0.086 mmol). The crude product was subjected to flash column chromatography (petroleum ether / ethyl acetate = 100/1 to 10/1 v/v) to afford **33a** as a white solid (25.6 mg, 84%).

TLC (petroleum ether / ethyl acetate, 20:1 v/v): R_f = 0.46.

OR: $[\alpha]_D^{25}$ = –60.1 (c = 0.91, $CHCl_3$).

IR (neat) ν_{max}: 3249, 3019, 2949, 1716, 1251, 1139, 1083, 1035, 753, 639 cm^{-1}.

^{1}H NMR (400 MHz, $CDCl_3$): δ 5.40 – 5.34 (m, 1H), 4.65 – 4.54 (m, 1H), 2.39 (s, 1H), 2.38 – 2.34 (m, 1H), 2.31 – 2.25 (m, 1H), 2.25 – 2.16 (m, 2H), 2.16 – 2.12 (m, 1H), 2.03 (s, 3H), 1.97 – 1.91 (m, 1H), 1.90 – 1.77 (m, 3H), 1.73 – 1.62 (m, 2H), 1.60 – 1.52 (m, 1H), 1.47 (s, 3H), 1.45 – 1.35 (m, 2H), 1.34 – 1.25 (m, 2H), 1.24 – 1.14 (m, 2H), 0.99 (s, 3H).

^{13}C NMR (100 MHz, $CDCl_3$): δ 170.6, 140.6, 123.3, 82.9, 74.0, 72.7, 64.9, 57.2, 53.3, 44.3, 40.9, 40.4, 38.2, 37.6, 36.9, 32.3, 29.3, 28.0, 27.6, 24.4, 21.5, 21.2, 18.7.

HRMS (ESI): *m/z* calcd. for $C_{23}H_{31}O_3$ $[M+H]^+$ 355.2268, found 355.2271.

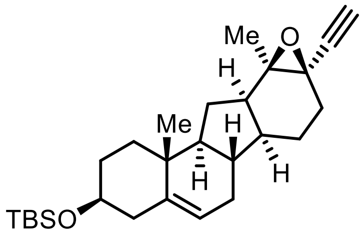

33b

Prepared according to general procedure A using **32b** (35 mg, 0.079 mmol). The crude product was subjected to flash column chromatography (petroleum ether / ethyl acetate = 150:1 to 50:1 v/v) to afford **33b** as a white solid (27.4 mg, 81%).

TLC (petroleum ether / ethyl acetate, 50:1 v/v): R_f = 0.49.

OR: $[\alpha]_D^{25}$ = –44.5 (c = 0.83, $CHCl_3$).

IR (neat) ν_{max}: 3310, 2929, 2892, 2855, 1462, 1437, 1379, 1250, 1091, 875 cm^{-1}.

^{1}H NMR (400 MHz, $CDCl_3$): δ 5.33 – 5.28 (m, 1H), 3.51 – 3.43 (m, 1H), 2.38 (s, 1H), 2.27 – 2.13 (m, 5H), 1.97 – 1.83 (m, 2H), 1.78 – 1.68 (m, 2H), 1.66 – 1.59 (m, 1H), 1.57 – 1.52 (m, 1H), 1.47 (s, 3H), 1.45 – 1.27 (m, 5H), 1.20 – 1.09 (m, 2H), 0.97 (s, 3H), 0.89 (s, 9H), 0.05 (s, 6H).

^{13}C NMR (100 MHz, $CDCl_3$): δ 142.6, 121.8, 83.0, 72.9, 72.7, 65.0, 57.2, 53.5, 44.4, 42.4, 41.0, 40.6, 38.7, 37.0, 32.3, 31.9, 29.4, 28.1, 26.0 (×3), 24.4, 21.2, 18.8, 18.4, –4.4 (×2).

HRMS (ESI): *m/z* calcd. for $C_{27}H_{43}O_2Si$ $[M+H]^+$ 427.3027, found 427.3030.

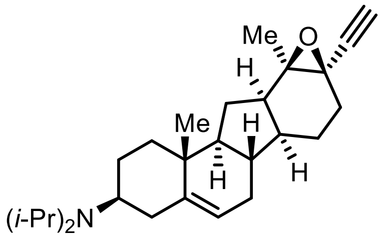

33c

Prepared according to general procedure A using **32c** (30.0 mg, 0.073 mmol). The crude product was subjected to flash column chromatography (dichloromethane / acetone = 10:1 to 1:1 v/v) to afford **33c** as a white solid (22.2 mg, 77%).

TLC (dichloromethane / acetone, 1:1 v/v): R_f = 0.47.

OR: $[\alpha]_D^{25}$ = –32.2 (c = 0.50, $CHCl_3$).

IR (neat) ν_{max}: 3310, 2959, 2931, 1728, 1455, 1219, 1116, 1073, 772, 650cm^{-1}.

^{1}H NMR (400 MHz, $CDCl_3$): δ 5.29 – 5.23 (m, 1H), 3.20 – 3.09 (m, 2H), 2.56 (tt, *J* = 12.0, 4.0 Hz, 1H), 2.38 (s, 1H), 2.36 – 2.27 (m, 1H), 2.25 – 2.11 (m, 3H), 1.98 – 1.83 (m, 3H), 1.77 (dt, *J* = 13.2, 3.4 Hz, 1H), 1.73 – 1.62 (m, 2H), 1.61 – 1.52 (m, 1H), 1.47 (s, 3H), 1.45 – 1.39 (m, 2H), 1.39 – 1.25 (m, 3H), 1.23 – 1.13 (m, 2H), 1.03 – 0.97 (m, 12H), 0.95 (s, 3H).

^{13}C NMR (100 MHz, $CDCl_3$): δ 144.8, 120.2, 83.0, 72.6, 65.0, 57.2, 54.9, 53.8, 44.7 (× 2), 44.5, 41.0, 40.6, 40.3, 39.7, 37.0, 32.4, 29.4, 29.2, 28.1, 24.4, 23.4 (×2), 23.3 (×2), 21.2, 18.9.

HRMS (ESI): *m/z* calcd. for $C_{27}H_{42}NO$ $[M+H]^+$ 396.3261, found 396.3260.

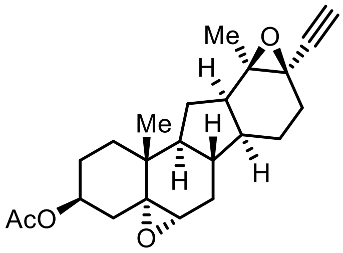

33d

Prepared according to general procedure A using **32d** (30.0 mg, 0.077 mmol). The crude product was subjected to flash column chromatography (petroleum ether / ethyl acetate = 20:1 to 10:1 v/v) to afford **33d** as a white solid (18.6 mg, 68%).

TLC (petroleum ether / ethyl acetate, 4:1 v/v): R_f = 0.39.

OR: $[\alpha]_D^{25}$ = –24.8 (c = 1.0, $CHCl_3$).

IR (neat) ν_{max}:3263, 2931, 1732, 1224, 1140, 1091, 1036, 772 cm^{-1}.

^{1}H NMR (400 MHz, $CDCl_3$): δ 5.02 – 4.90 (m, 1H), 2.91 (d, *J* = 4.4 Hz, 1H), 2.38 (s, 1H), 2.22 – 2.06 (m, 4H), 2.01 (s, 3H), 1.95 – 1.85 (m, 1H), 1.76 – 1.69 (m, 1H), 1.67 (s, 1H), 1.65 – 1.57 (m, 2H), 1.57 – 1.48 (m, 3H), 1.42 (s, 3H), 1.36 (ddd, *J* = 12.8, 5.2, 2.0 Hz, 1H), 1.32 – 1.27 (m, 1H), 1.27 – 1.14 (m, 4H), 1.03 (s, 3H).

^{13}C NMR (100 MHz, $CDCl_3$): δ 170.3, 82.7, 72.8, 71.5, 66.2, 64.9, 59.9, 57.1, 45.1, 42.2, 41.4, 40.5, 35.5, 34.6, 33.3, 29.0, 28.9, 27.6, 27.0, 24.2, 21.4, 20.8, 15.2.

HRMS (ESI): *m/z* calcd. for $C_{23}H_{31}O_4$ $[M+H]^+$ 371.2217, found 371.2213.

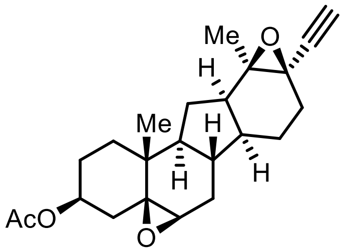

33e

Prepared according to general procedure A using **32e** (30.0 mg, 0.077 mmol). The crude product was subjected to flash column chromatography (petroleum ether / ethyl acetate = 20:1 to 10:1 v/v) to afford **33e** (18.0 mg, 65%) as colorless oil.

TLC (petroleum ether / ethyl acetate, 4:1 v/v): R_f = 0.39.

OR: $[\alpha]_D^{25}$ = –21.9 (c = 1.0, $CHCl_3$).

IR (neat) ν_{max}: 3303, 2943, 2869, 1729, 1241, 1141, 1095, 1036 cm^{-1}.

^{1}H NMR (400 MHz, $CDCl_3$): δ 4.82 – 4.71 (m, 1H), 3.07 (d, *J* = 2.4 Hz, 1H), 2.38 (s, 1H), 2.23 (dd, *J* = 10.8, 2.8 Hz, 1H), 2.17 – 2.06 (m, 2H), 2.02 (s, 3H), 1.95 – 1.80 (m, 3H), 1.79 – 1.72 (m, 1H), 1.56 – 1.45 (m, 3H), 1.43 (s, 3H), 1.41 – 1.30 (m, 5H), 1.29 – 1.22 (m, 2H), 0.96 (s, 3H), 0.93 – 0.86 (m, 1H).

^{13}C NMR (100 MHz, $CDCl_3$): δ 170.7, 82.7, 72.8, 71.2, 65.1, 64.5, 62.6, 57.0, 53.4, 40.7, 40.4, 39.1, 37.5, 37.2, 34.7, 32.5, 29.2, 28.1, 27.1, 24.0, 21.4, 21.1, 16.4.

HRMS (ESI): *m/z* calcd. for $C_{23}H_{31}O_4$ $[M+H]^+$ 371.2217, found 371.2215.

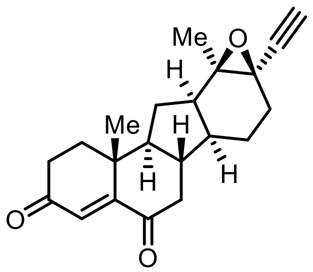
33f

Prepared according to general procedure A using **32f** (25.0 mg, 0.076 mmol). The crude product was subjected to flash column chromatography (petroleum ether / ethyl acetate = 30:1 to 10:1 v/v) to afford **33f** as a white solid (10.5 mg, 45%).

TLC (petroleum ether / ethyl acetate, 3:1 v/v): R_f = 0.28.

OR: $[\alpha]_D^{25}$ = –26.6 (c = 0.40, $CHCl_3$).

IR (neat) ν_{max}: 3238, 2926, 2855, 1678, 1218, 1138, 1091, 770 cm^{-1}.

^{1}H NMR (400 MHz, $CDCl_3$) δ 6.25 (s, 1H), 2.85 (dd, *J* = 16.0, 4.4 Hz, 1H), 2.68 – 2.55 (m, 1H), 2.53 – 2.45 (m, 1H), 2.42 (s, 1H), 2.41 – 2.34 (m, 1H), 2.25 – 2.13 (m, 2H), 2.13 – 2.05 (m, 1H), 2.05 – 1.93 (m, 3H), 1.83 – 1.71 (m, 1H), 1.70 – 1.55 (m, 4H), 1.49 (s, 3H), 1.44 – 1.35 (m, 1H), 1.15 (s, 3H).

^{13}C NMR (100 MHz, $CDCl_3$) δ 201.3, 199.5, 160.0, 127.3, 82.3, 73.2, 64.7, 57.2, 52.9, 46.5, 44.5, 40.6, 40.4, 37.9, 36.9, 34.0, 28.6, 27.5, 23.6, 20.7, 17.2.

HRMS (ESI): *m/z* calcd. for $C_{21}H_{25}O_3$ $[M+H]^+$ 325.1798, found 325.1799.

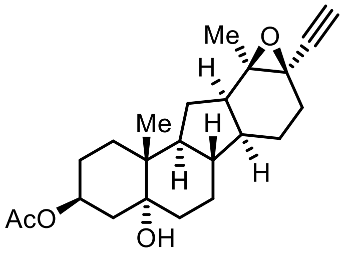
33g

Prepared according to general procedure A using **32g** (32.0 mg, 0.082 mmol). The crude product was subjected to flash column chromatography (petroleum ether / ethyl acetate = 20:1 to 10:1 v/v) to afford **33g** as a white solid (15.6 mg, 51%).

TLC (petroleum ether / ethyl acetate, 4:1 v/v): R_f = 0.40.

OR: $[\alpha]_D^{25}$ = +0.86 (c = 0.5, $CHCl_3$).

IR (neat) ν_{max}: 3467, 3306, 2928, 2862, 1715, 1246, 1143, 1086 cm^{-1}.

^{1}H NMR (400 MHz, $CDCl_3$): δ 5.25 – 5.13 (m, 1H), 2.39 (s, 1H), 2.27 – 2.18 (m, 1H), 2.14 (dt, *J* = 14.8, 3.6 Hz, 1H), 2.02 (s, 3H), 1.99 – 1.92 (m, 1H), 1.92 – 1.83 (m, 1H), 1.82 – 1.69 (m, 4H), 1.69 – 1.56 (m, 6H), 1.43 (s, 3H), 1.38 – 1.31 (m, 2H), 1.30 – 1.20 (m, 4H), 1.17 – 1.11 (m, 1H), 0.95 (s, 3H).

^{13}C NMR (100 MHz, $CDCl_3$): δ 170.8, 82.9, 75.4, 72.7, 71.0, 65.3, 57.2, 47.4, 46.9, 40.2, 39.6, 39.5, 38.6, 35.4, 31.2, 28.9, 28.1, 26.5, 25.6, 24.0, 21.6, 20.6, 15.1.

HRMS (ESI): *m/z* calcd. for $C_{23}H_{33}O_4$ $[M+H]^+$ 373.2373, found 373.2370.

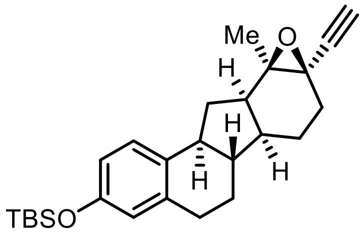

33h

Prepared according to general procedure A using **32h** (32.0 mg, 0.075 mmol). The crude product was subjected to flash column chromatography (petroleum ether / ethyl acetate = 200/1 to 60/1 v/v) to afford **33h** as a white solid (22.4 mg, 73%).

TLC (petroleum ether / ethyl acetate, 30:1 v/v): R_f = 0.52.

OR: $[\alpha]_D^{25}$ = +26.9 (c = 1.80, $CHCl_3$).

IR (neat) ν_{max}: 3310, 2928, 1608, 1570, 1497, 1472, 1415, 1152, 1088, 773 cm^{-1}.

^{1}H NMR (400 MHz, $CDCl_3$): δ 6.98 (d, *J* = 8.0 Hz, 1H), 6.63 – 6.55 (m, 2H), 2.94 – 2.77 (m, 2H), 2.57 – 2.32 (m, 4H), 2.19 (dt, *J* = 14.4, 3.6 Hz, 1H), 2.08 – 1.94 (m, 2H), 1.75 – 1.64 (m, 1H), 1.63 – 1.54 (m, 2H), 1.52 (s, 3H), 1.50 – 1.27 (m, 3H), 0.98 (s, 9H), 0.18 (s, 6H).

^{13}C NMR (100 MHz, $CDCl_3$): δ 153.6, 138.0, 133.5, 126.4, 119.9, 117.0, 82.9, 72.8, 65.1, 57.1, 52.3, 45.0, 41.0, 39.4, 32.3, 30.0, 28.9, 27.1, 25.8 (×3), 23.9, 20.6, 18.3, 4.2 (×2).

HRMS (ESI): *m/z* calcd. for $C_{26}H_{37}O_2Si$ $[M+H]^+$ 409.2557, found 409.2555

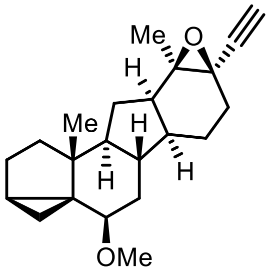

33i

Prepared according to general procedure A using **32i** (30.0 mg, 0.087 mmol). The crude product was subjected to flash column chromatography (petroleum ether / ethyl acetate = 100:1 to 10:1 v/v) to afford **33i** as a white solid (23.3 mg, 82%).

TLC (petroleum ether / ethyl acetate, 10:1 v/v): R_f = 0.47.

OR: $[\alpha]_D^{25}$ = +34.2 (c = 0.62, $CHCl_3$).

IR (neat) ν_{max}: 3307, 2927, 2868, 1218, 1139, 1086, 1012, 770, 752, 665 cm^{-1}.

^{1}H NMR (400 MHz, $CDCl_3$): δ 3.28 (s, 3H), 2.79 – 2.77 (m, 1H), 2.39 (s, 1H), 2.23 – 2.13 (m, 2H), 2.05 (dt, *J* = 13.2, 3.2 Hz, 1H), 1.97 – 1.88 (m, 1H), 1.86 – 1.75 (m, 2H), 1.63 – 1.51 (m, 3H), 1.45 (s, 3H), 1.43 – 1.31 (m, 4H), 1.19 – 1.10 (m, 2H), 1.02 – 0.98 (m, 4H), 0.95 – 0.88 (m, 1H), 0.67 – 0.60 (m, 1H), 0.47 (dd, *J* = 8.0, 5.2 Hz, 1H).

^{13}C NMR (100 MHz, $CDCl_3$): δ 83.4, 83.0, 72.6, 65.0, 57.1, 56.7, 50.0, 43.1, 42.5, 40.3, 40.0, 35.5, 34.8, 34.7, 29.2, 29.2, 25.0, 24.0, 21.8, 20.9, 18.1, 12.2.

HRMS (ESI): *m/z* calcd. for $C_{22}H_{31}O_2$ $[M+H]^+$ 327.2319, found 327.2320.

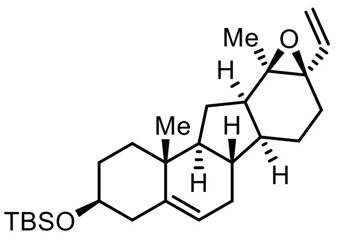

33j

Prepared according to general procedure A using **32j** (35.0 mg, 0.079 mmol). The crude product was subjected to flash column chromatography (petroleum ether / ethyl acetate = 150:1 to 50:1 v/v) to afford **33j** as a white solid (24.5 mg, 73%).

TLC (petroleum ether / ethyl acetate, 50:1 v/v): R_f = 0.47.

OR: $[\alpha]_D^{25}$ = –41.2 (c = 1.10, $CHCl_3$).

IR (neat) ν_{max}: 2927, 2901, 2855, 1689, 1471, 1437, 1077, 1005, 987, 916, 875 cm^{-1}.

^{1}H NMR (600 MHz, $CDCl_3$) δ 5.94 (dd, *J* = 17.4, 10.8 Hz, 1H), 5.38 – 5.21 (m, 3H), 3.53 – 3.43 (m, 1H), 2.27 – 2.13 (m, 4H), 1.90 – 1.84 (m, 2H), 1.79 – 1.65 (m, 3H), 1.64 – 1.53 (m, 3H), 1.49 – 1.40 (m, 2H), 1.40 – 1.32 (m, 2H), 1.25 (s, 3H), 1.21 – 1.11 (m, 2H), 0.98 (s, 3H), 0.89 (s, 9H), 0.06 (s, 6H).

^{13}C NMR (100 MHz, $CDCl_3$) δ 142.6, 137.4, 121.9, 116.7, 72.9, 65.6, 65.4, 53.6, 44.6, 42.4, 41.9, 41.0, 38.7, 37.0, 32.4, 31.9, 28.1, 27.6, 26.0 (×3), 24.9, 19.4, 18.8, 18.4, -4.5 (×2).

HRMS (ESI): *m/z* calcd. for $C_{27}H_{45}O_2Si$ $[M+H]^+$ 429.3183, found 429.3185.

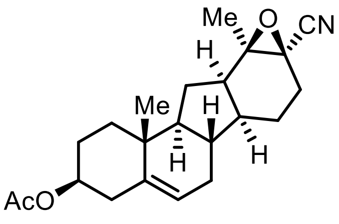

33k

Prepared according to general procedure B using **32k** (30.0 mg, 0.080 mmol). The crude product was subjected to flash column chromatography (petroleum ether / ethyl acetate = 50:1 to 5:1 v/v) to afford **33k** as a white solid (19.4 mg, 68%).

TLC (petroleum ether / ethyl acetate, 5:1 v/v): R_f = 0.52.

OR: $[\alpha]_D^{25}$ = –59.7 (c = 0.97, $CHCl_3$).

IR (neat) ν_{max}: 2961, 2920, 2943, 2881, 2829, 1724, 1247, 1093, 1035, 961 cm^{-1}.

^{1}H NMR (400 MHz, $CDCl_3$): δ 5.40 – 5.34 (m, 1H), 4.66 – 4.53 (m, 1H), 2.43 – 2.33 (m, 1H), 2.32 – 2.22 (m, 3H), 2.21 – 2.12 (m, 1H), 2.08 – 1.98 (m, 4H), 1.96 – 1.76 (m, 3H), 1.74 – 1.61 (m, 3H), 1.56 (s, 3H), 1.52 – 1.26 (m, 5H), 1.24 – 1.16 (m, 1H), 0.98 (s, 3H).

^{13}C NMR (100 MHz, $CDCl_3$): δ 170.6, 140.6, 123.1, 118.7, 73.9, 65.0, 55.1, 53.1, 44.1, 40.4, 39.9, 38.2, 37.6, 36.9, 32.1, 28.2, 27.6, 26.9, 23.5, 21.5, 21.4, 18.6.

HRMS (ESI): *m/z* calcd. for $C_{22}H_{30}NO_3$ $[M+H]^+$ 356.2220, found 356.2223.

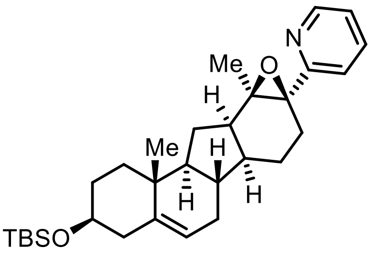

33l

Prepared according to general procedure B using **32l** (45.0 mg, 0.089 mmol). The crude product was purified by flash column chromatography (petroleum ether / diethyl ether = 20:1 to 2:1 v/v) to afford **33l** (22.5 mg, 52%) and **S18** (6.50 mg, 15%) both as a white solid.

TLC (petroleum ether / diethyl ether, 2:1 v/v): R_f = 0.47.

OR: $[\alpha]_D^{25}$ = –9.0 (c = 0.37, $CHCl_3$).

IR (neat) ν_{max}: 2928, 2856, 1592, 1471, 1436, 1380, 1082, 1006, 748, 608 cm^{-1}.

^{1}H NMR (400 MHz, $CDCl_3$): δ 8.59 – 8.53 (m, 1H), 7.66 (td, *J* = 7.6, 2.0 Hz, 1H), 7.38 (d, *J* = 7.6 Hz, 1H), 7.21 – 7.14 (m, 1H), 5.37 – 5.31 (m, 1H), 3.56 – 3.42 (m, 1H), 2.52 – 2.42 (m, 1H), 2.37 – 2.16 (m, 4H), 1.99 – 1.86 (m, 2H), 1.81 – 1.66 (m, 4H), 1.65 – 1.57 (m, 2H), 1.56 – 1.45 (m, 2H), 1.44 – 1.36 (m, 1H), 1.23 – 1.11 (m, 2H), 1.04 – 0.96 (m, 6H), 0.89 (s, 9H), 0.06 (s, 6H).

^{13}C NMR (100 MHz, $CDCl_3$): δ 161.1, 148.9, 142.5, 136.3, 122.1, 122.0, 121.0, 72.9, 68.3, 66.1, 53.6, 44.7, 42.4, 41.8, 40.8, 38.7, 37.0, 32.4, 32.0, 28.5, 28.1, 26.0 (×3), 25.3, 19.8, 18.8, 18.4, –4.4 (×2).

HRMS (ESI): *m/z* calcd. for $C_{30}H_{46}NO_2Si$ $[M+H]^+$ 480.3292, found 480.3295.

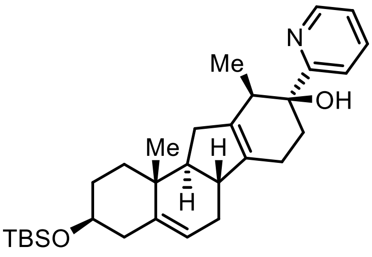

S18

TLC (petroleum ether / diethyl ether, 2:1 v/v): R_f = 0.40.

OR: $[\alpha]_D^{25}$ = +11.7 (c = 0.33, $CHCl_3$).

IR (neat) ν_{max}: 3407, 3018, 2917, 2851, 1473, 1257, 1214, 1089, 1008, 750 cm^{-1}.

^{1}H NMR (400 MHz, $CDCl_3$): δ 8.51 (d, *J* = 4.8 Hz, 1H), 7.66 (td, *J* = 8.0, 2.0 Hz, 1H), 7.25 – 7.15 (m, 2H), 5.63 (s, 1H), 5.44 – 5.29 (m, 1H), 3.63 – 3.38 (m, 1H), 2.36 – 2.18 (m, 5H), 1.99 – 1.86 (m, 3H), 1.80 – 1.68 (m, 4H), 1.65 – 1.45 (m, 5H), 1.31 – 1.29 (m, 3H), 0.98 (s, 3H), 0.90 (s, 9H), 0.07 (s, 6H).

^{13}C NMR (100 MHz, $CDCl_3$): δ 165.2, 147.0, 143.8, 142.5, 136.8, 126.2, 121.7, 121.3, 120.5, 75.0, 72.7, 52.4, 49.6, 42.4, 41.9, 41.7, 38.4, 36.7, 31.8, 31.2, 29.7, 29.7, 28.6, 25.9 (×3), 24.4, 18.7, 18.3, 13.9, –4.58 (×2).

HRMS (ESI): *m/z* calcd. for $C_{30}H_{46}NO_2Si$ $[M+H]^+$ 480.3292, found 480.3295.

3. X-ray Crystallographic Data

A single crystal of **15** was obtained by recrystallization from acetone. Crystallographic data for **15** have been deposited in the Cambridge Crystallographic Data Centre (CCDC 2321471). These data can be obtained free of charge via www.ccdc.cam.ac.uk.

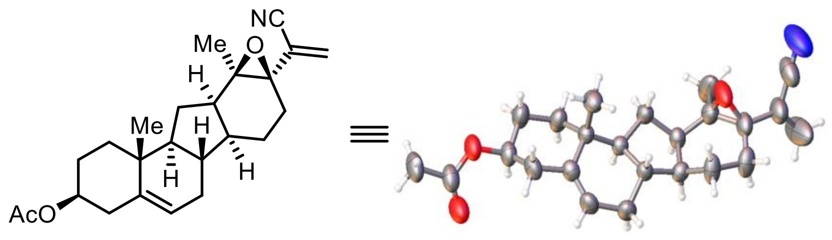

15 [X-ray]

Supplementary Table 6. Crystal data and structure refinement for compound 15.

CCDC	2321471
Empirical formula	$C_{24}H_{31}NO_3$
Formula weight	381.50
Temperature/K	293.15
Crystal system	monoclinic
Space group	$P2_1$
a/Å	12.1076(10)
b/Å	6.3719(5)
c/Å	14.5135(13)
α/°	90
β/°	103.836(9)
γ/°	90
Volume/Å^3	1087.20(16)
Z	2
ρ_{calc}g/cm^3	1.165
μ/mm^{-1}	0.076
F(000)	412.0
Crystal size/mm^3	0.35 × 0.3 × 0.25
Radiation	MoKα (λ = 0.71073)
2Θ range for data collection/°	6.842 to 52.738
Index ranges	$-14 \leq h \leq 15, -7 \leq k \leq 7, -18 \leq l \leq 17$
Reflections collected	5075

Independent reflections	3735 [R_{int} = 0.0192, R_{sigma} = 0.0558]
Data/restraints/parameters	3735/2/264
Goodness-of-fit on F^2	1.036
Final R indexes [I>=2σ (I)]	R_1 = 0.0590, wR_2 = 0.1145
Final R indexes [all data]	R_1 = 0.0984, wR_2 = 0.1383
Largest diff. peak/hole / e Å^{-3}	0.12/-0.16
Flack parameter	-0.4(10)

A single crystal of **20'** was obtained by recrystallization from petroleum ether / acetone (3:1). Crystallographic data for **20'** have been deposited in the Cambridge Crystallographic Data Centre (CCDC 2321472). These data can be obtained free of charge via www.ccdc.cam.ac.uk.

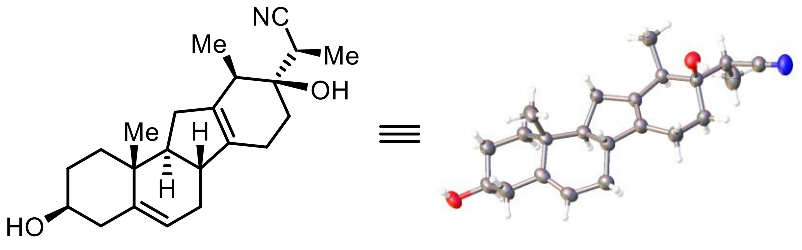

20' [X-ray]

Supplementary Table 7. Crystal data and structure refinement for compound 20'.

CCDC	2321472
Empirical formula	$C_{22}H_{31}NO_2$
Formula weight	341.48
Temperature/K	300.0
Crystal system	triclinic
Space group	P^1
a/Å	6.8393(3)
b/Å	11.6163(5)
c/Å	12.5216(5)
α/°	89.311(2)
β/°	81.056(2)
γ/°	76.822(2)
Volume/Å^3	956.55(7)
Z	2
ρ_{calc}g/cm^3	1.186

μ/mm^{-1}	0.581
F(000)	372.0
Crystal size/mm^3	0.38 × 0.23 × 0.21
Radiation	CuKα (λ = 1.54178)
2Θ range for data collection/°	7.148 to 134.142
Index ranges	-8 ≤ h ≤ 8, -13 ≤ k ≤ 13, -14 ≤ l ≤ 14
Reflections collected	26701
Independent reflections	6638 [R_{int} = 0.0538, R_{sigma} = 0.0452]
Data/restraints/parameters	6638/3/461
Goodness-of-fit on F^2	1.041
Final R indexes [I>=2σ (I)]	R_1 = 0.0394, wR_2 = 0.1025
Final R indexes [all data]	R_1 = 0.0399, wR_2 = 0.1029
Largest diff. peak/hole / e Å^{-3}	0.16/-0.24
Flack parameter	0.09(9)

A single crystal of **32d** was obtained by recrystallization from acetone. Crystallographic data for **32d** have been deposited in the Cambridge Crystallographic Data Centre (CCDC 2321473). These data can be obtained free of charge via www.ccdc.cam.ac.uk.

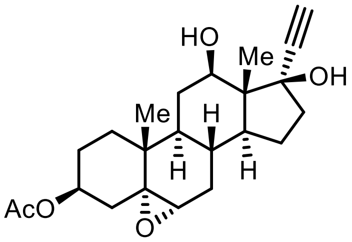

32d

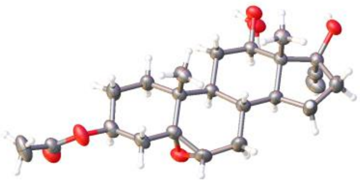

[X-ray]

Supplementary Table 8. Crystal data and structure refinement for compound 32d.

CCDC	2321473
Empirical formula	$C_{23}H_{33}O_{5.5}$
Formula weight	397.49
Temperature/K	282.0
Crystal system	orthorhombic
Space group	$P2_12_12$
a/Å	9.5203(10)
b/Å	37.228(4)

c/Å	6.4068(6)
α/°	90
β/°	90
γ/°	90
Volume/$Å^3$	2270.7(4)
Z	4
ρcalcg/cm^3	1.163
μ/mm^{-1}	0.663
F(000)	860.0
Crystal size/mm^3	0.42 × 0.34 × 0.08
Radiation	CuKα (λ = 1.54178)
2Θ range for data collection/°	4.748 to 140.372
Index ranges	$-11 \leq h \leq 10$, $-42 \leq k \leq 41$, $-7 \leq l \leq 7$
Reflections collected	20133
Independent reflections	4169 [R_{int} = 0.0705, R_{sigma} = 0.0433]
Data/restraints/parameters	4169/0/269
Goodness-of-fit on F^2	1.075
Final R indexes [I>=2σ (I)]	R_1 = 0.0472, wR_2 = 0.1352
Final R indexes [all data]	R_1 = 0.0525, wR_2 = 0.1388
Largest diff. peak/hole / e $Å^{-3}$	0.21/-0.25
Flack parameter	0.06(10)

4. NMR Spectra

^{1}H NMR of **18** (400 MHz, $CDCl_3$)

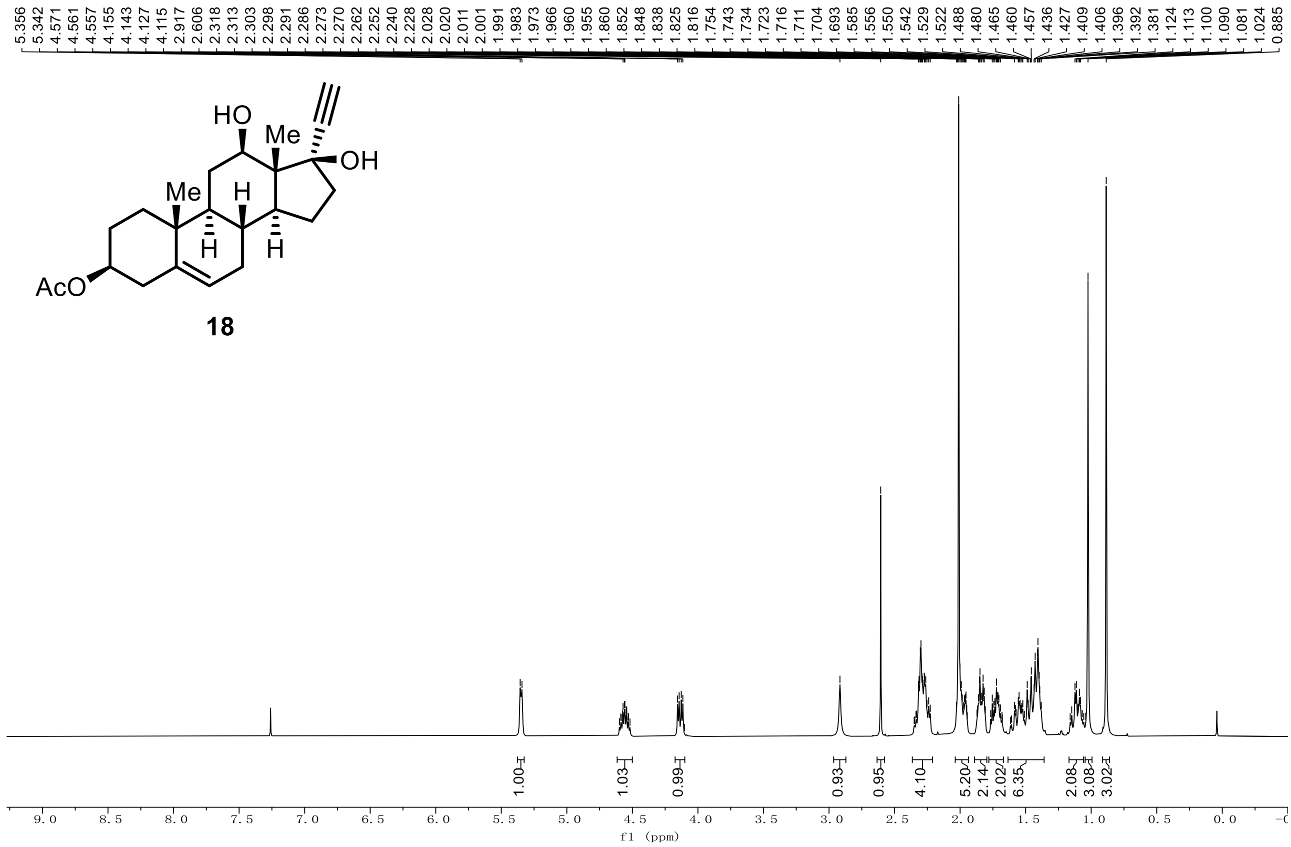

^{13}C NMR of **18** (100 MHz, $CDCl_3$)

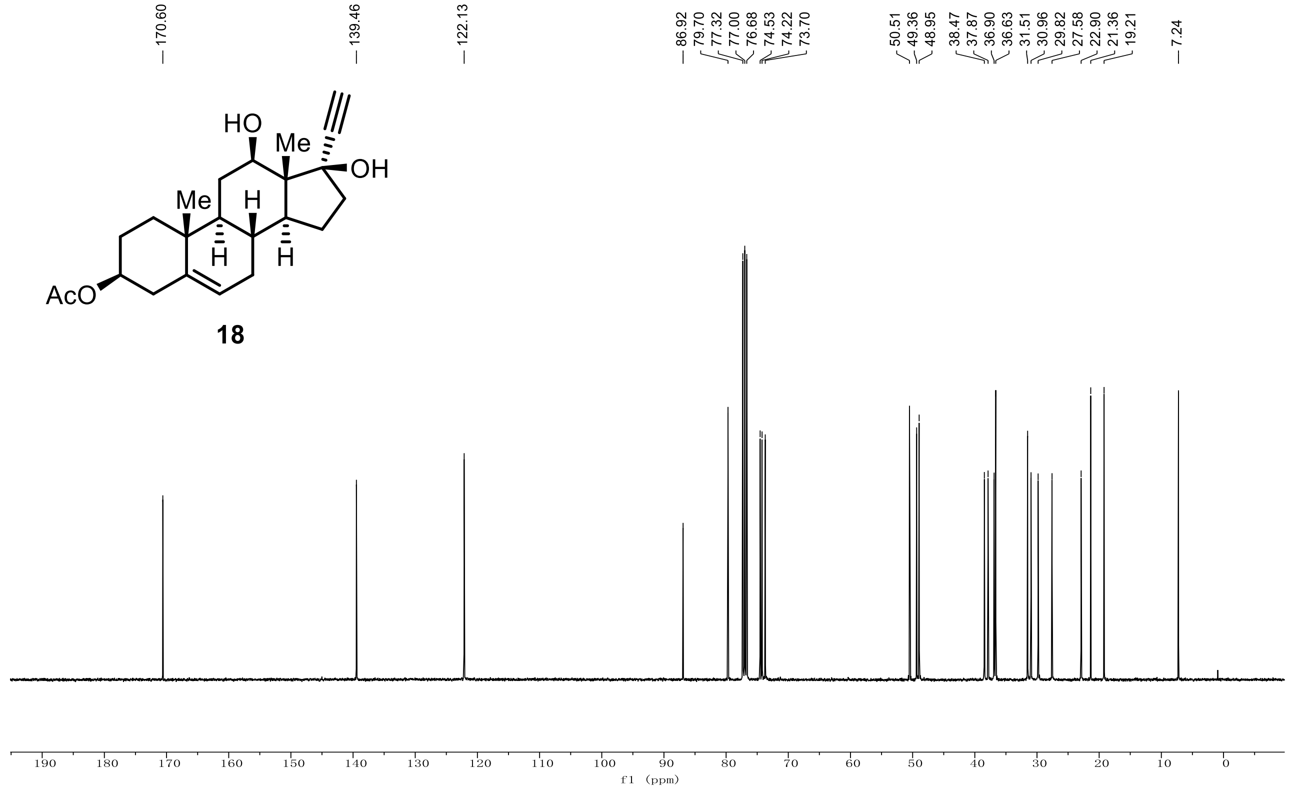

^{1}H NMR of **16** (400 MHz, $CDCl_3$)

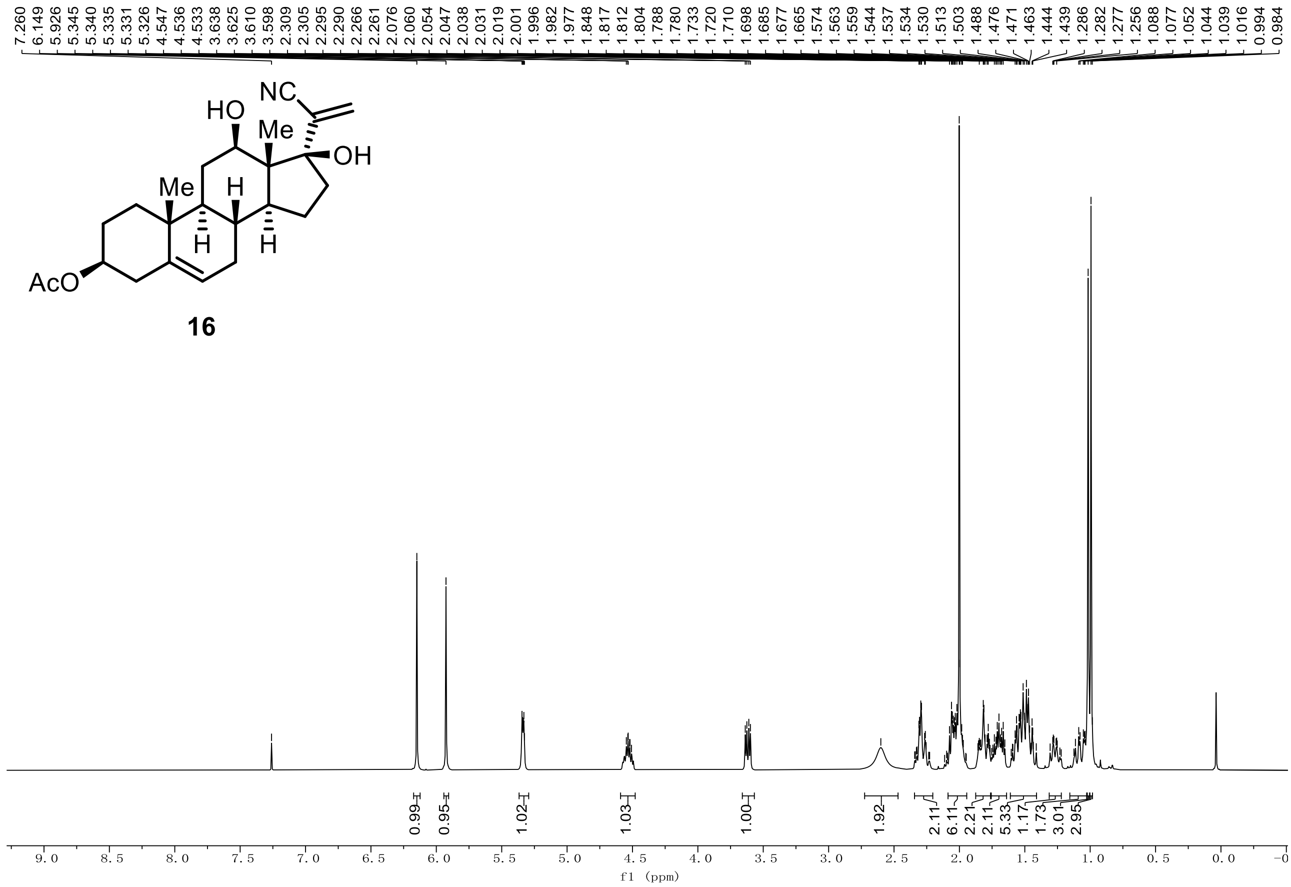

^{13}C NMR of **16** (100 MHz, $CDCl_3$)

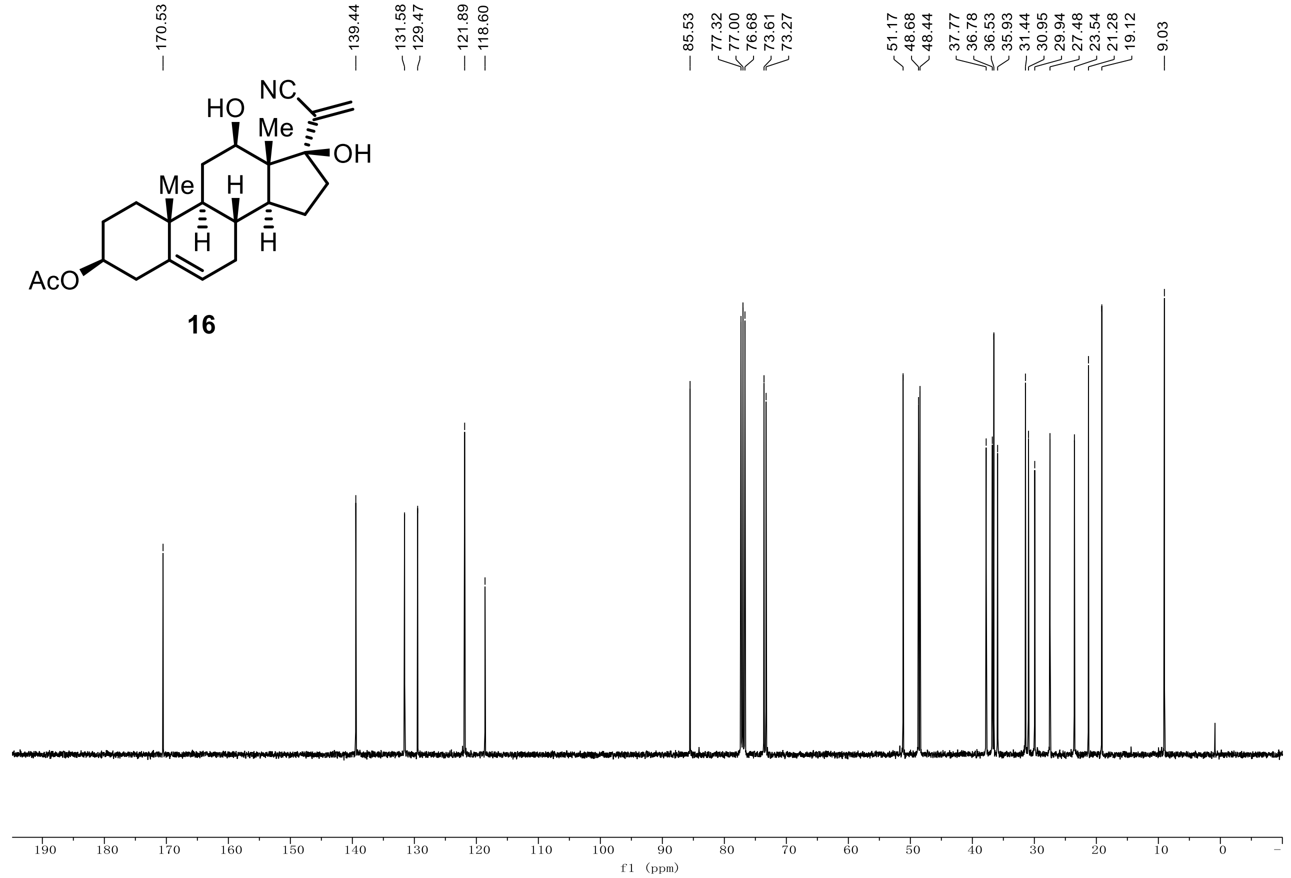

^{1}H NMR of **15** (400 MHz, $CDCl_3$)

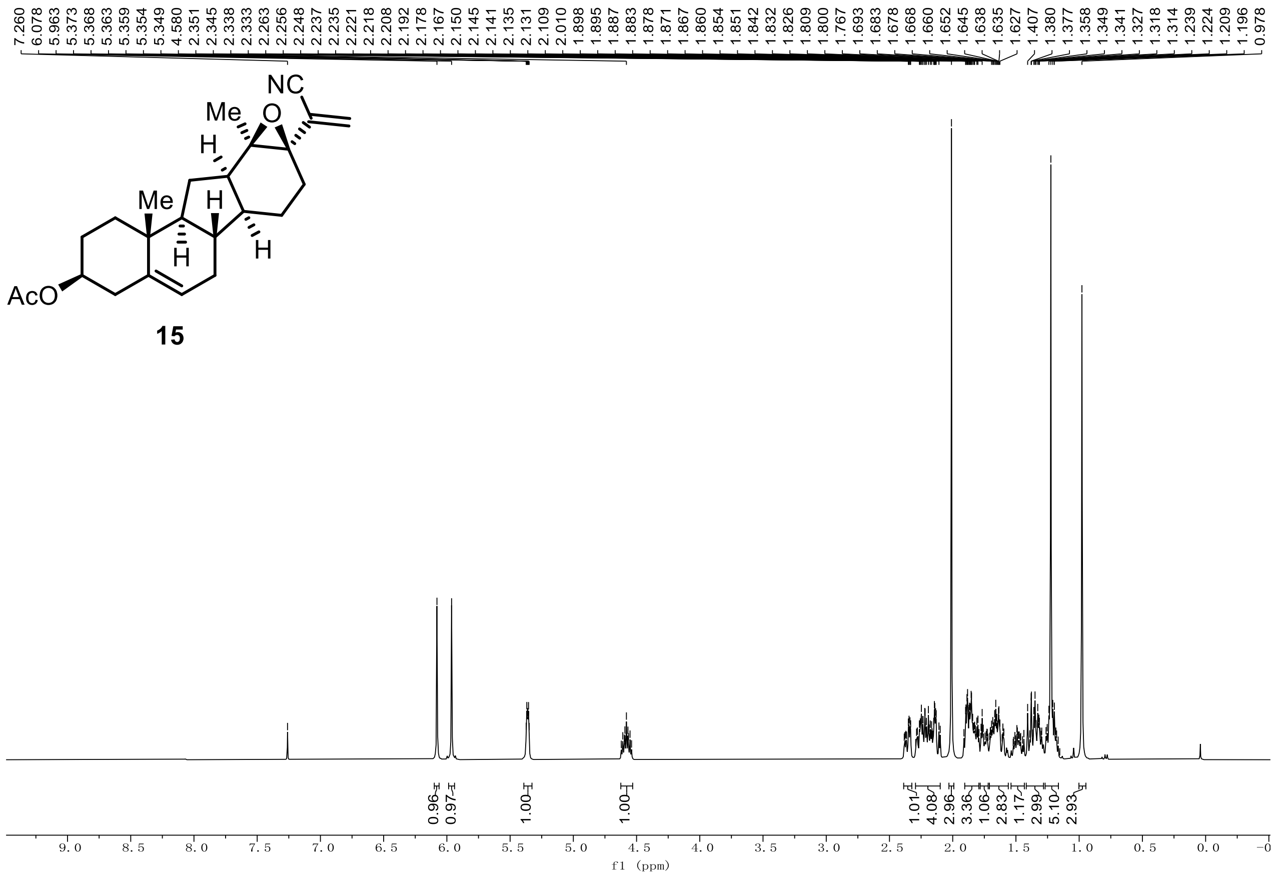

^{13}C NMR of **15** (100 MHz, $CDCl_3$)

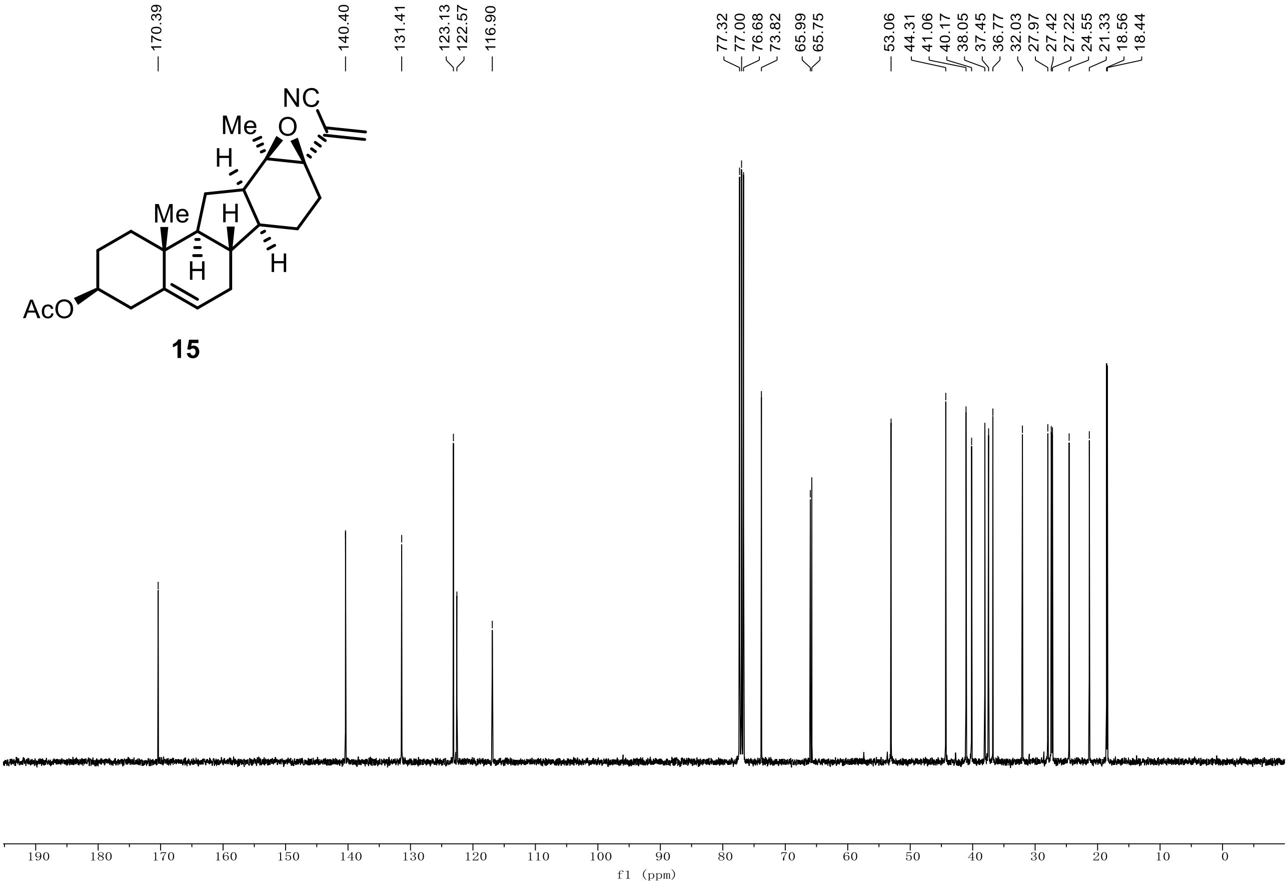

^{1}H NMR of **19** (400 MHz, $CDCl_3$)

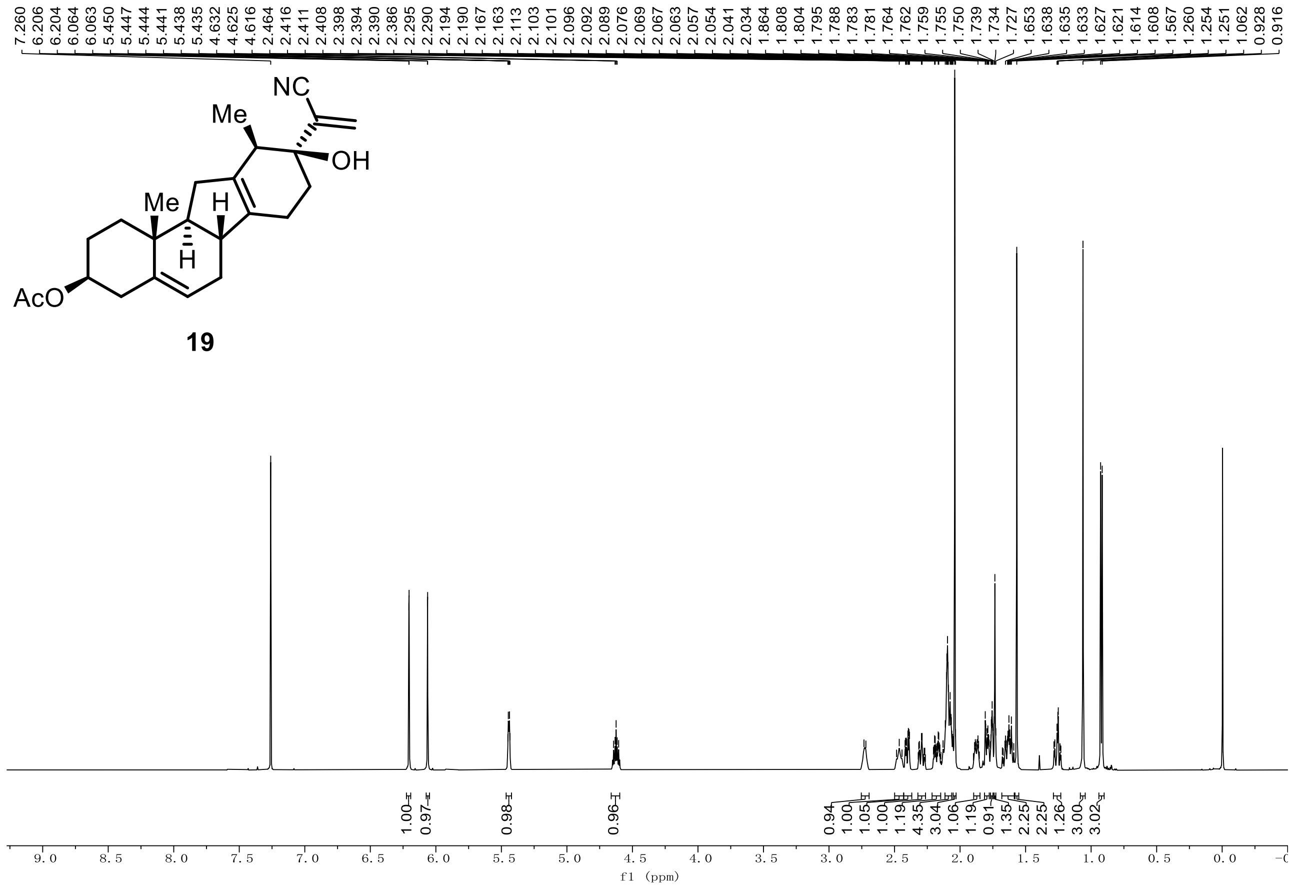

^{13}C NMR of **19** (100 MHz, $CDCl_3$)

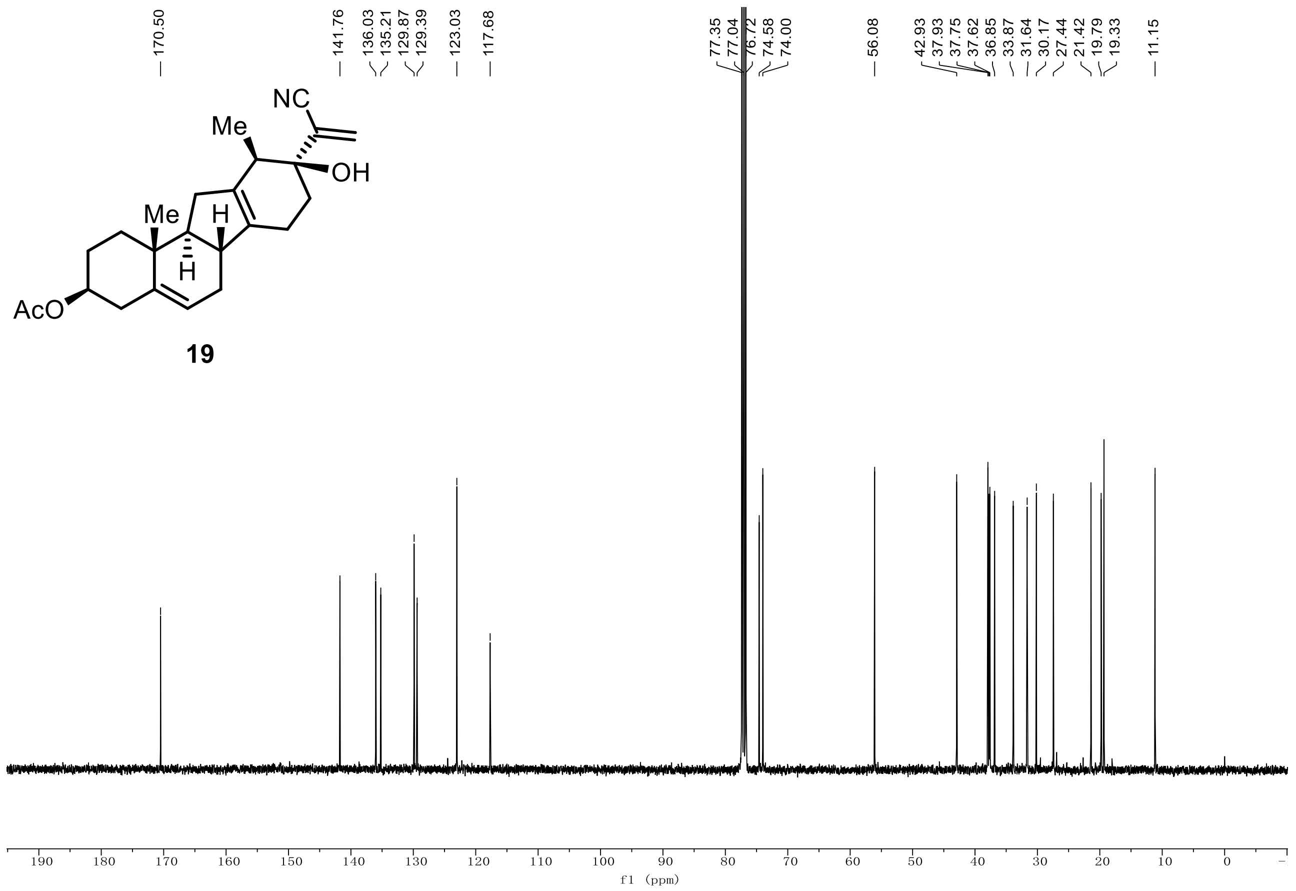

^{1}H NMR of **20** (400 MHz, $CDCl_3$)

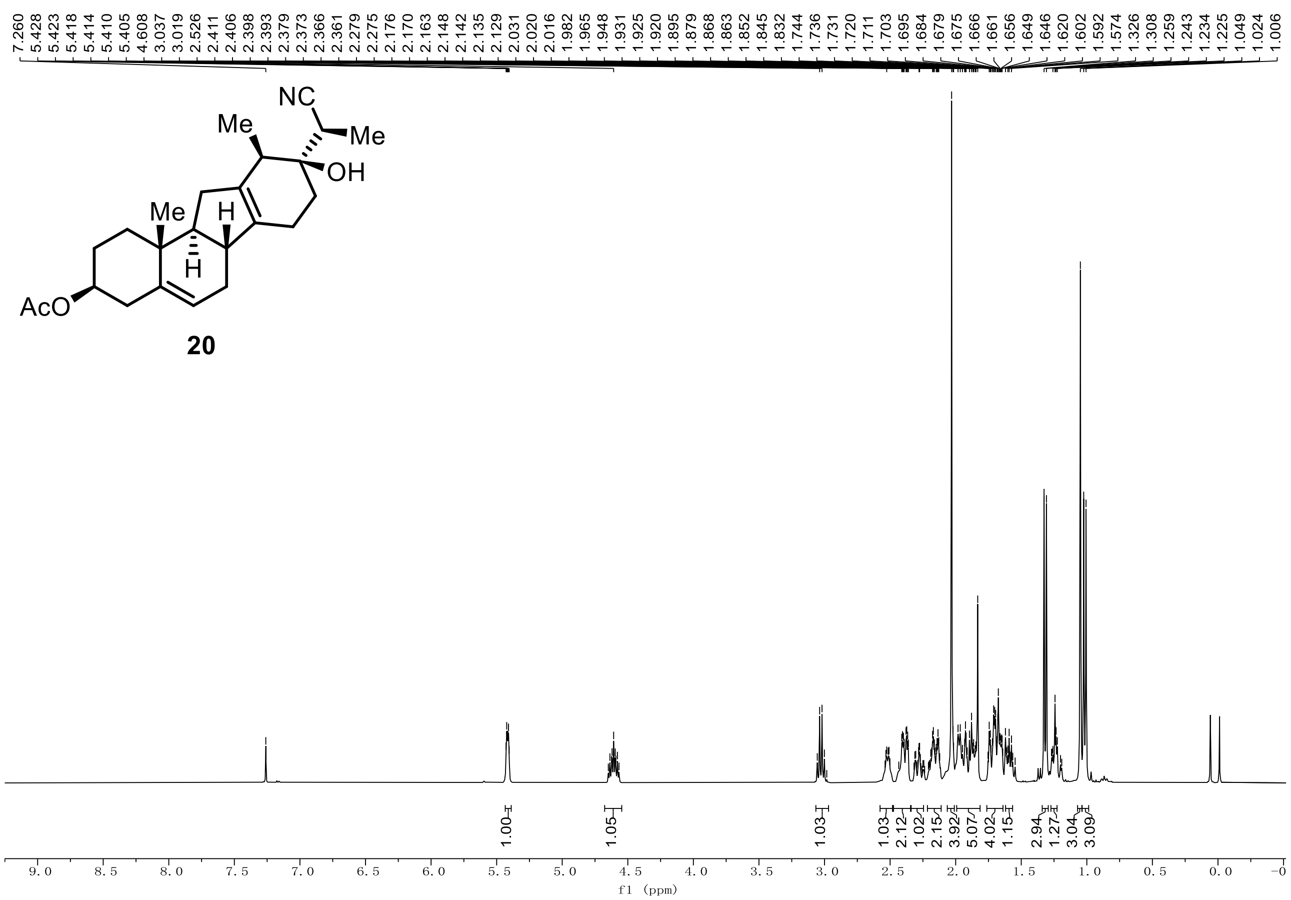

^{13}C NMR of **20** (100 MHz, $CDCl_3$)

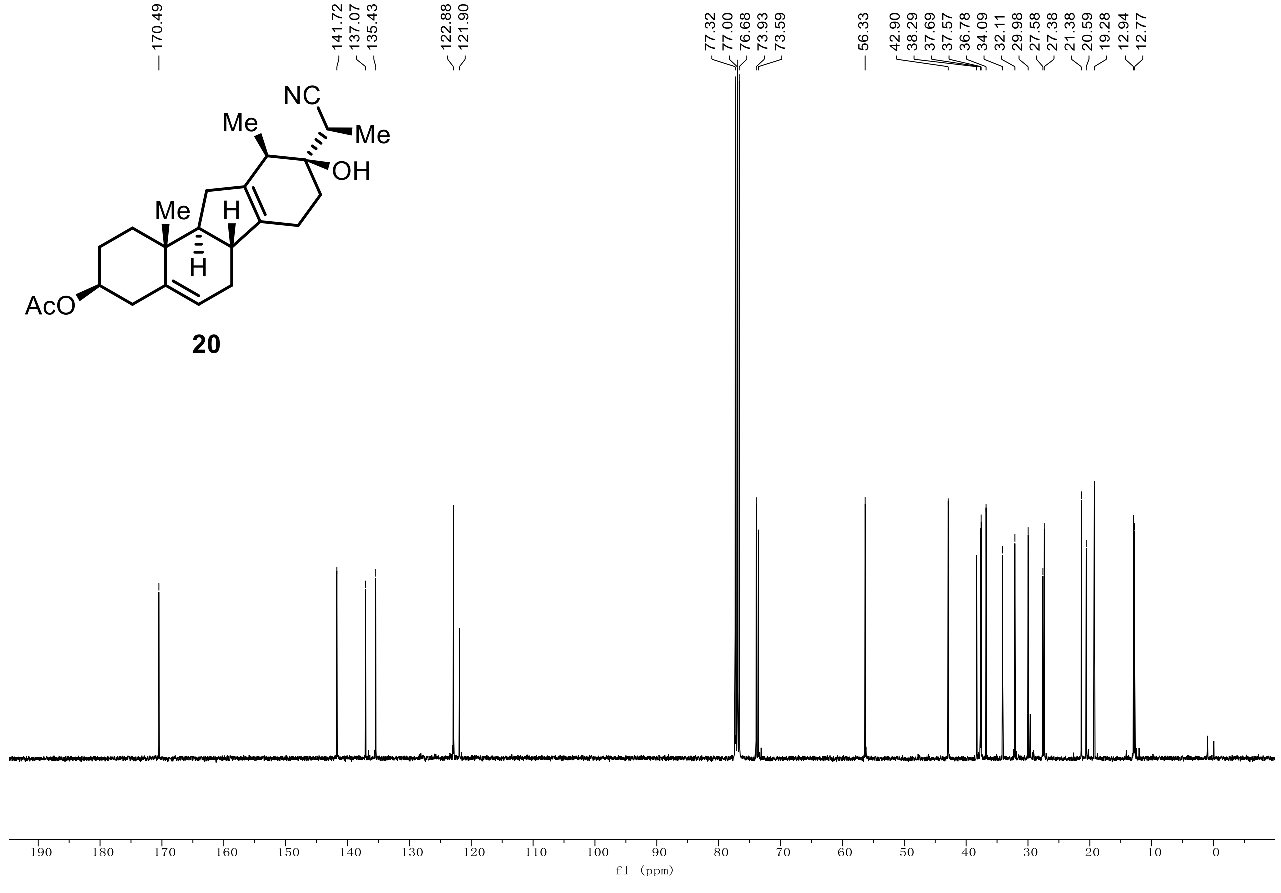

^{1}H NMR of **20'** (400 MHz, $CDCl_3$)

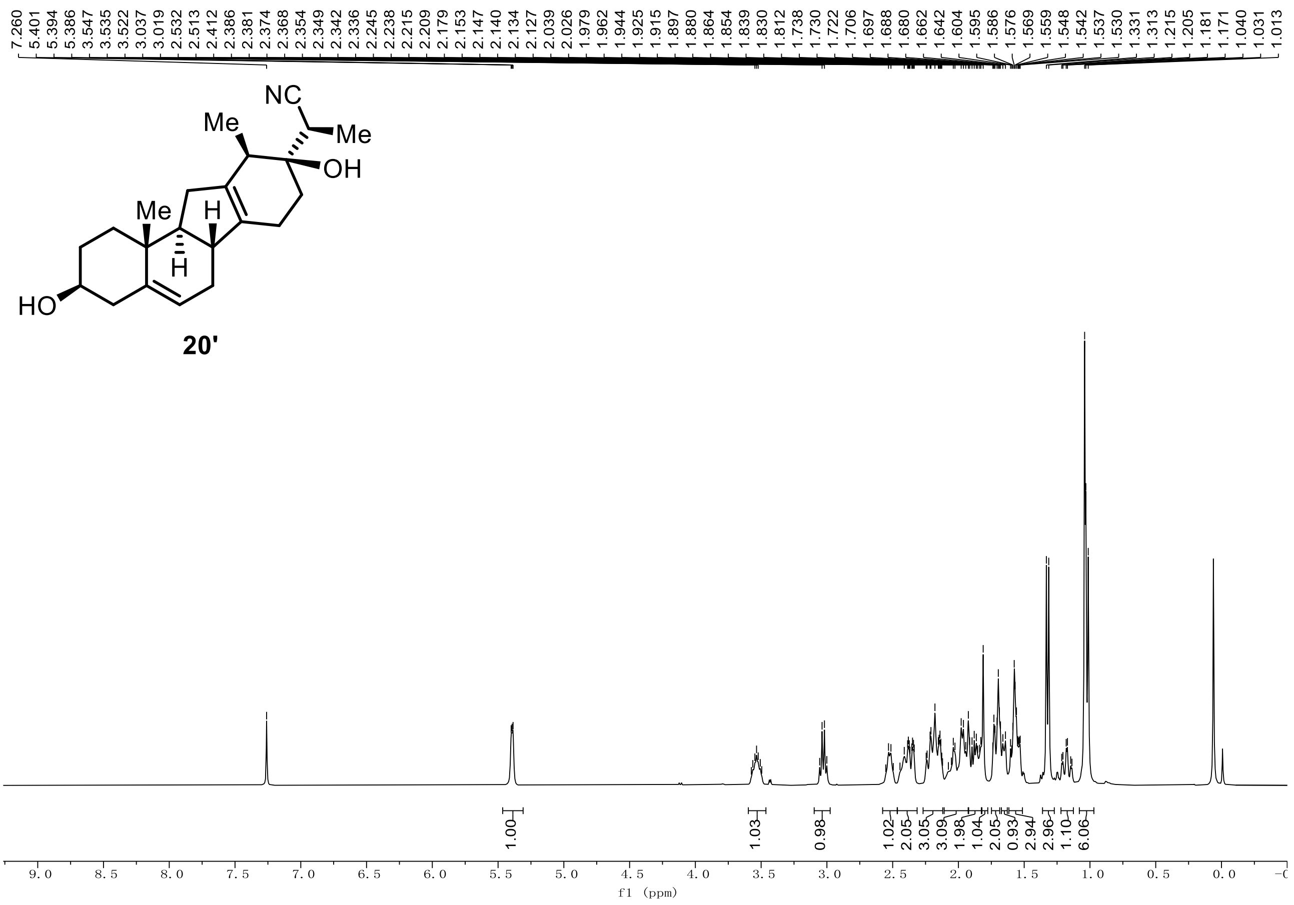

^{13}C NMR of **20'** (100 MHz, $CDCl_3$)

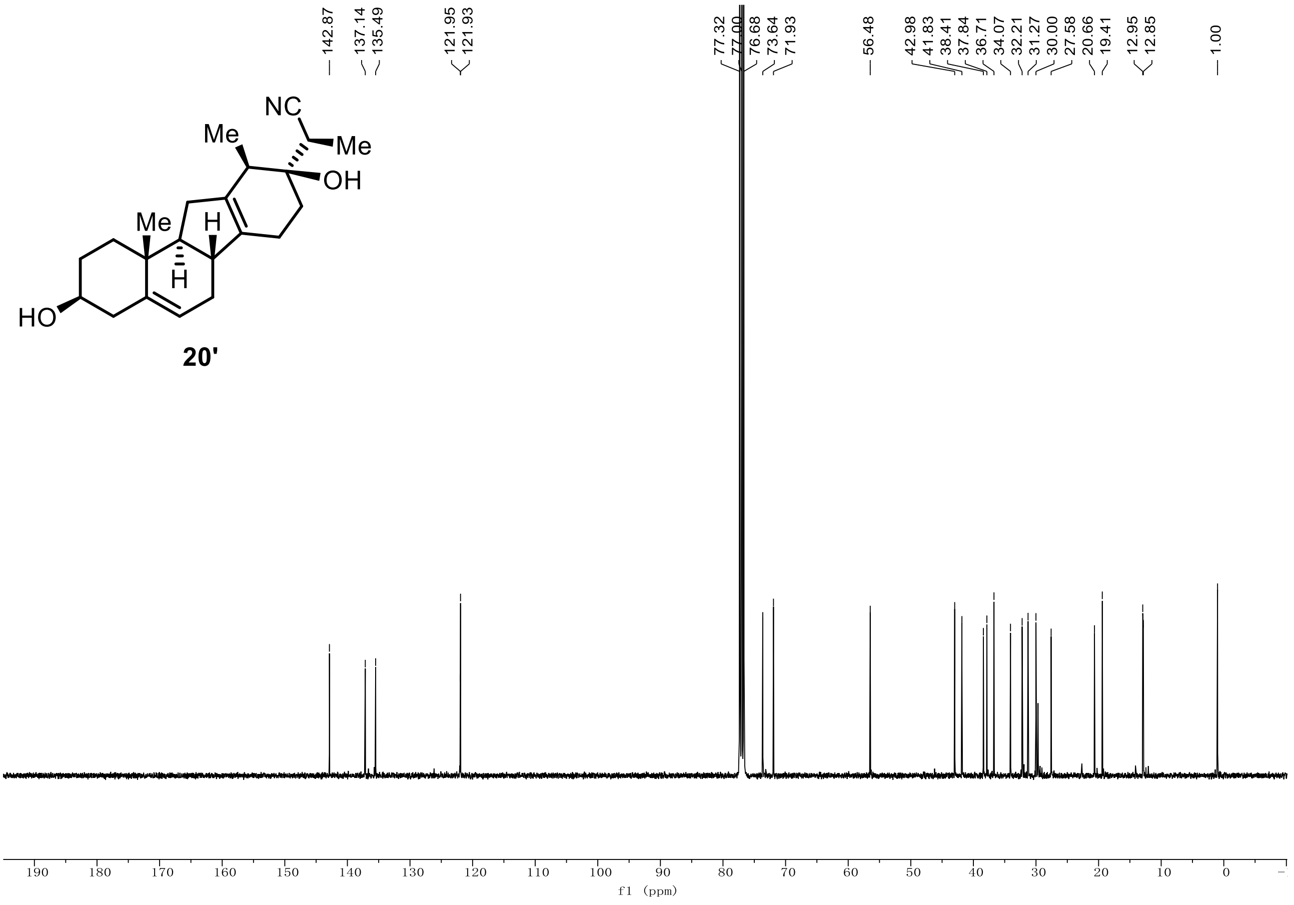

^{1}H NMR of **21** (400 MHz, $CDCl_3$)

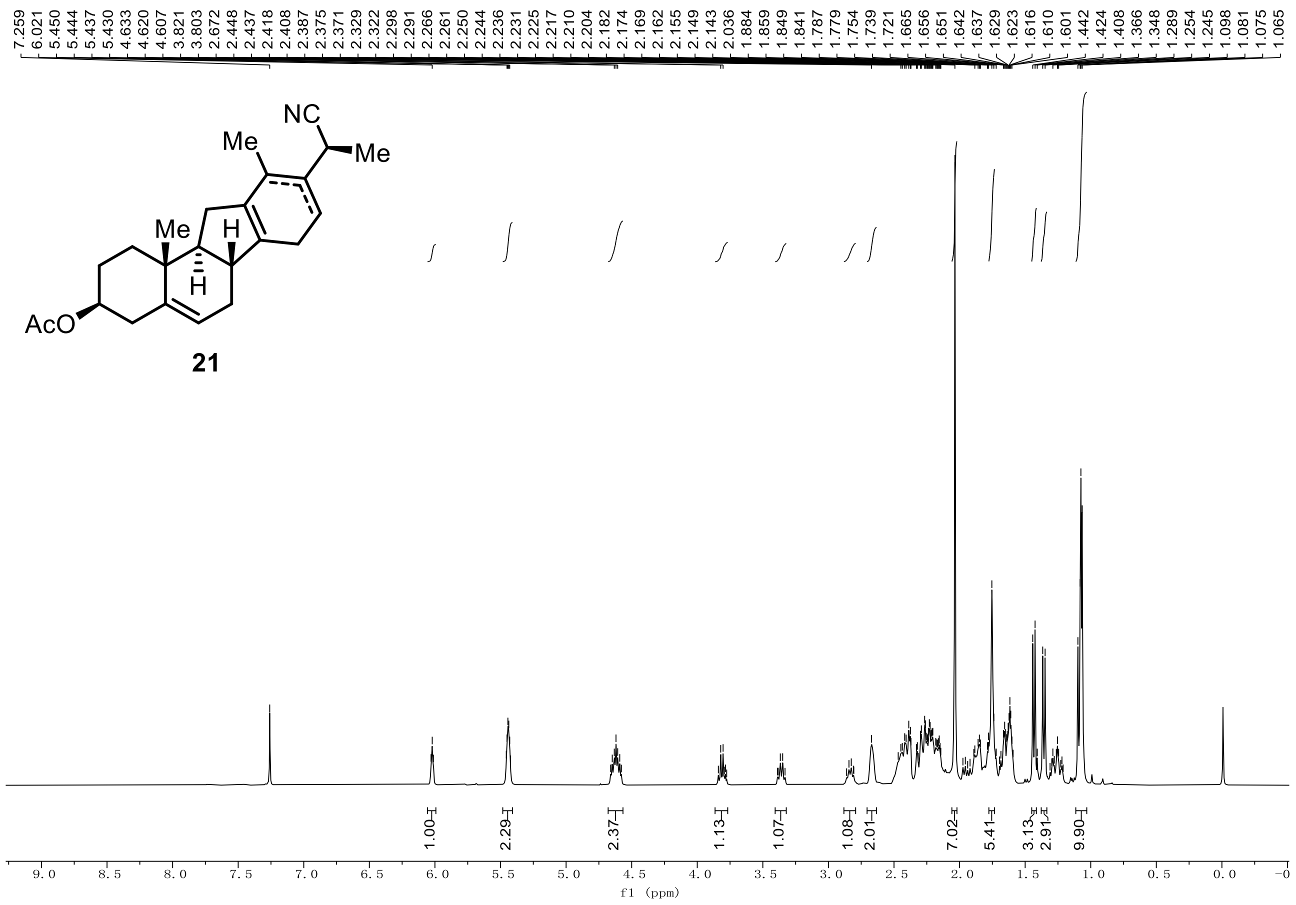

^{13}C NMR of **21** (100 MHz, $CDCl_3$)

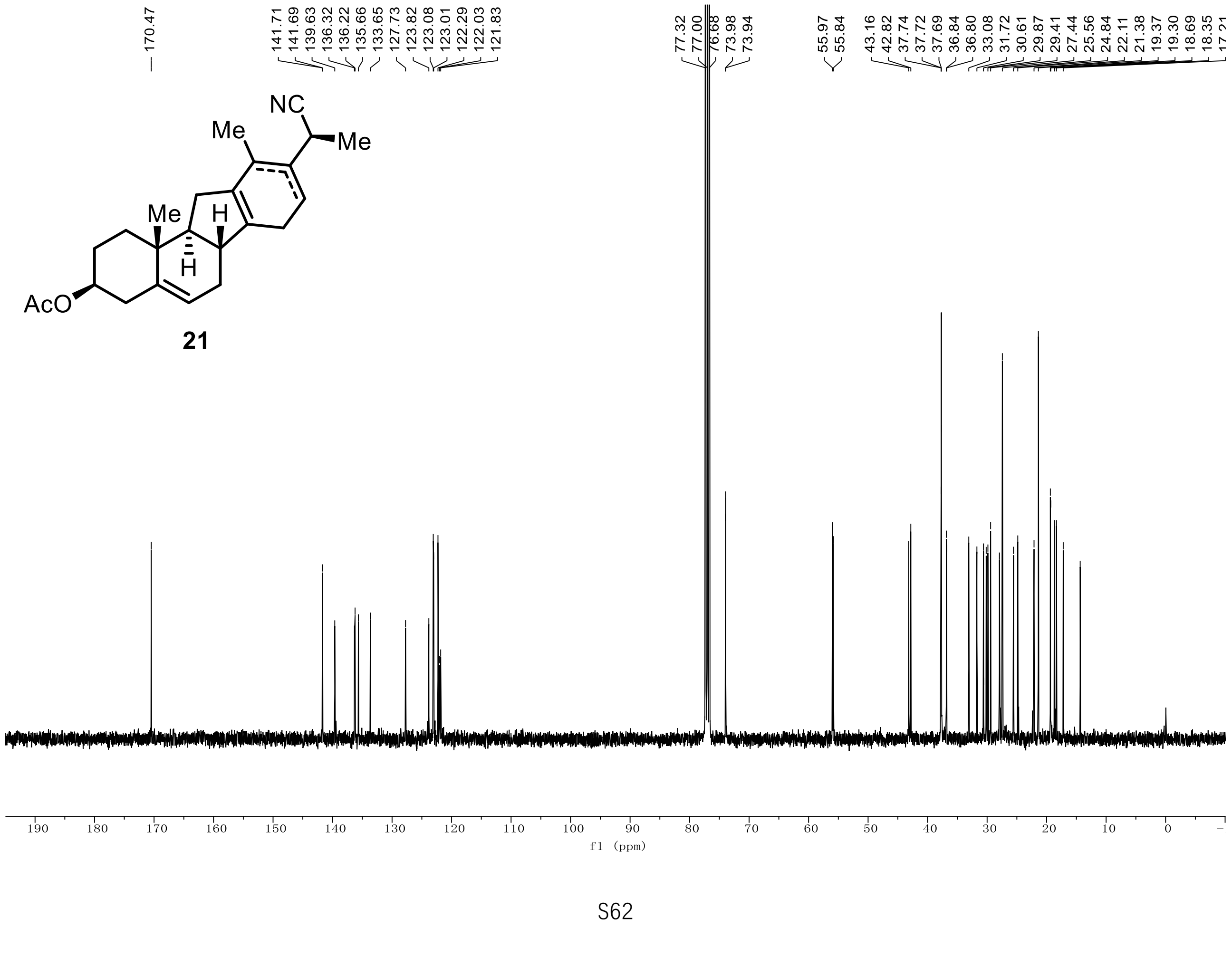

^{1}H NMR of **22** (400 MHz, CDCl$_3$)

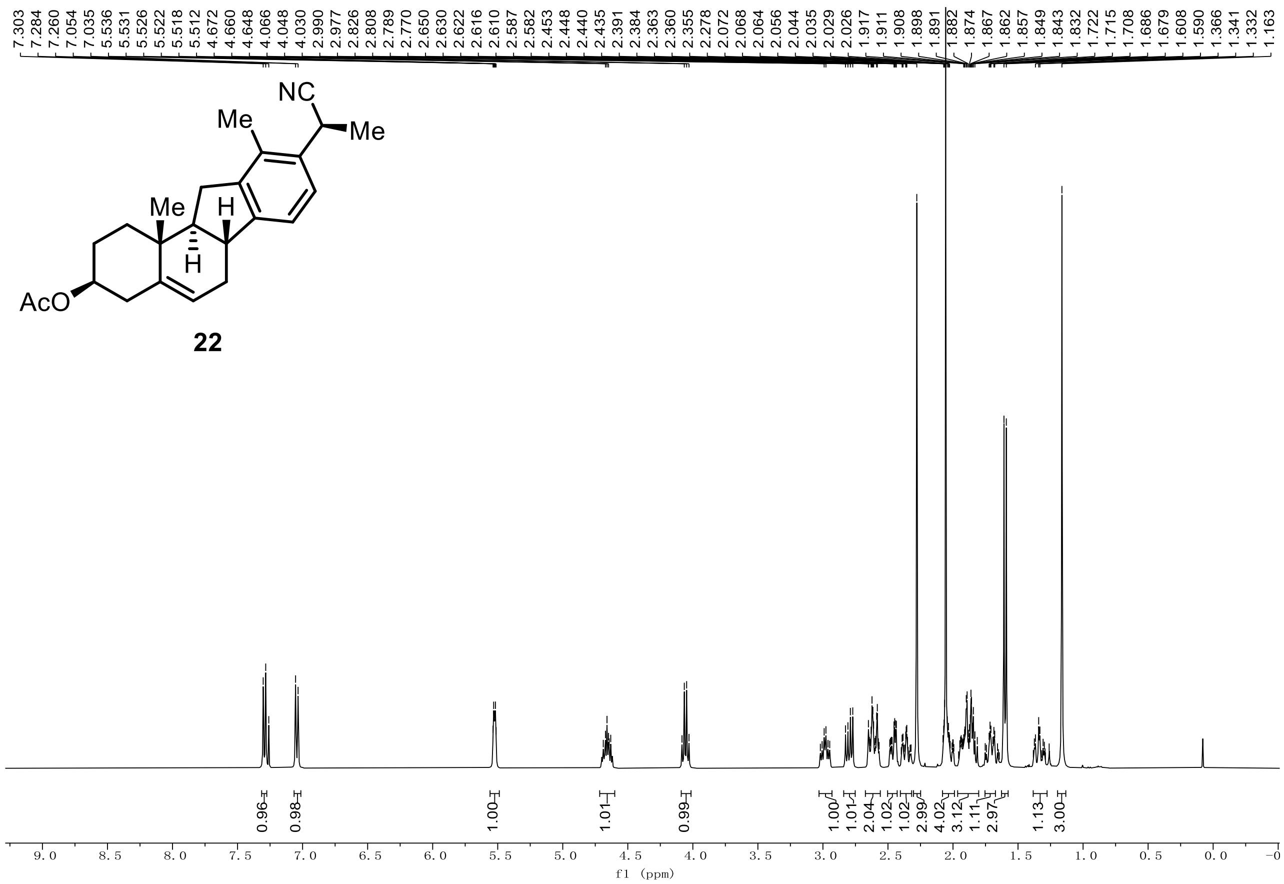

^{13}C NMR of **22** (100 MHz, CDCl$_3$)

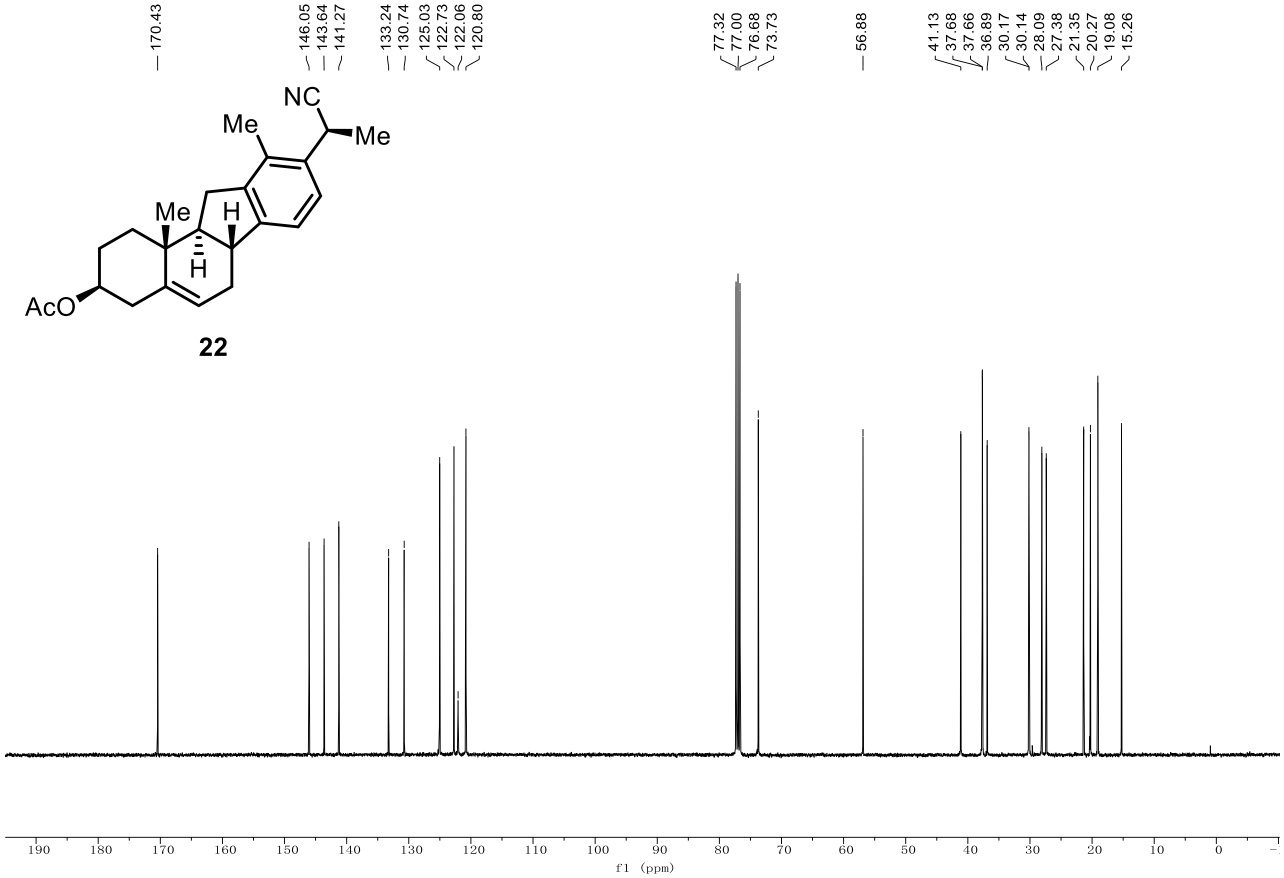

^{1}H NMR of **23** (400 MHz, CD_3CN)

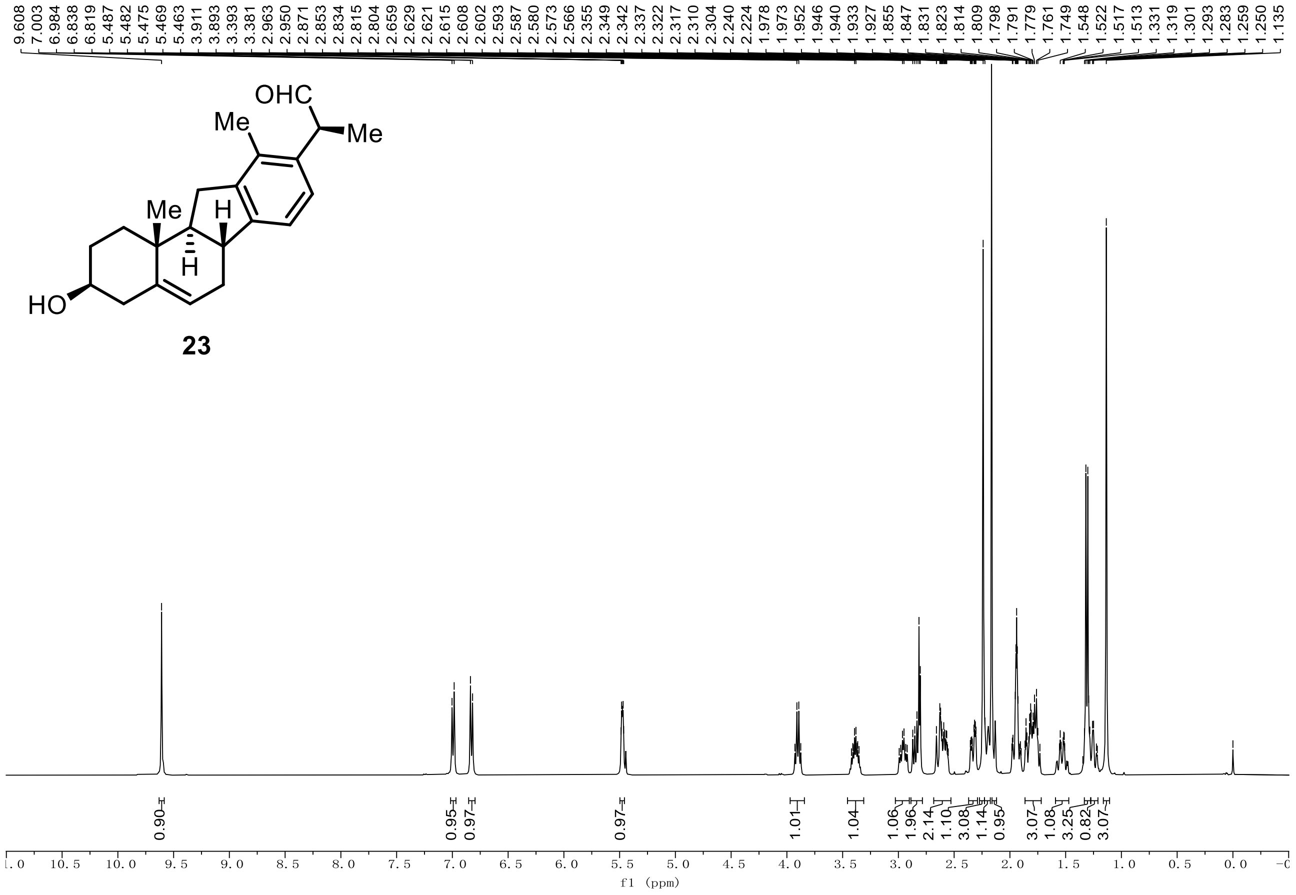

^{13}C NMR of **23** (100 MHz, CD_3CN)

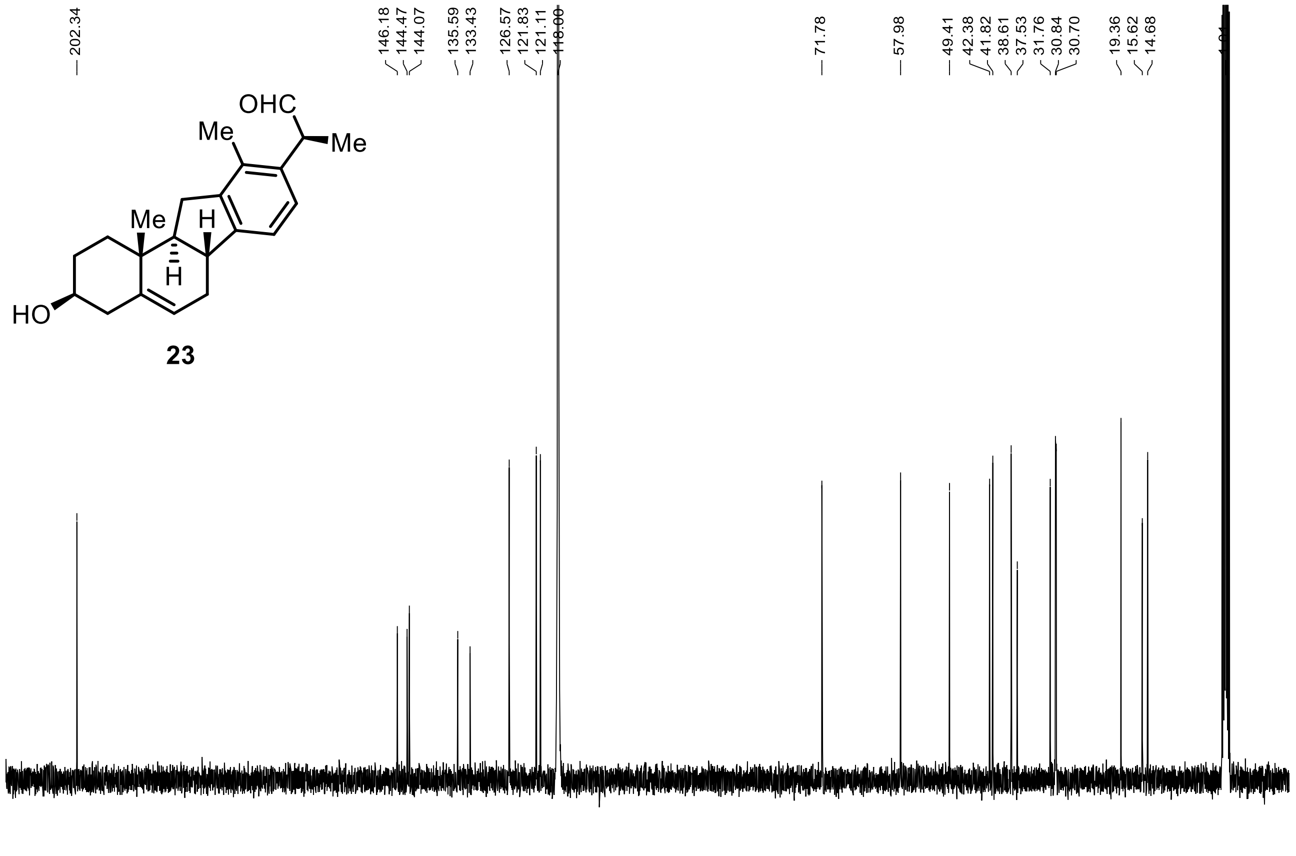

^{1}H NMR of **12** (400 MHz, $CDCl_3$)

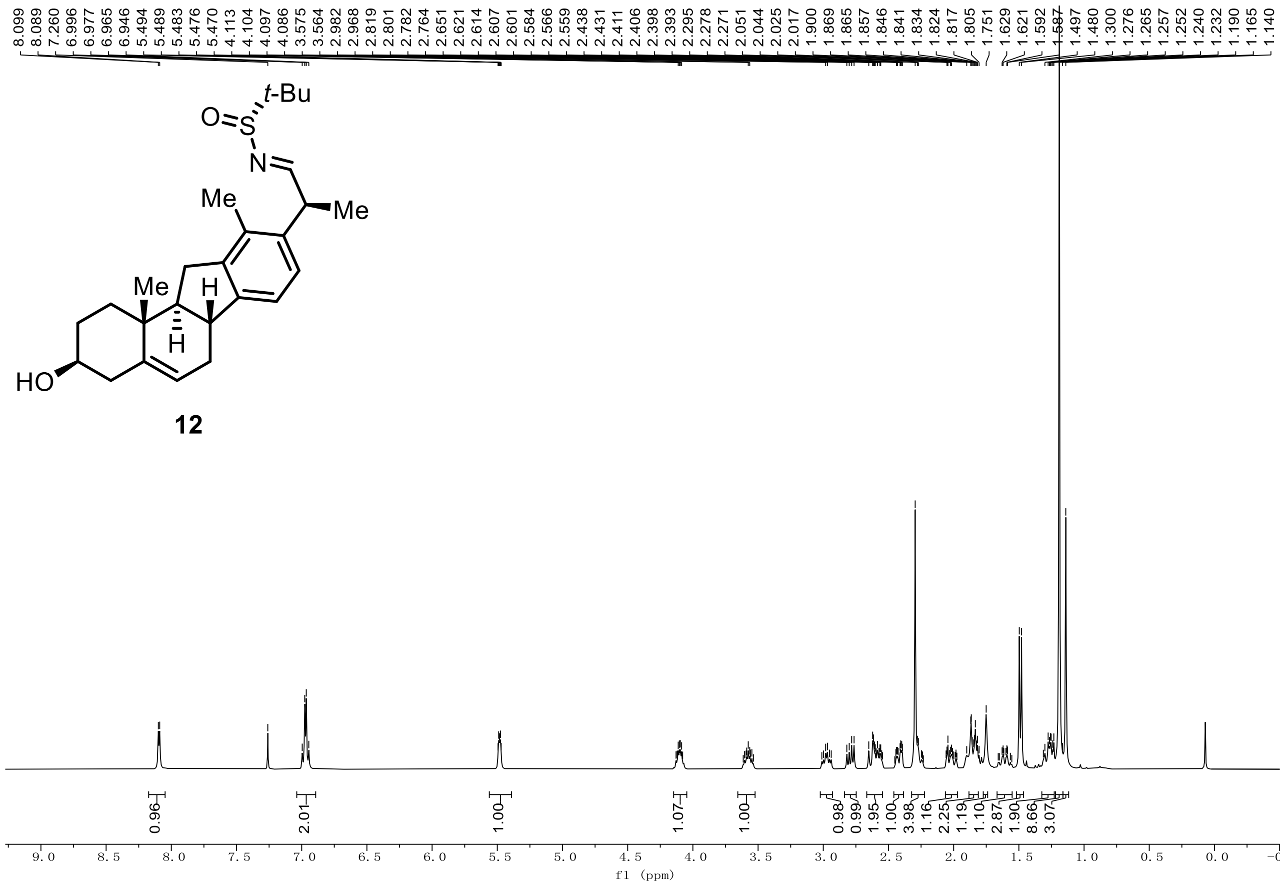

^{13}C NMR of **12** (100 MHz, $CDCl_3$)

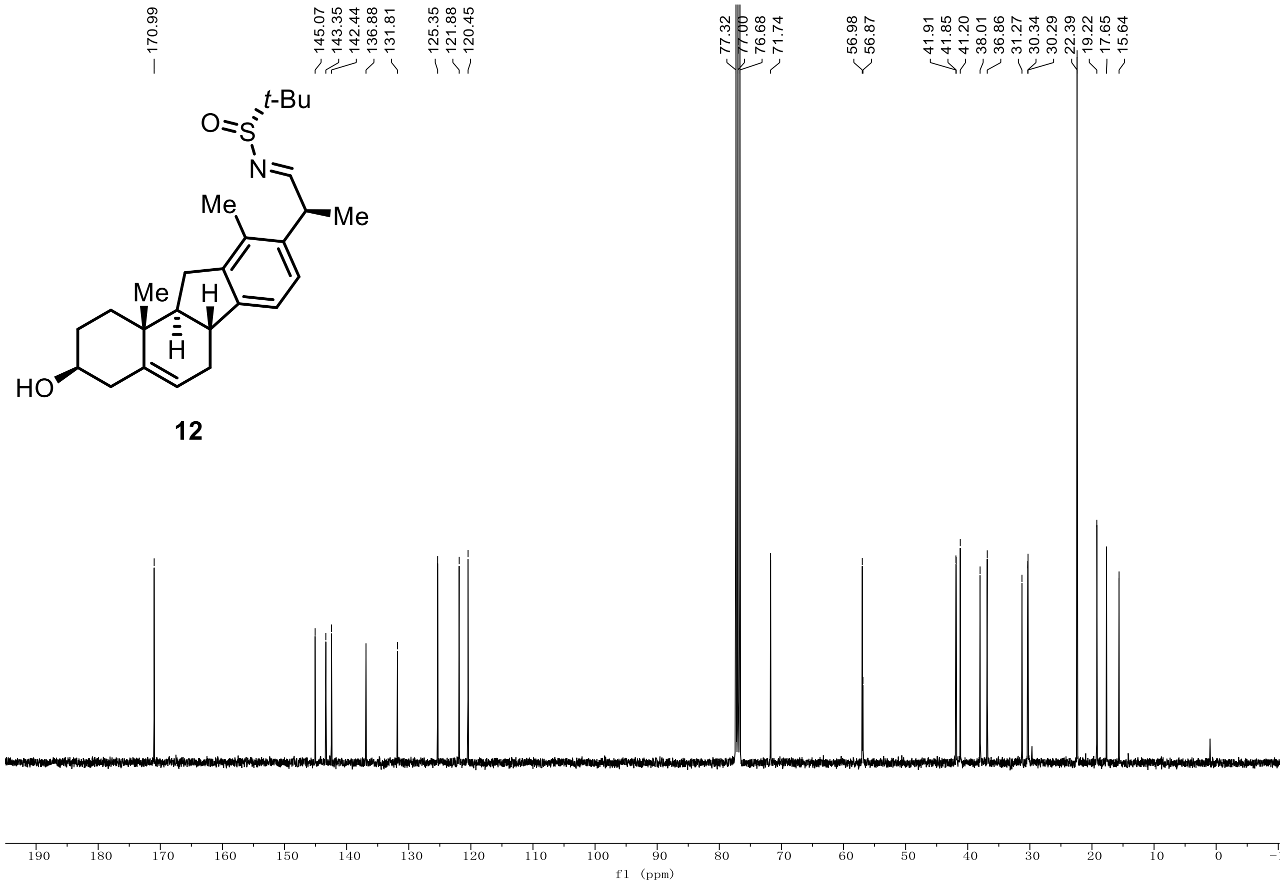

^{1}H NMR of **14** (400 MHz, $CDCl_3$)

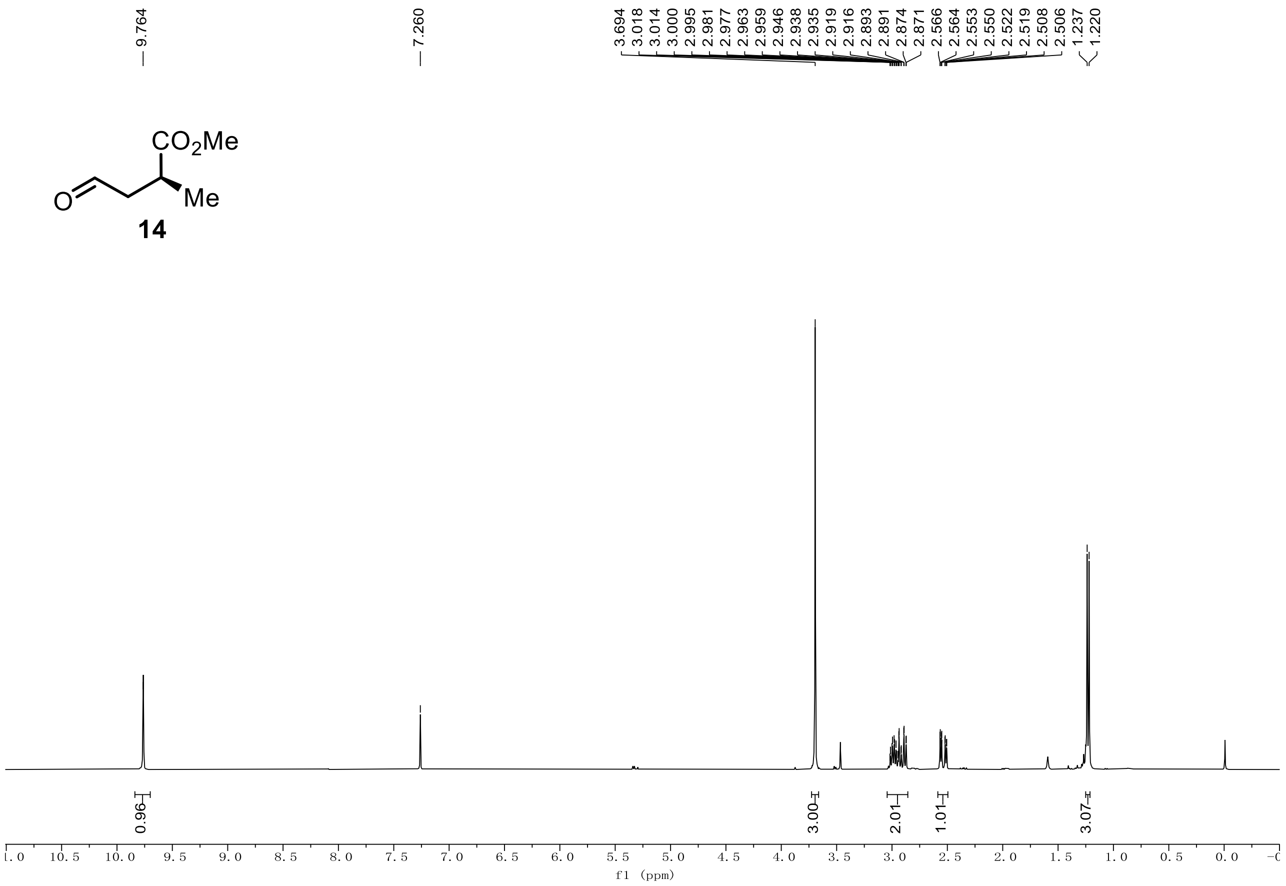

^{13}C NMR of **14** (100 MHz, $CDCl_3$)

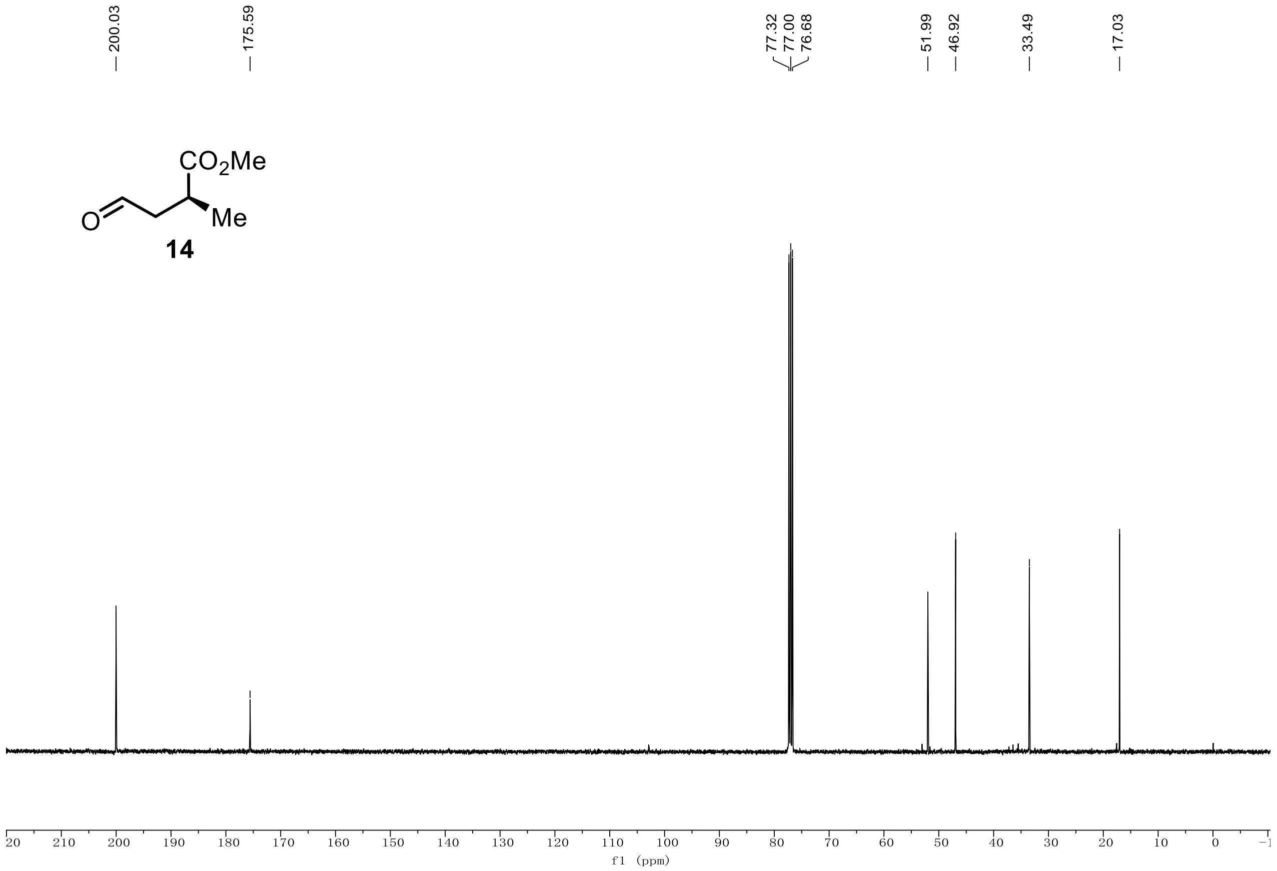

^{1}H NMR of **28** (400 MHz, $CDCl_3$)

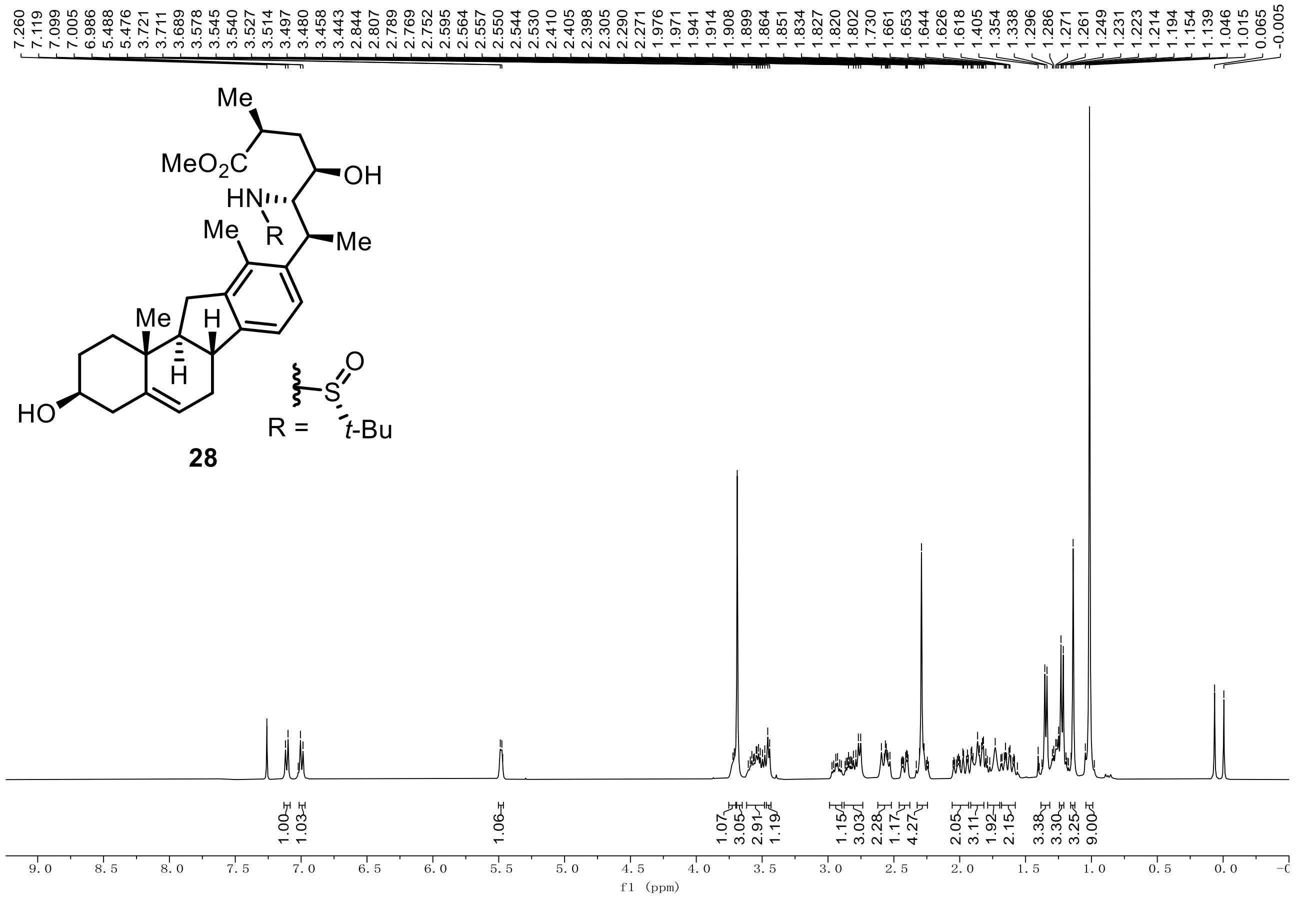

^{13}C NMR of **28** (100 MHz, $CDCl_3$)

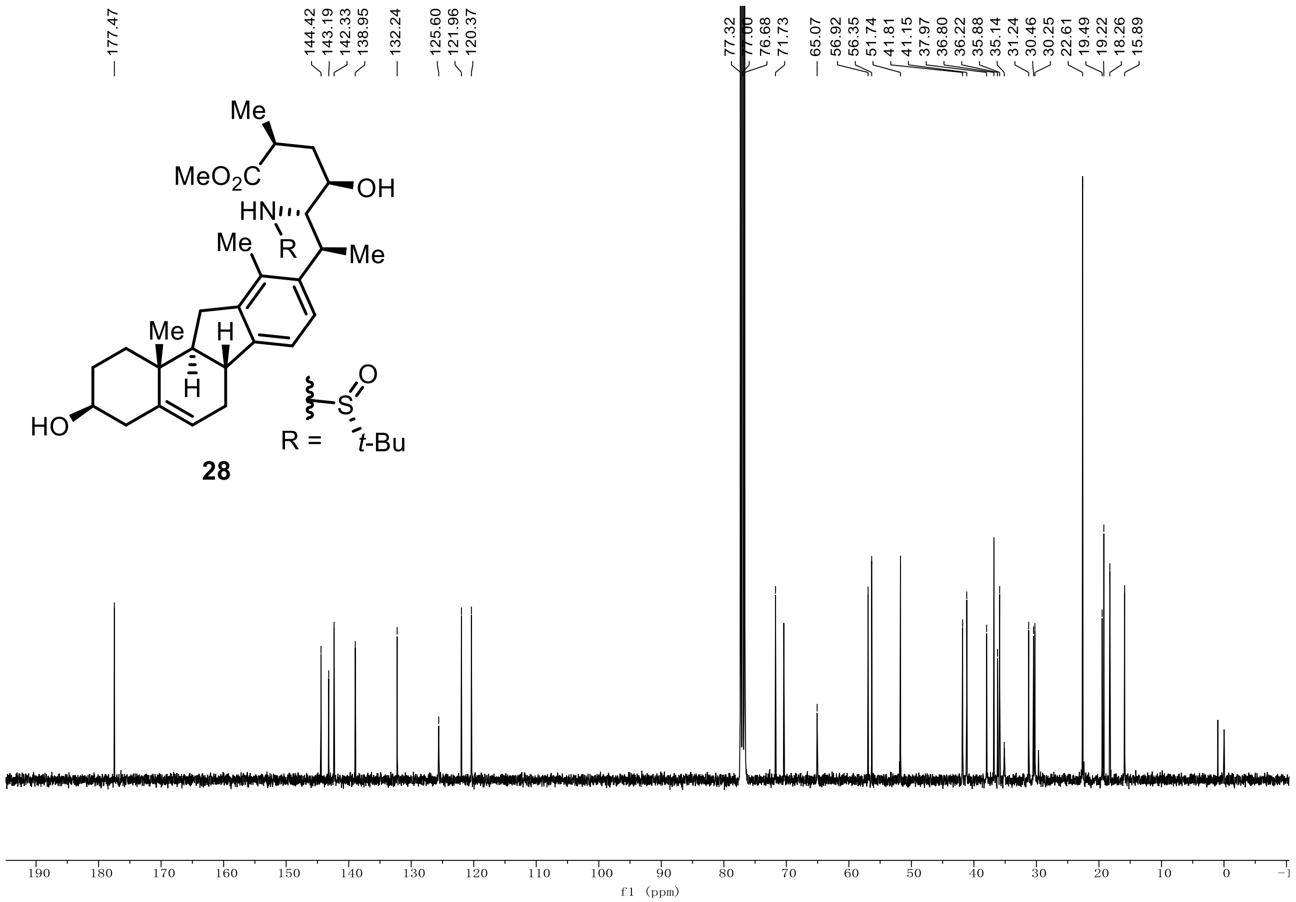

^{1}H NMR of **29** (400 MHz, CD_3OD)

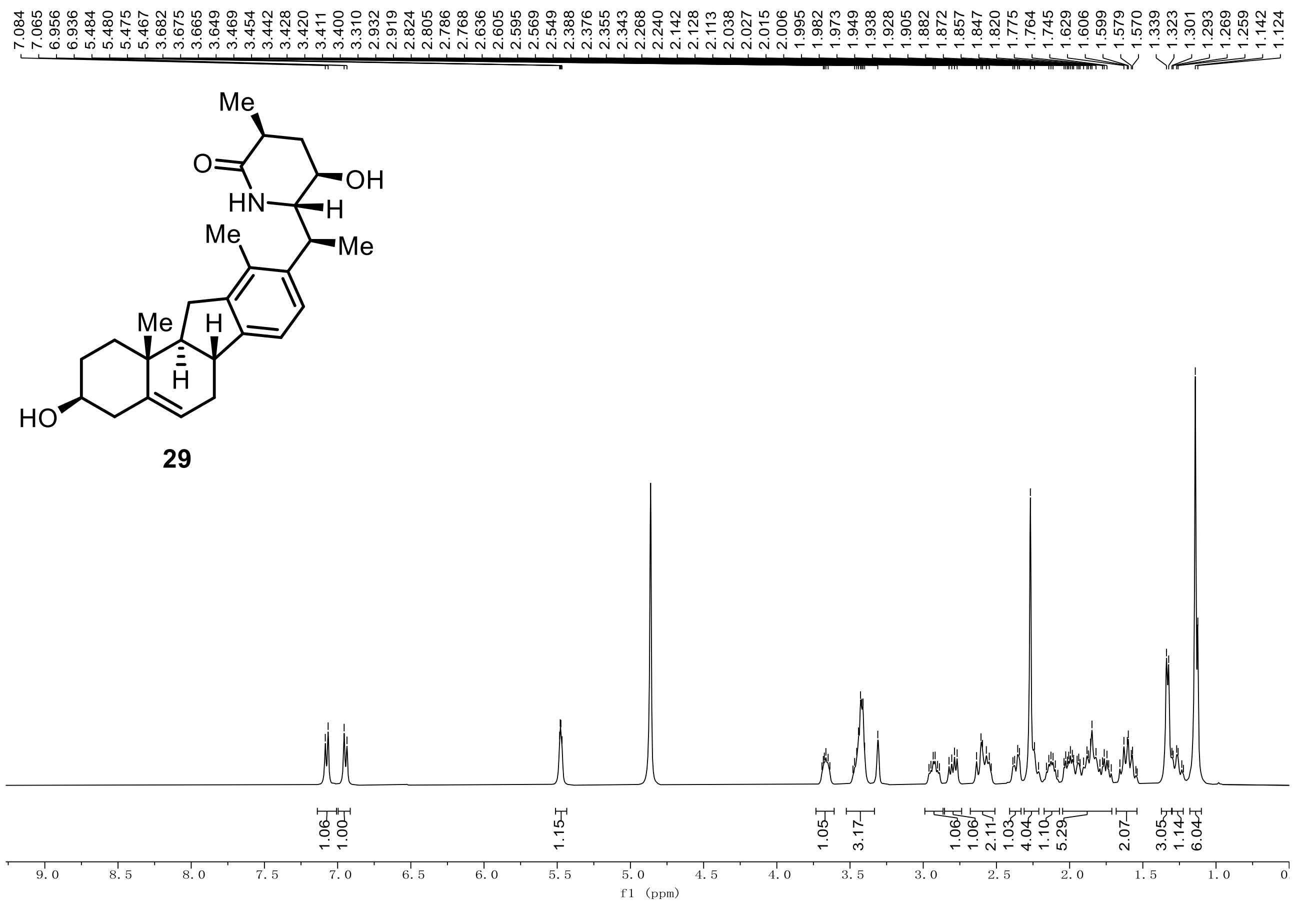

^{13}C NMR of **29** (100 MHz, CD_3OD)

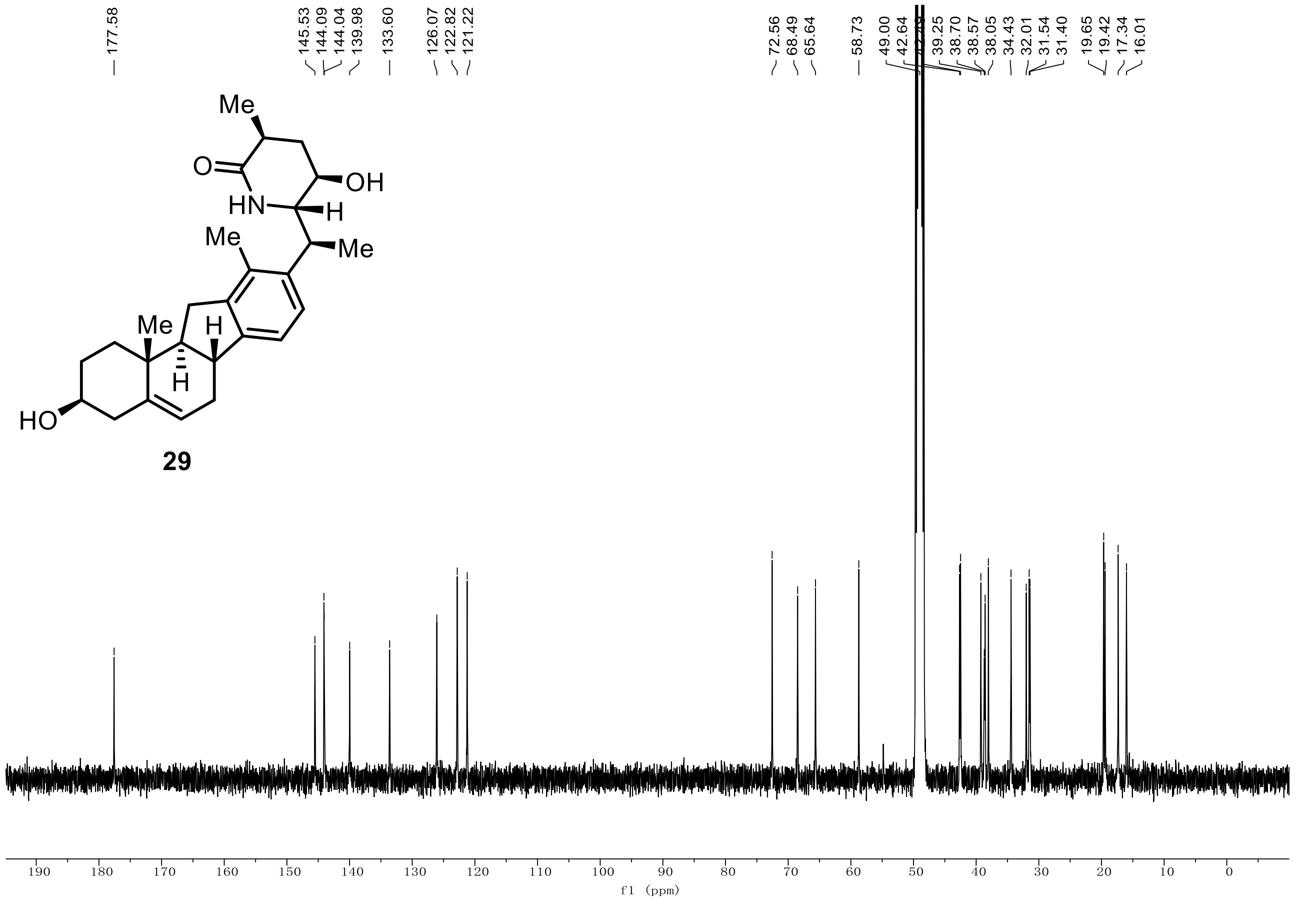

^{1}H NMR of **1** (400 MHz, C_5D_5N)

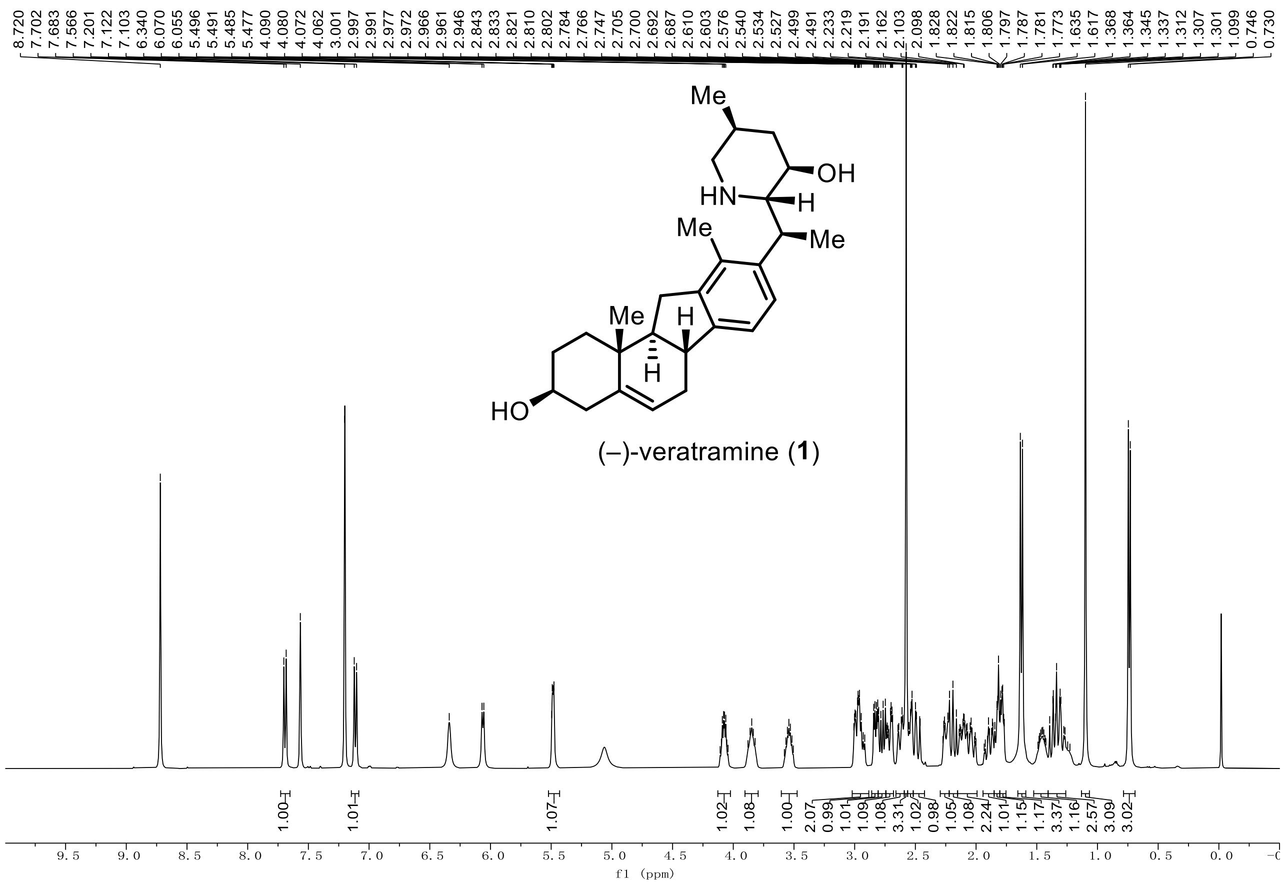

^{13}C NMR of **1** (100 MHz, C_5D_5N)

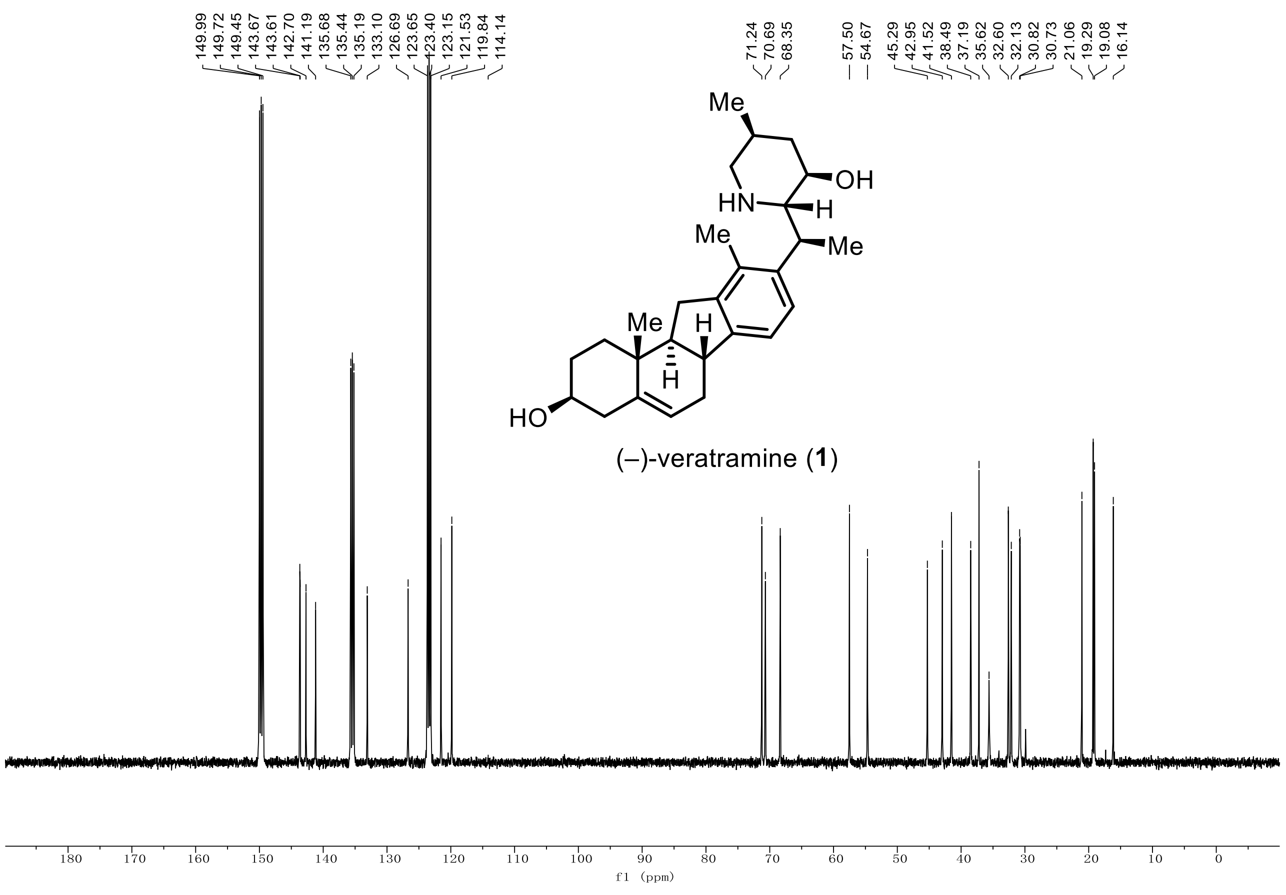

^{1}H NMR of **24** (400 MHz, $CDCl_3$)

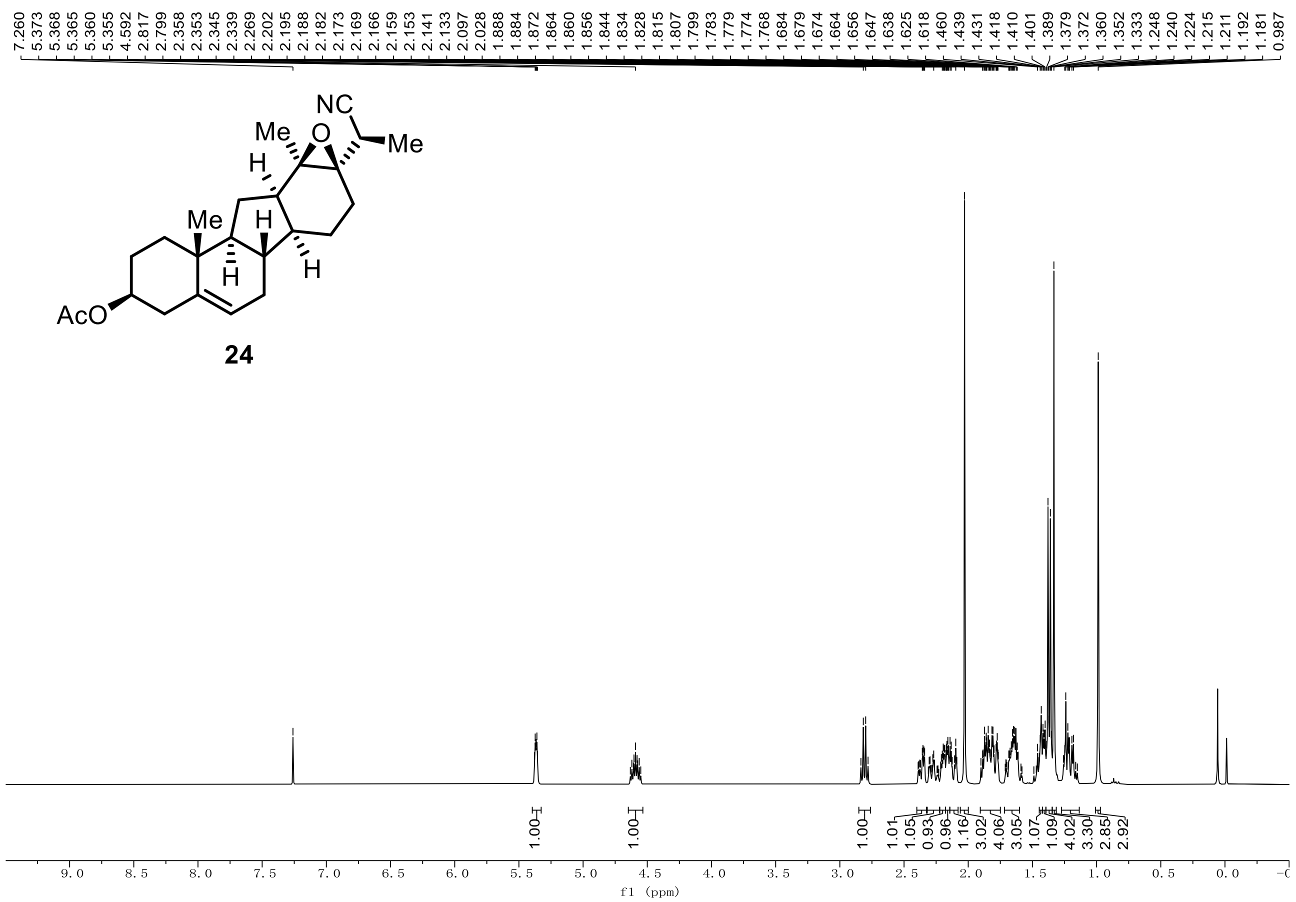

^{13}C NMR of **24** (100 MHz, $CDCl_3$)

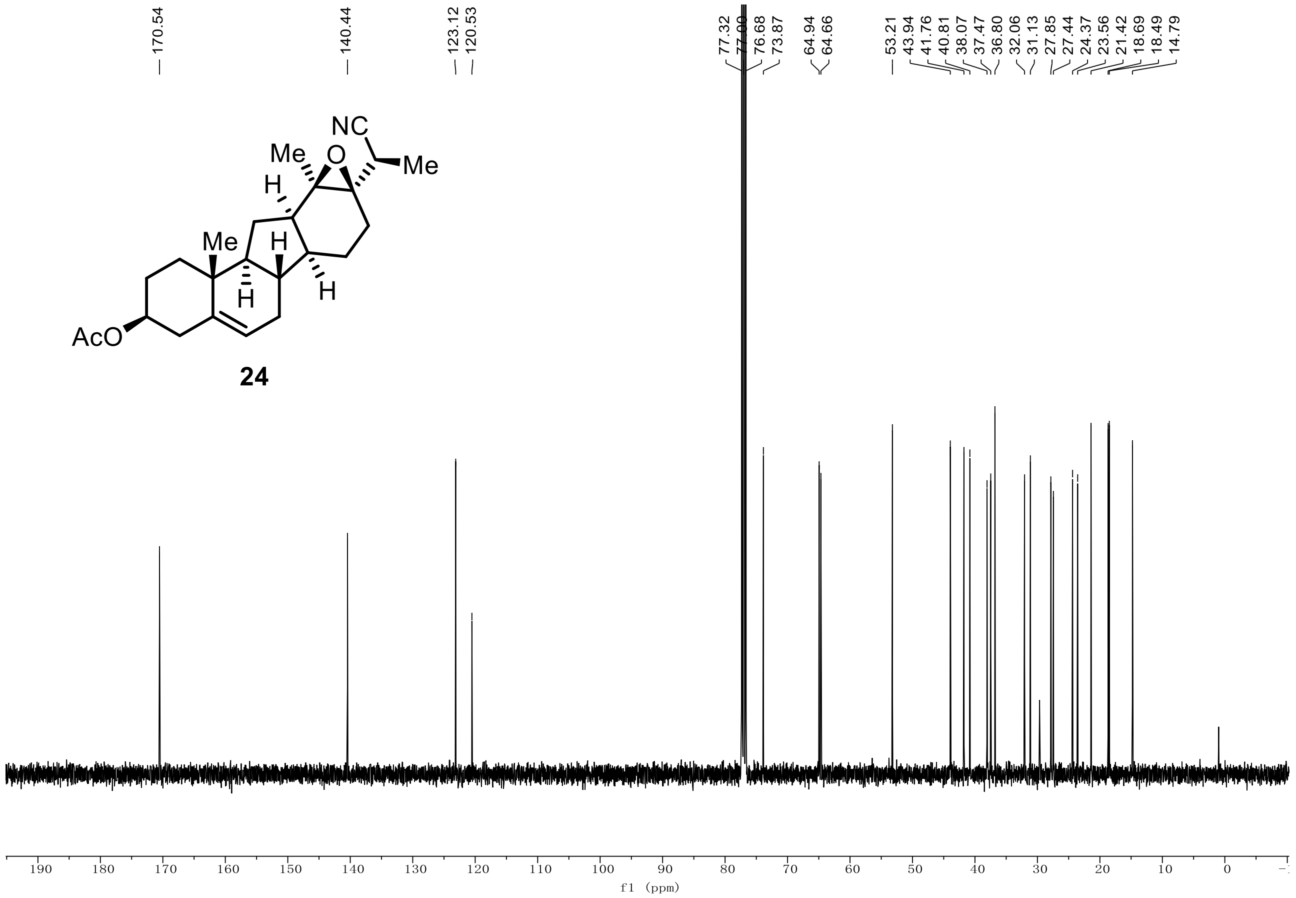

^{1}H NMR of **S5** (400 MHz, $CDCl_3$)

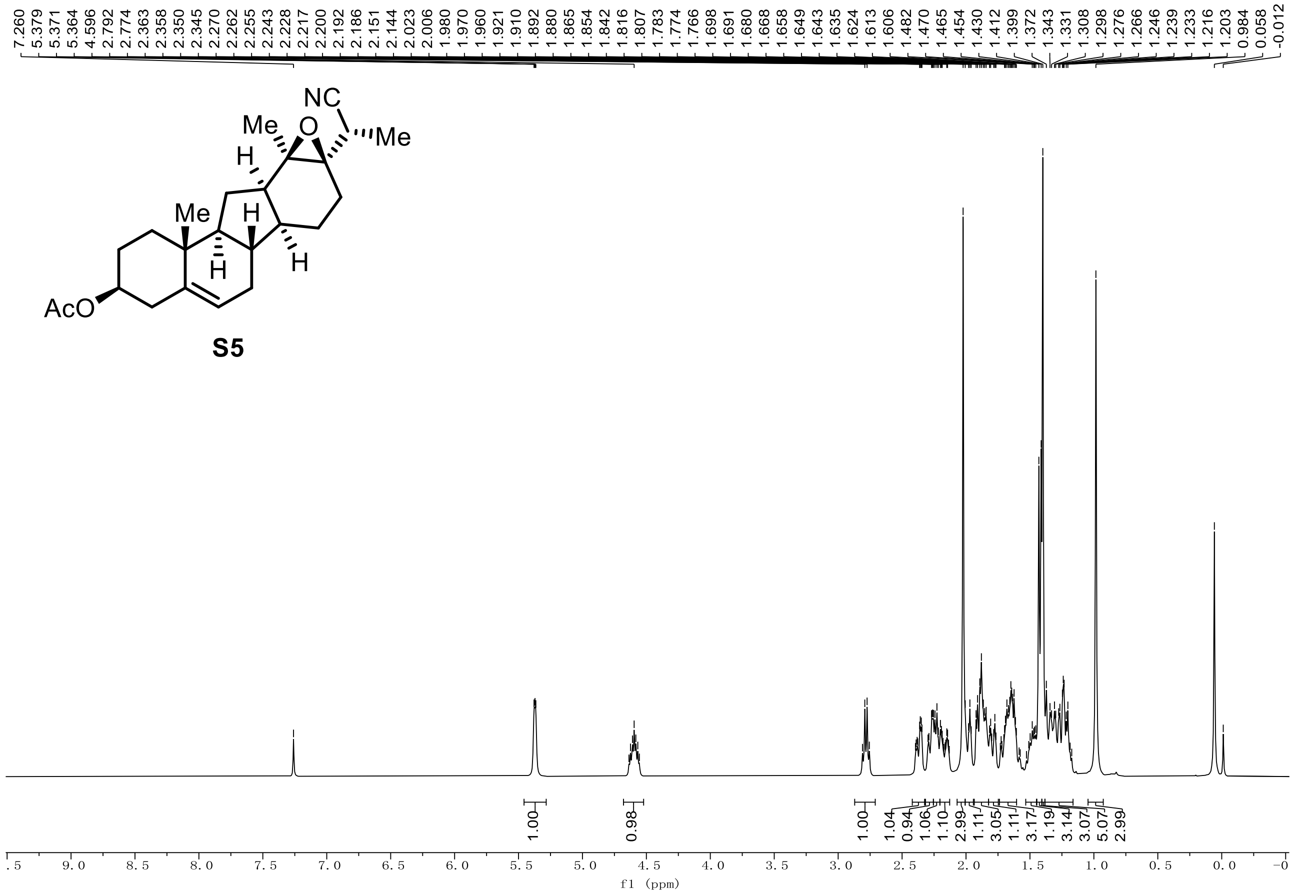

^{13}C NMR of **S5** (100 MHz, CDCl$_3$)

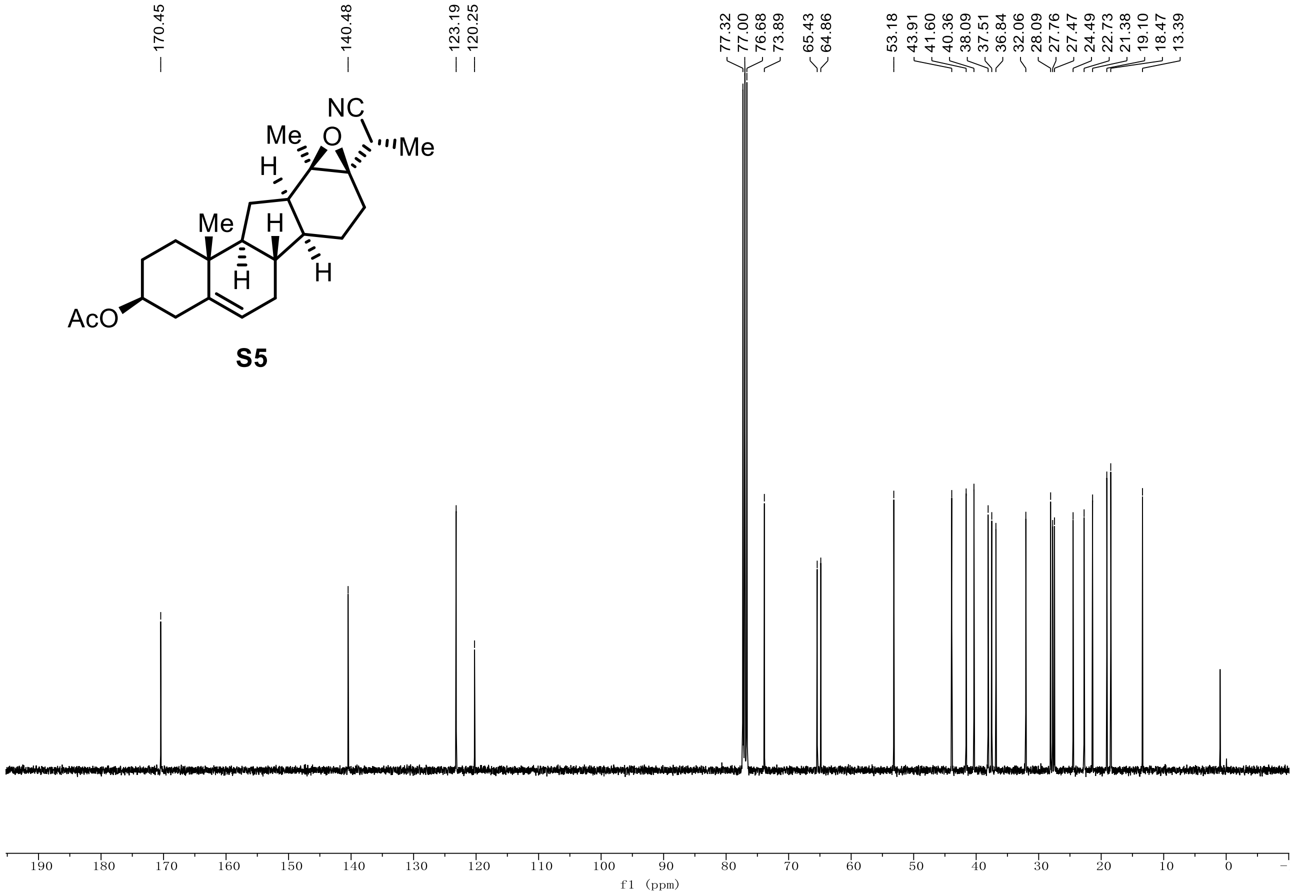

^{1}H NMR of **25** (400 MHz, CDCl$_3$)

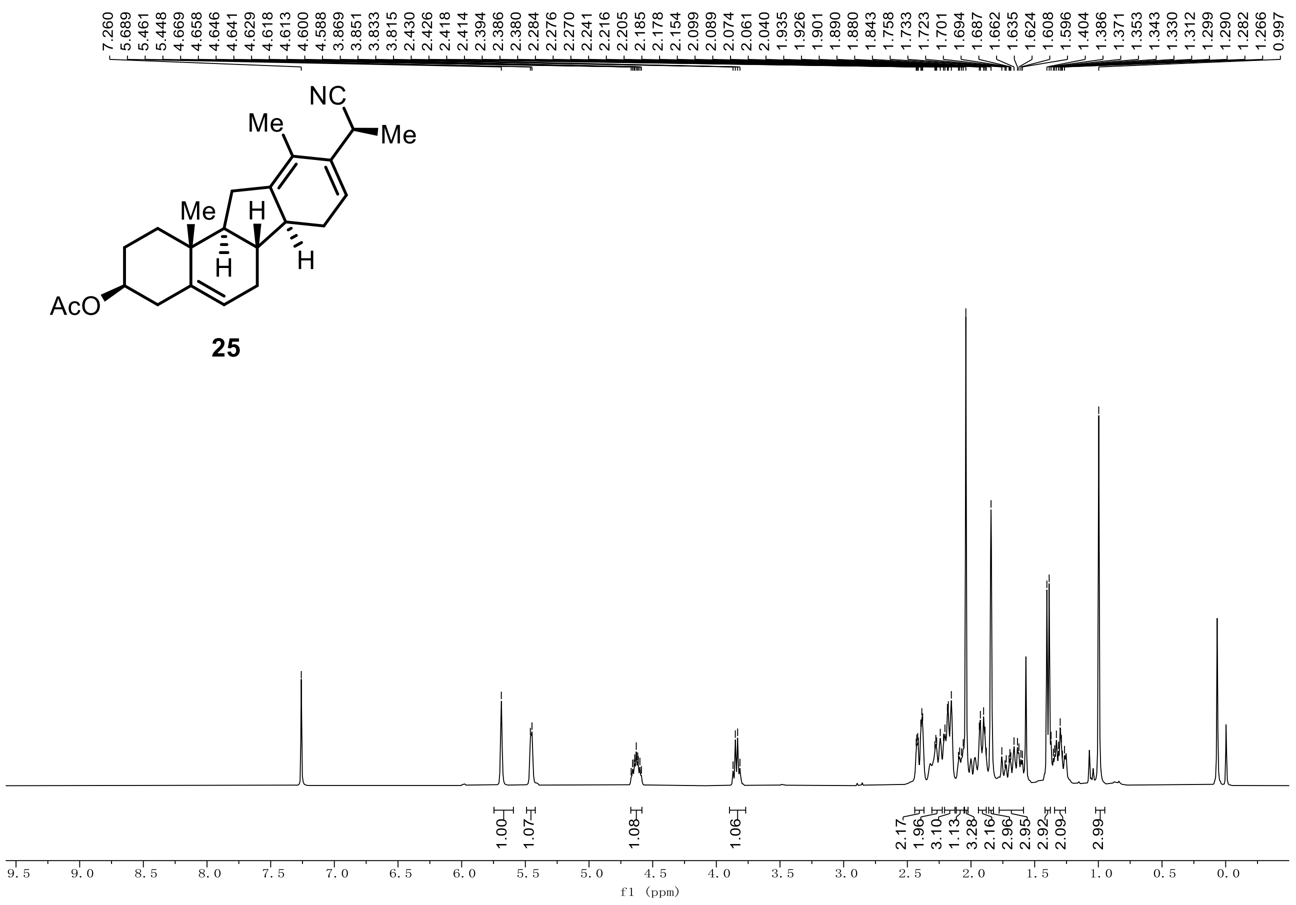

^{13}C NMR of **25** (100 MHz, $CDCl_3$)

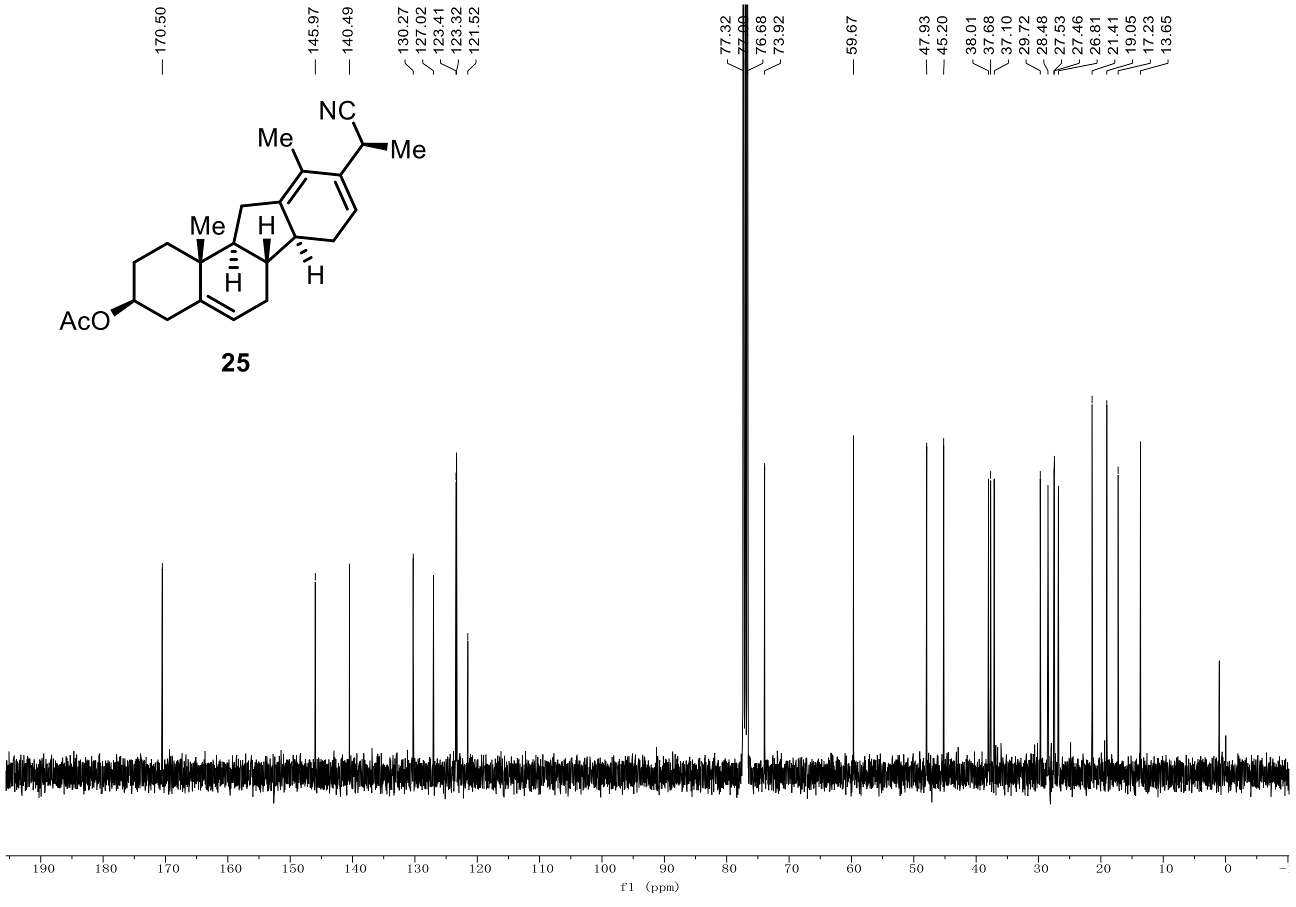

^{1}H NMR of **26** (400 MHz, $CDCl_3$)

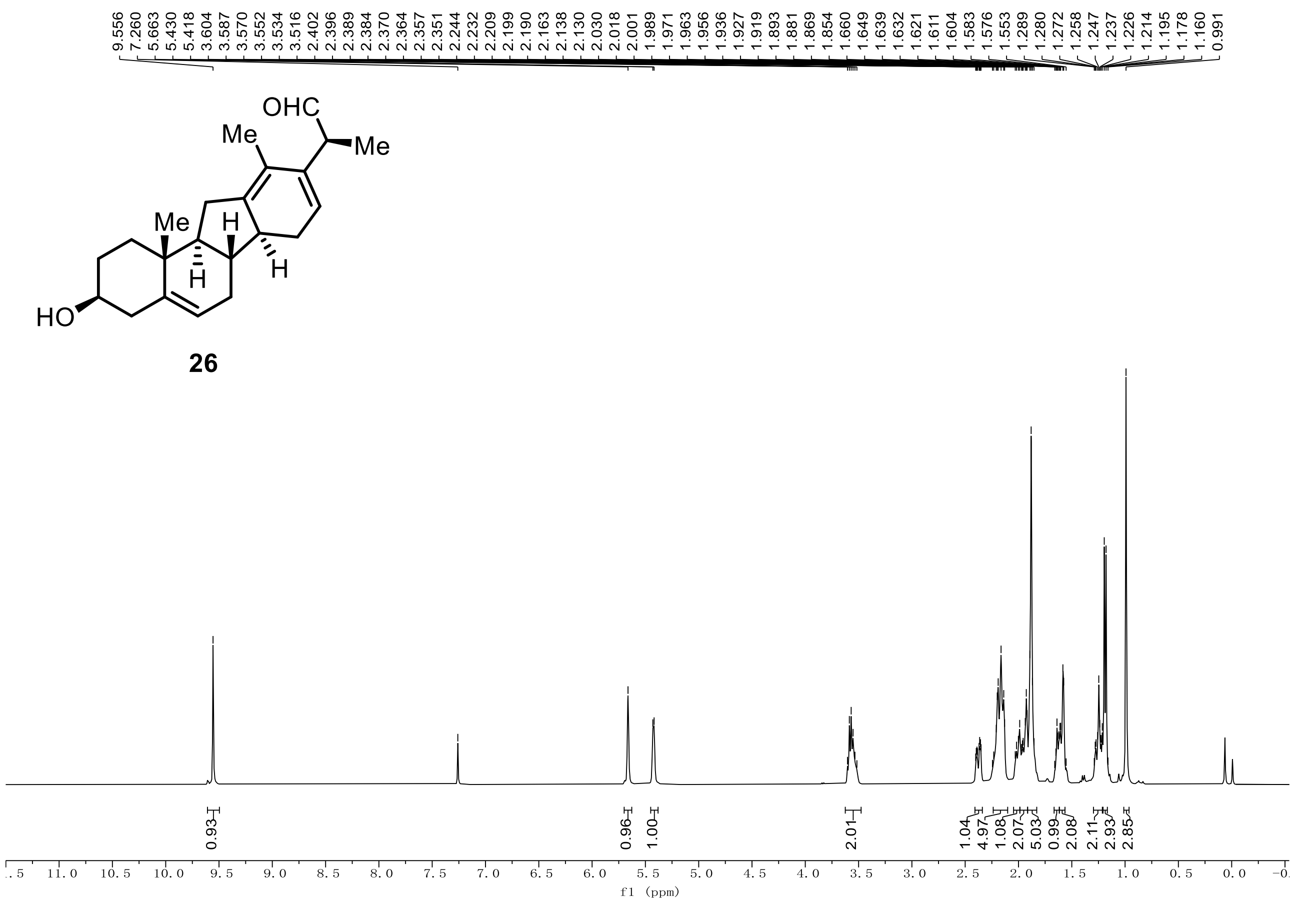

^{13}C NMR of **26** (100 MHz, $CDCl_3$)

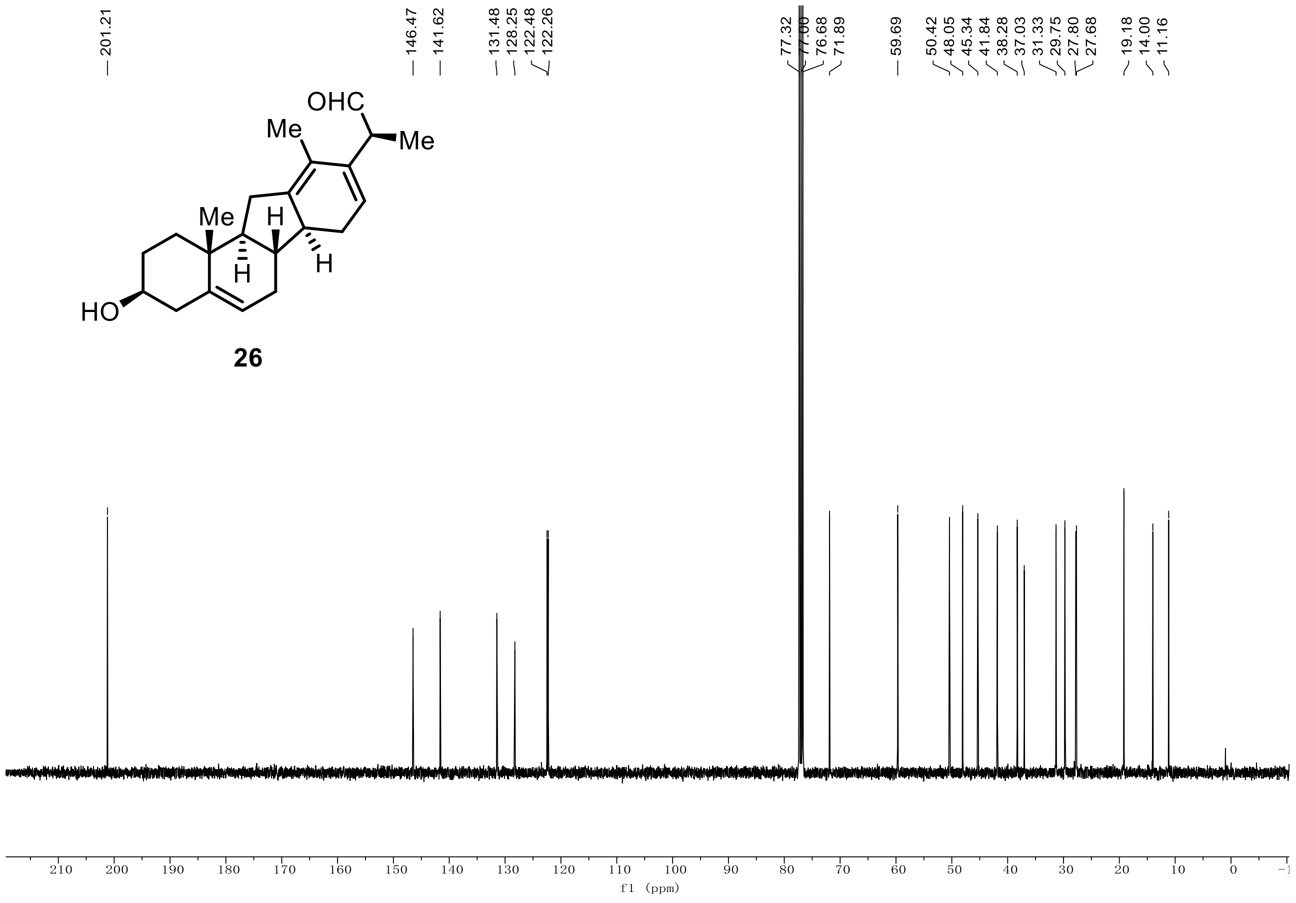

^{1}H NMR of **13** (400 MHz, $CDCl_3$)

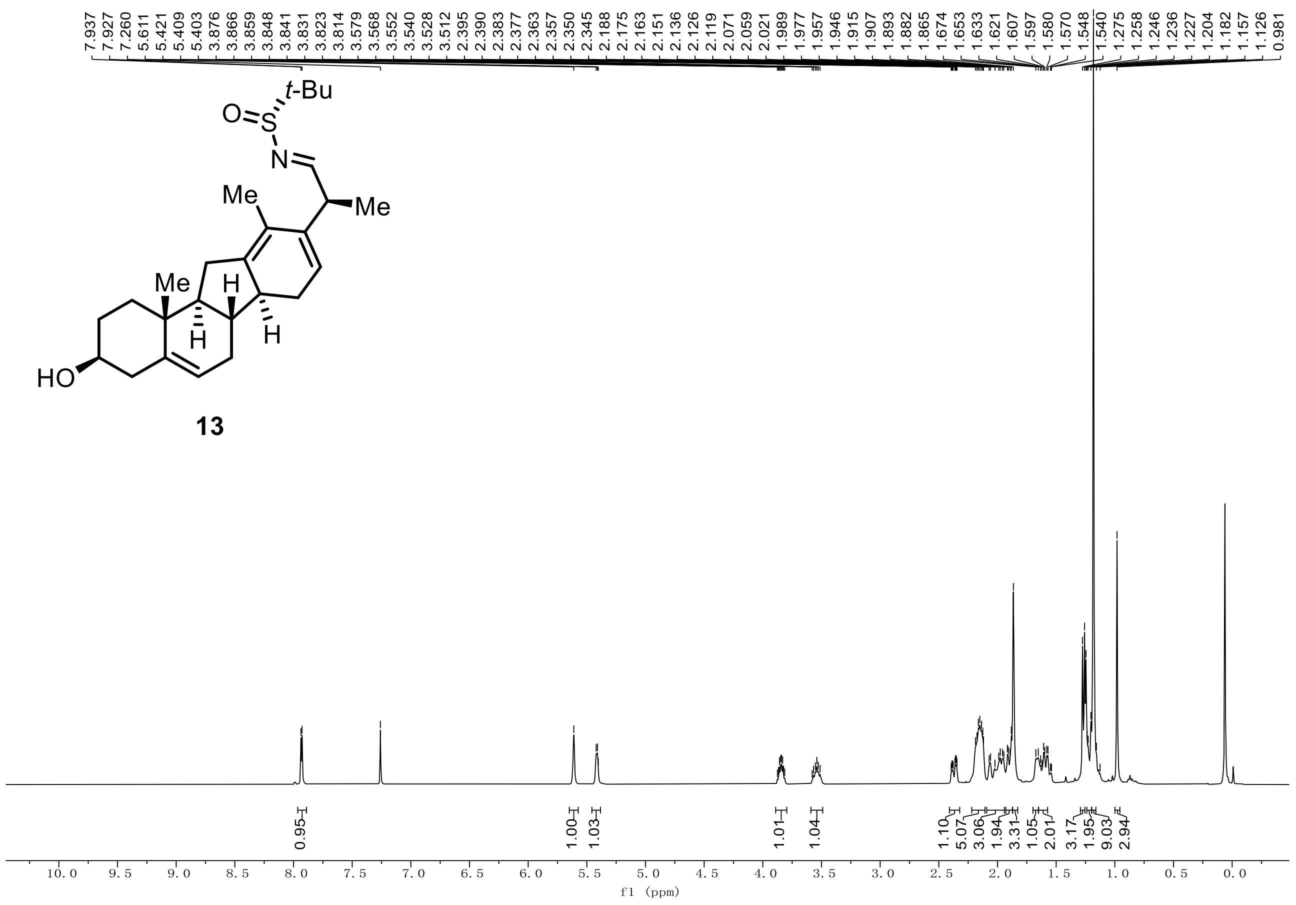

^{13}C NMR of **13** (100 MHz, $CDCl_3$)

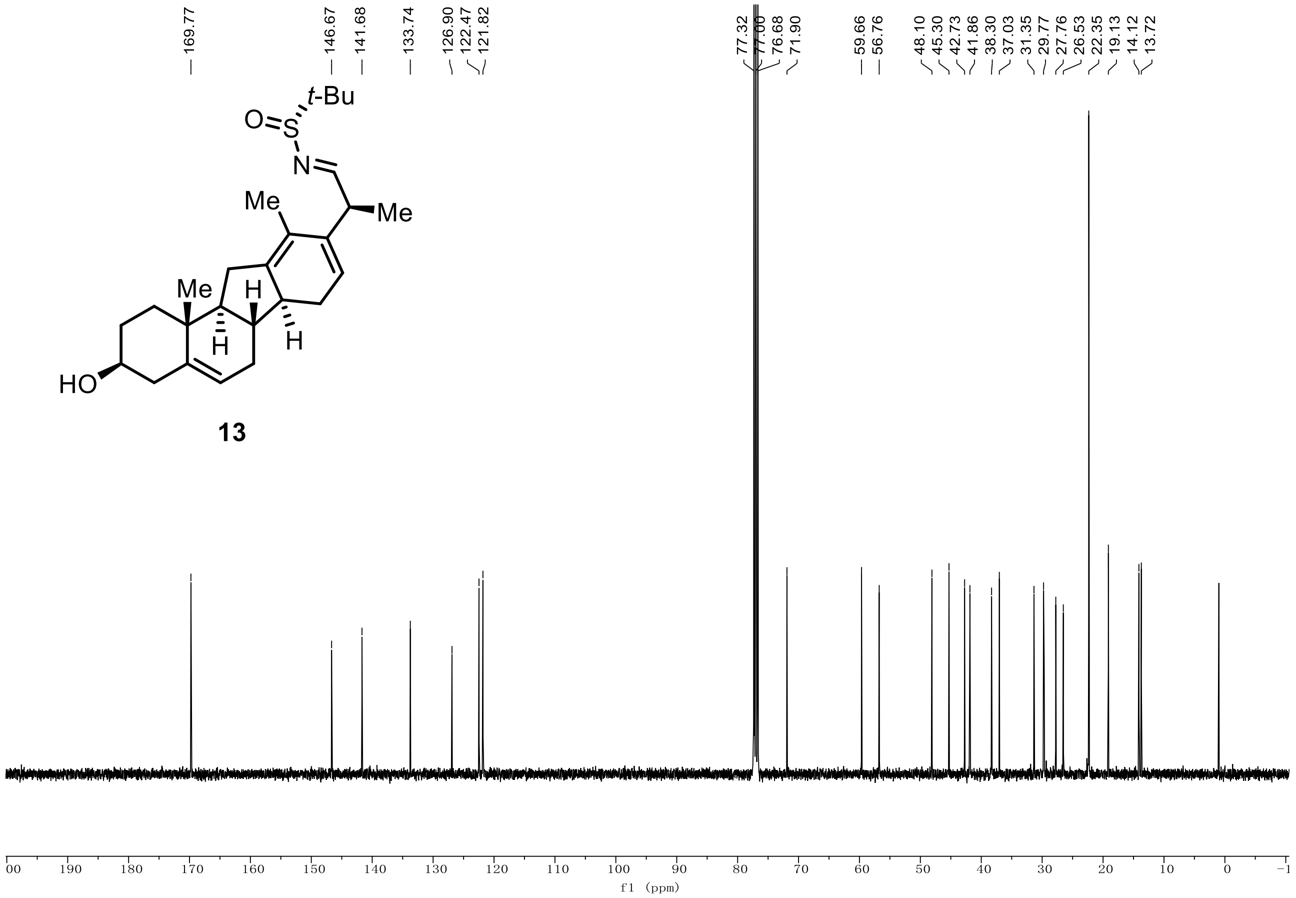

^{1}H NMR of **30** (400 MHz, $CDCl_3$)

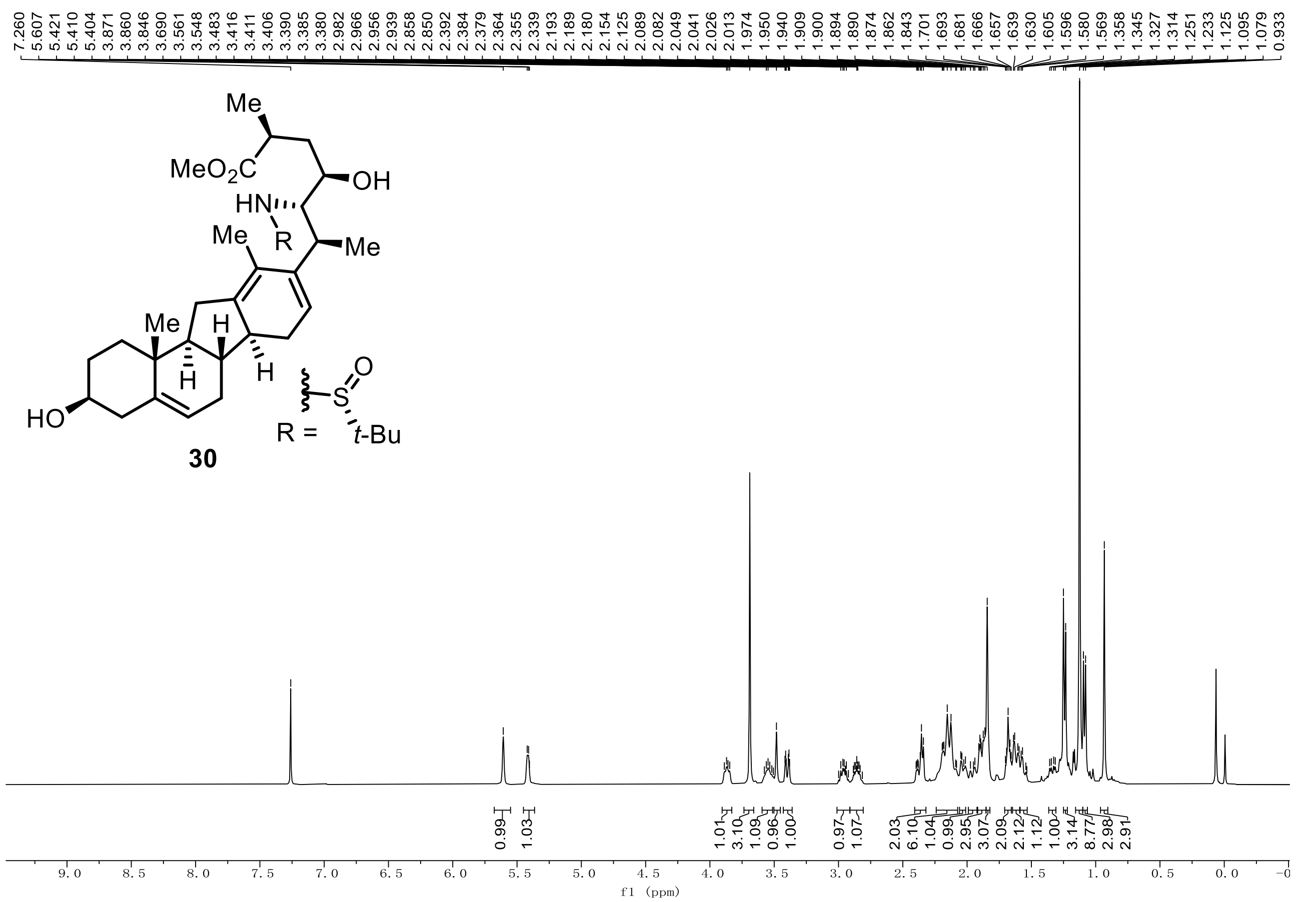

^{13}C NMR of **30** (100 MHz, $CDCl_3$)

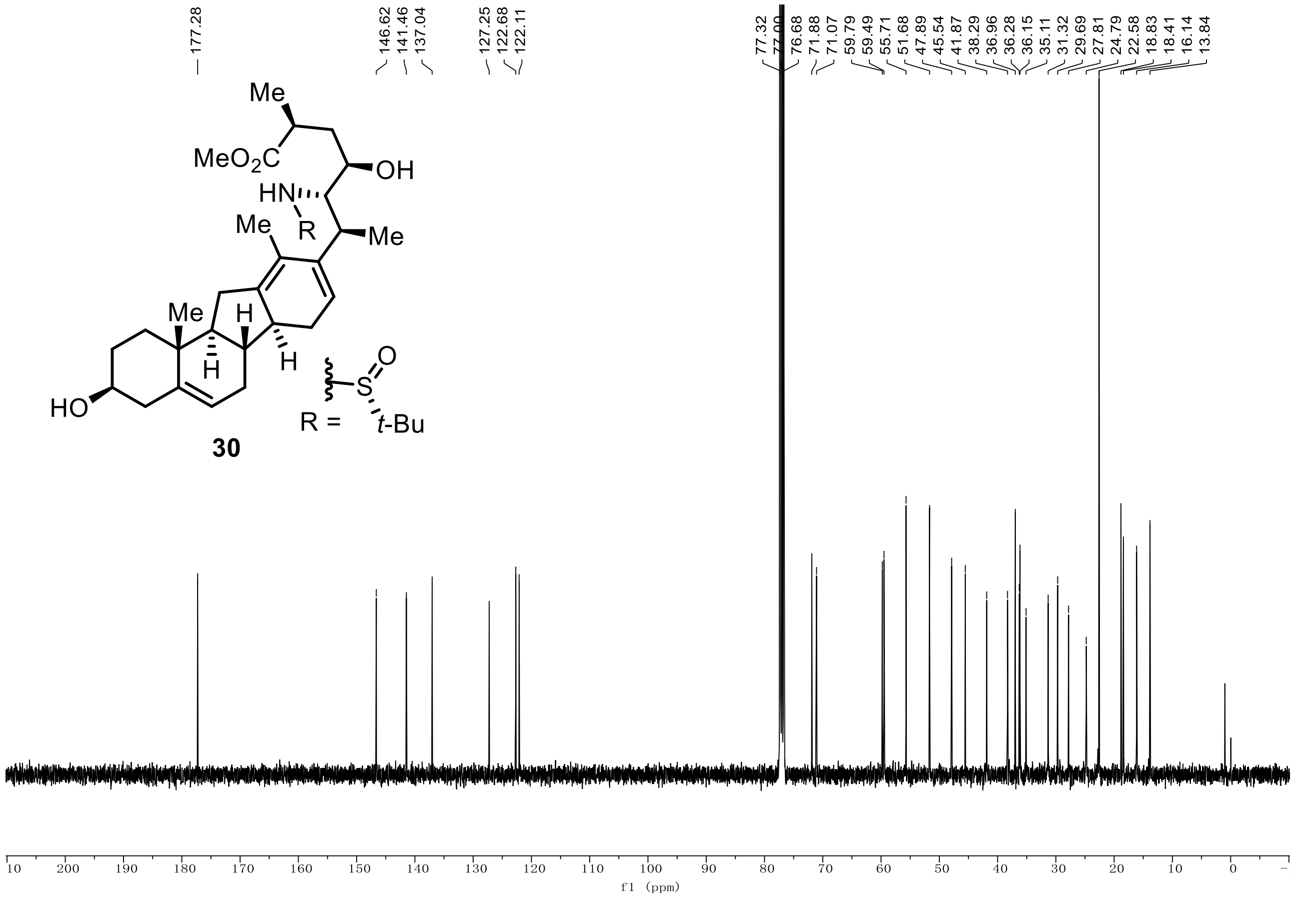

^{1}H NMR of **25'** (400 MHz, $CDCl_3$)

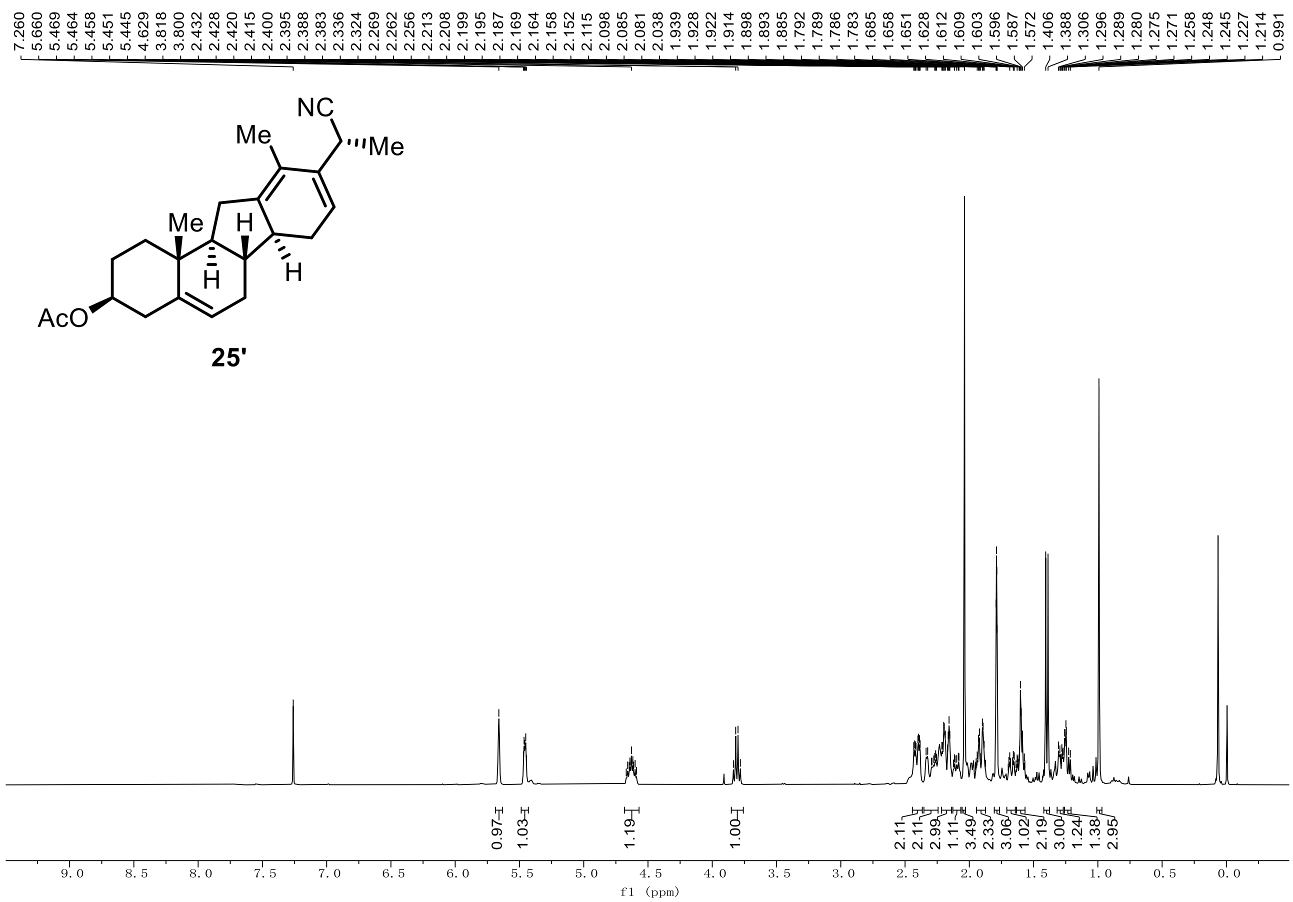

^{13}C NMR of **25'** (100 MHz, $CDCl_3$)

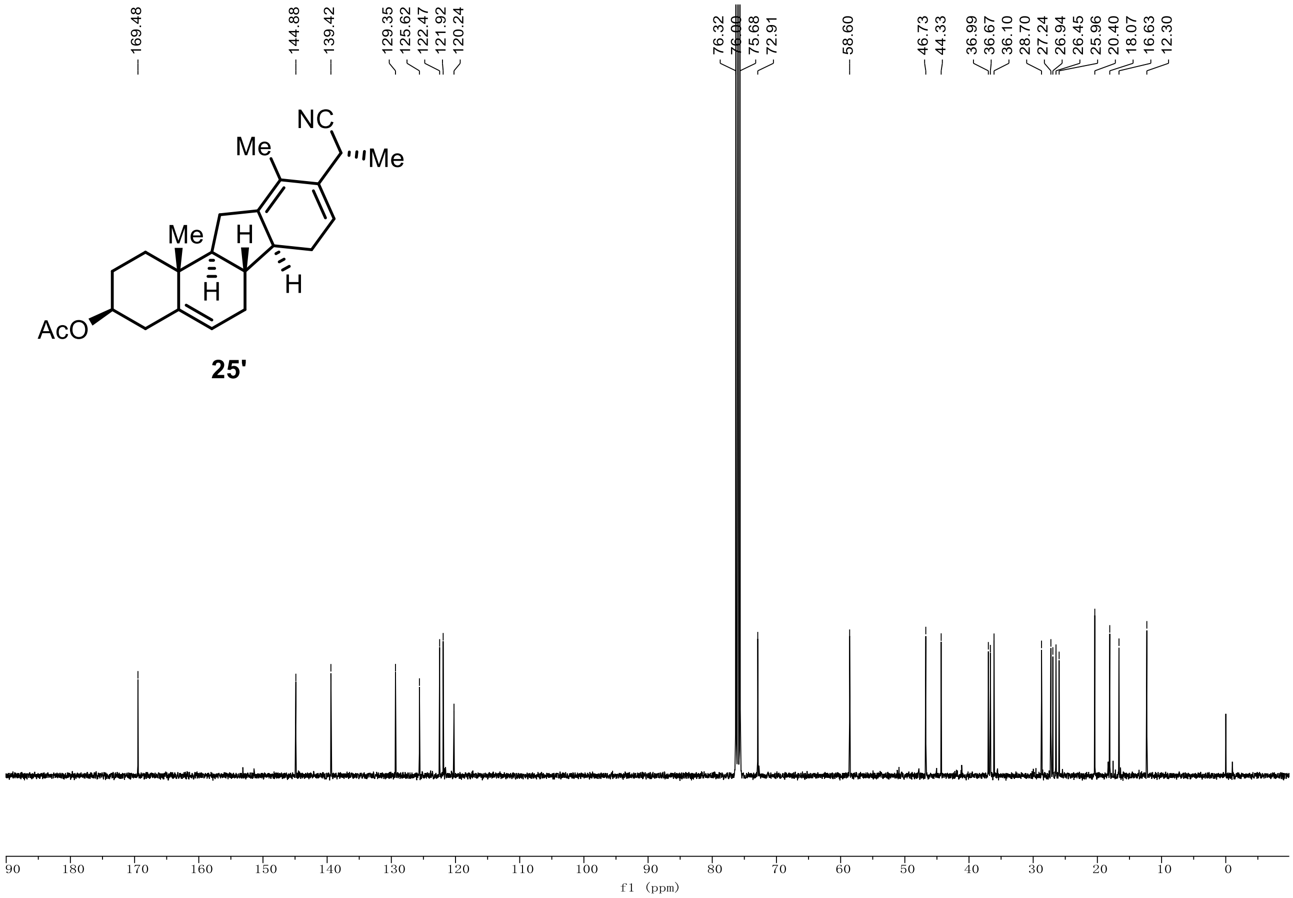

^{1}H NMR of **26'** (400 MHz, $CDCl_3$)

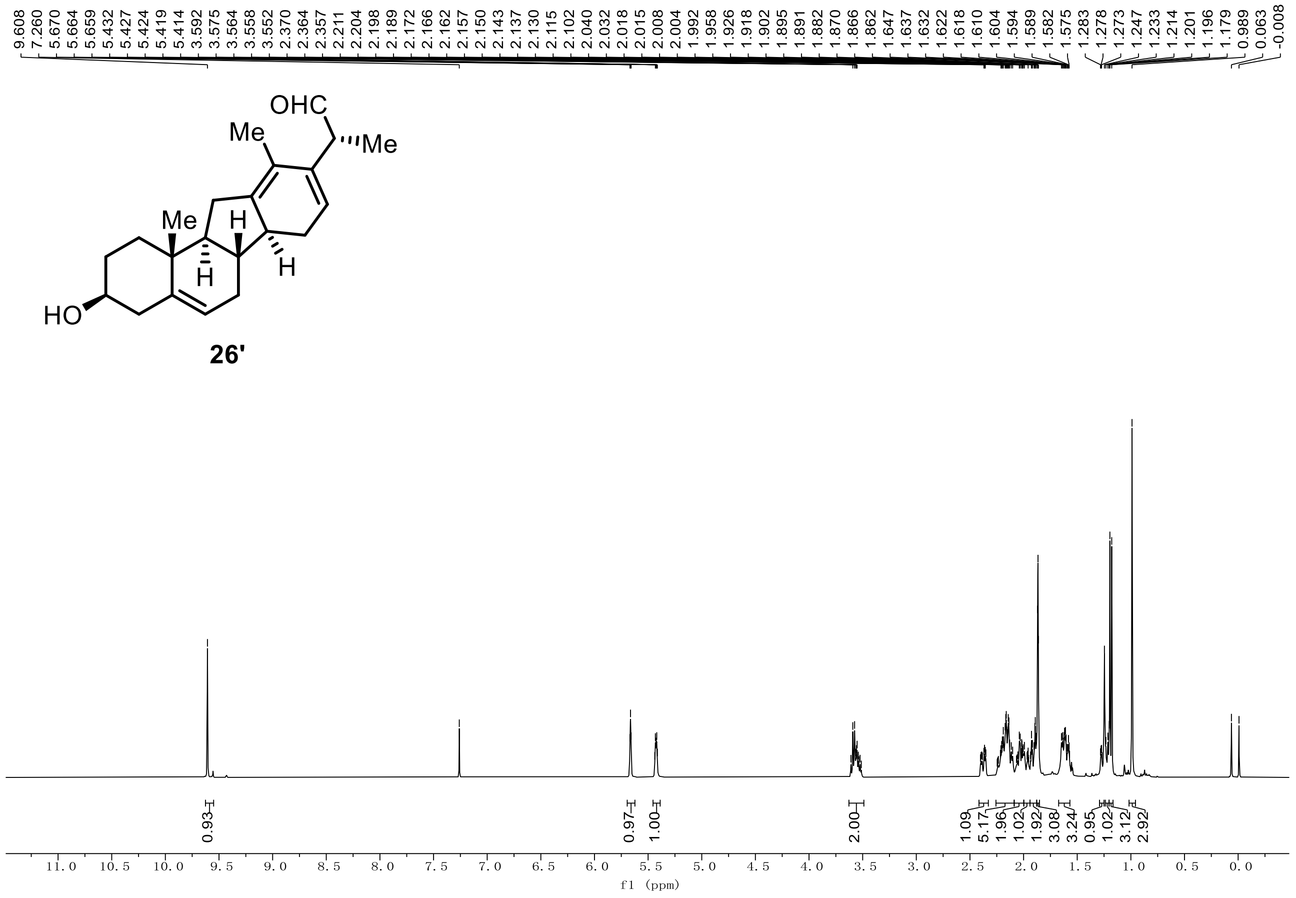

^{13}C NMR of **26'** (100 MHz, $CDCl_3$)

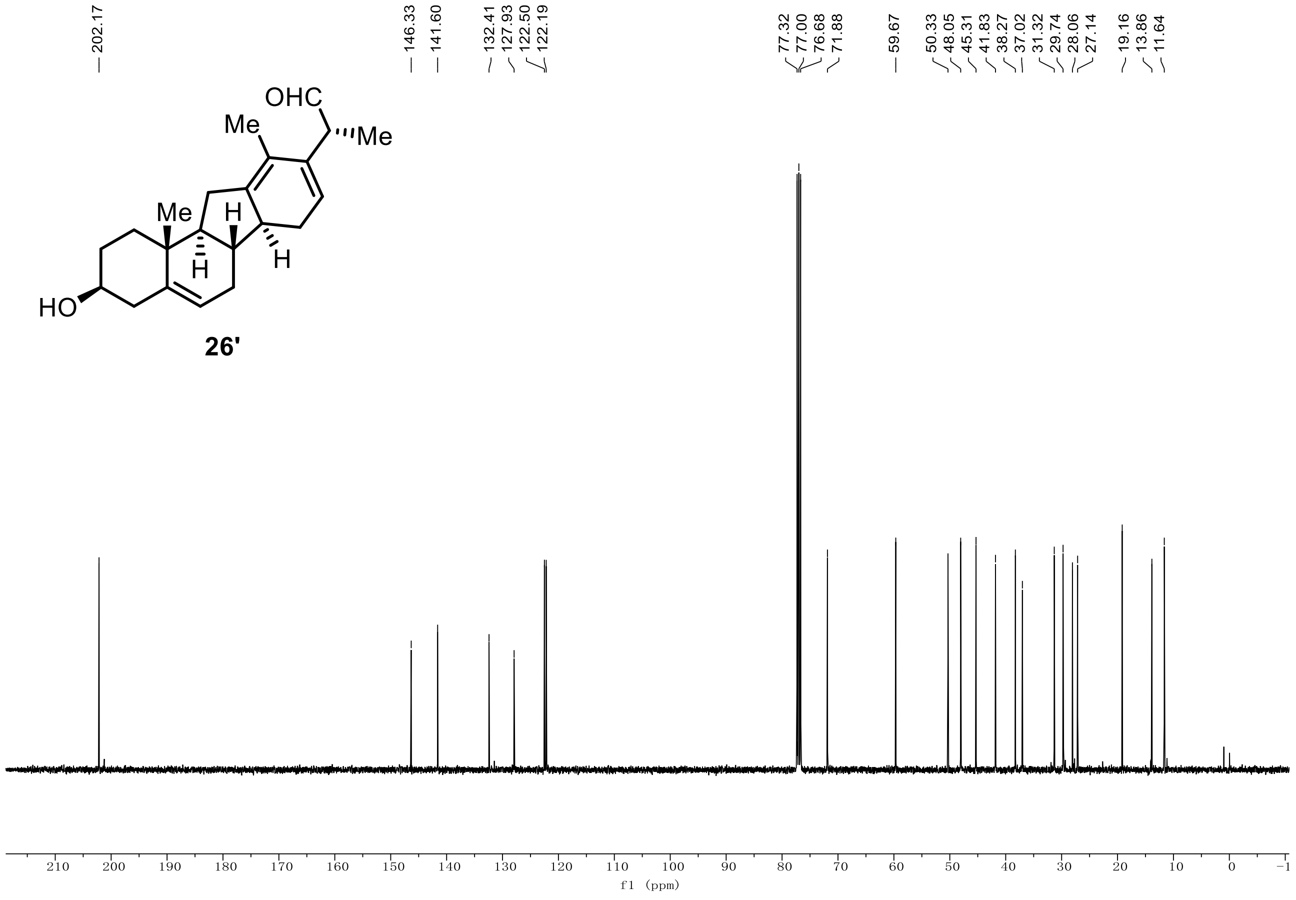

^{1}H NMR of **13'** (400 MHz, $CDCl_3$)

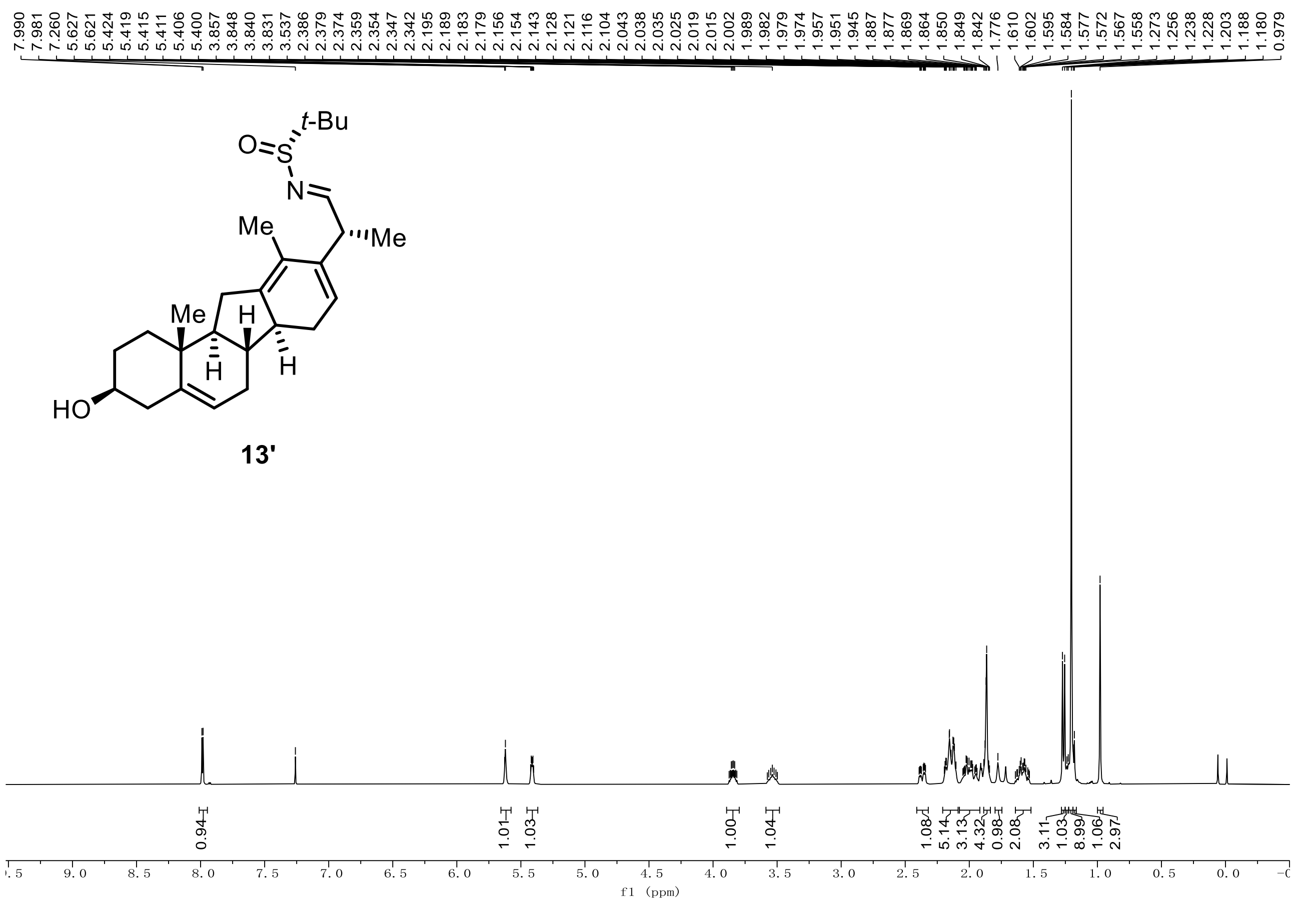

13'

^{13}C NMR of **13'** (100 MHz, CDCl$_3$)

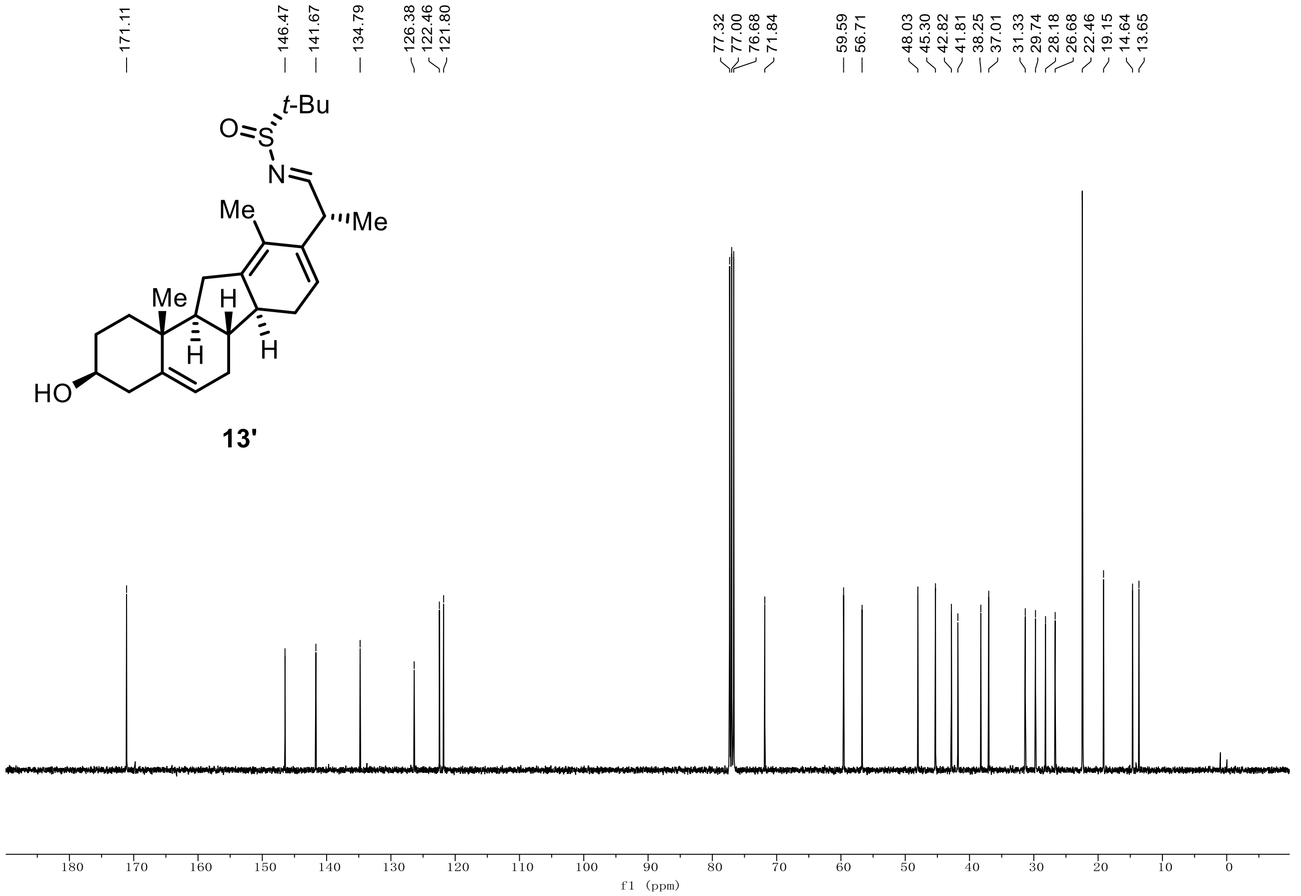

13'

^{1}H NMR of **30'** (400 MHz, CDCl$_3$)

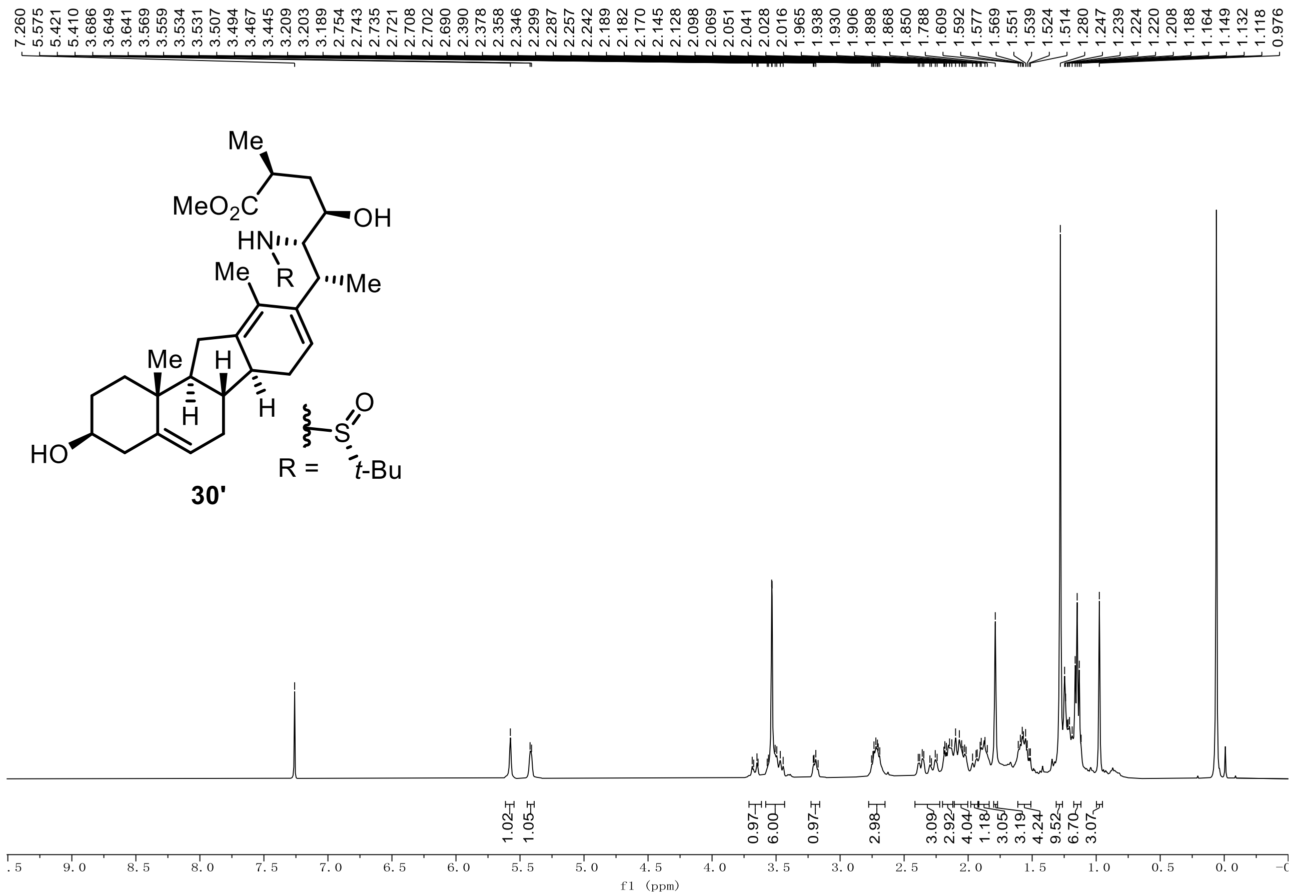

^{13}C NMR of **30'** (100 MHz, $CDCl_3$)

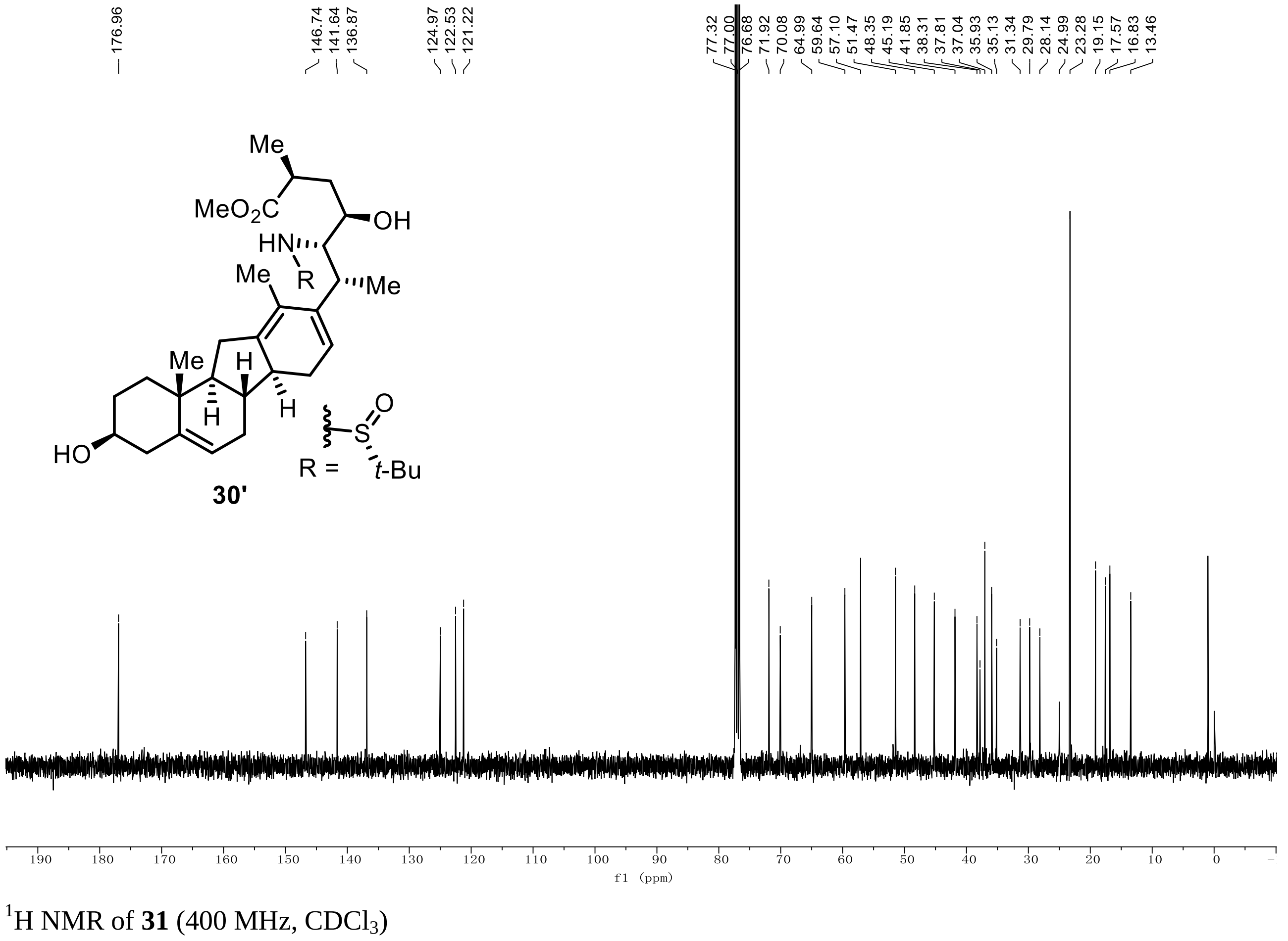

^{1}H NMR of **31** (400 MHz, $CDCl_3$)

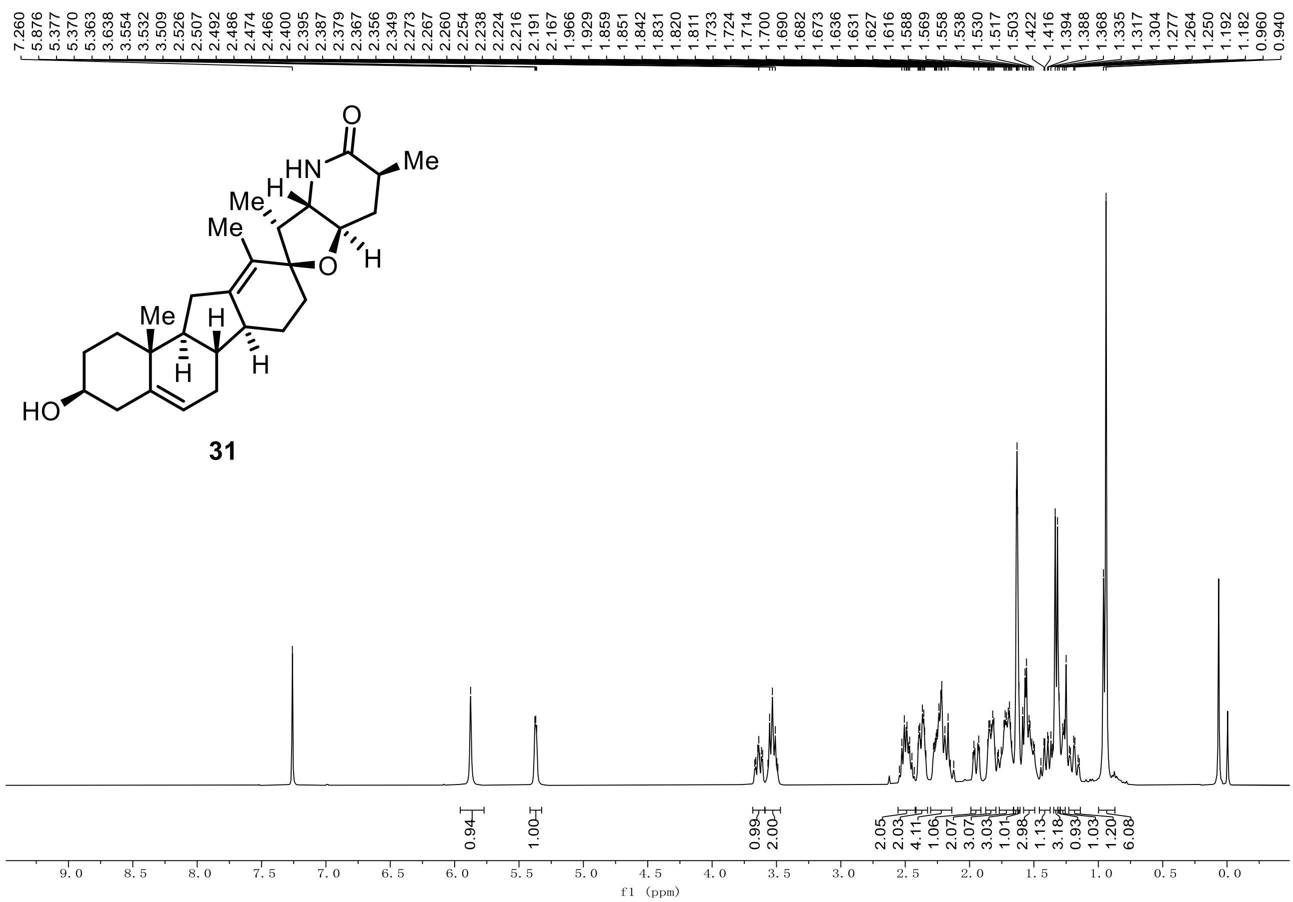

^{13}C NMR of **31** (100 MHz, $CDCl_3$)

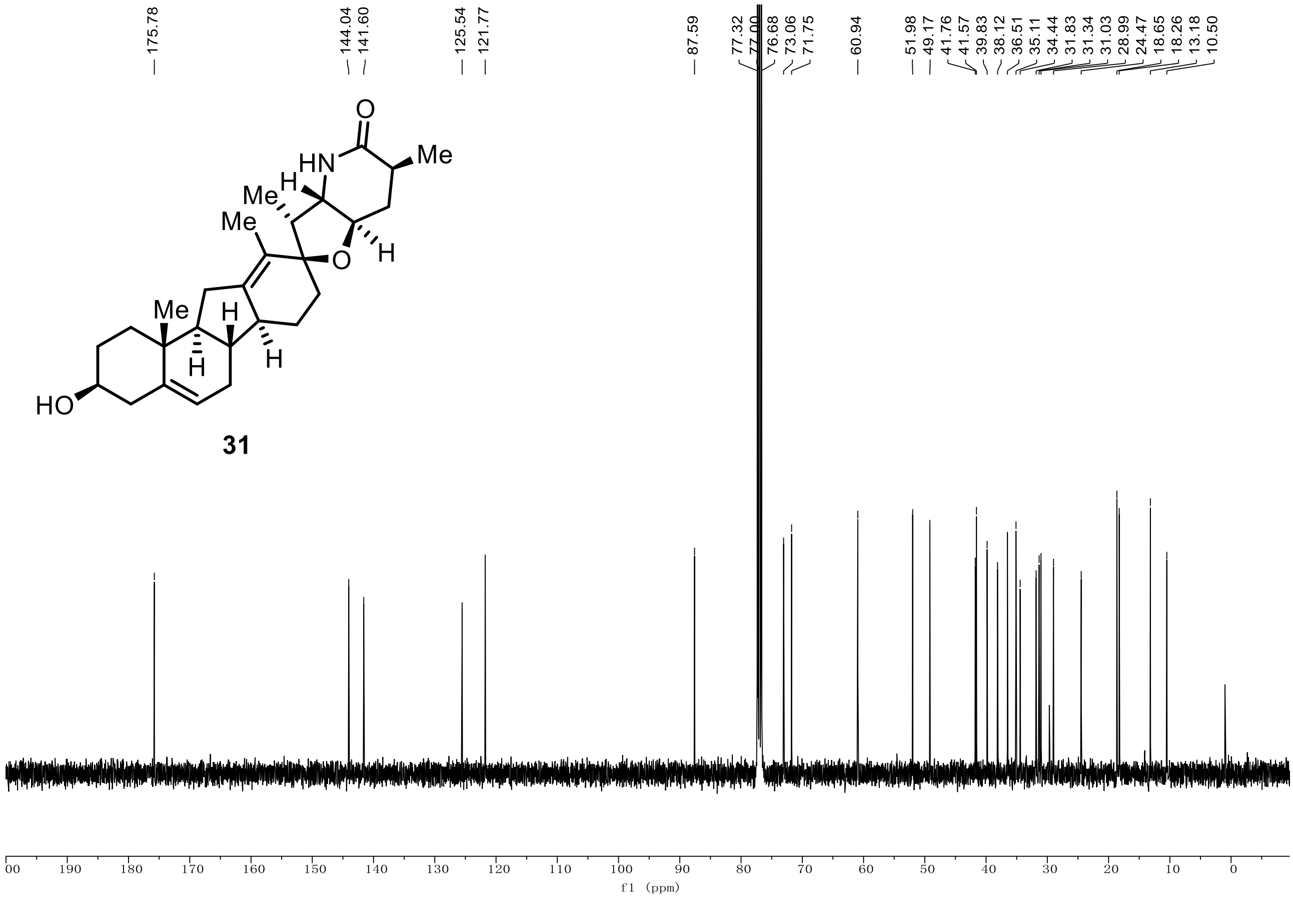

^{1}H NMR of **4** (400 MHz, CD_2Cl_2)

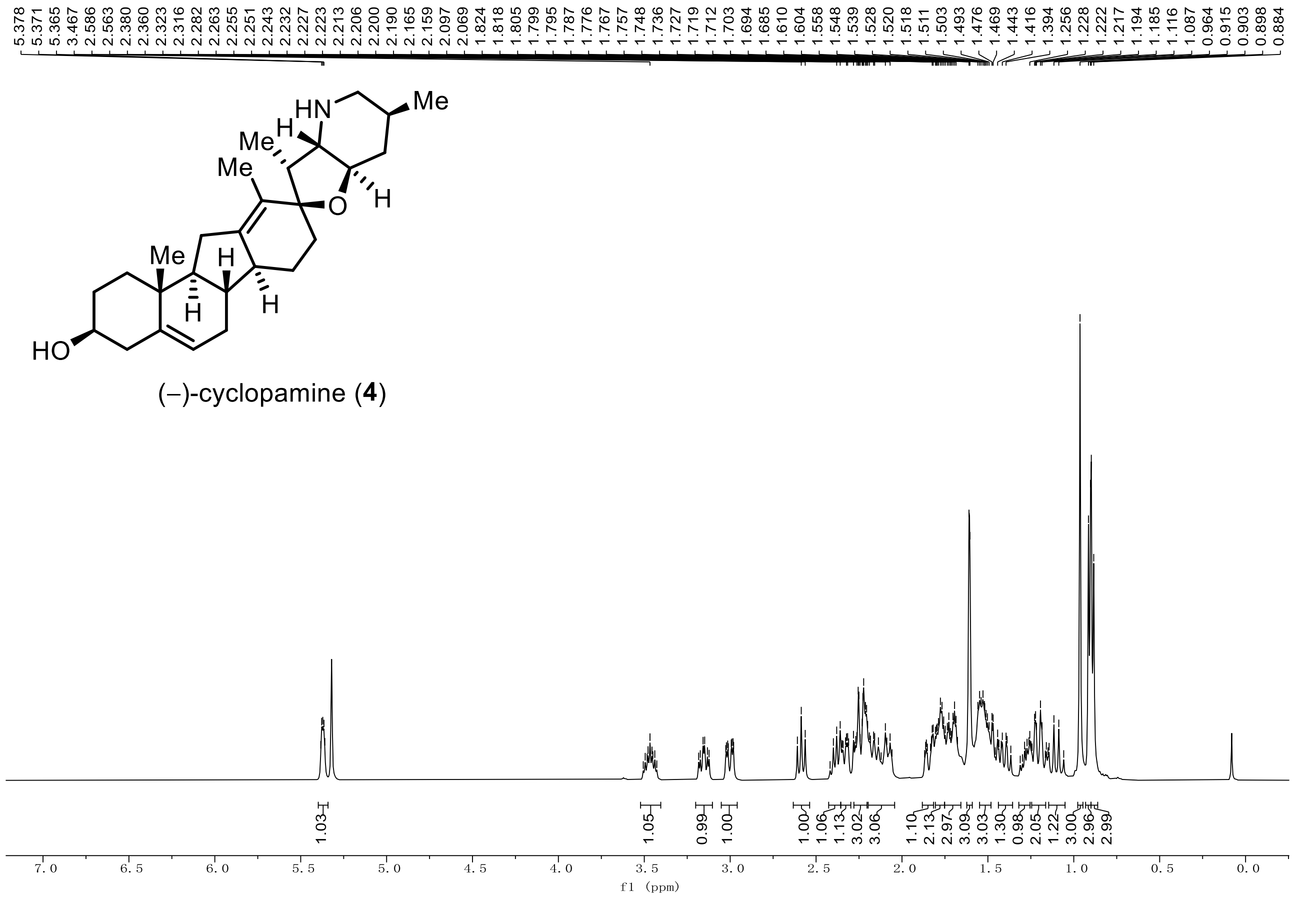

^{13}C NMR of **4** (100 MHz, CD_2Cl_2)

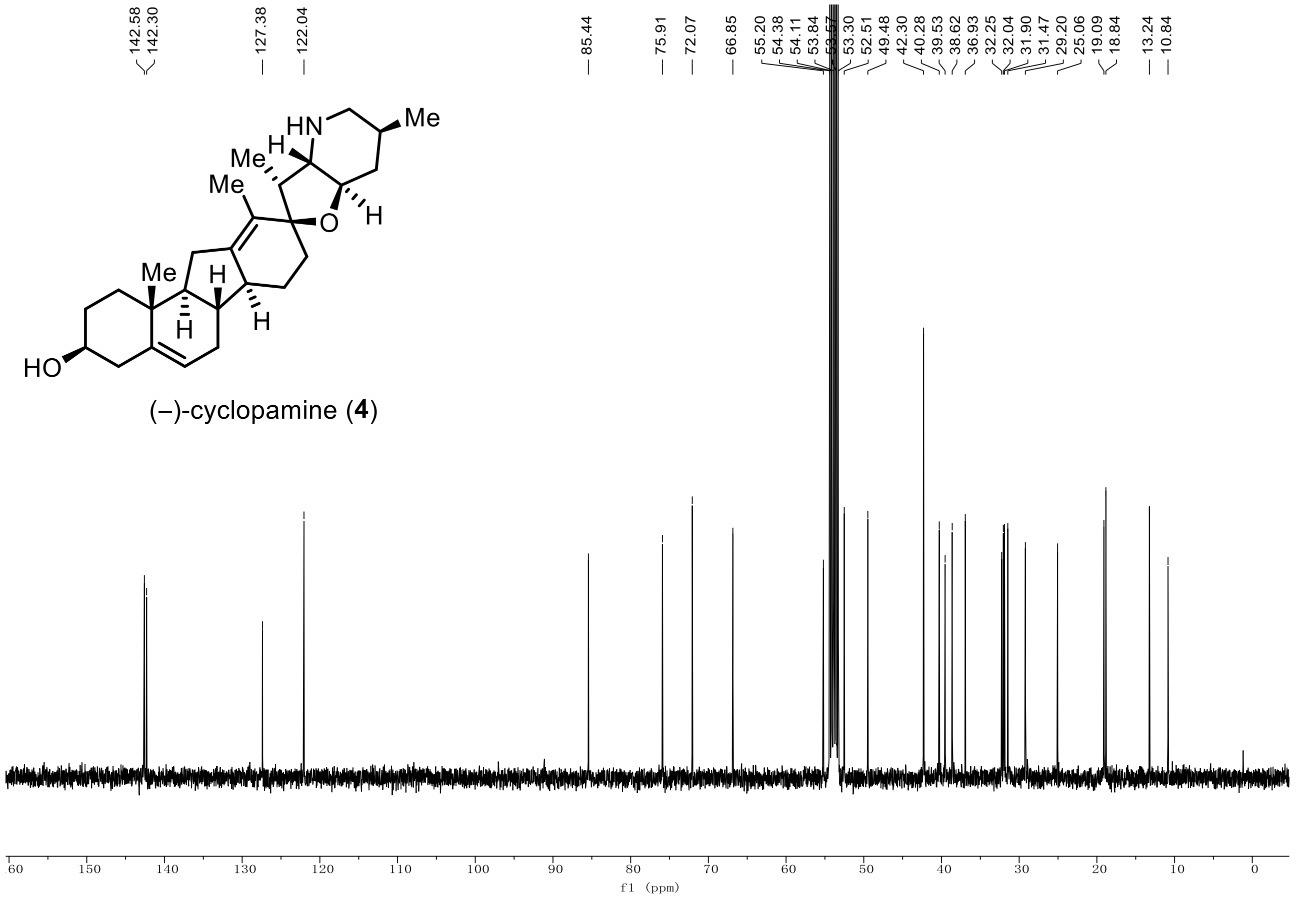

^{1}H NMR of **S8** (400 MHz, $CDCl_3$)

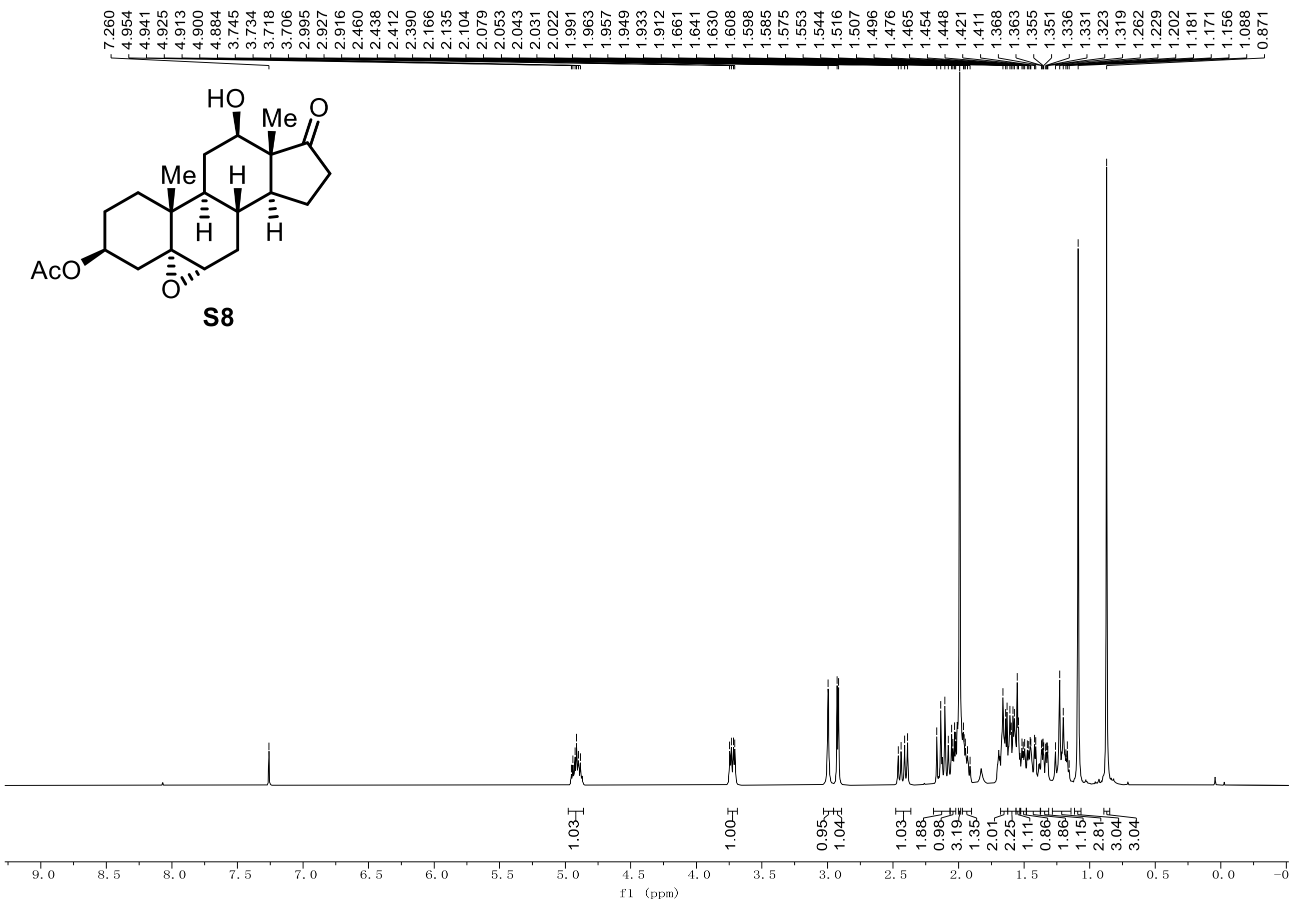

^{13}C NMR of **S8** (150 MHz, $CDCl_3$)

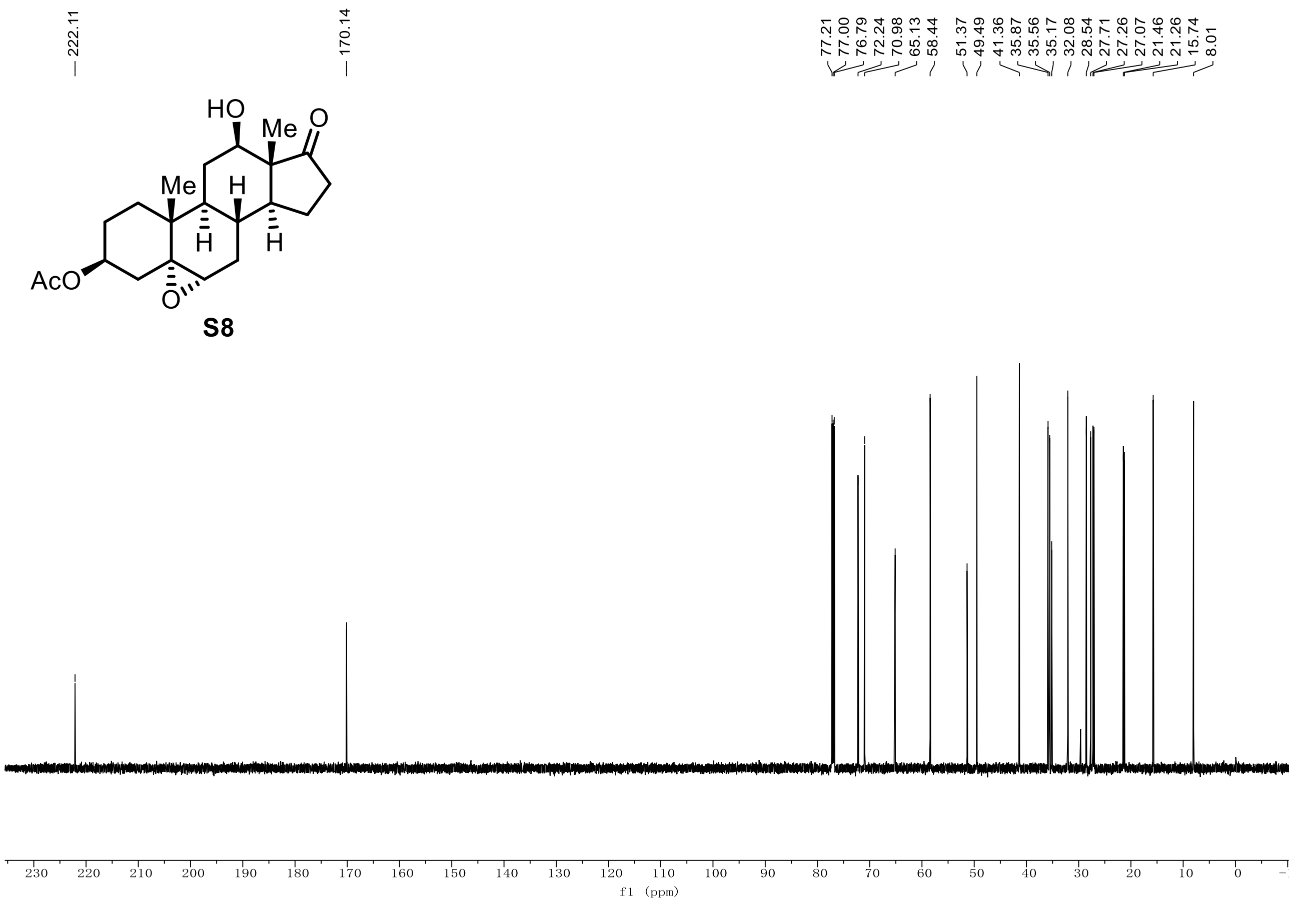

^{1}H NMR of **S9** (400 MHz, $CDCl_3$)

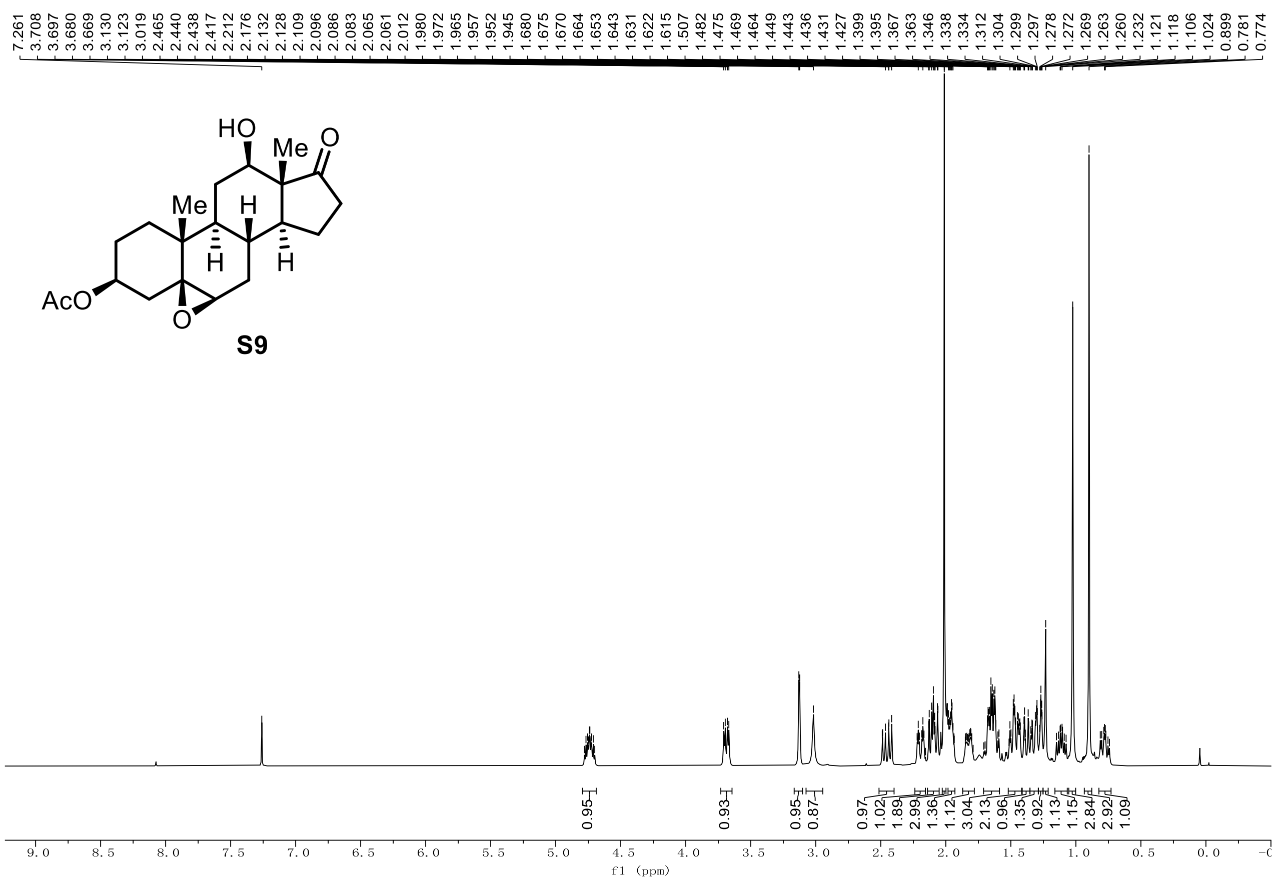

^{13}C NMR of **S9** (150 MHz, $CDCl_3$)

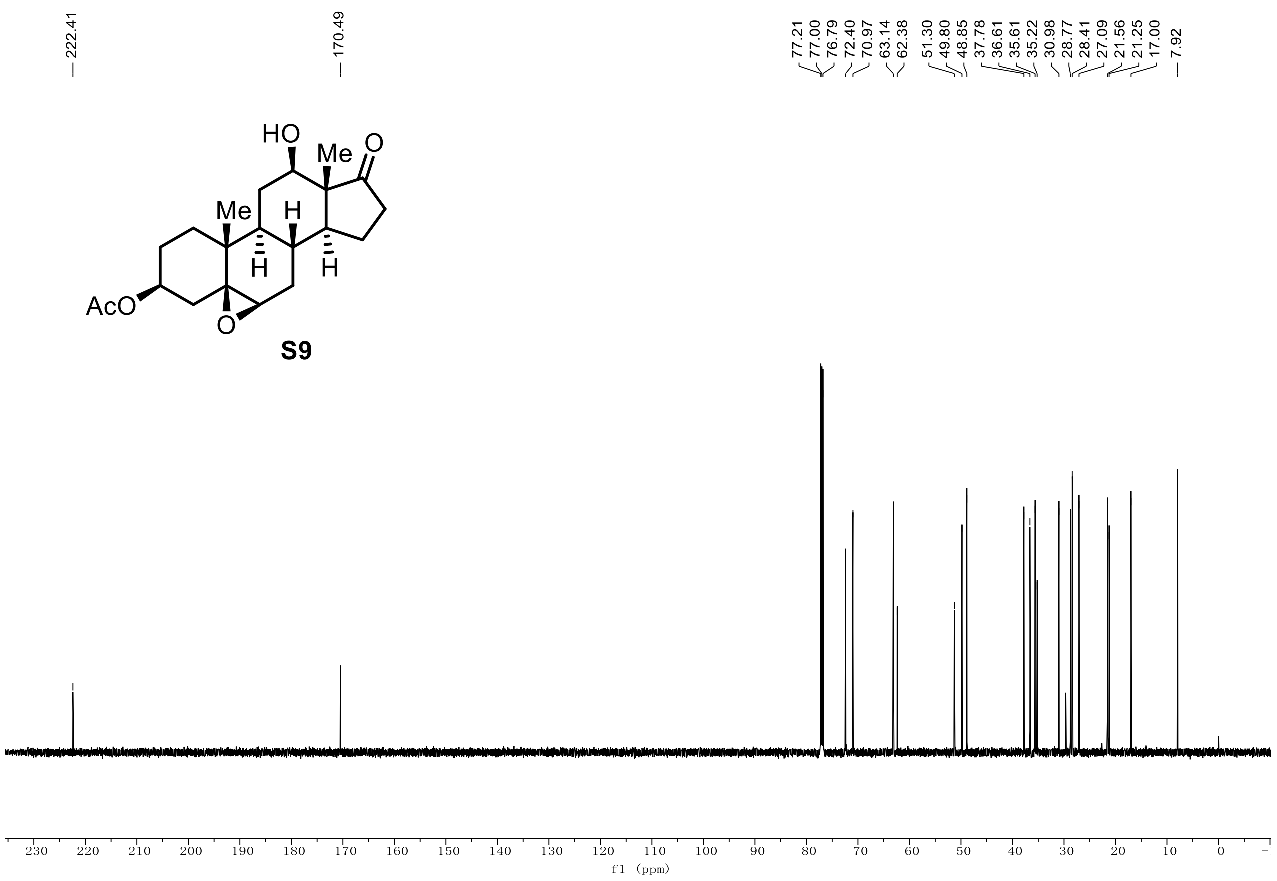

^{1}H NMR of **S10** (400 MHz, $CDCl_3$)

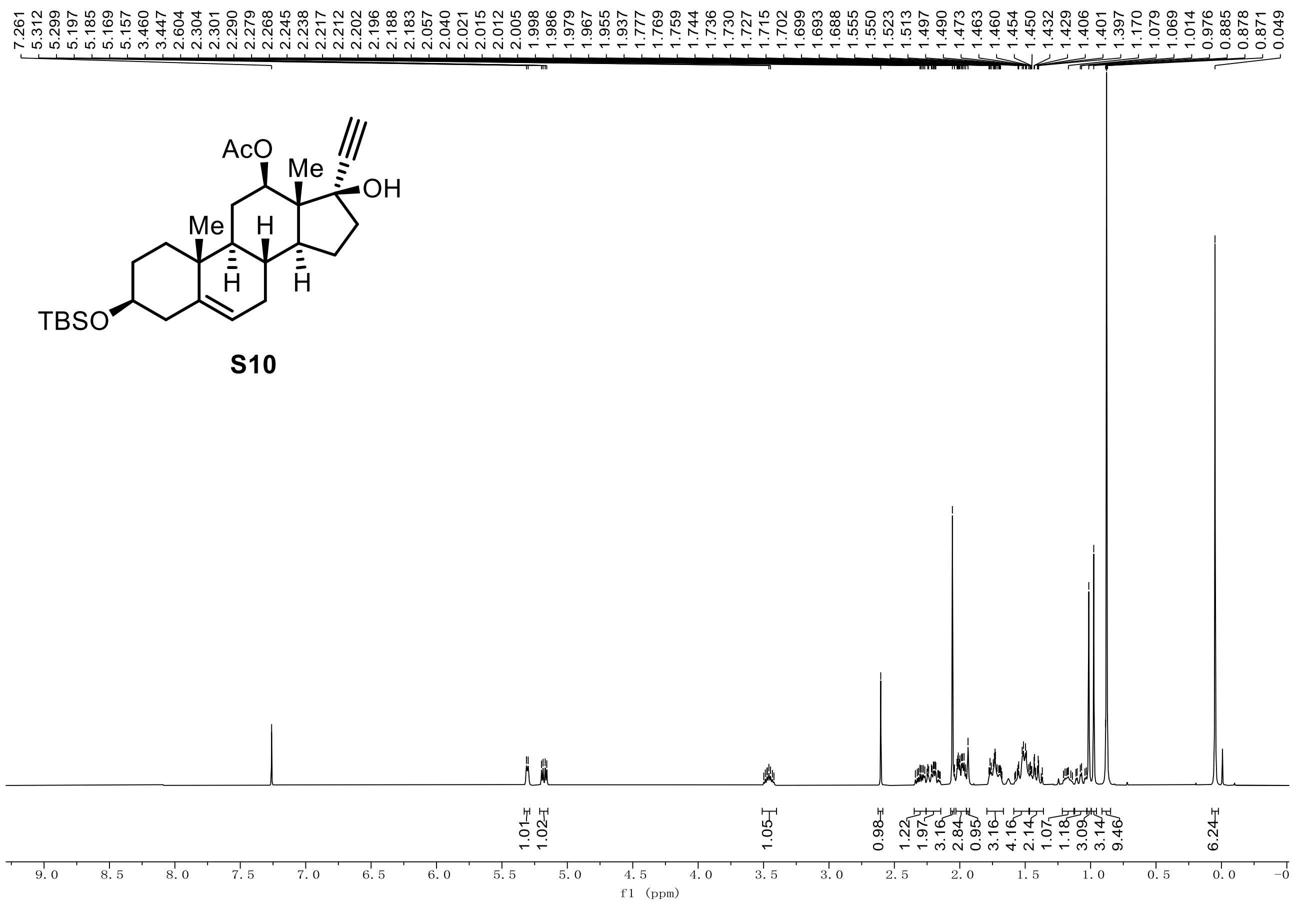

^{13}C NMR of **S10** (100 MHz, $CDCl_3$)

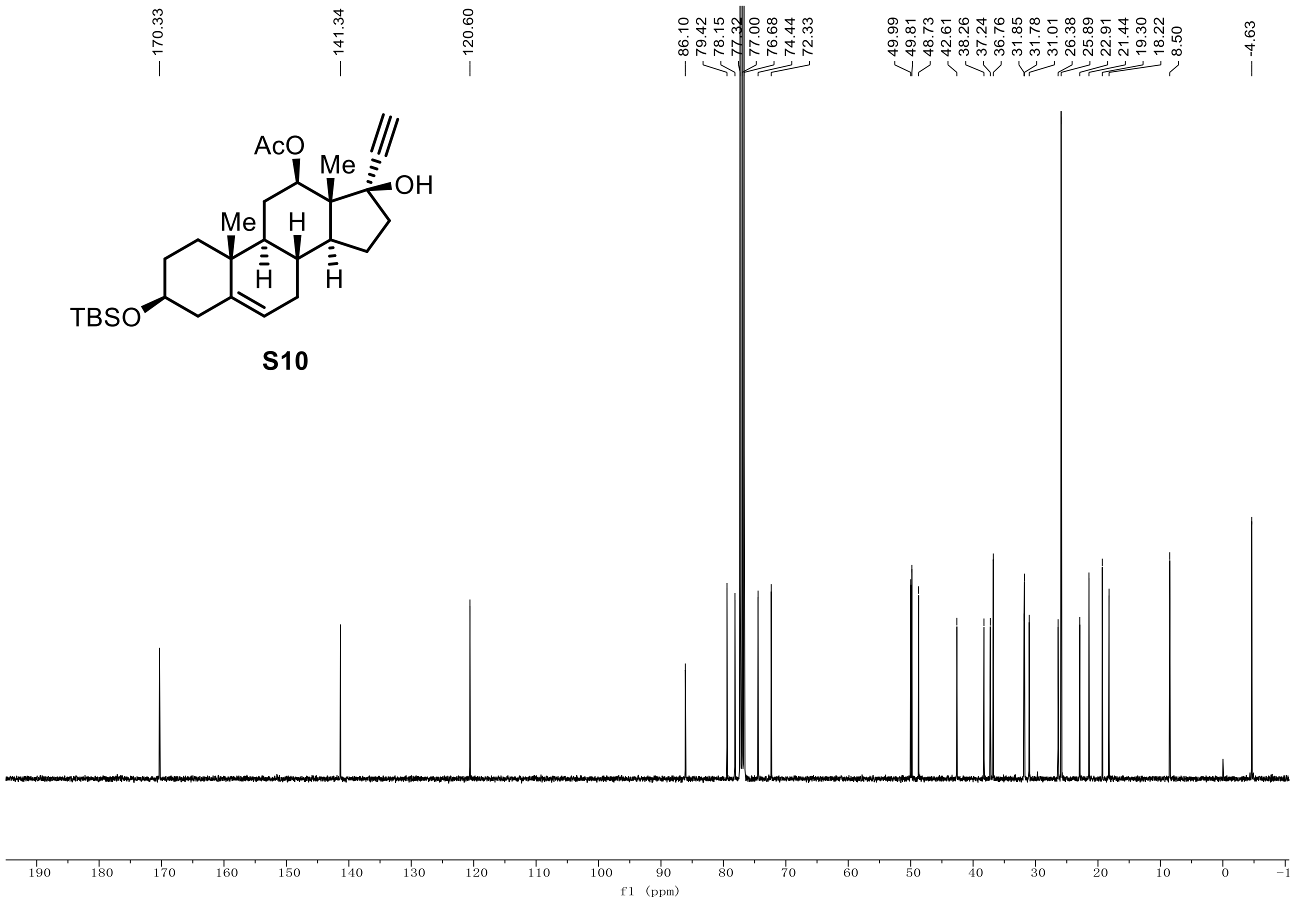

^{1}H NMR of **S11** (400 MHz, $CDCl_3$)

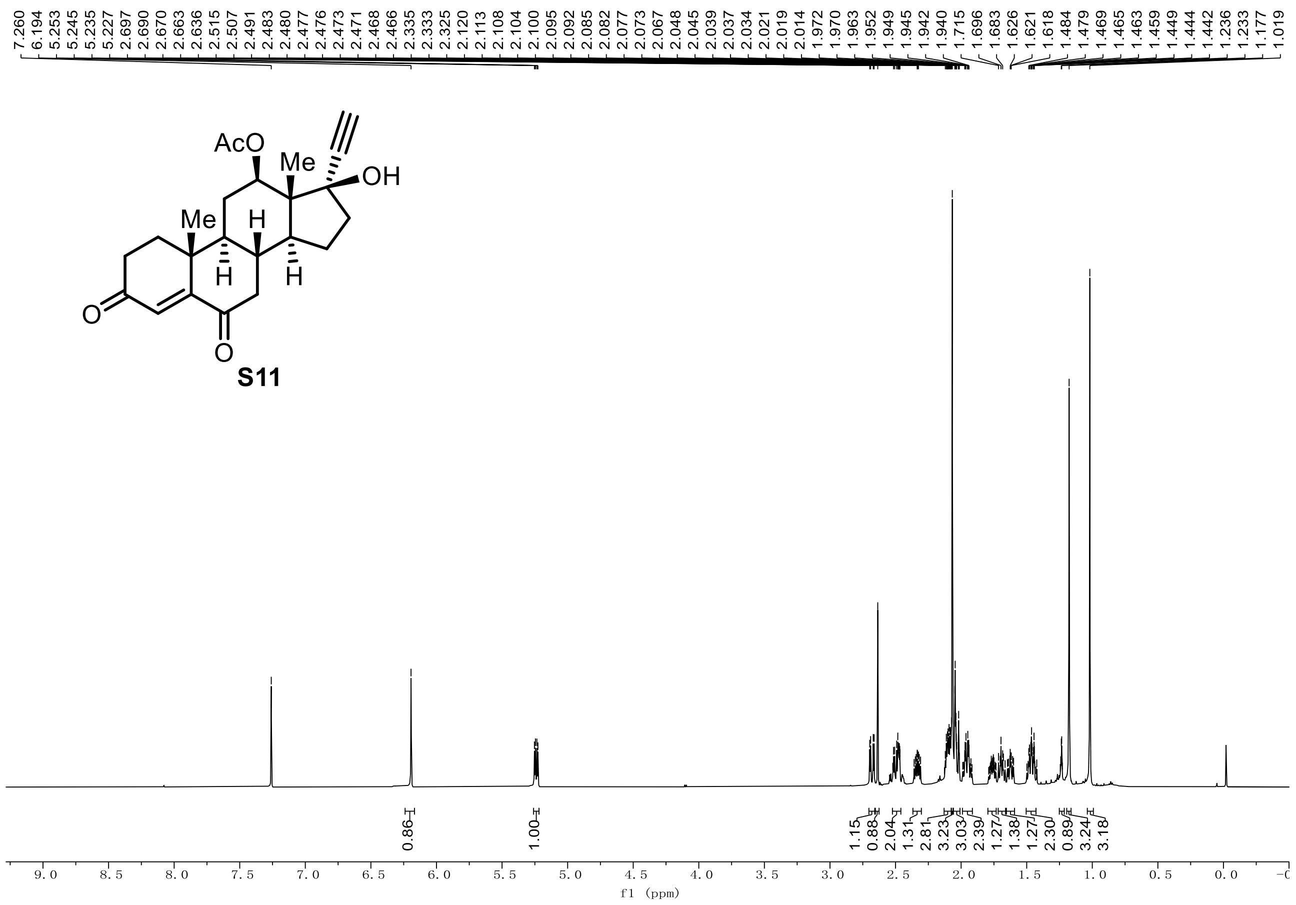

^{13}C NMR of **S11** (100 MHz, $CDCl_3$)

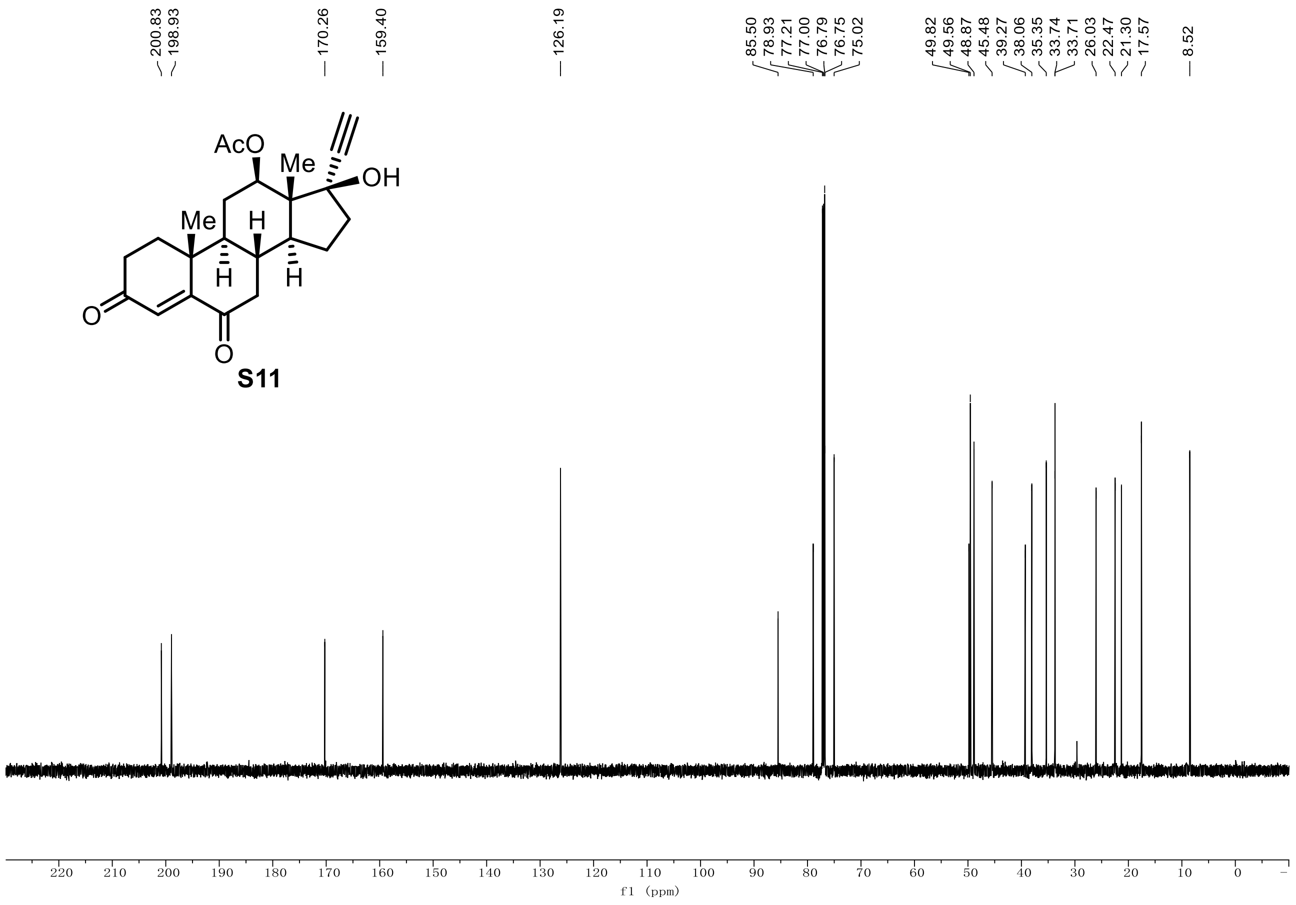

^{1}H NMR of **S12** (400 MHz, $CDCl_3$)

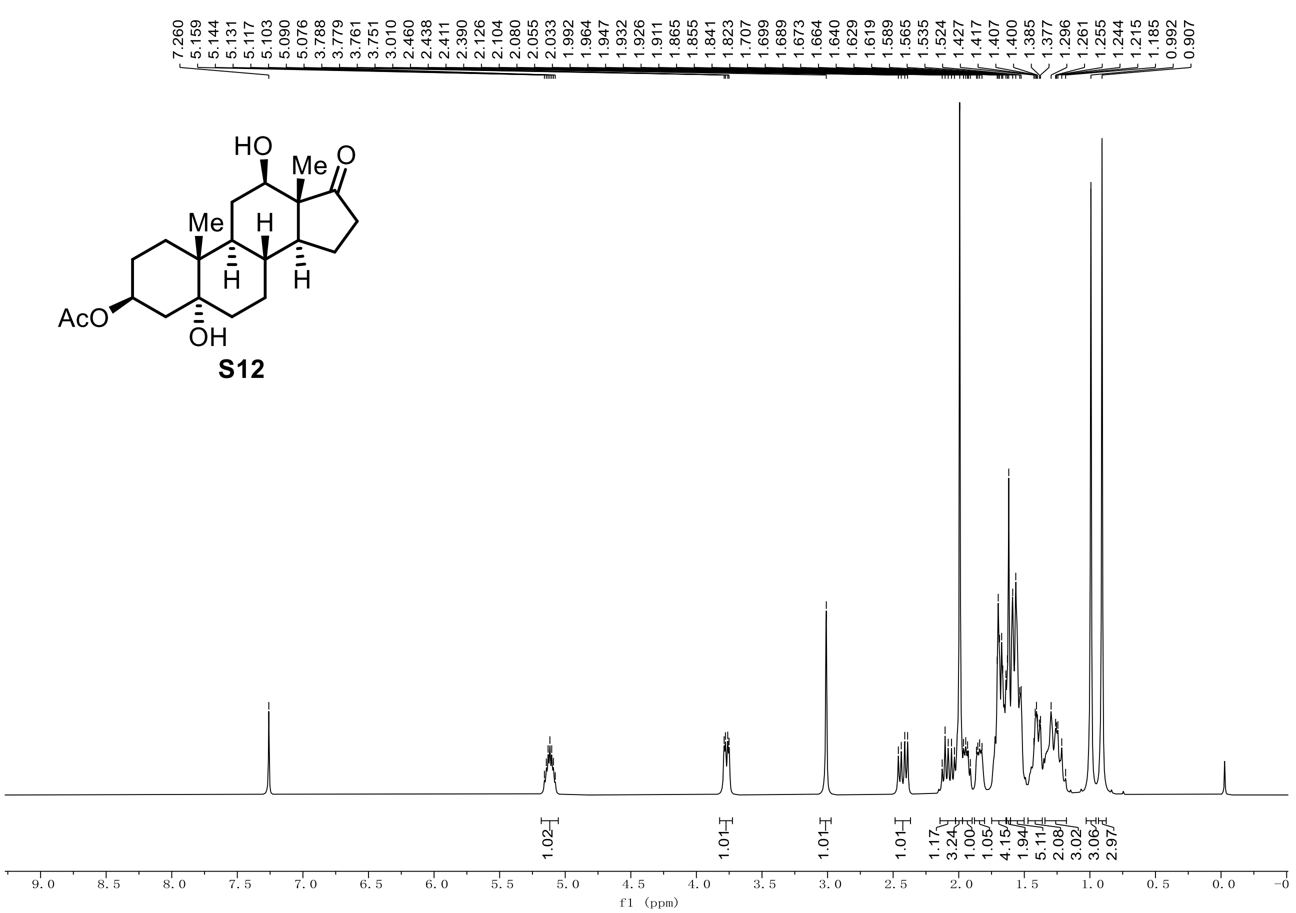

^{13}C NMR of **S12** (150 MHz, $CDCl_3$)

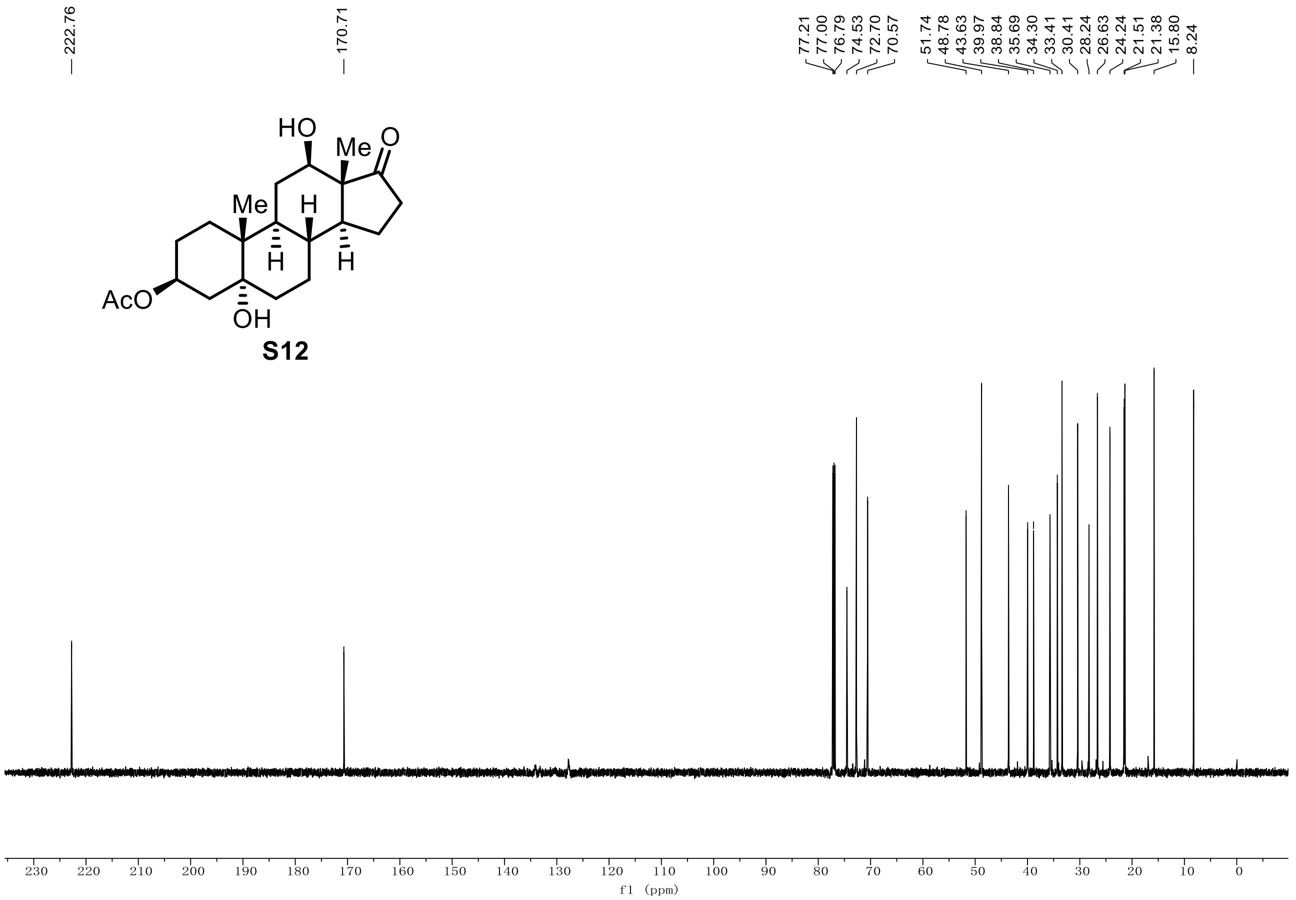

^{1}H NMR of **S15** (400 MHz, $CDCl_3$)

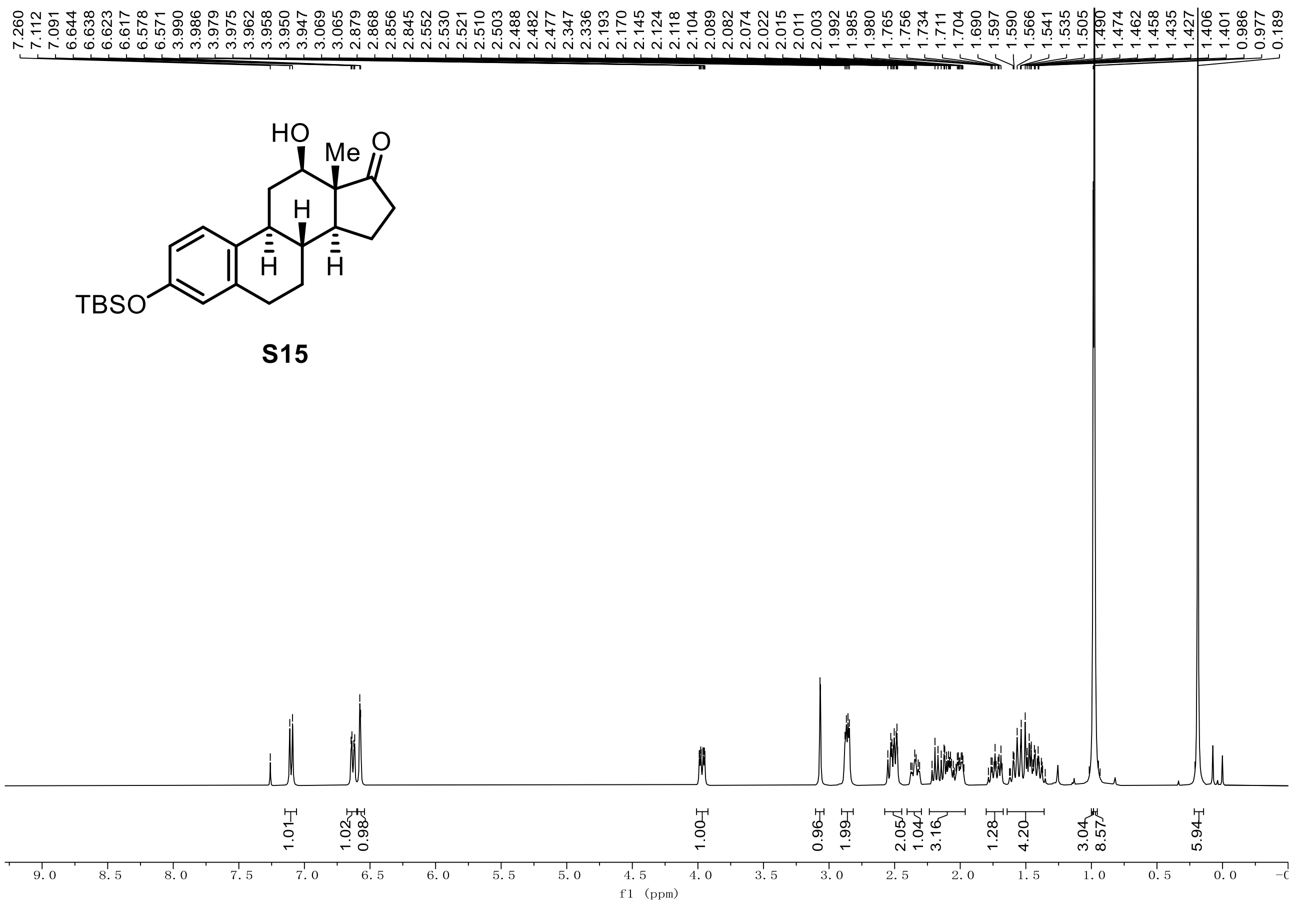

^{13}C NMR of **S15** (100 MHz, $CDCl_3$)

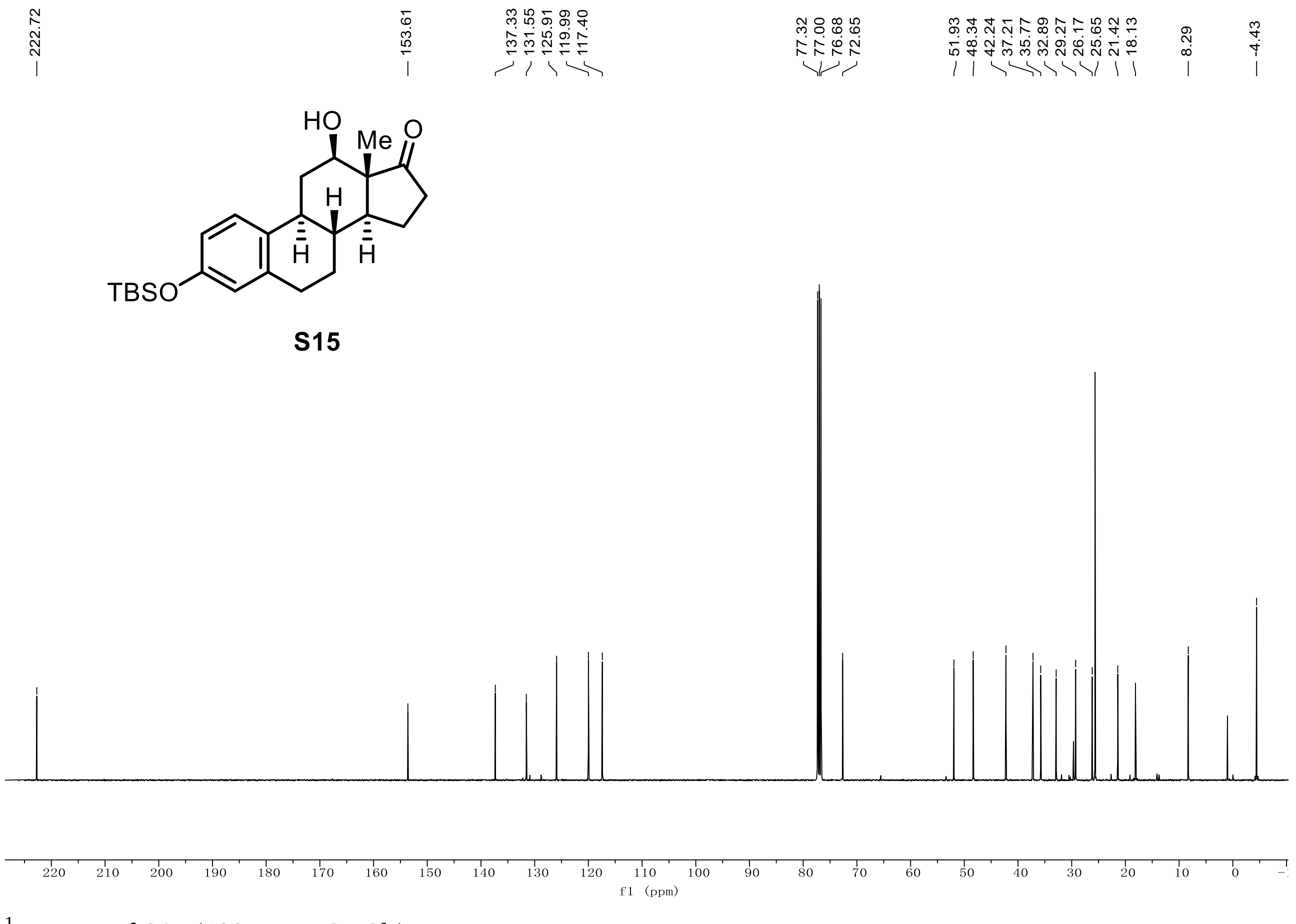

^{1}H NMR of **S17** (400 MHz, $CDCl_3$)

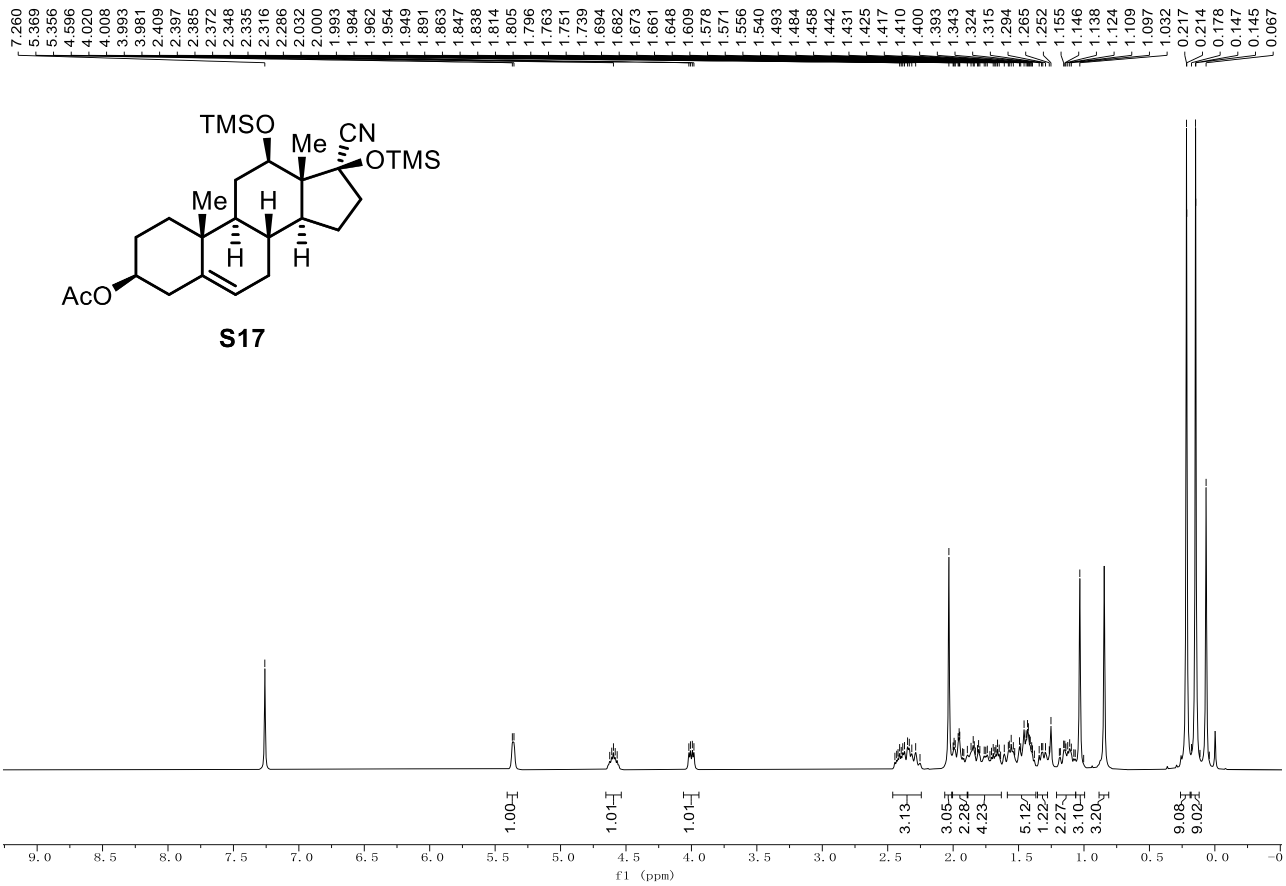

^{13}C NMR of **S17** (100 MHz, $CDCl_3$)

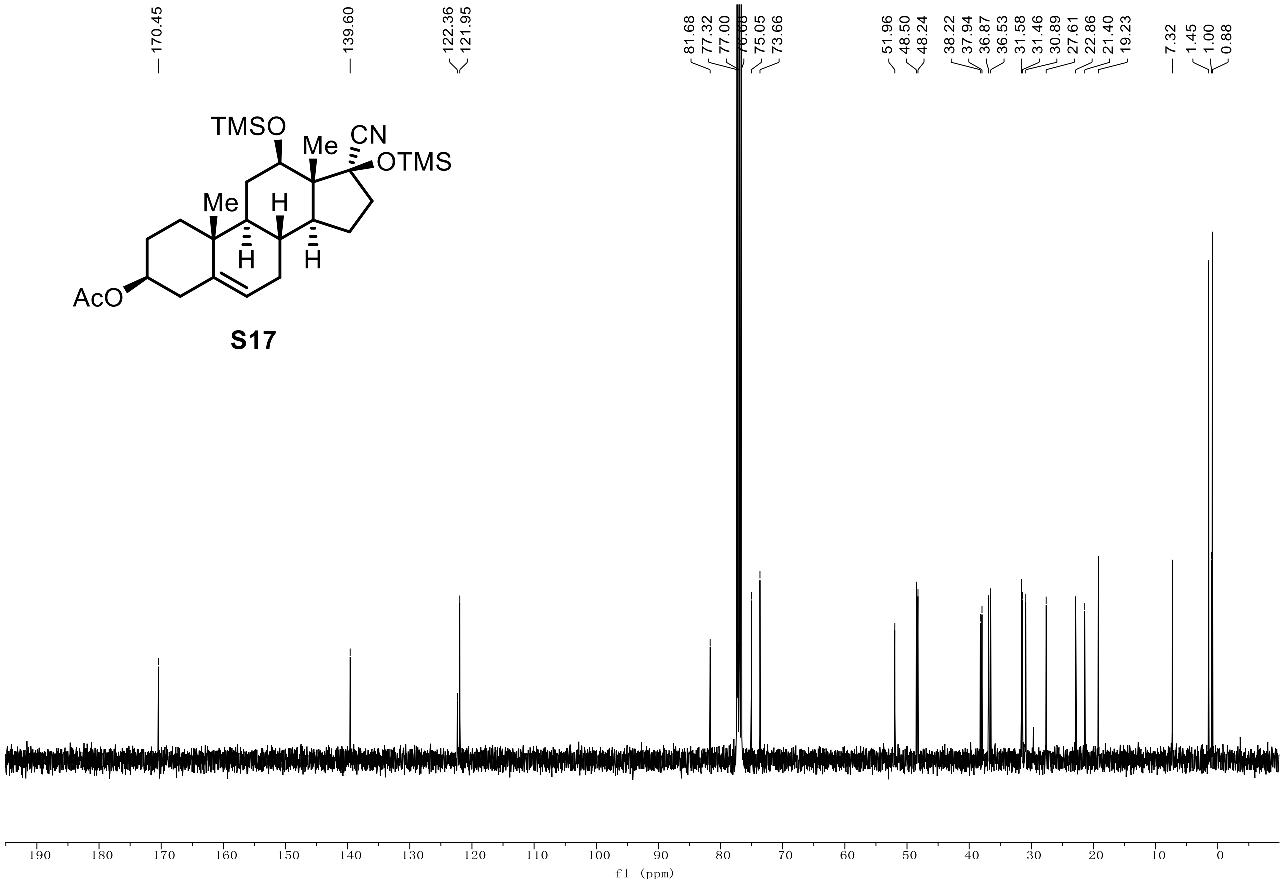

^{1}H NMR of **32b** (400 MHz, $CDCl_3$)

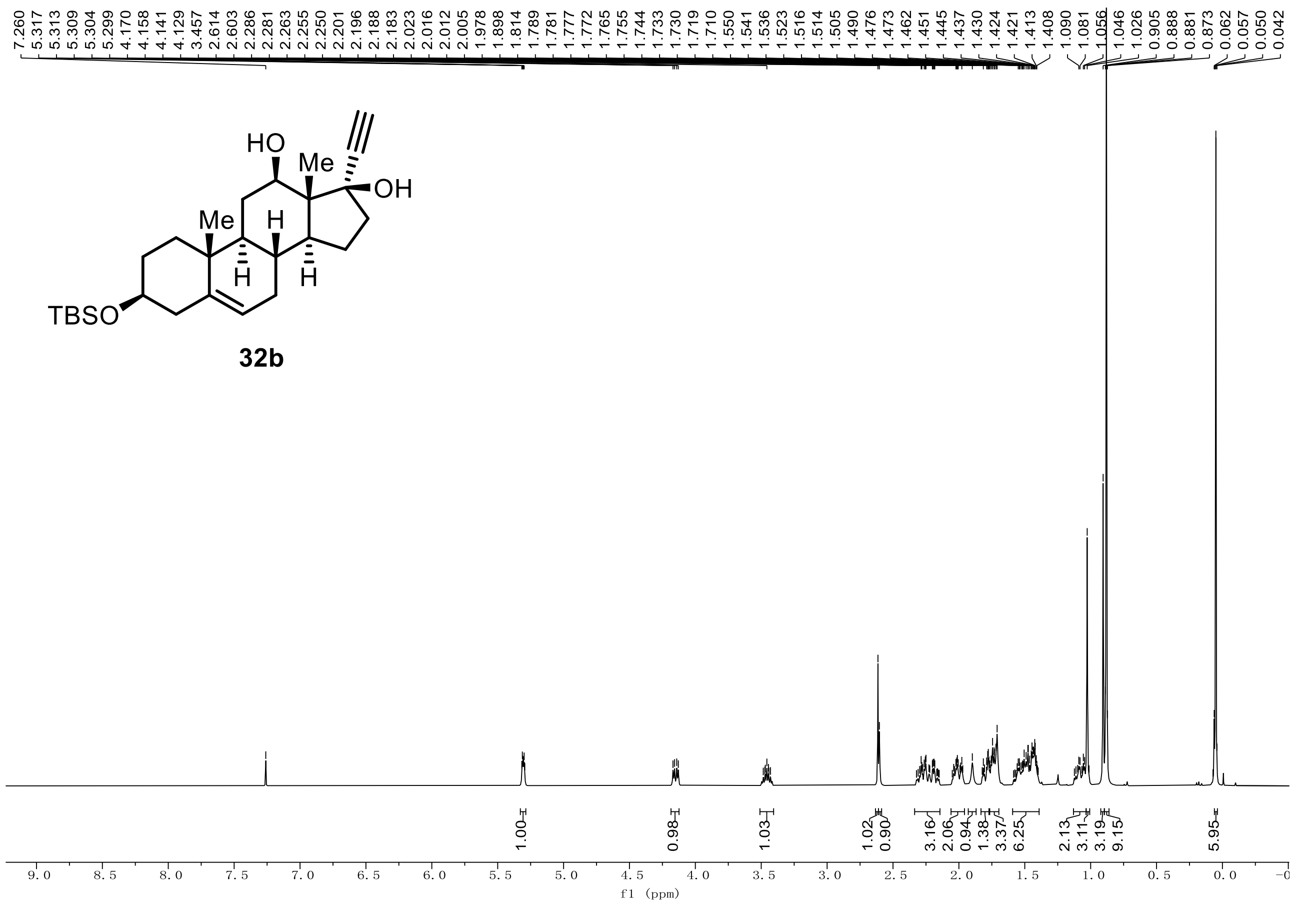

^{13}C NMR of **32b** (100 MHz, $CDCl_3$)

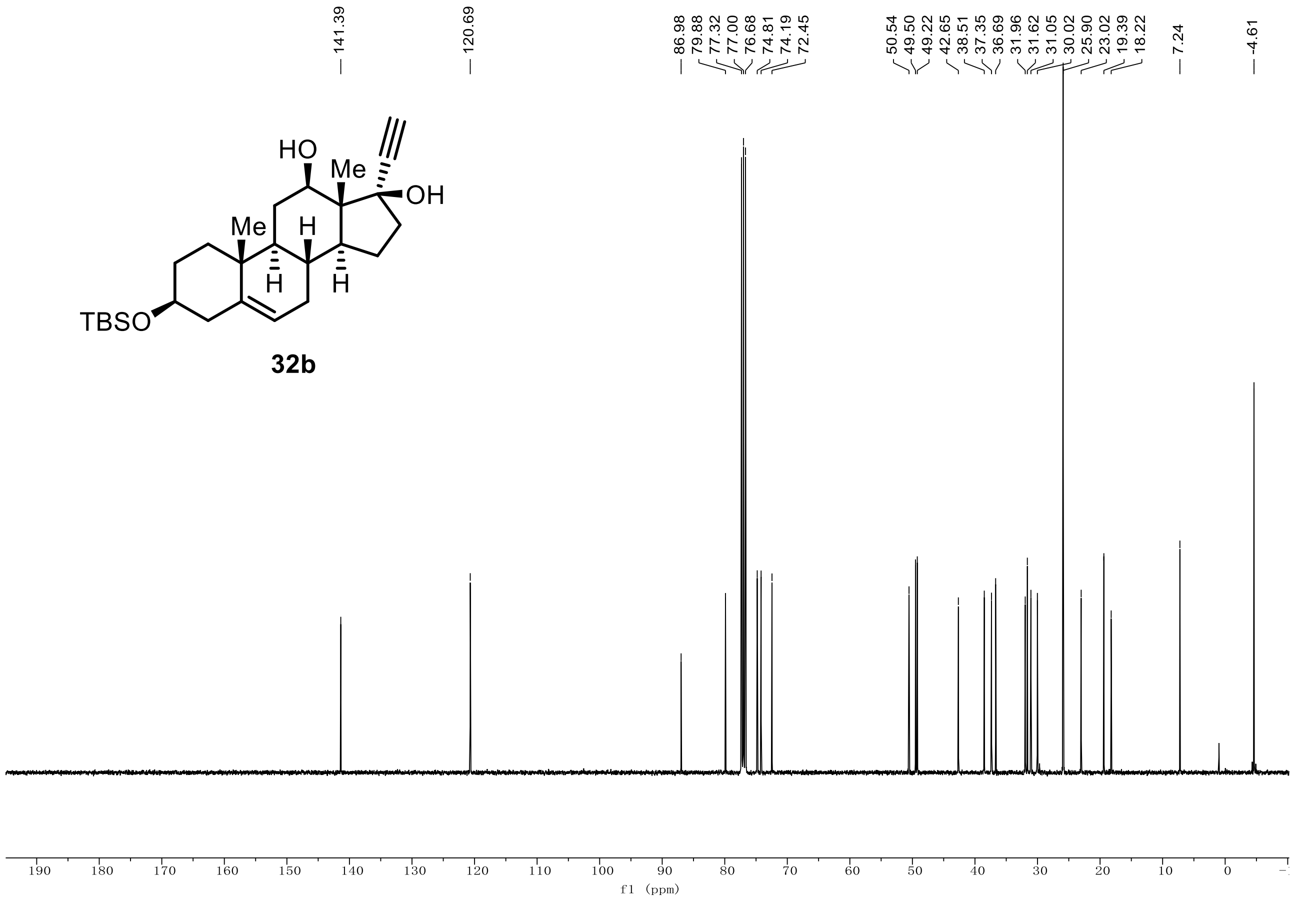

^{1}H NMR of **32c** (400 MHz, $CDCl_3$)

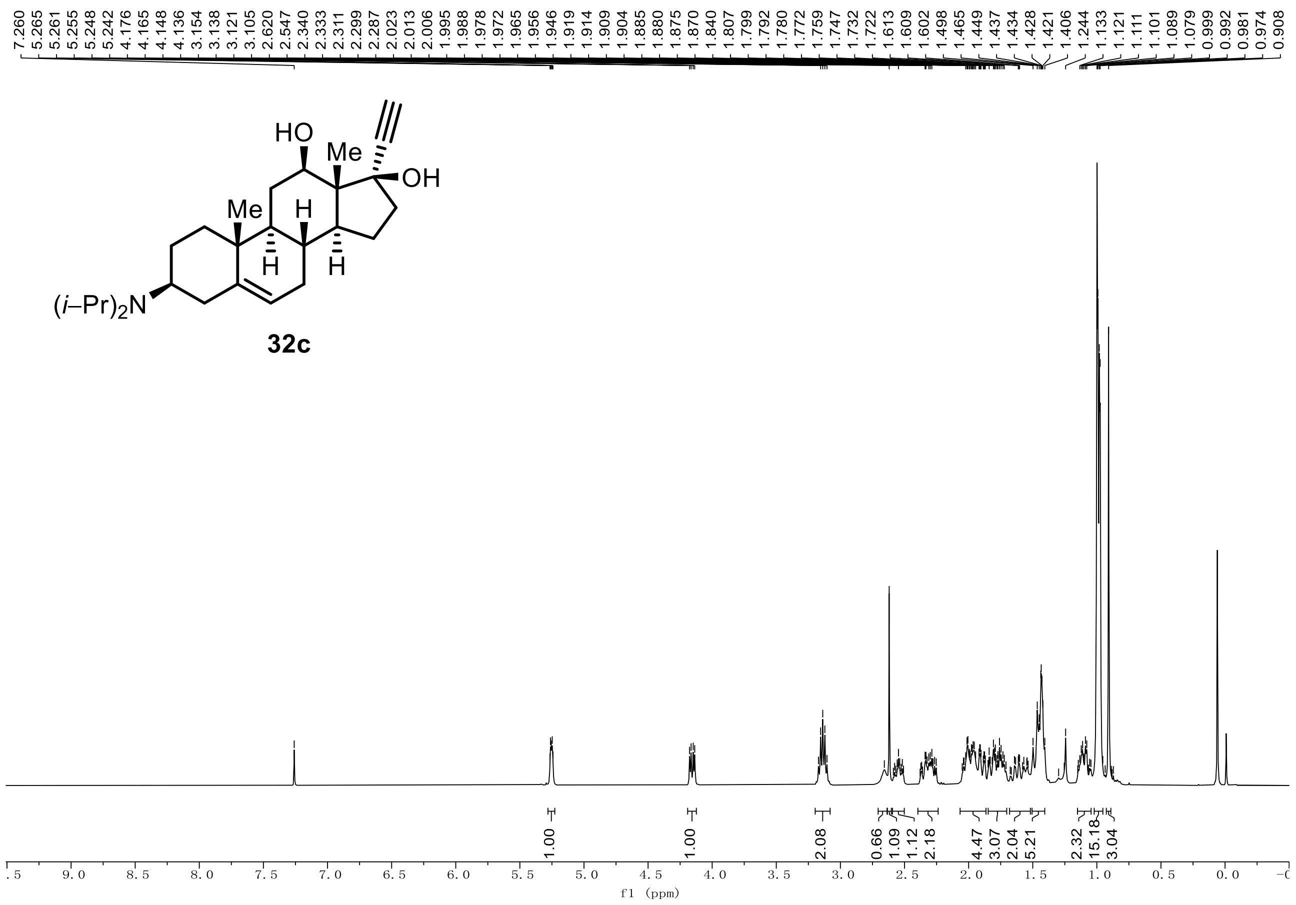

^{13}C NMR of **32c** (100 MHz, $CDCl_3$)

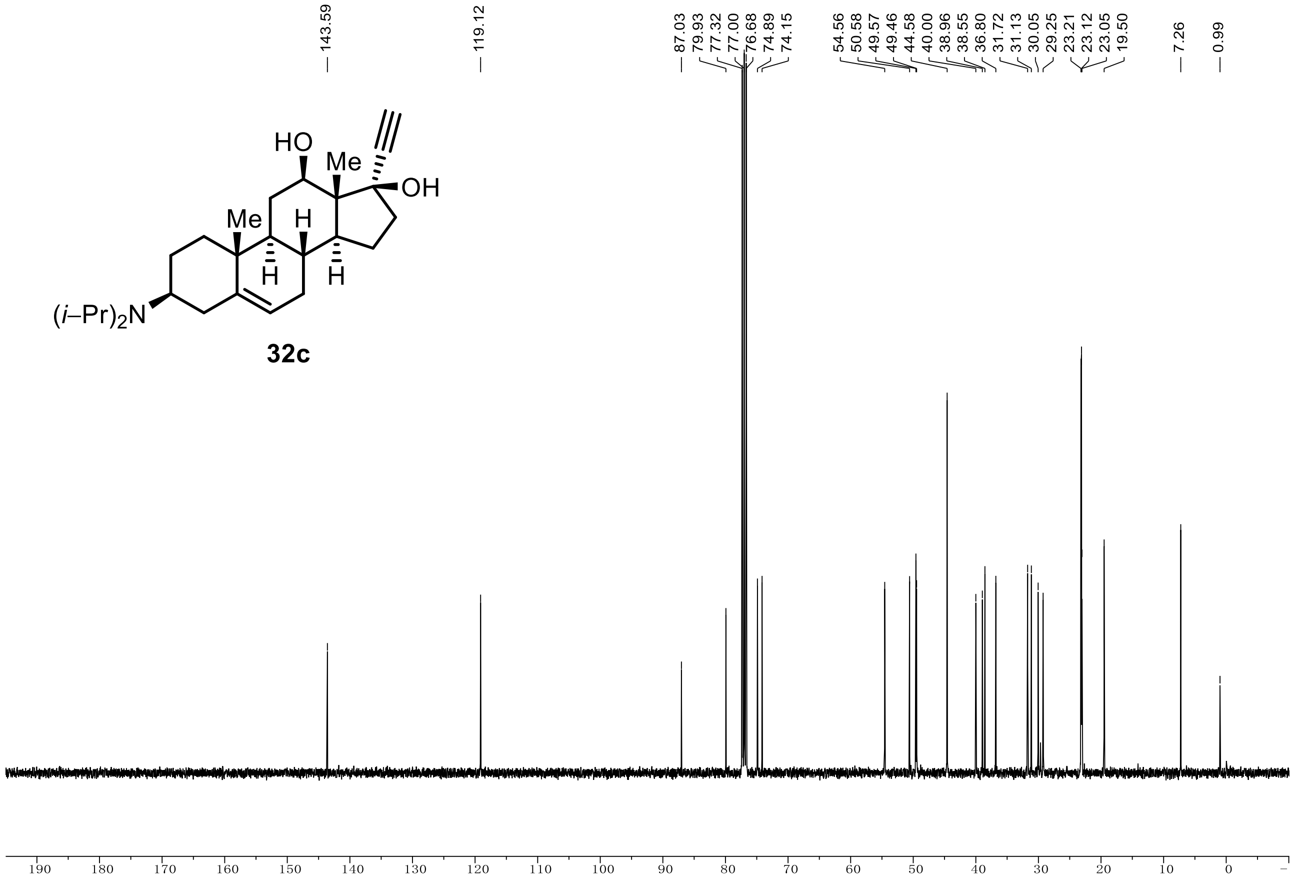

^{1}H NMR of **32d** (400 MHz, $CDCl_3$)

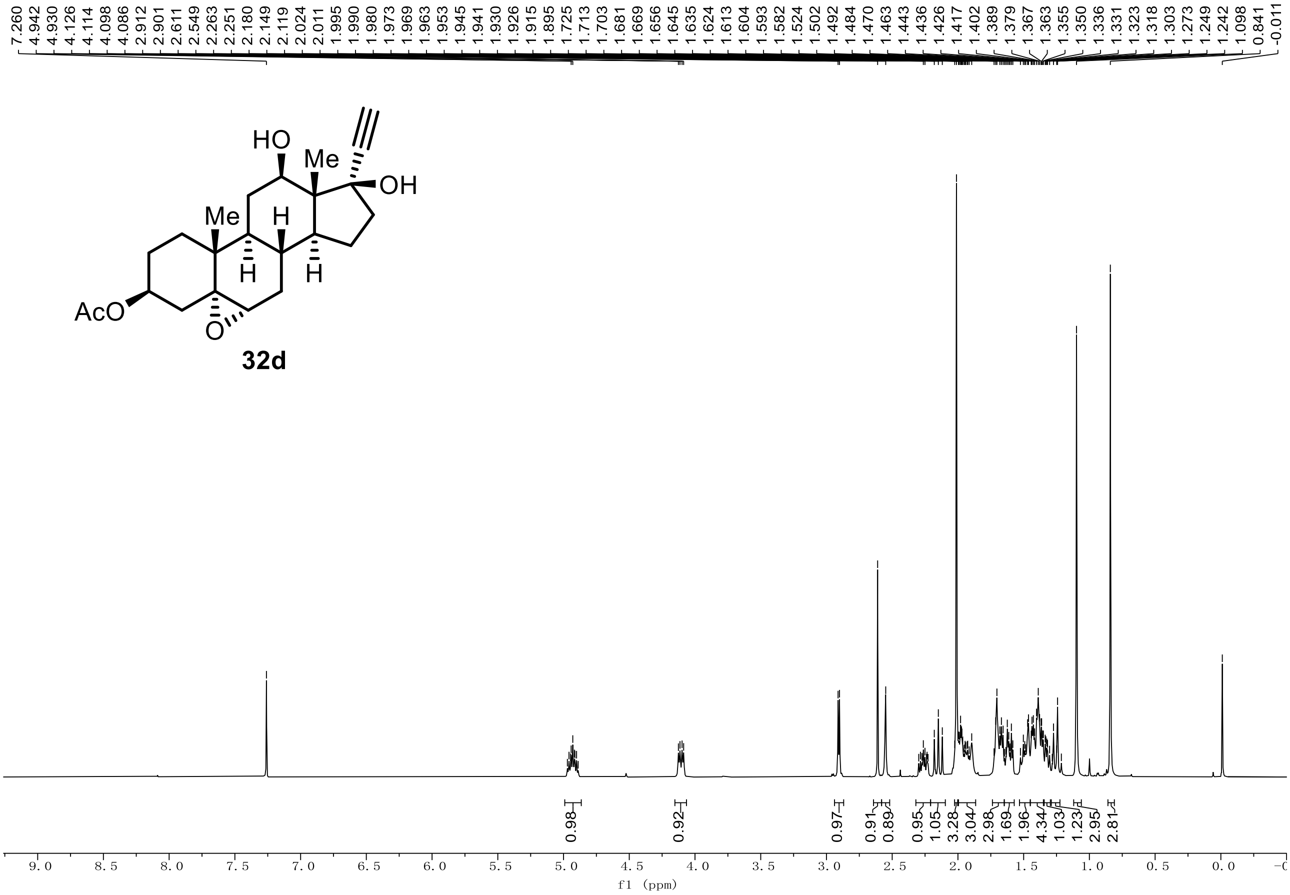

^{13}C NMR of **32d** (100 MHz, $CDCl_3$)

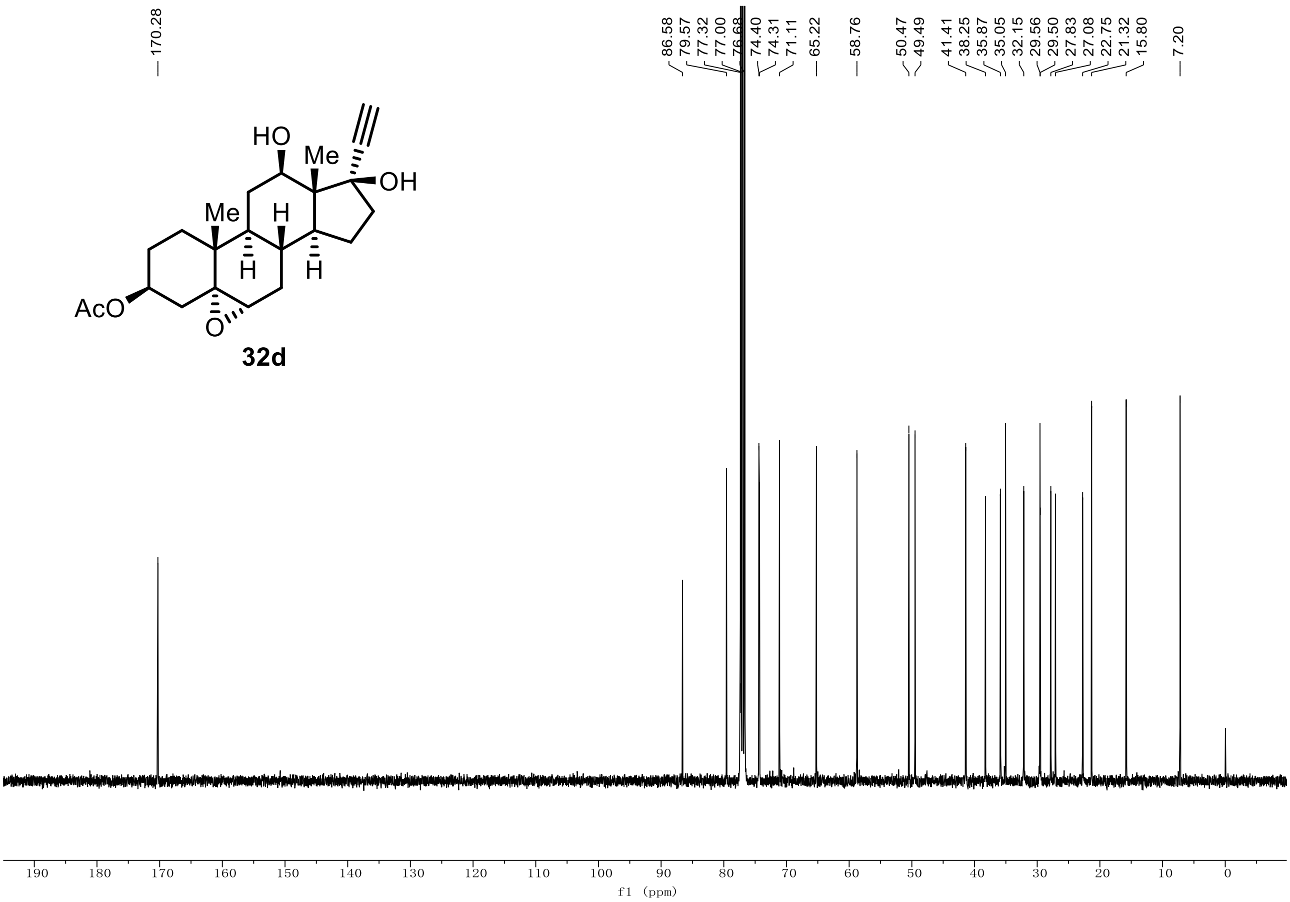

^{1}H NMR of **32e** (400 MHz, $CDCl_3$)

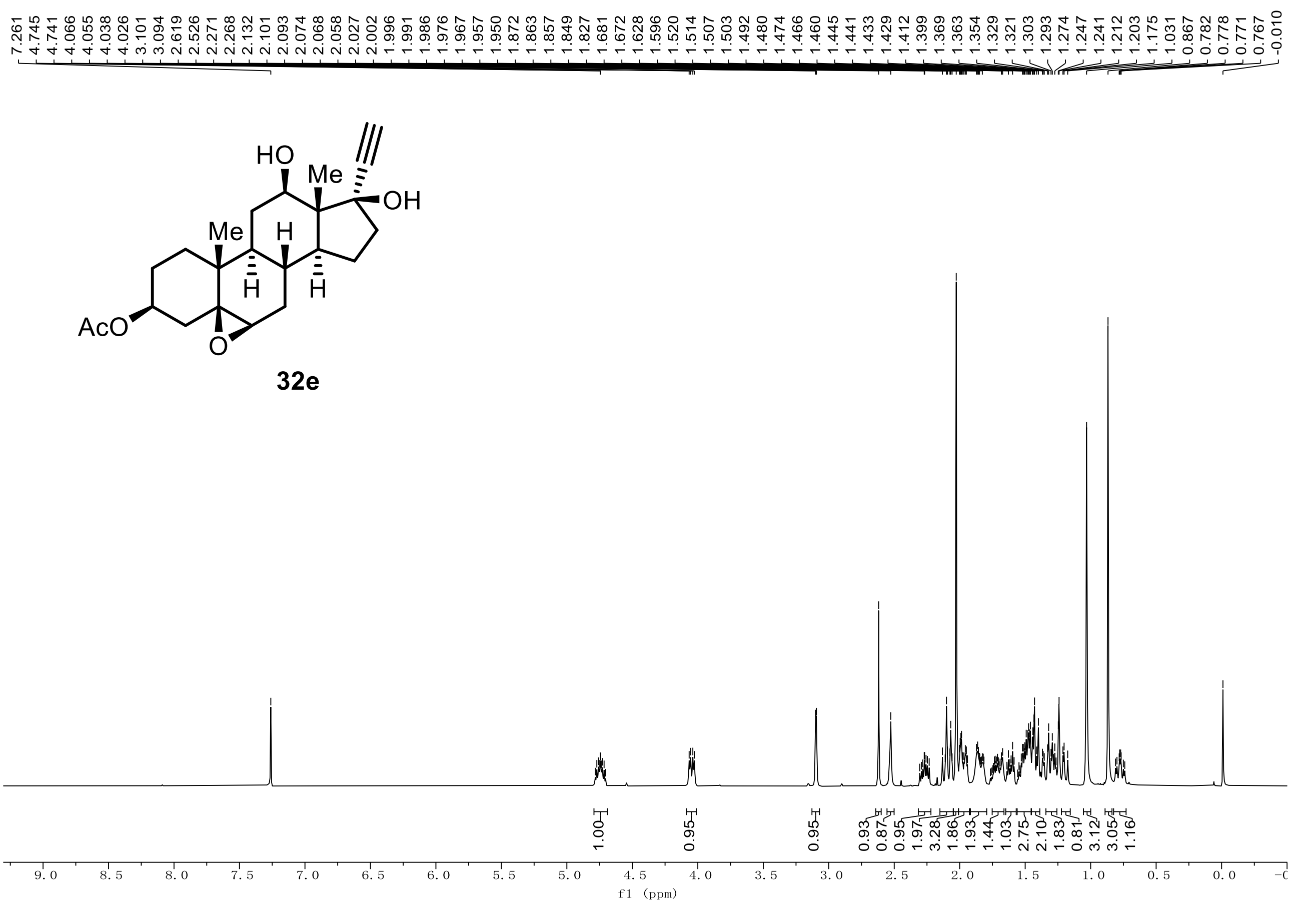

^{13}C NMR of **32e** (100 MHz, $CDCl_3$)

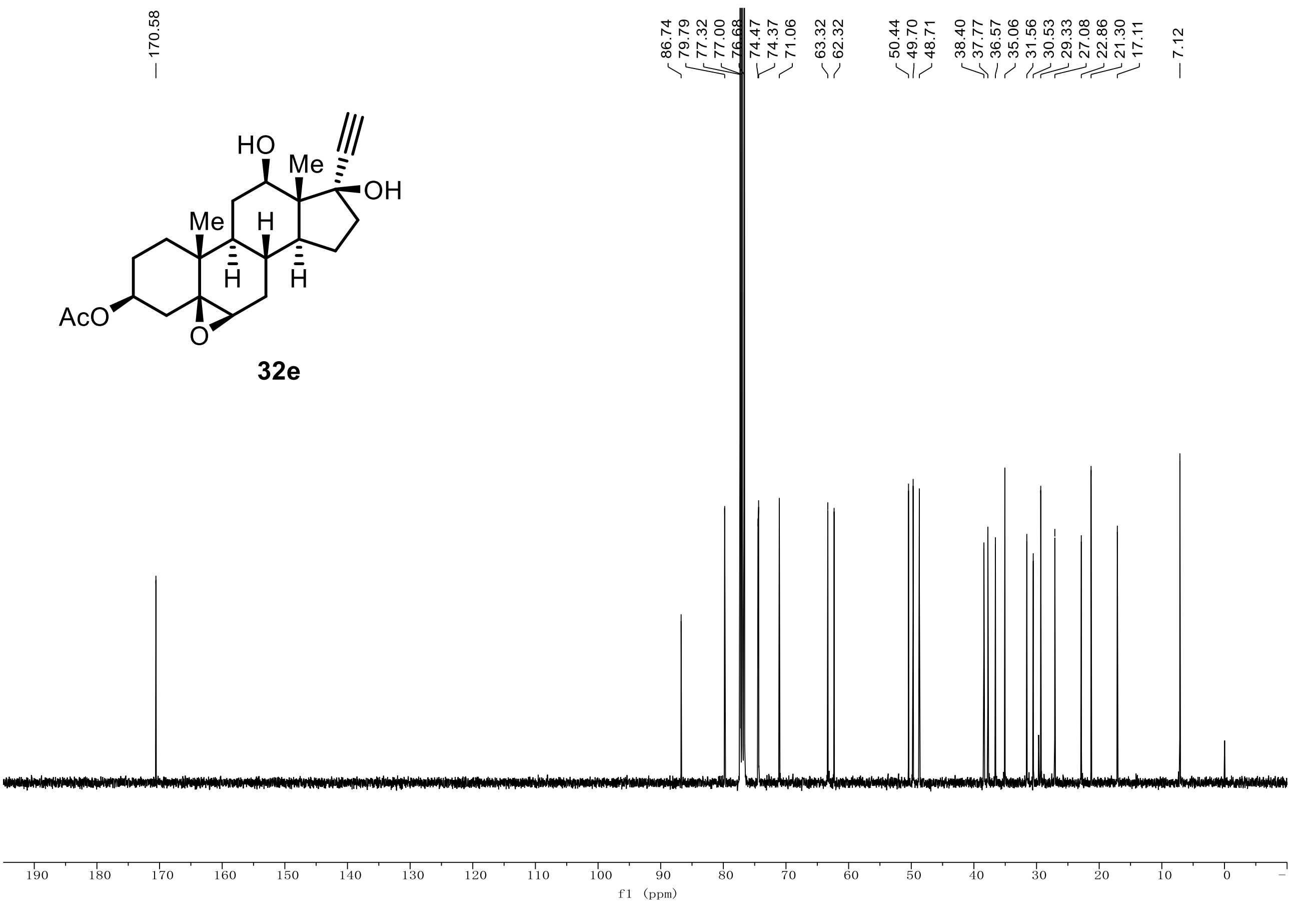

^{1}H NMR of **32f** (400 MHz, CD_3OD)

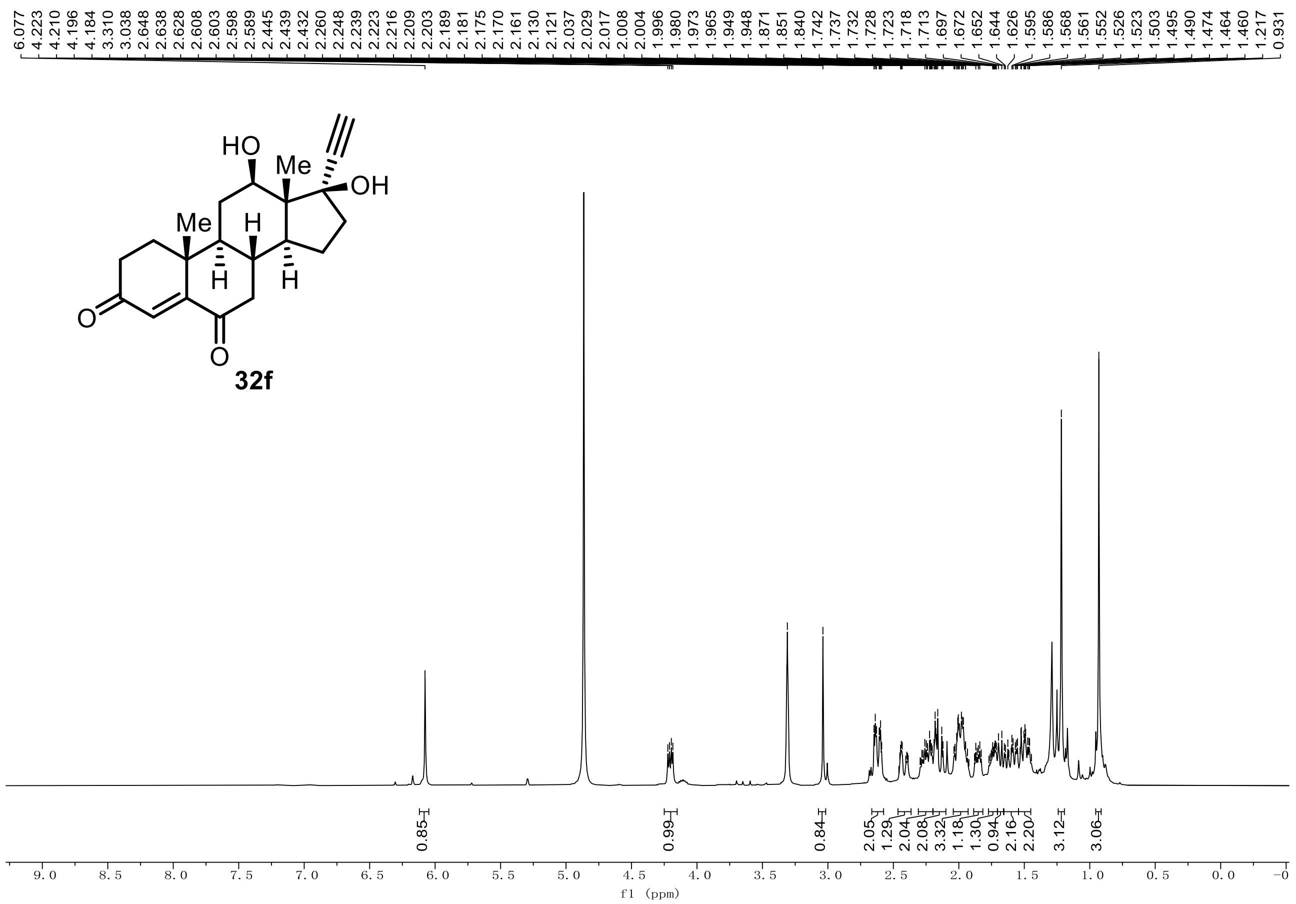

^{13}C NMR of **32f** (100 MHz, CD_3OD)

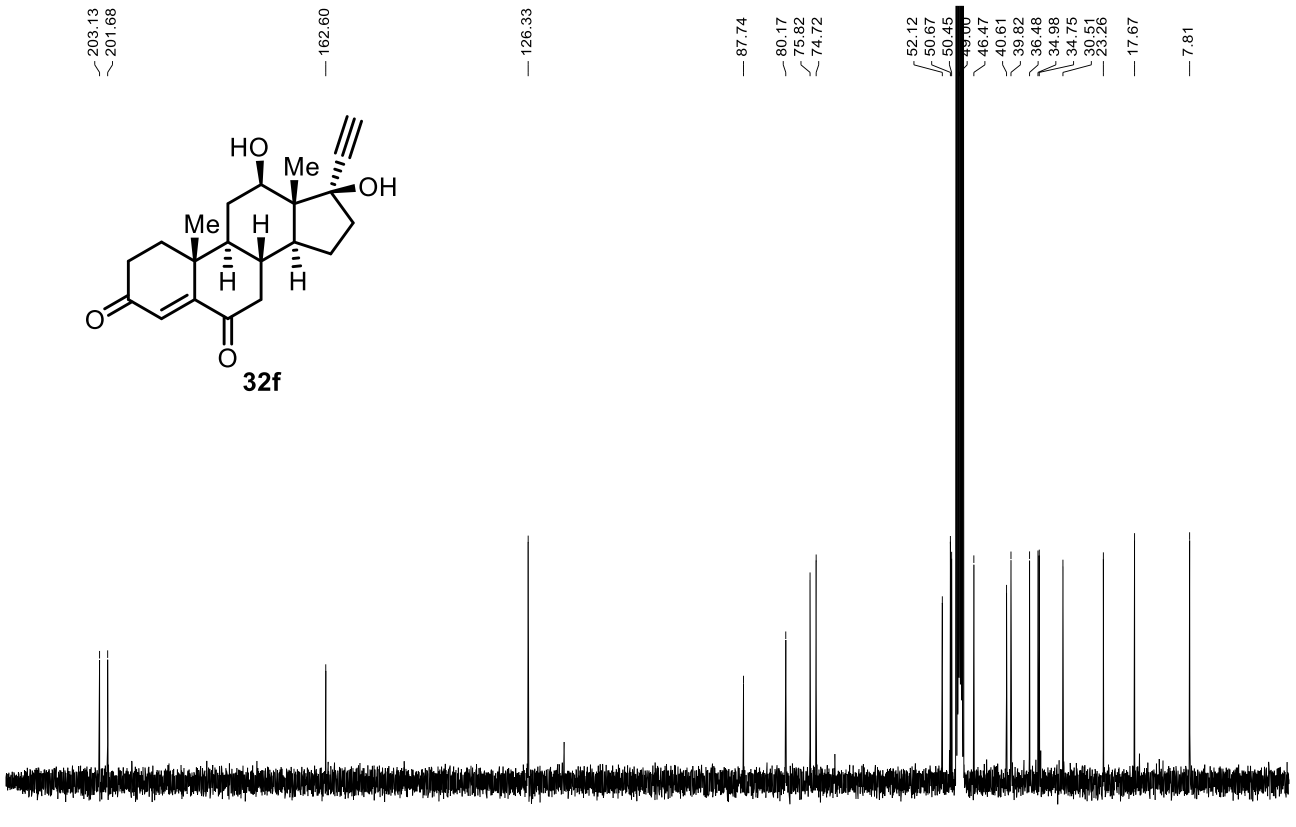

^{1}H NMR of **32g** (400 MHz, C_5D_5N)

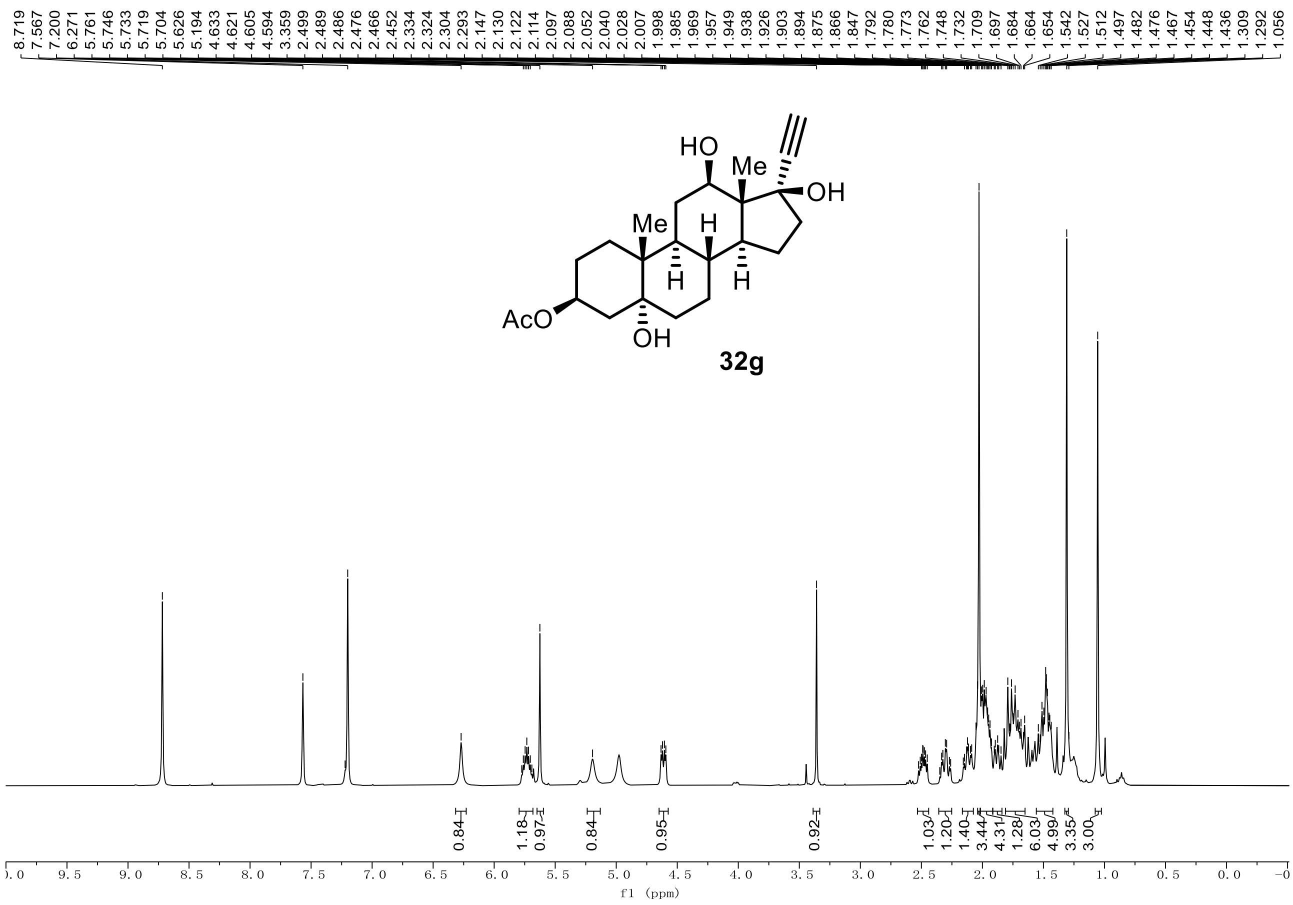

^{13}C NMR of **32g** (100 MHz, C_5D_5N)

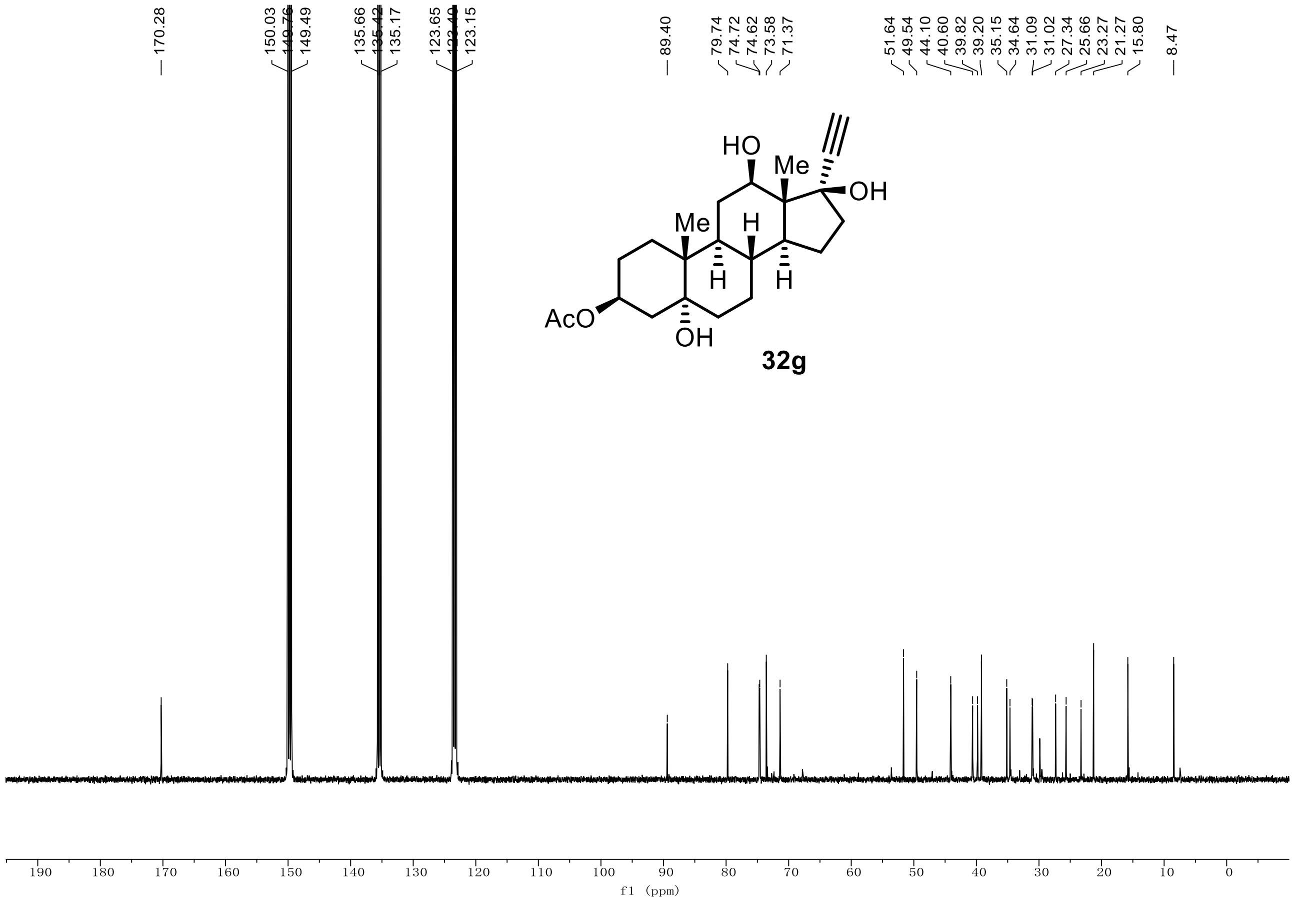

^{1}H NMR of **32h** (400 MHz, $CDCl_3$)

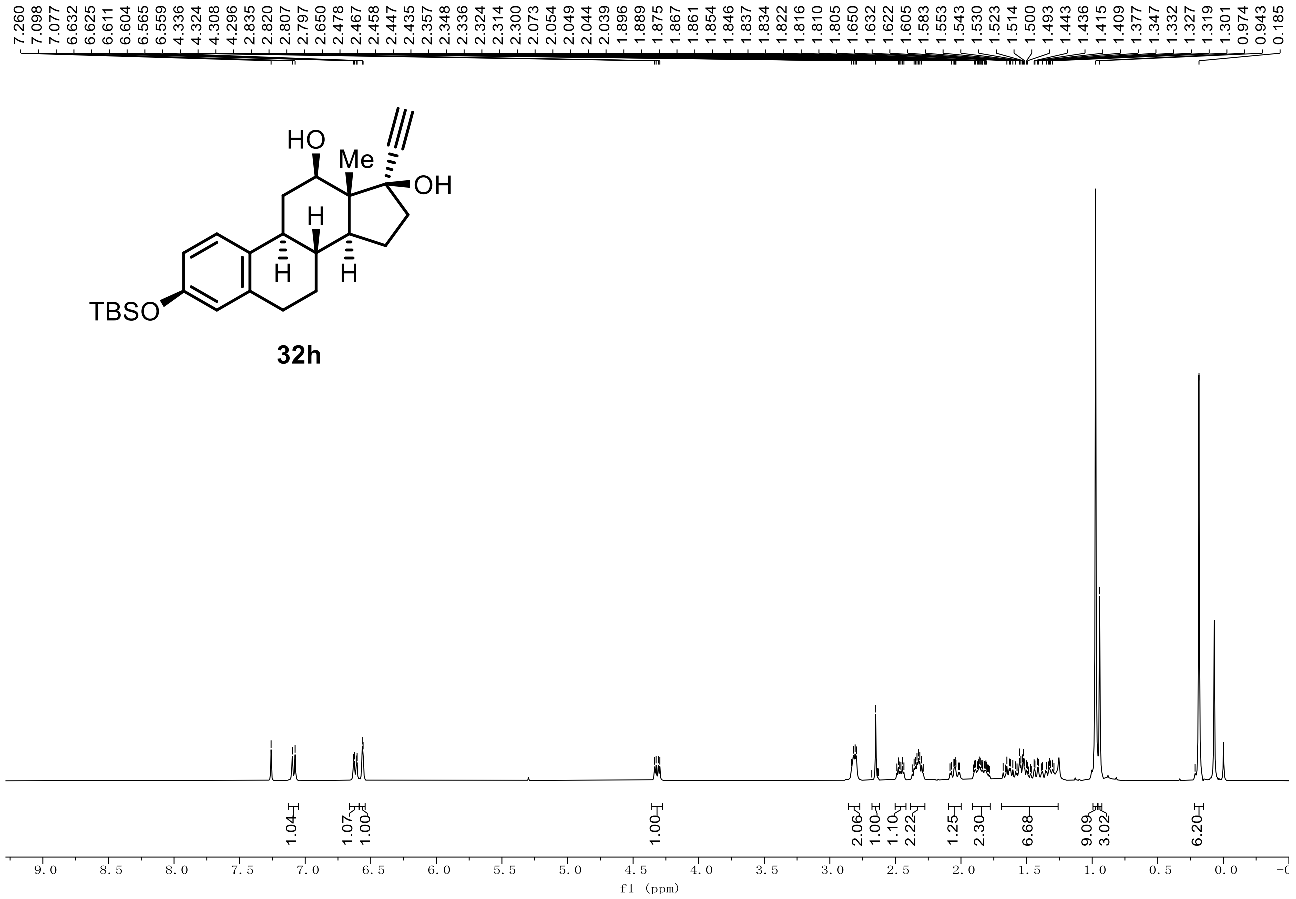

^{13}C NMR of **32h** (100 MHz, $CDCl_3$)

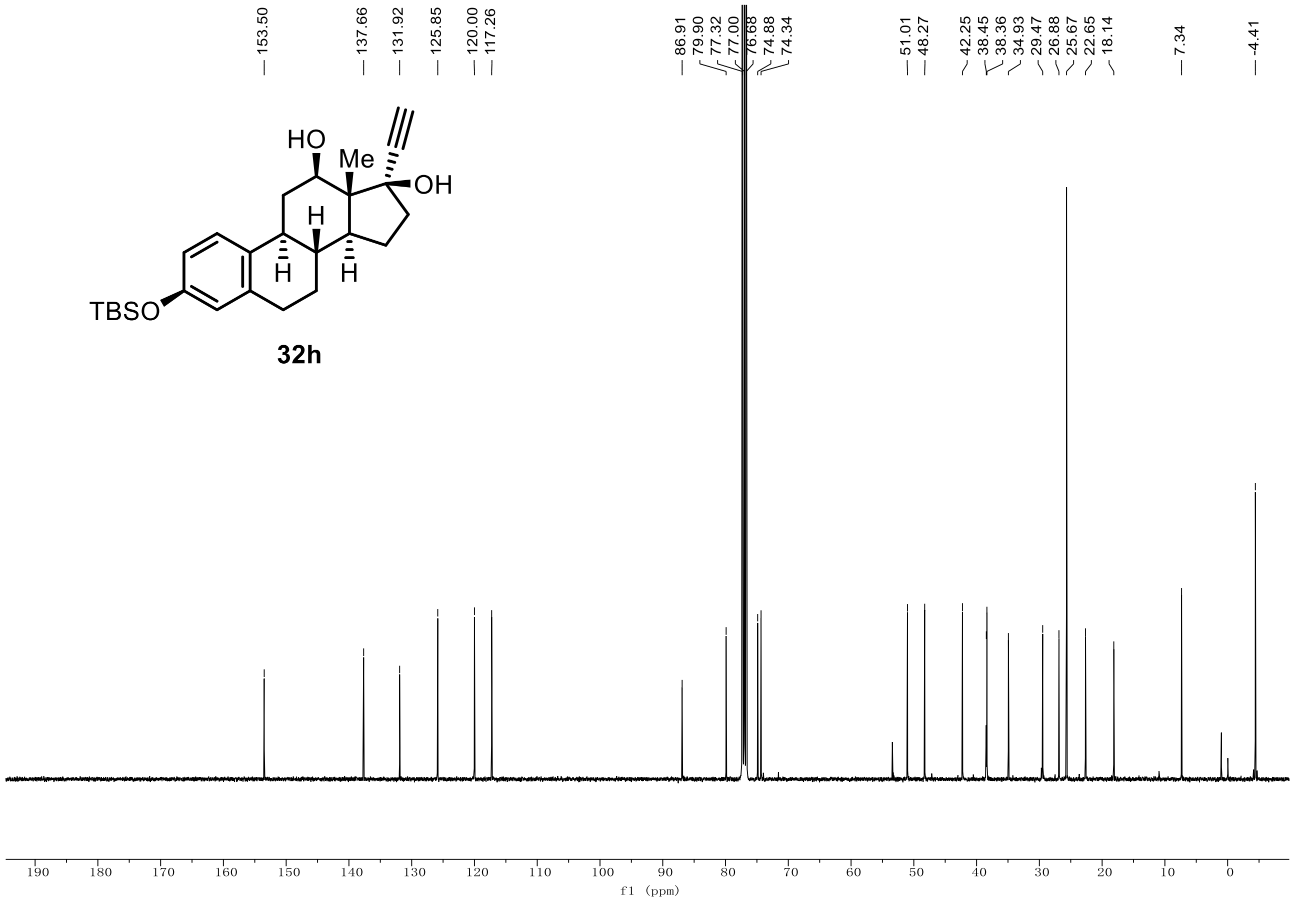

^{1}H NMR of **32i** (400 MHz, $CDCl_3$)

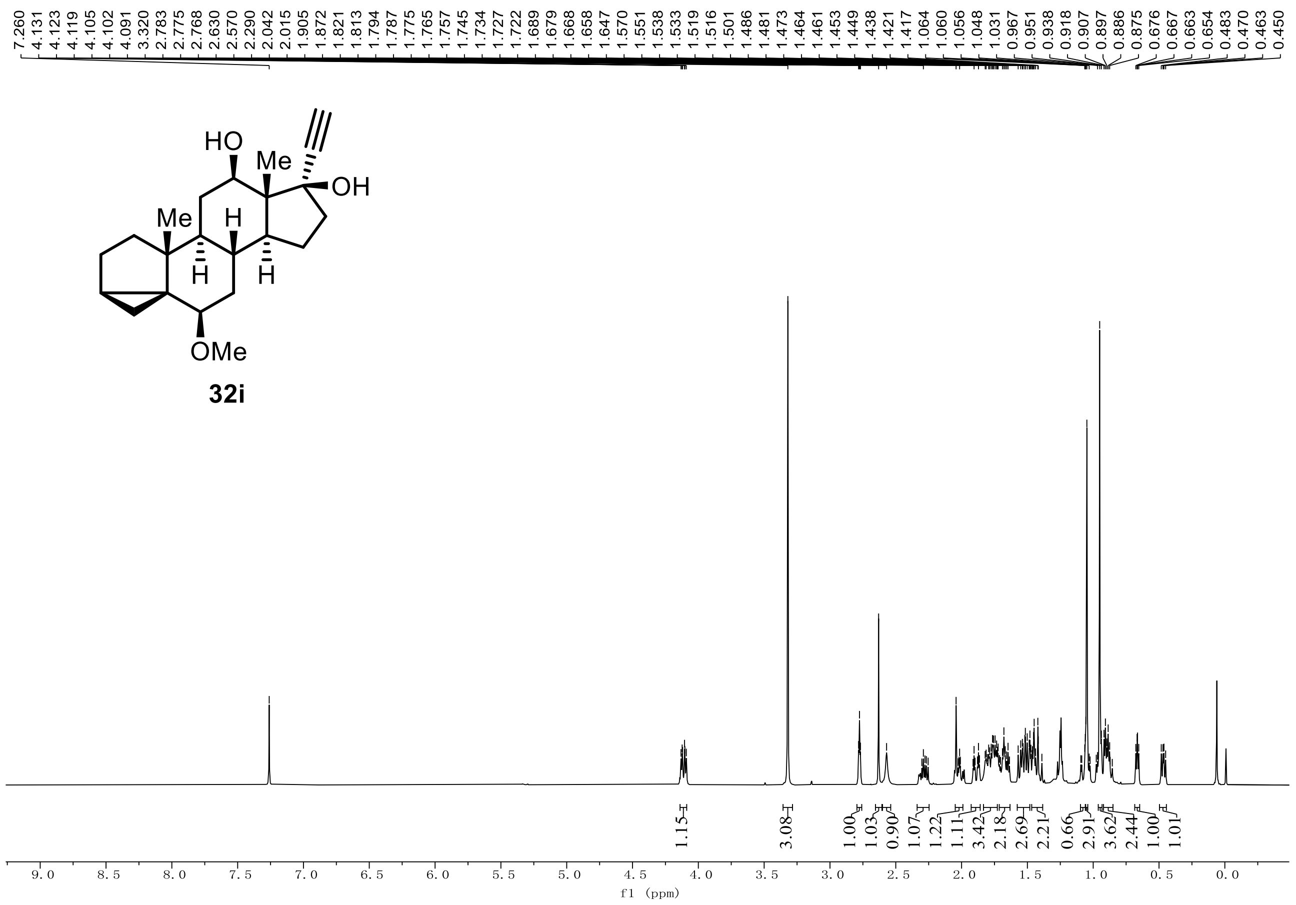

^{13}C NMR of **32i** (100 MHz, $CDCl_3$)

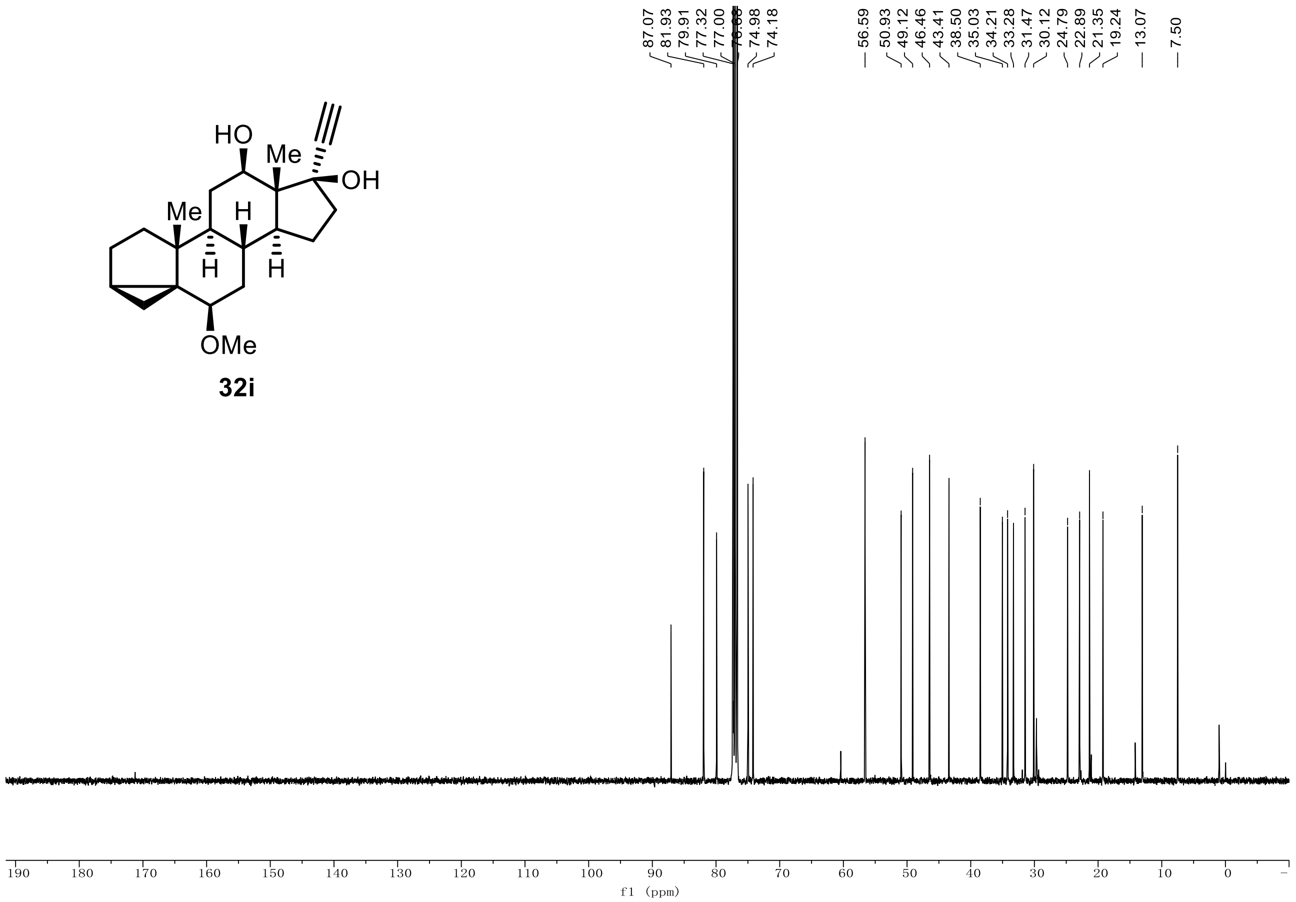

^{1}H NMR of **32j** (400 MHz, $CDCl_3$)

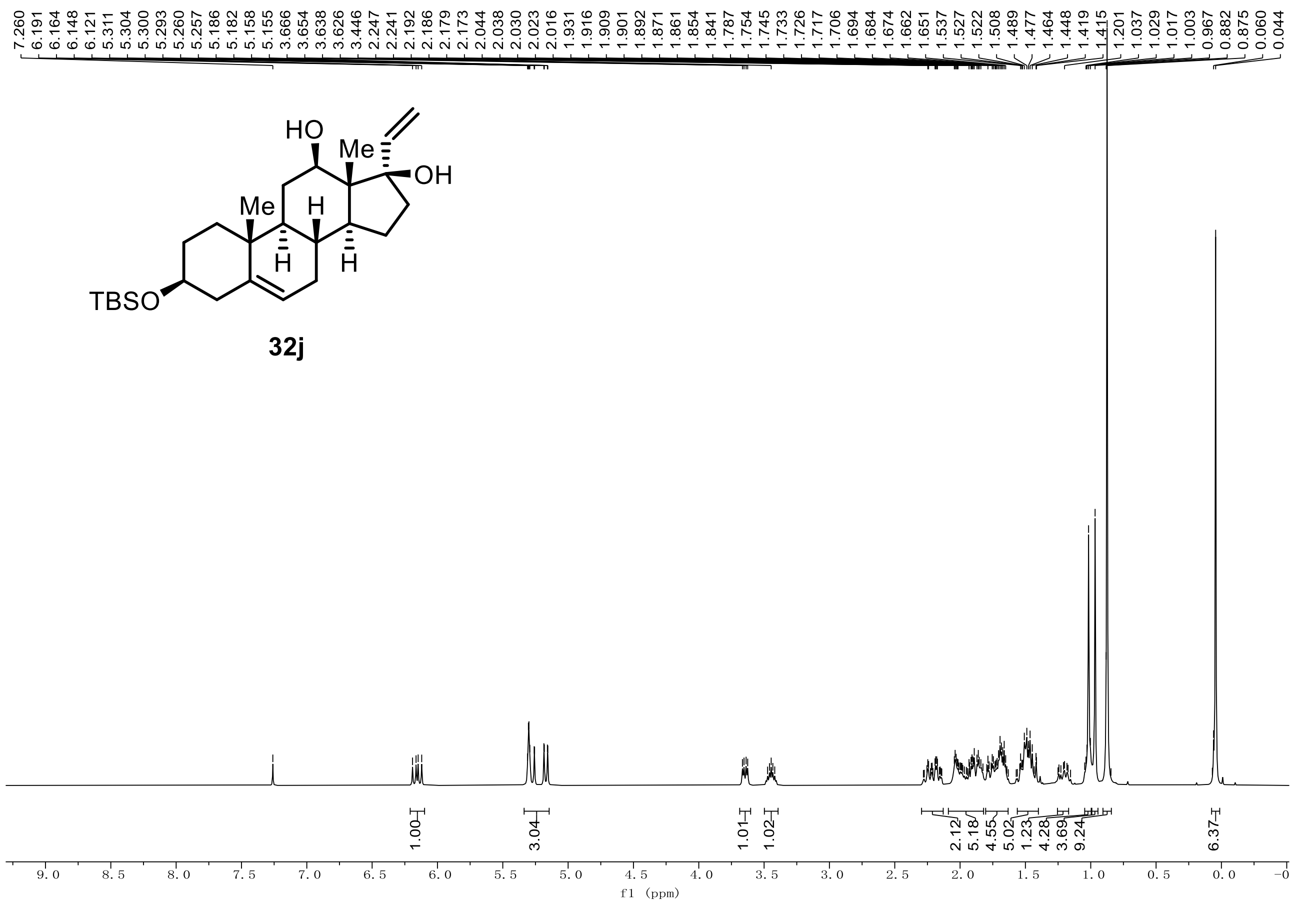

^{13}C NMR of **32j** (100 MHz, $CDCl_3$)

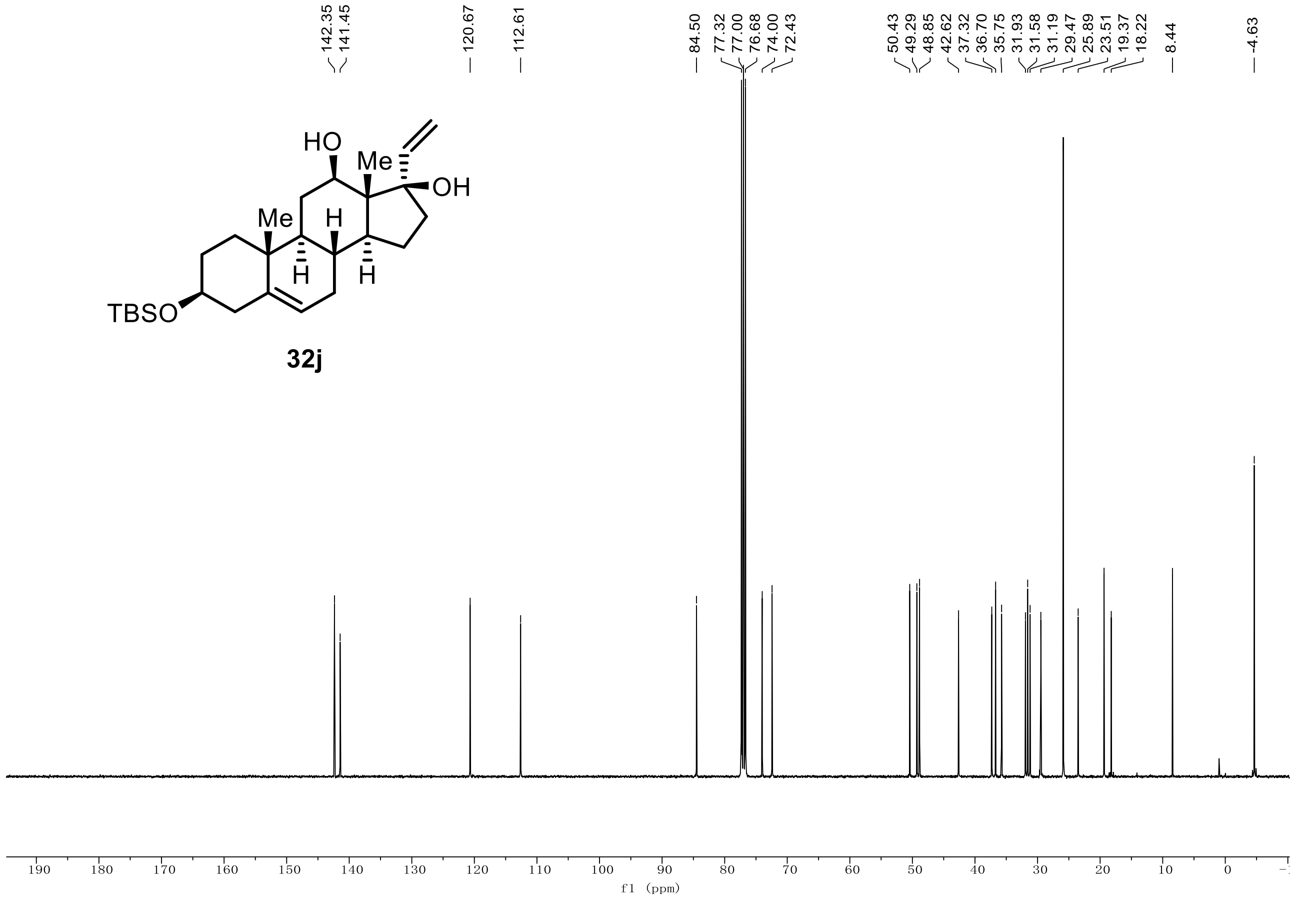

^{1}H NMR of **32k** (400 MHz, $CDCl_3$)

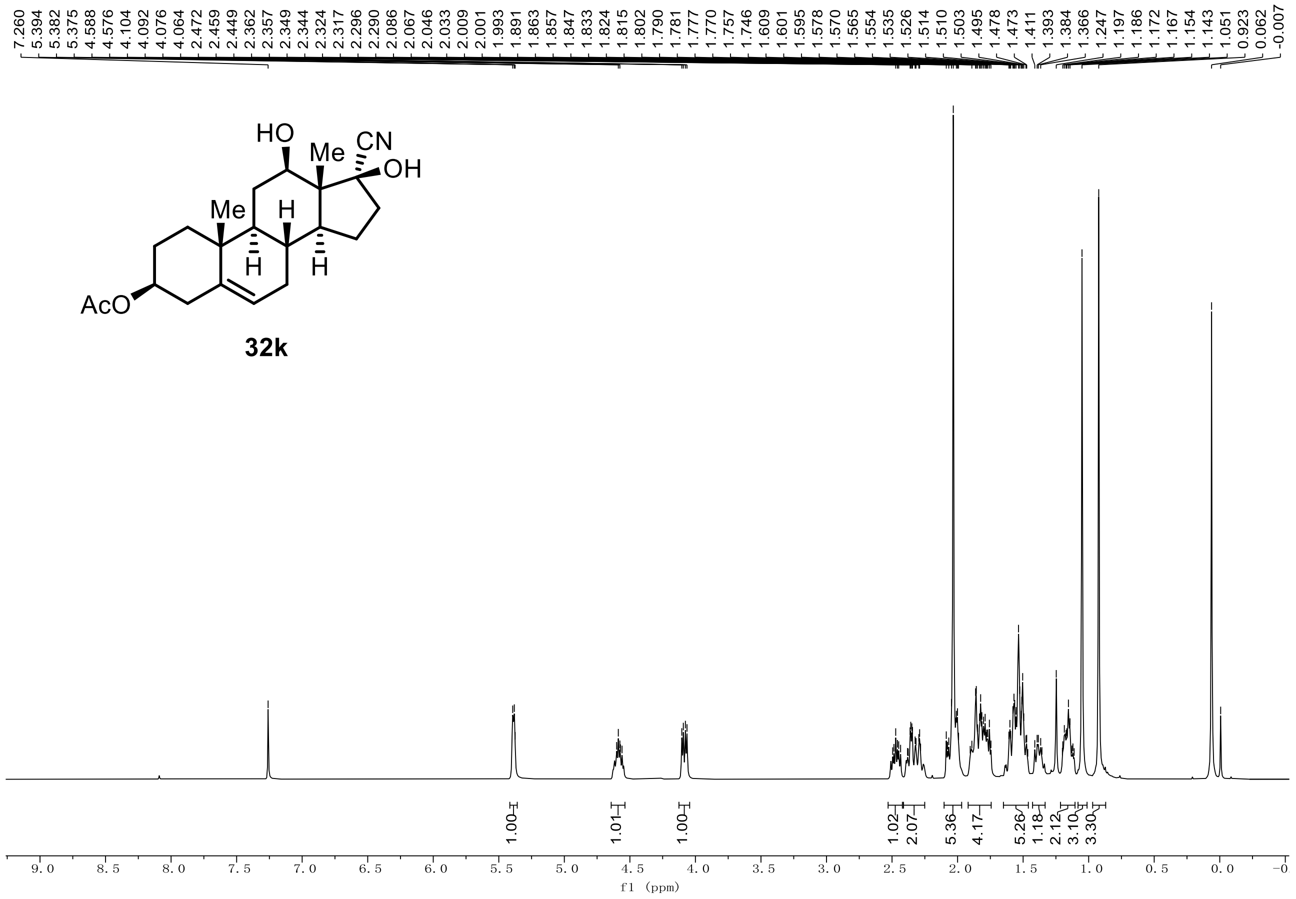

^{13}C NMR of **32k** (100 MHz, $CDCl_3$)

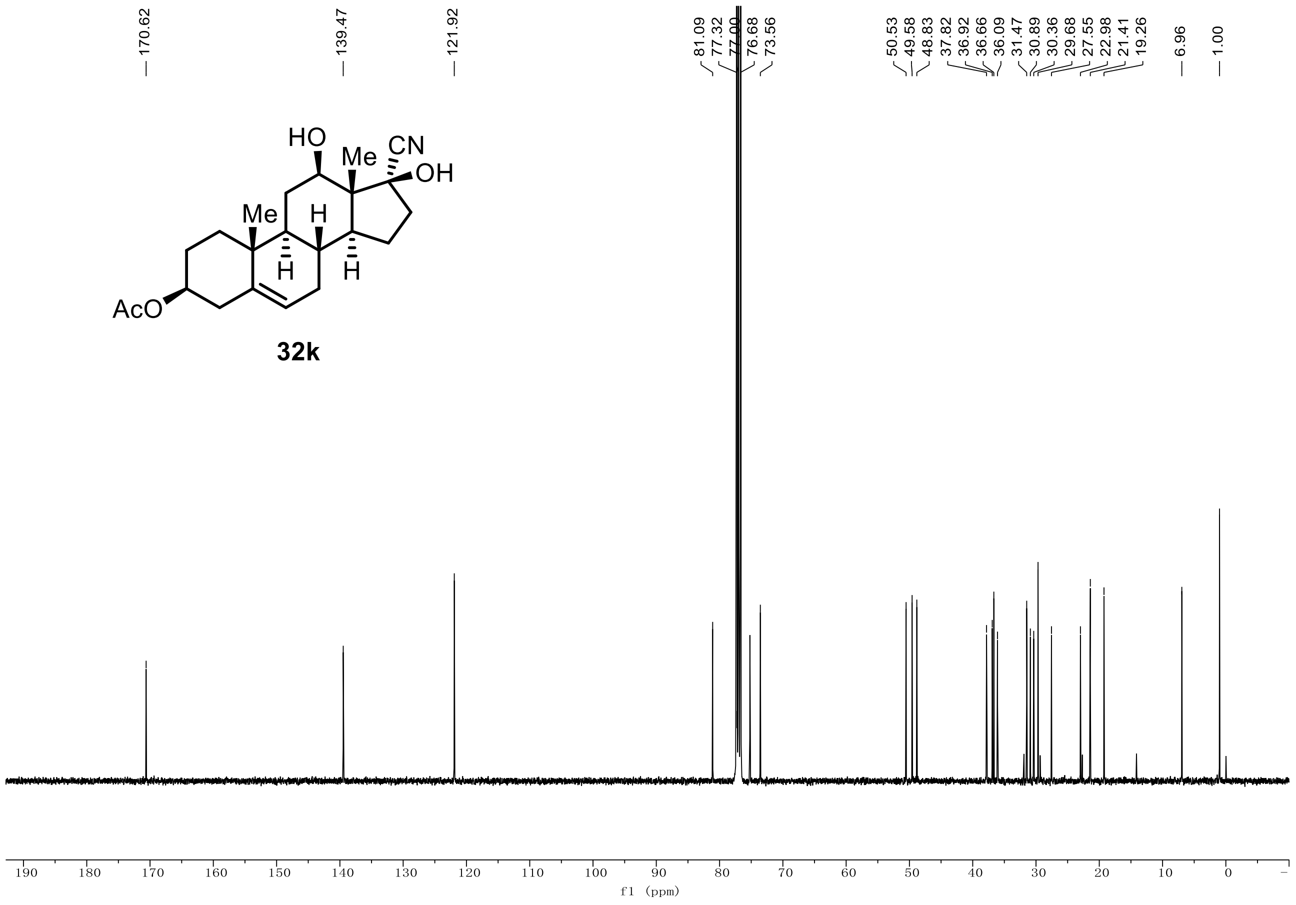

^{1}H NMR of **32l** (400 MHz, $CDCl_3$)

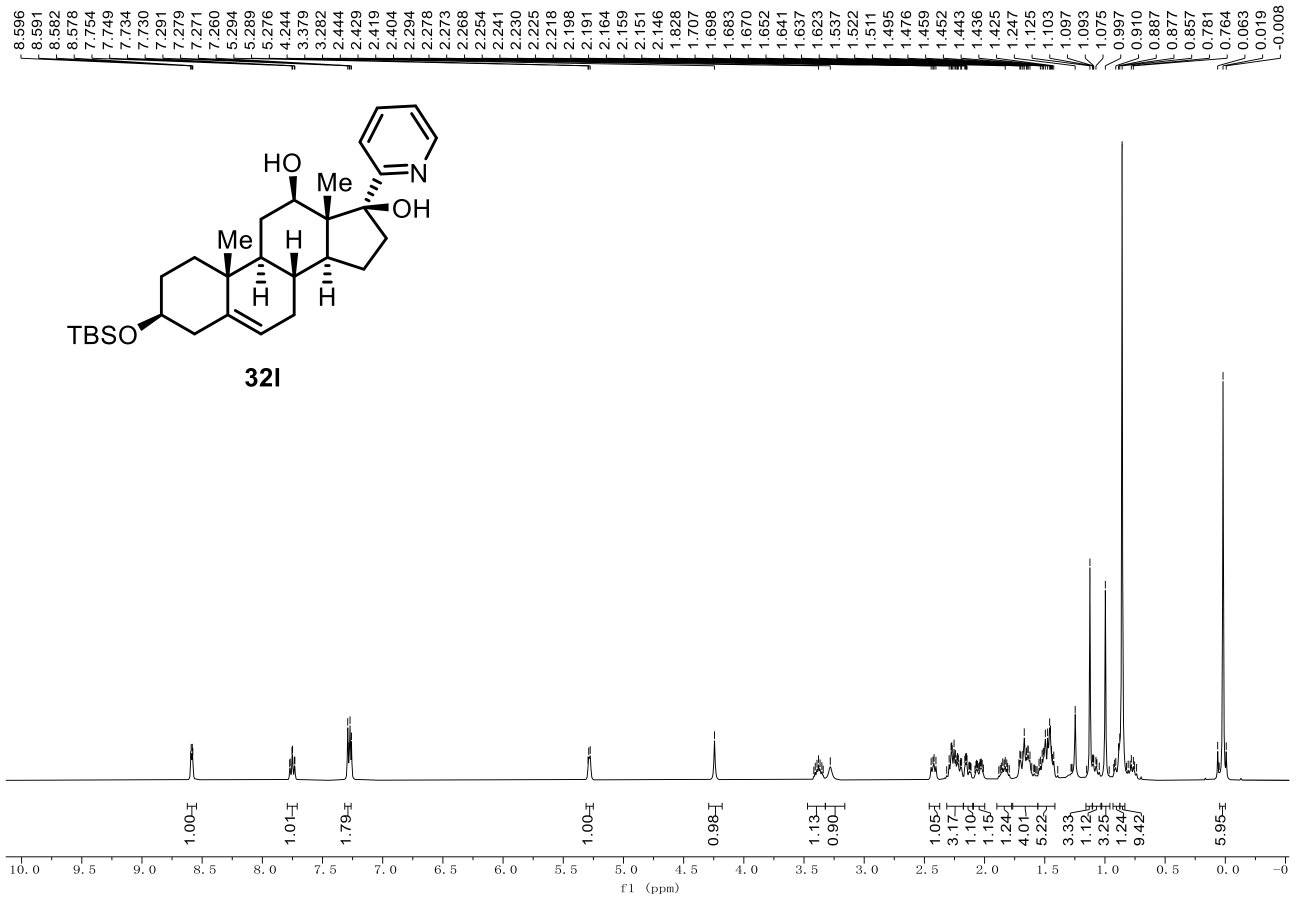

^{13}C NMR of **32l** (100 MHz, $CDCl_3$)

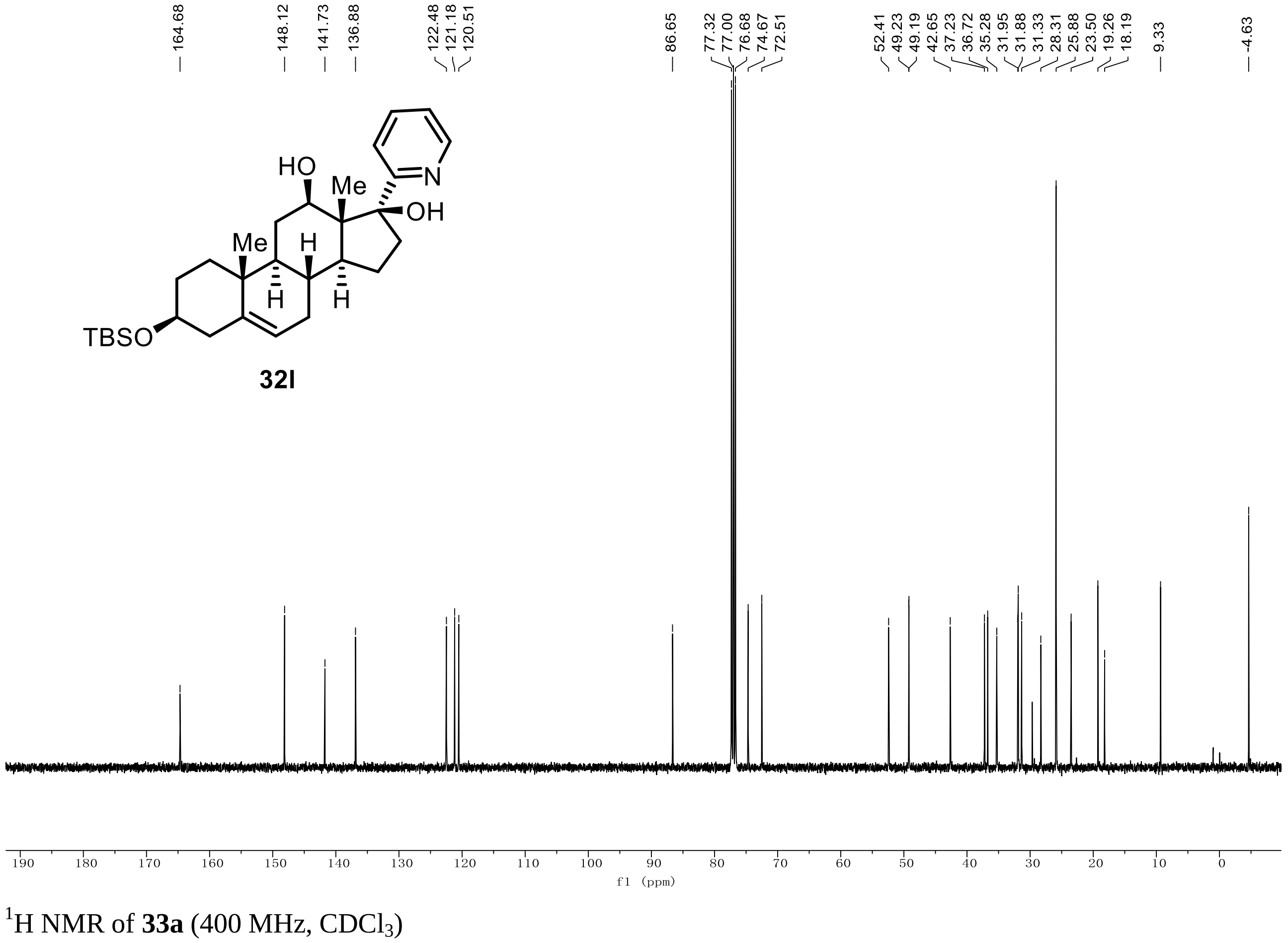

^{1}H NMR of **33a** (400 MHz, $CDCl_3$)

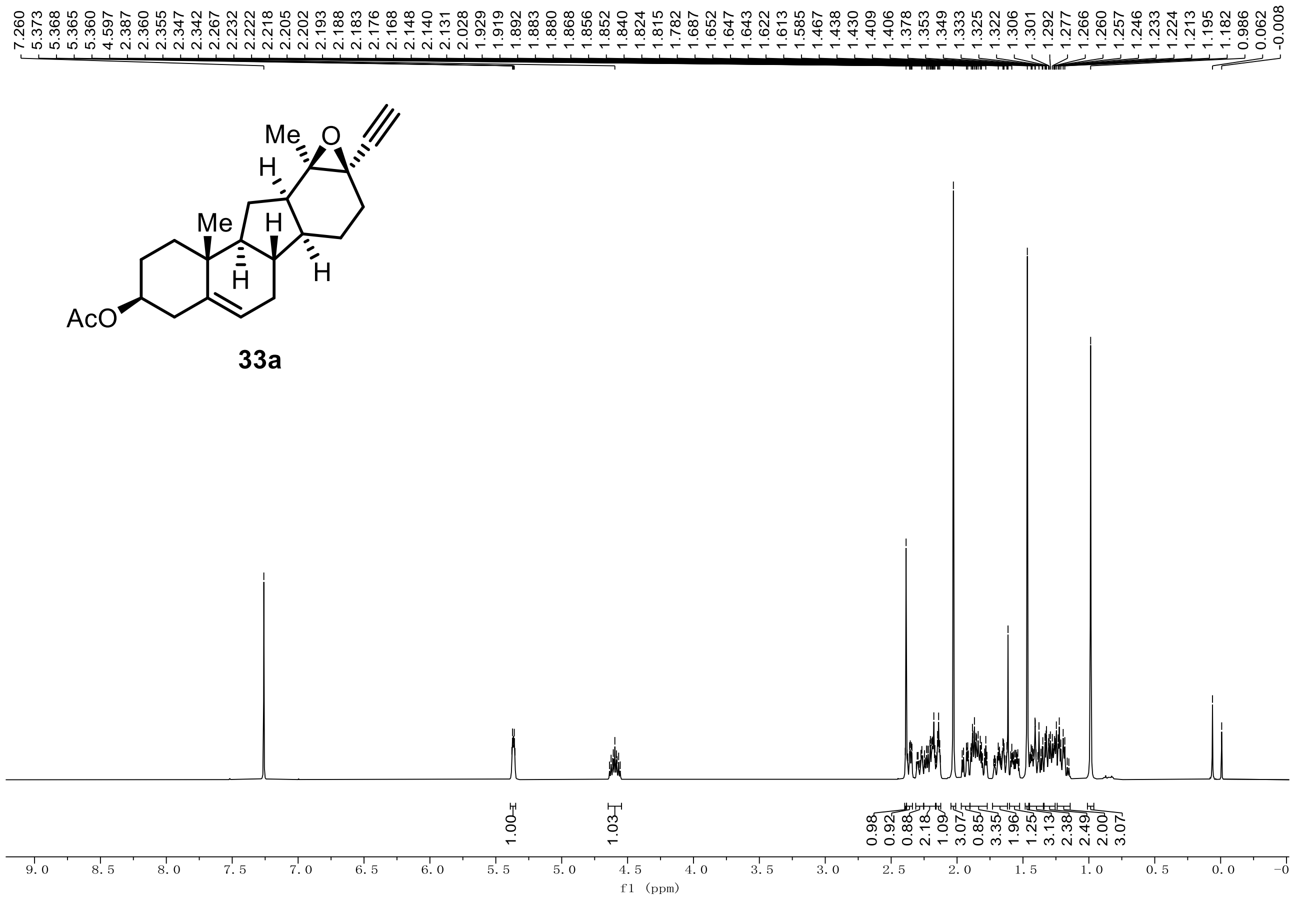

^{13}C NMR of **33a** (100 MHz, $CDCl_3$)

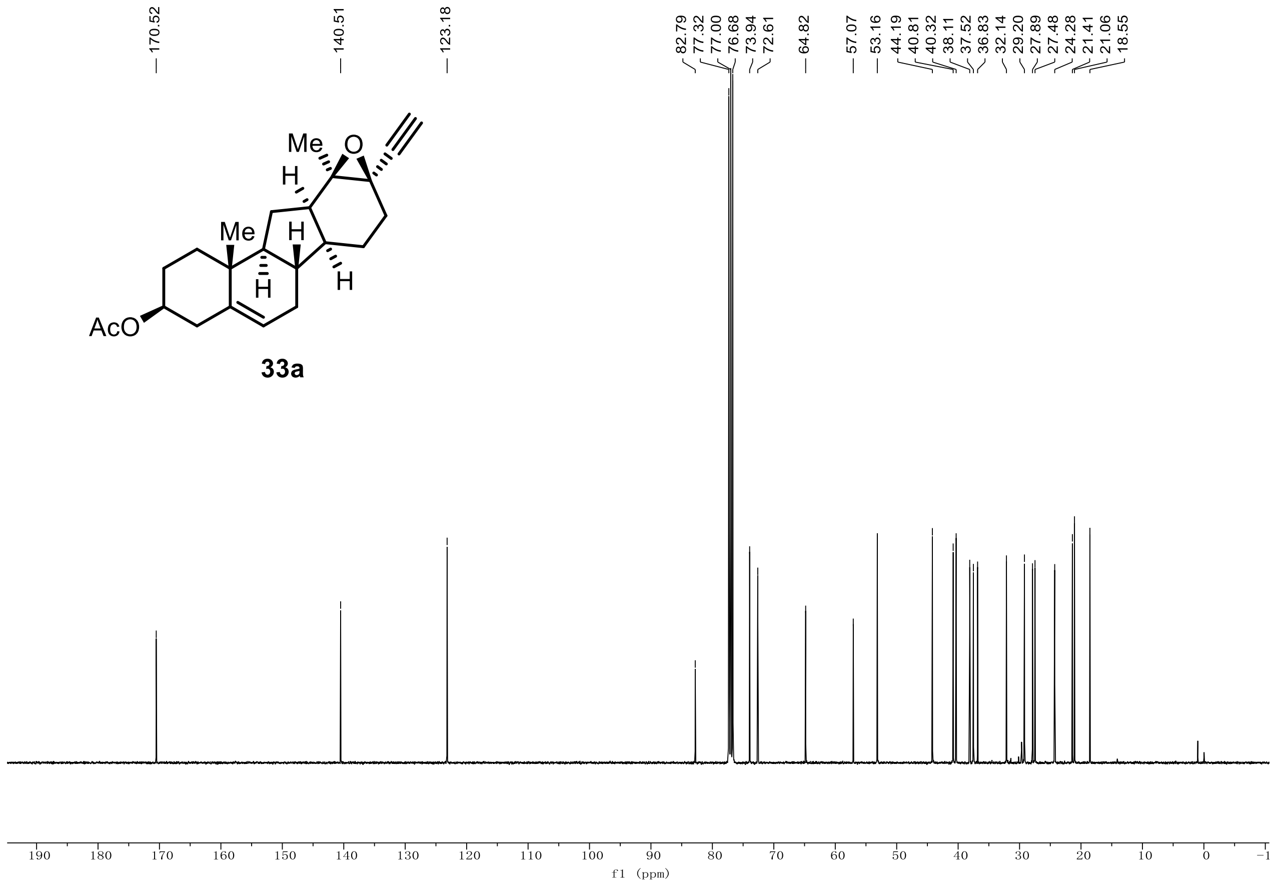

^{1}H NMR of **33b** (400 MHz, $CDCl_3$)

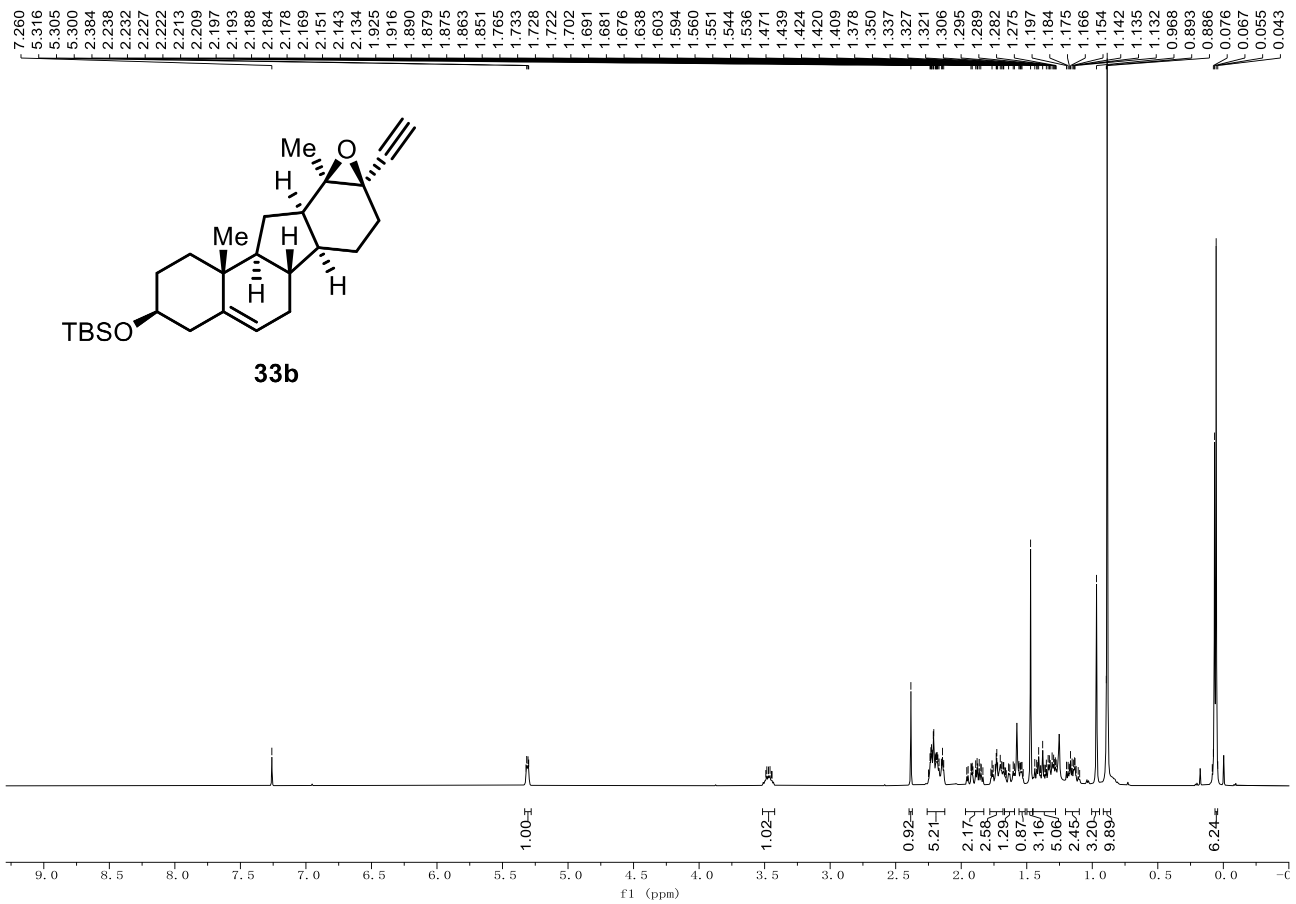

^{13}C NMR of **33b** (100 MHz, CDCl$_3$)

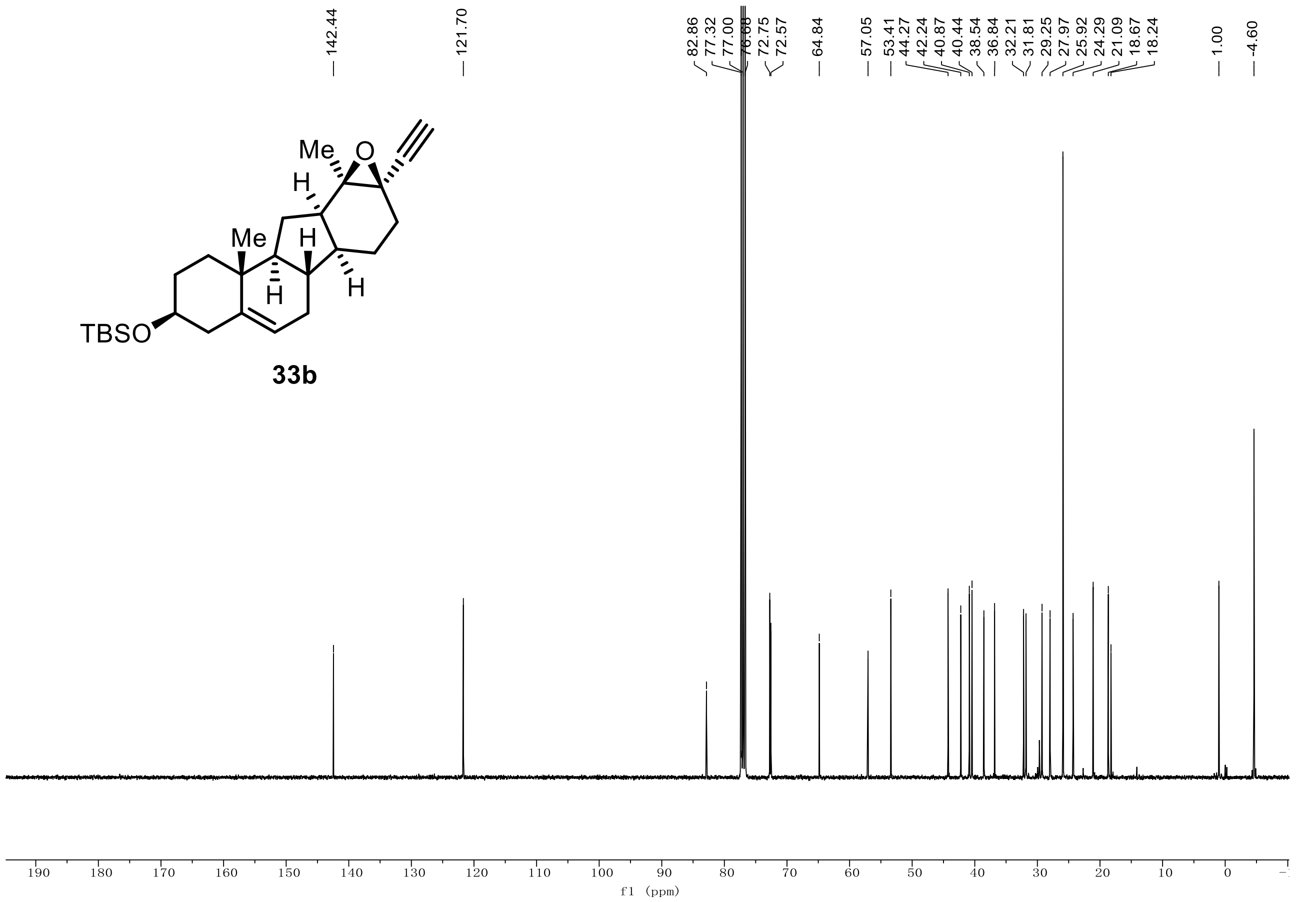

^{1}H NMR of **33c** (400 MHz, CDCl$_3$)

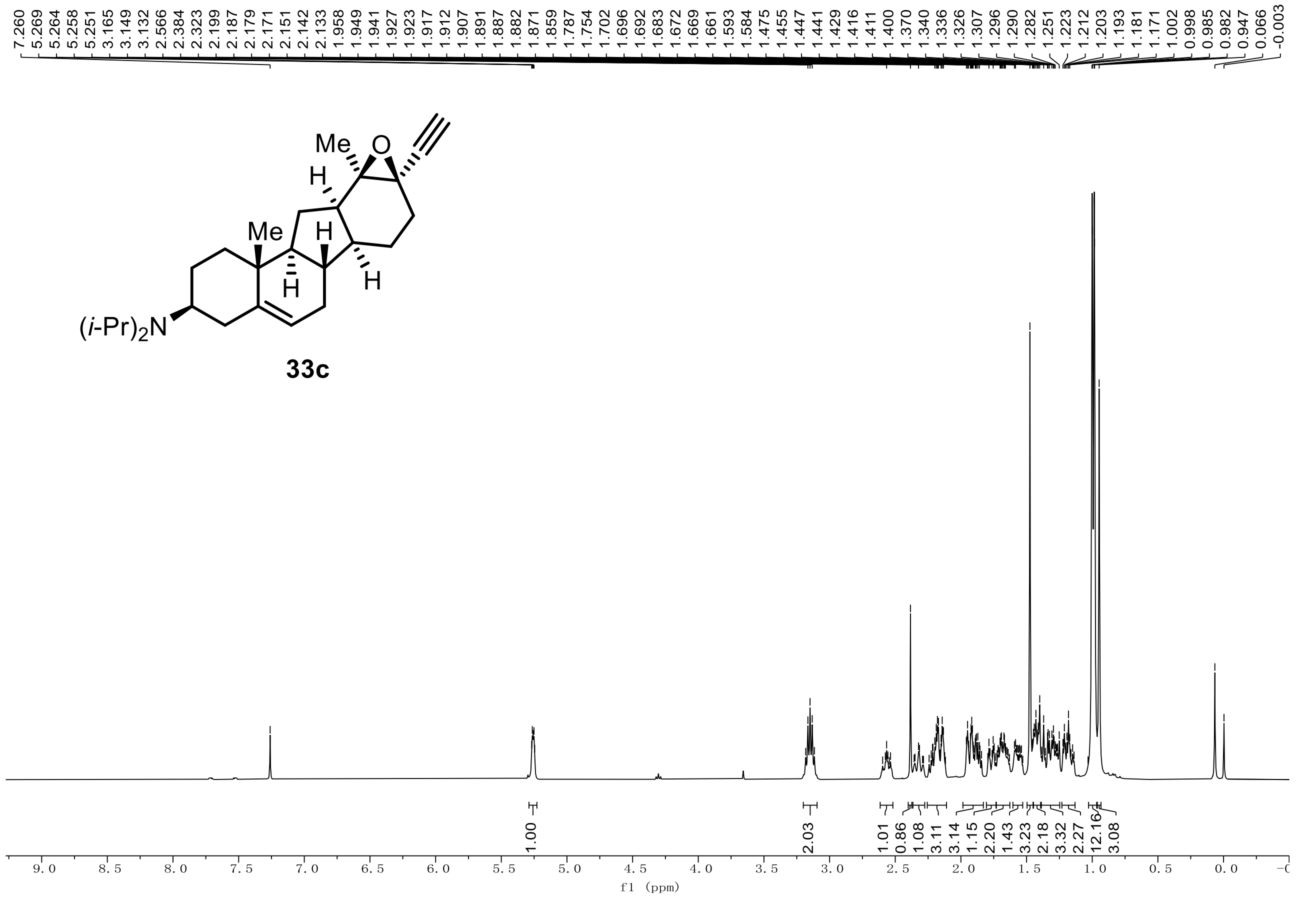

^{13}C NMR of **33c** (100 MHz, $CDCl_3$)

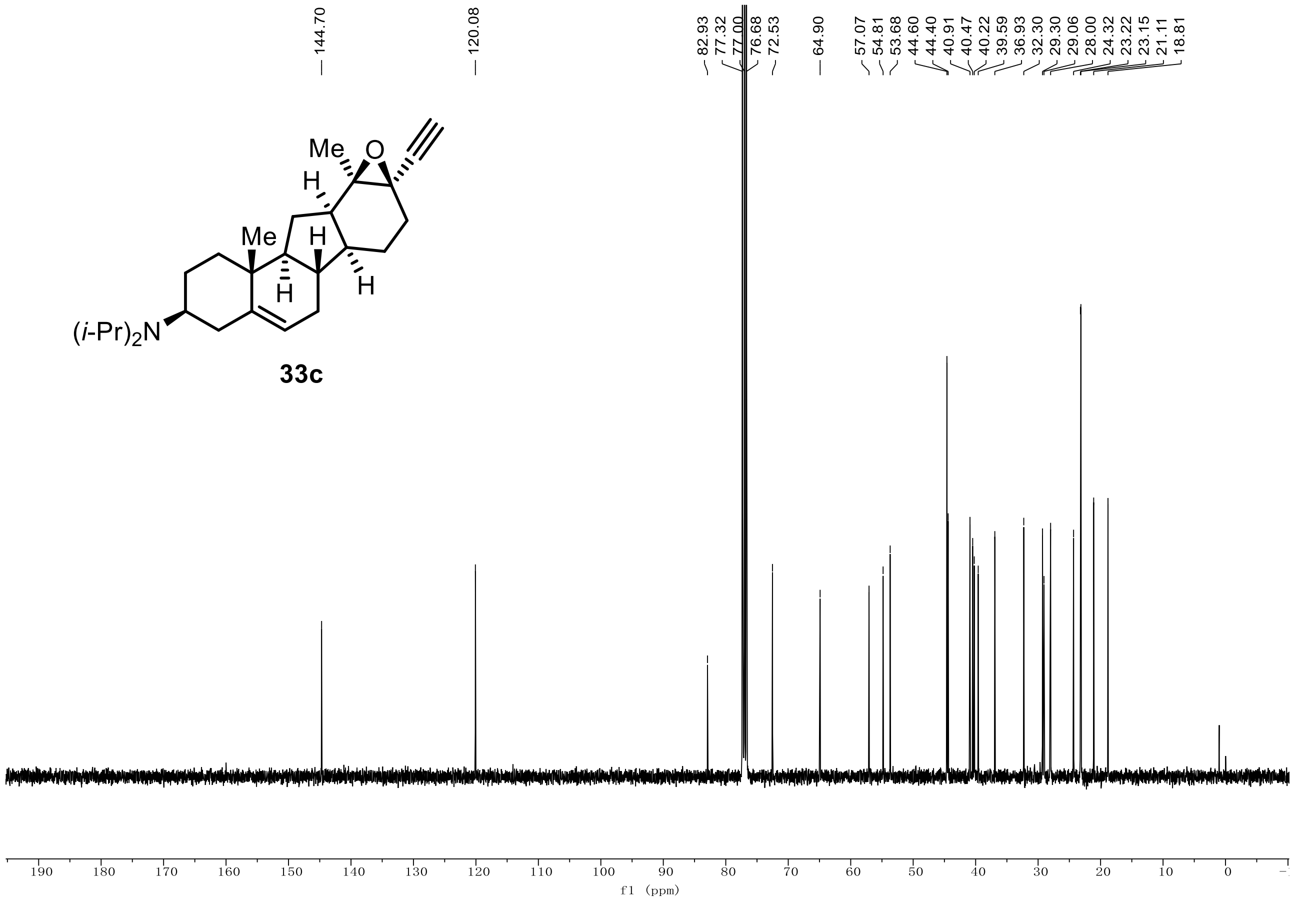

^{1}H NMR of **33d** (400 MHz, $CDCl_3$)

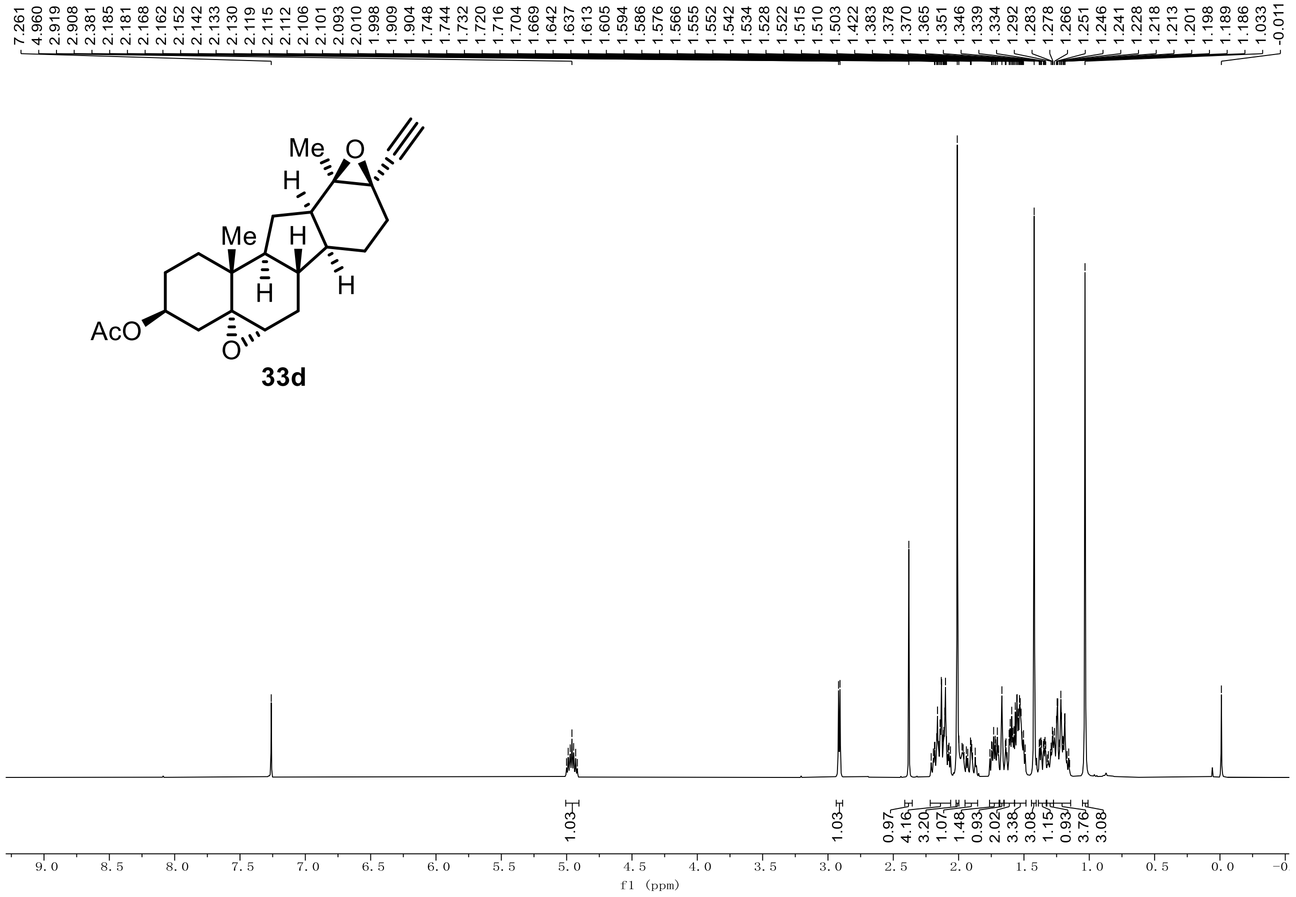

^{13}C NMR of **33d** (100 MHz, $CDCl_3$)

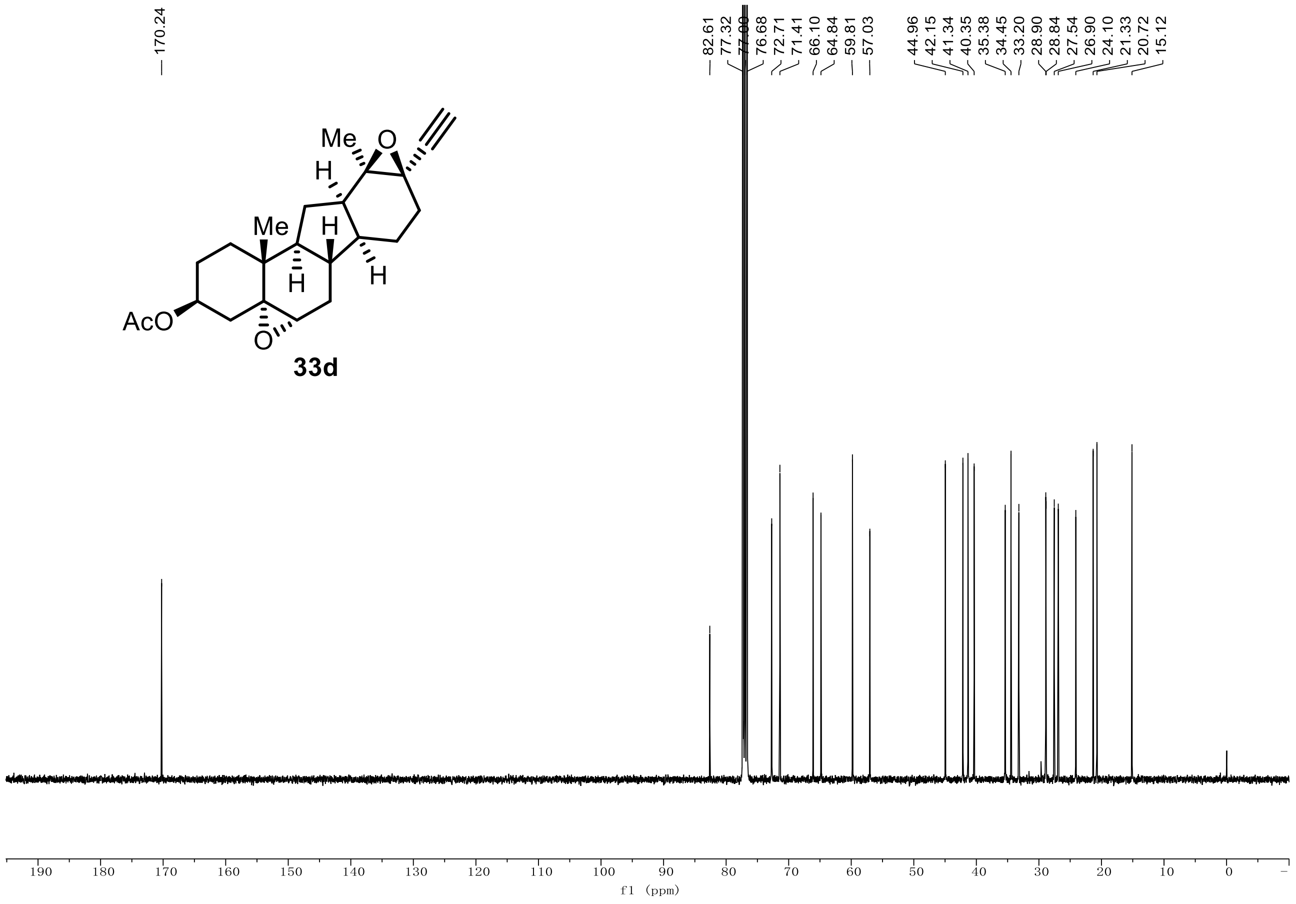

^{1}H NMR of **33e** (400 MHz, $CDCl_3$)

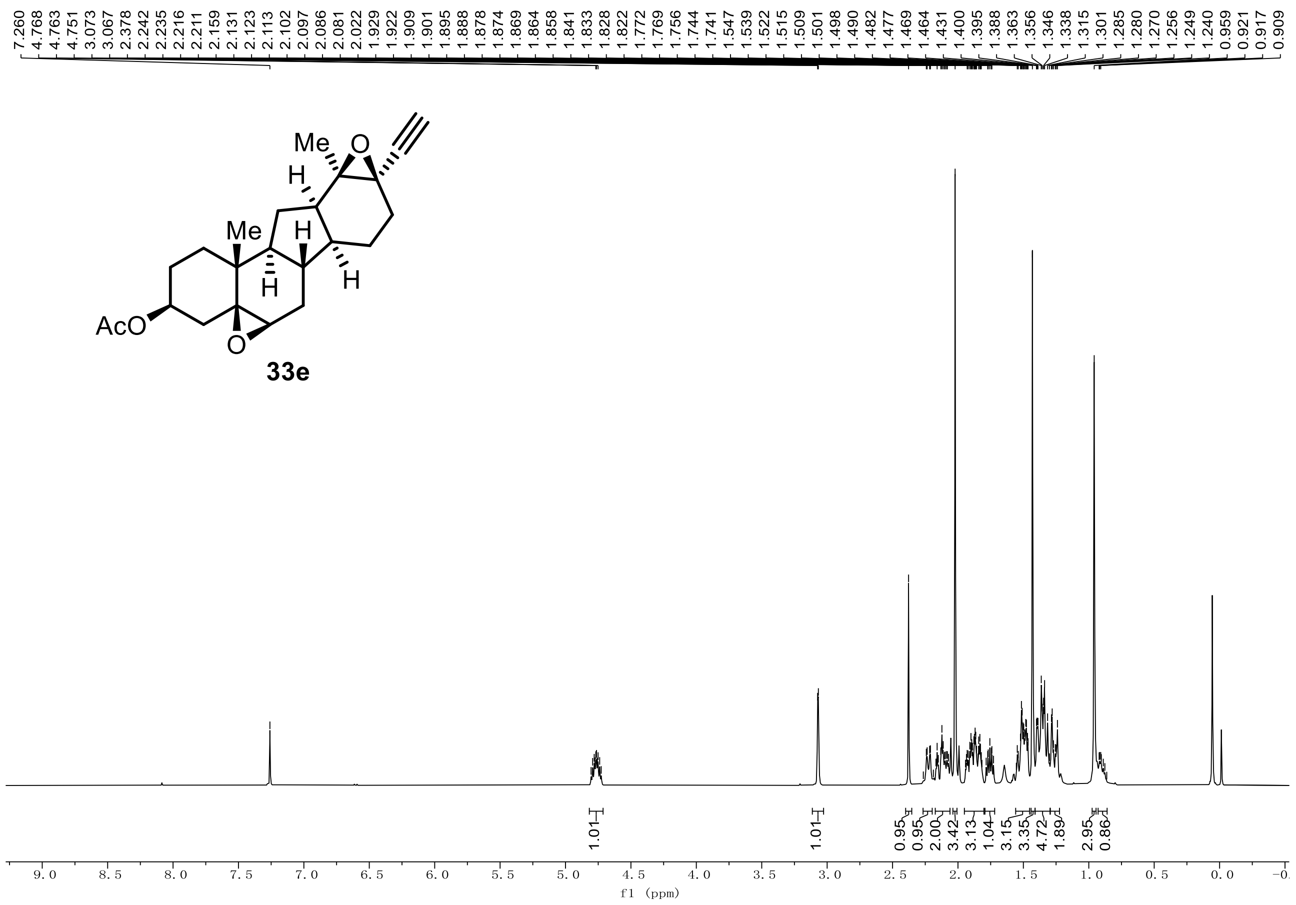

^{13}C NMR of **33e** (100 MHz, $CDCl_3$)

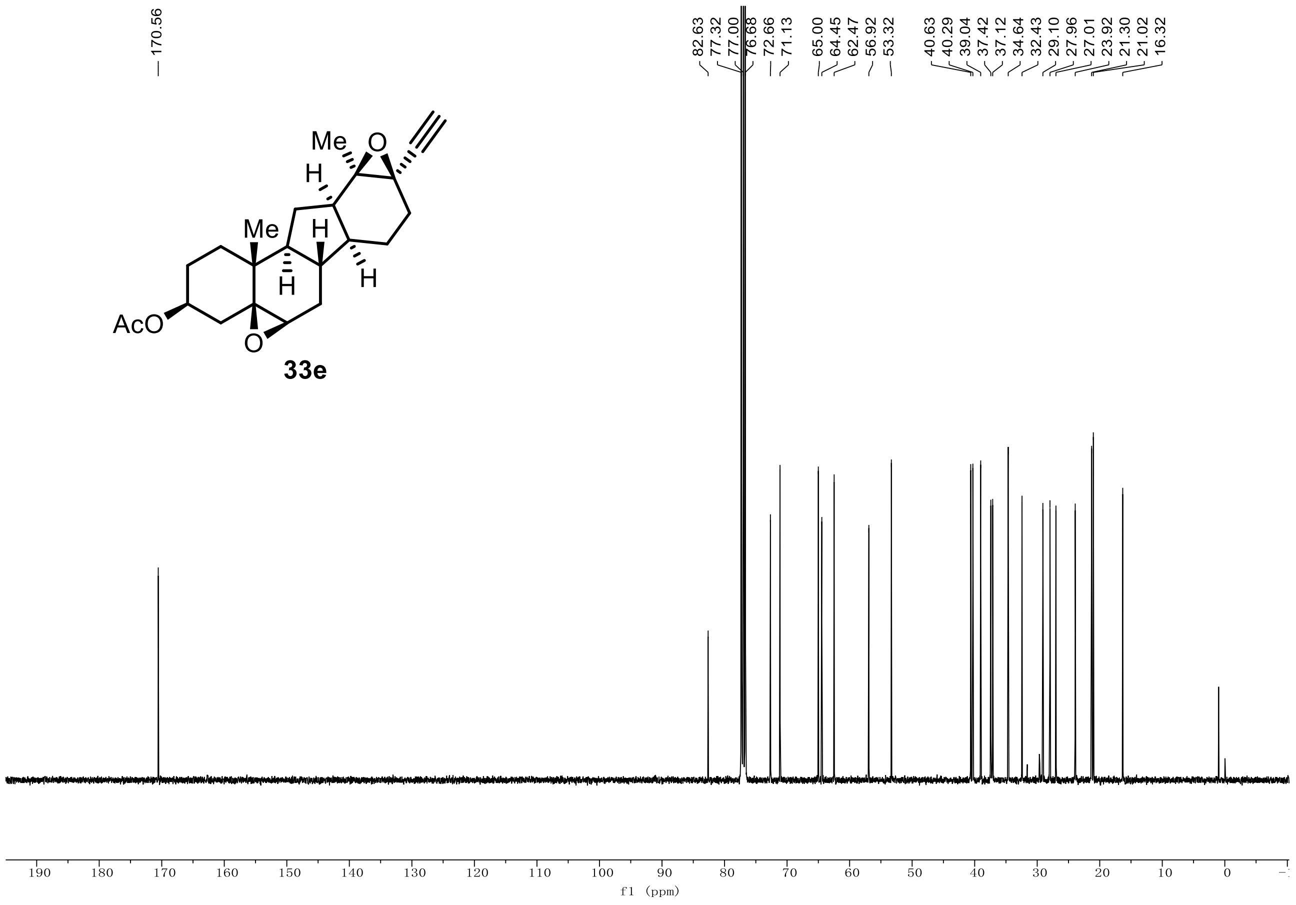

^{1}H NMR of **33f** (400 MHz, $CDCl_3$)

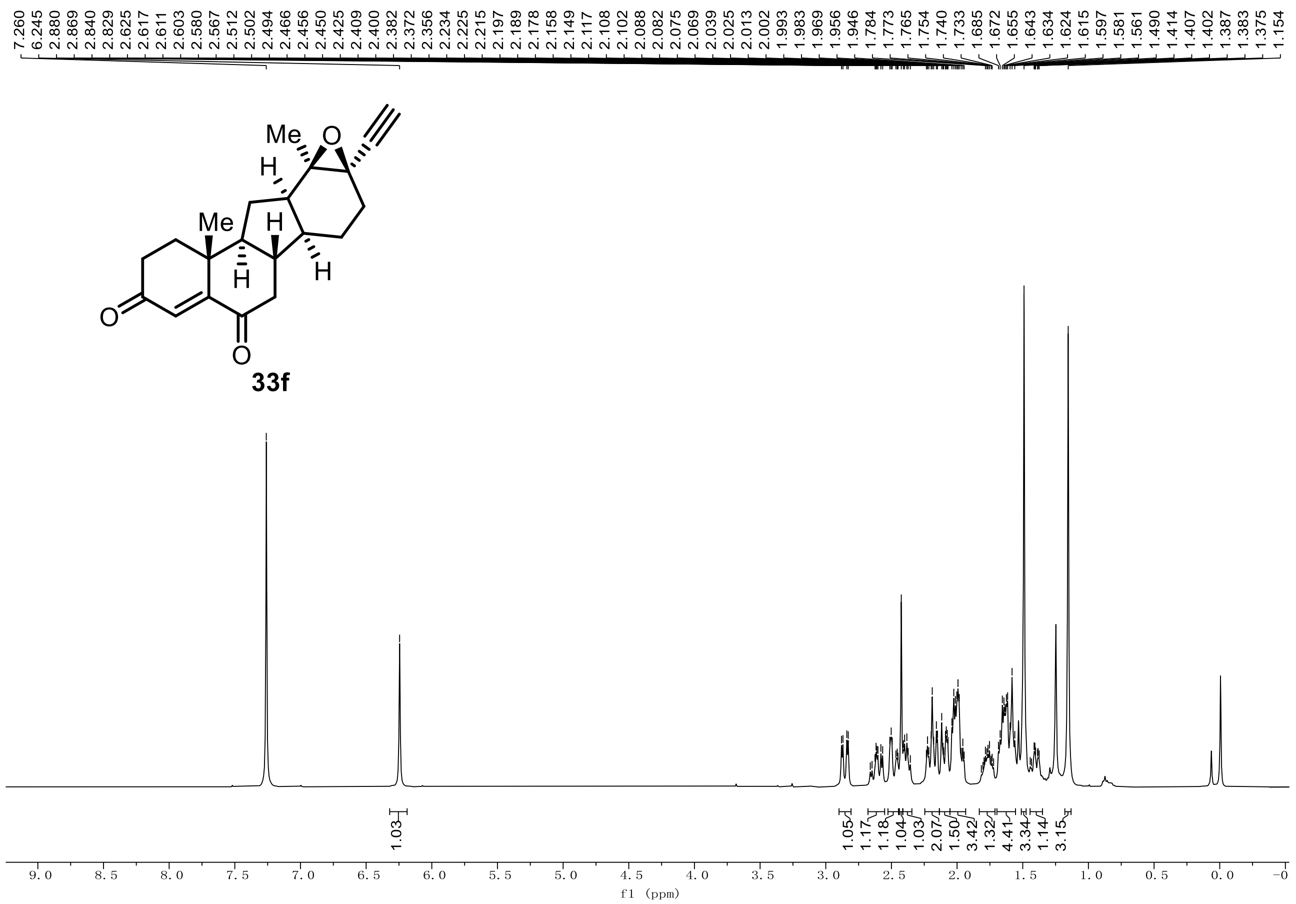

^{13}C NMR of **33f** (100 MHz, $CDCl_3$)

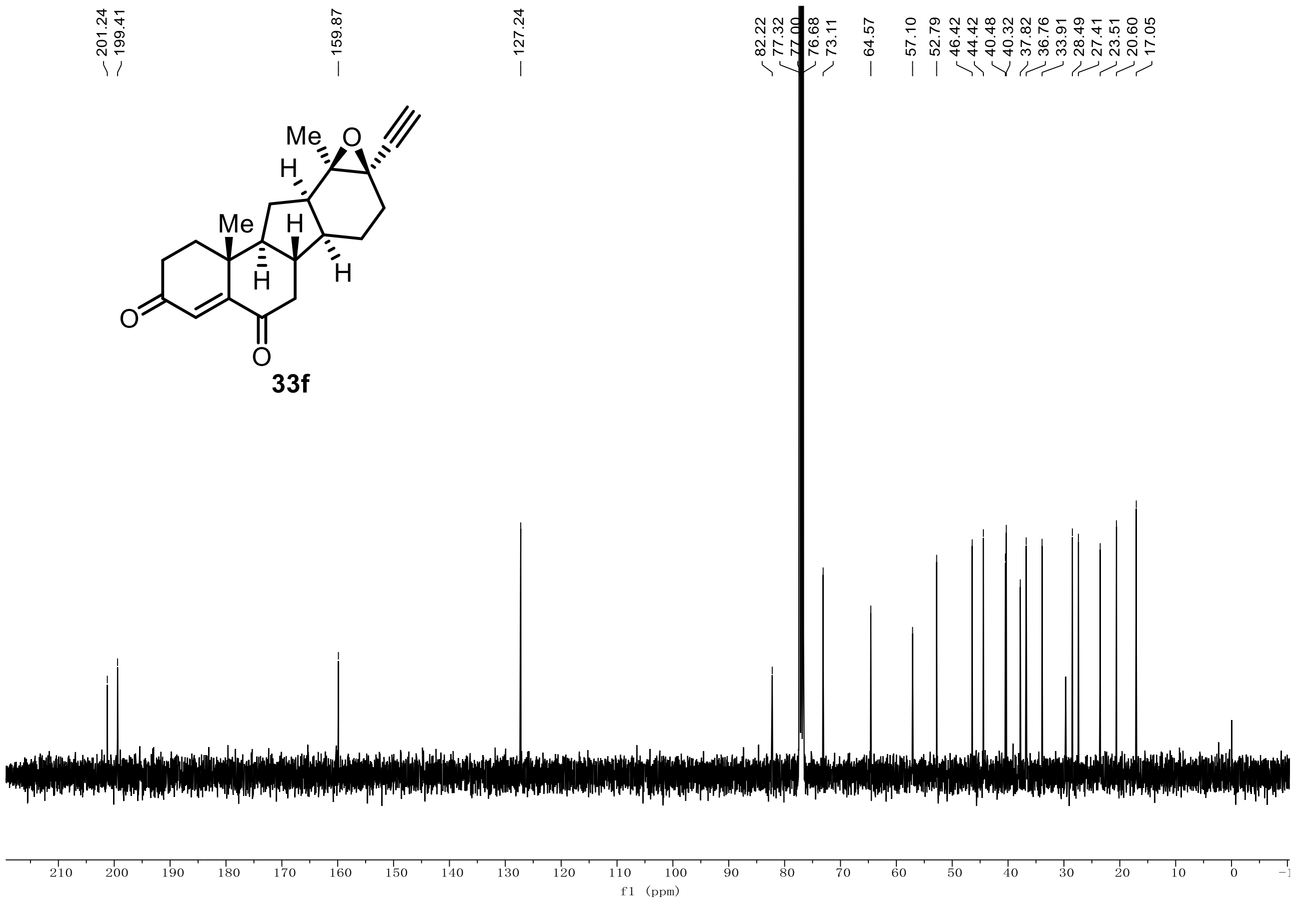

^{1}H NMR of **33g** (400 MHz, $CDCl_3$)

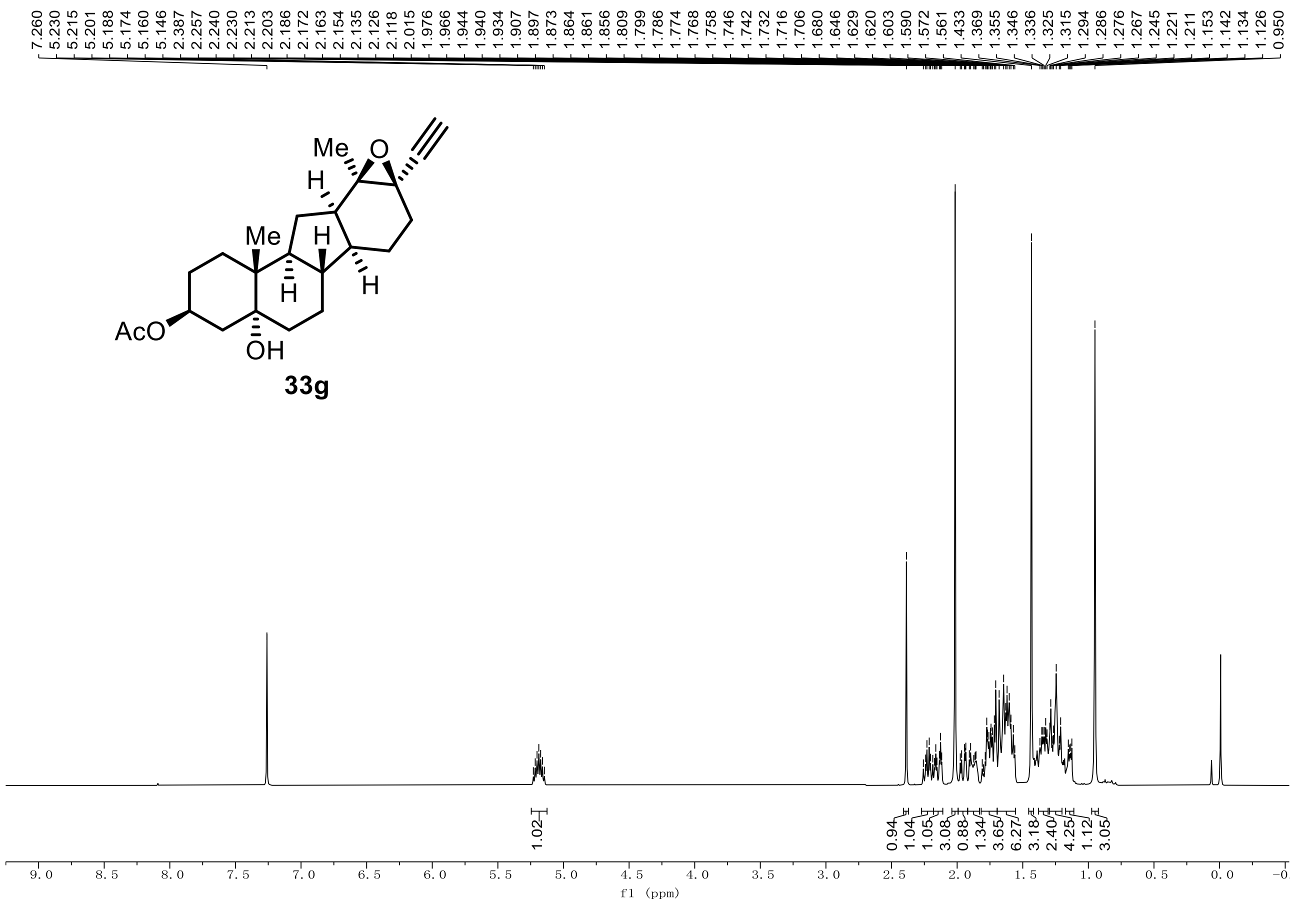

^{13}C NMR of **33g** (100 MHz, $CDCl_3$)

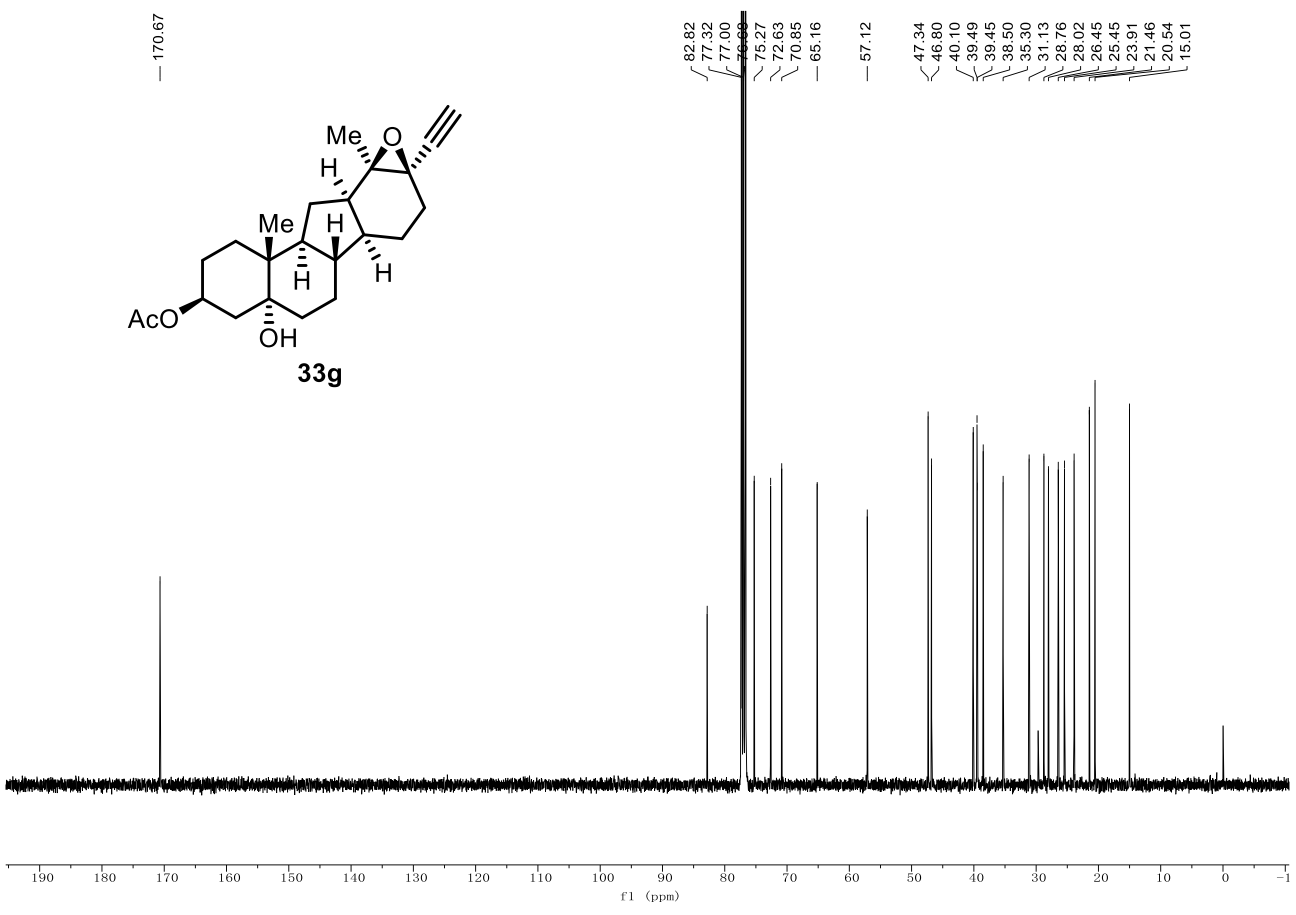

^{1}H NMR of **33h** (400 MHz, $CDCl_3$)

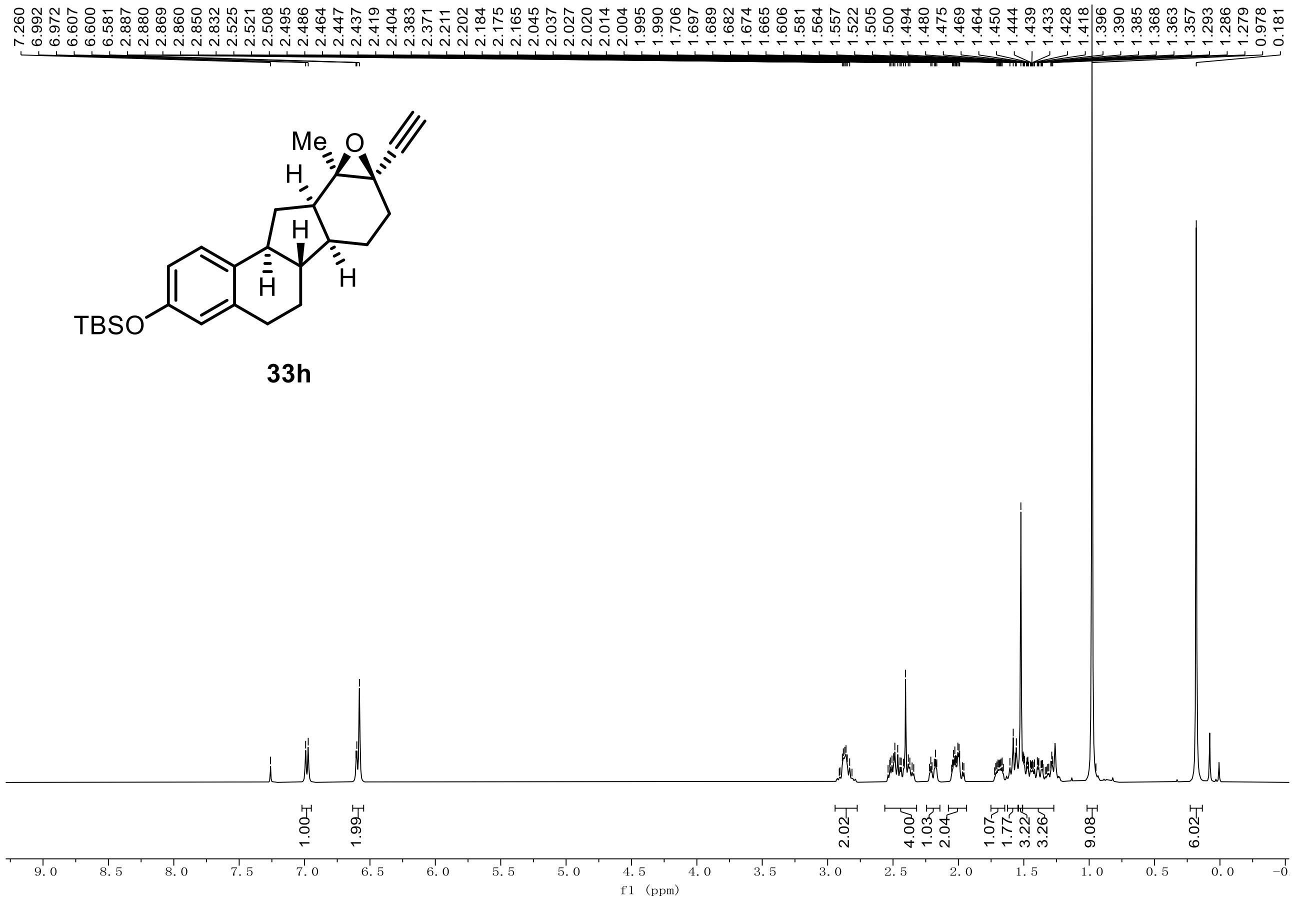

^{13}C NMR of **33h** (100 MHz, $CDCl_3$)

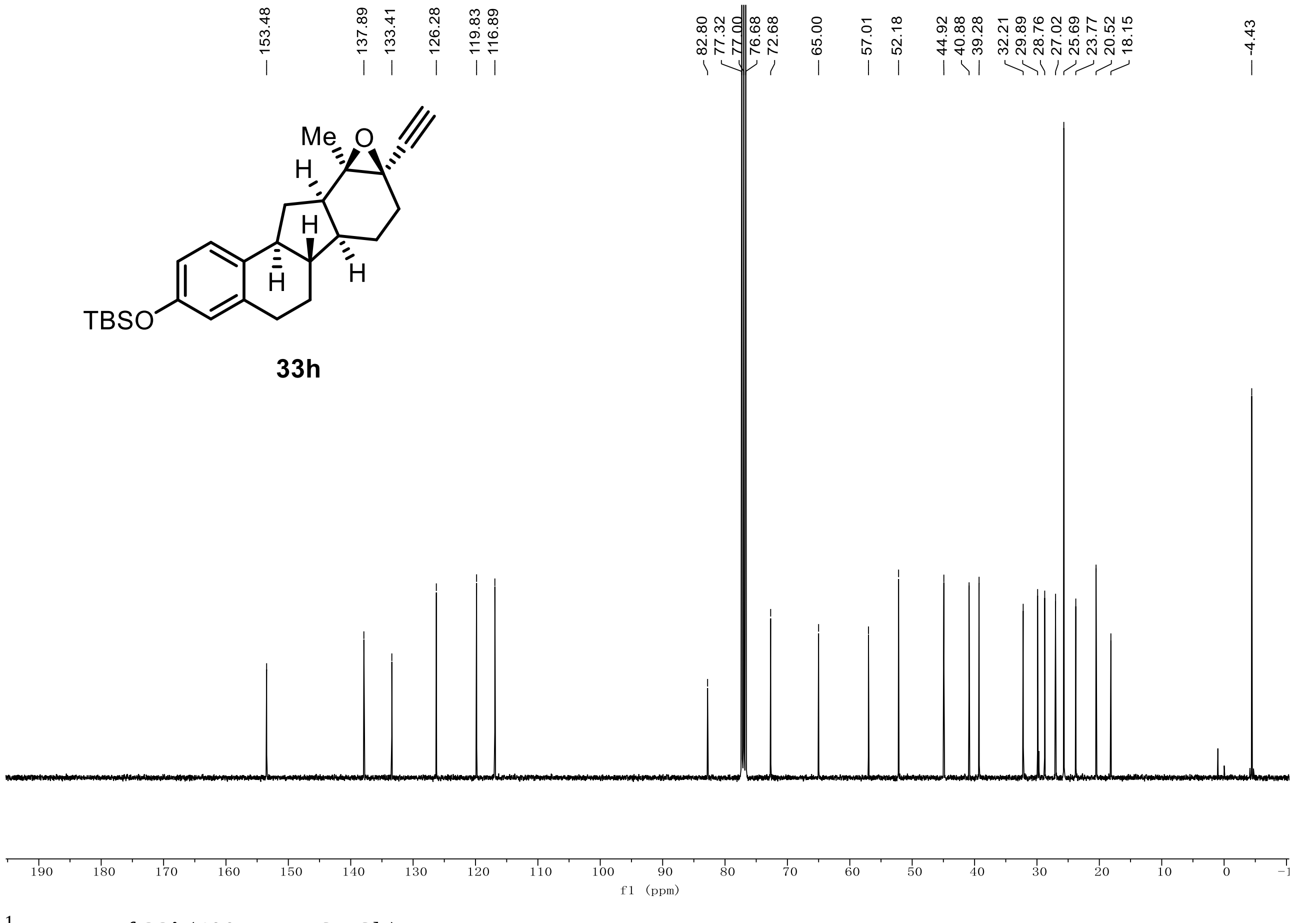

^{1}H NMR of **33i** (400 MHz, $CDCl_3$)

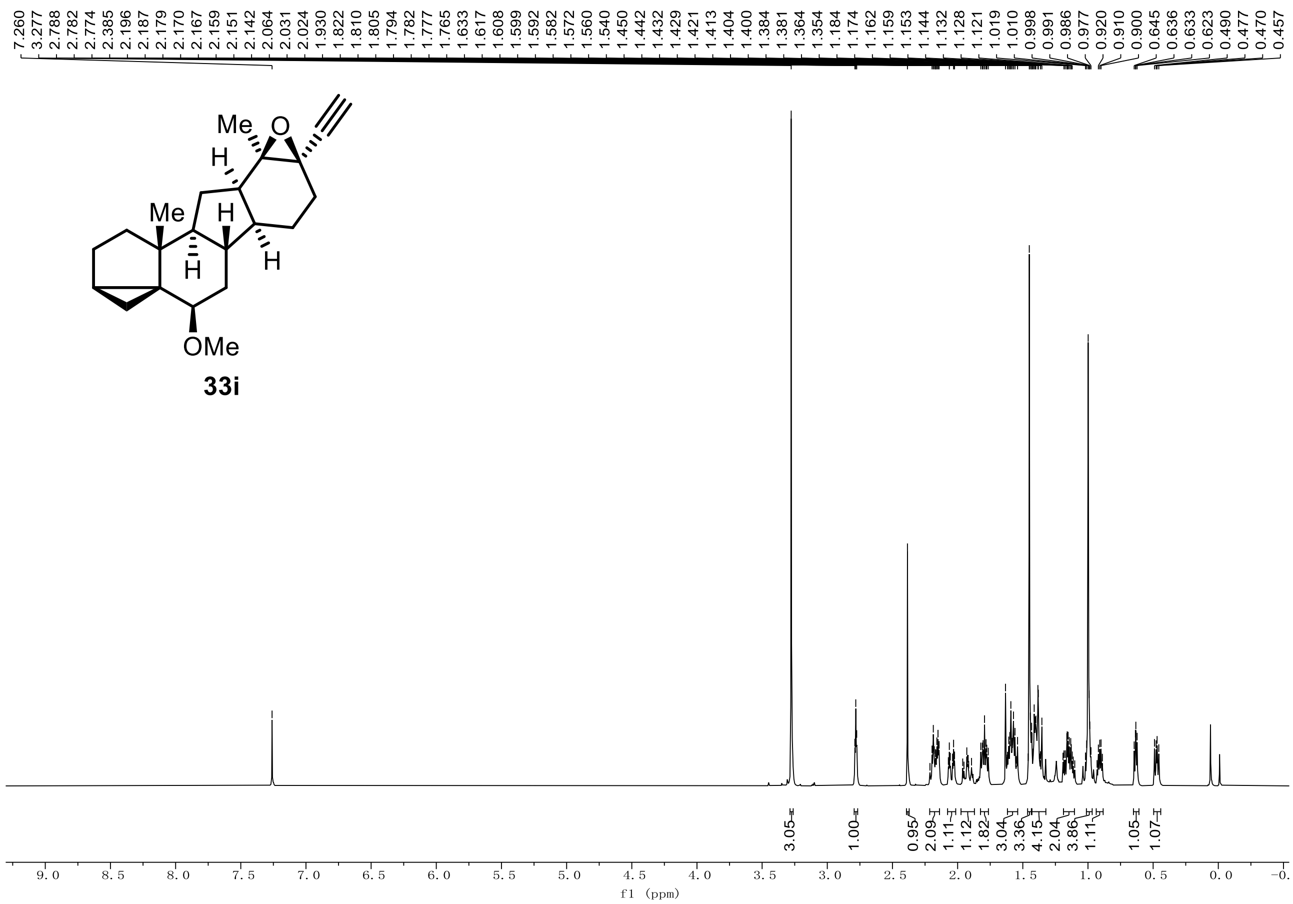

^{13}C NMR of **33i** (100 MHz, $CDCl_3$)

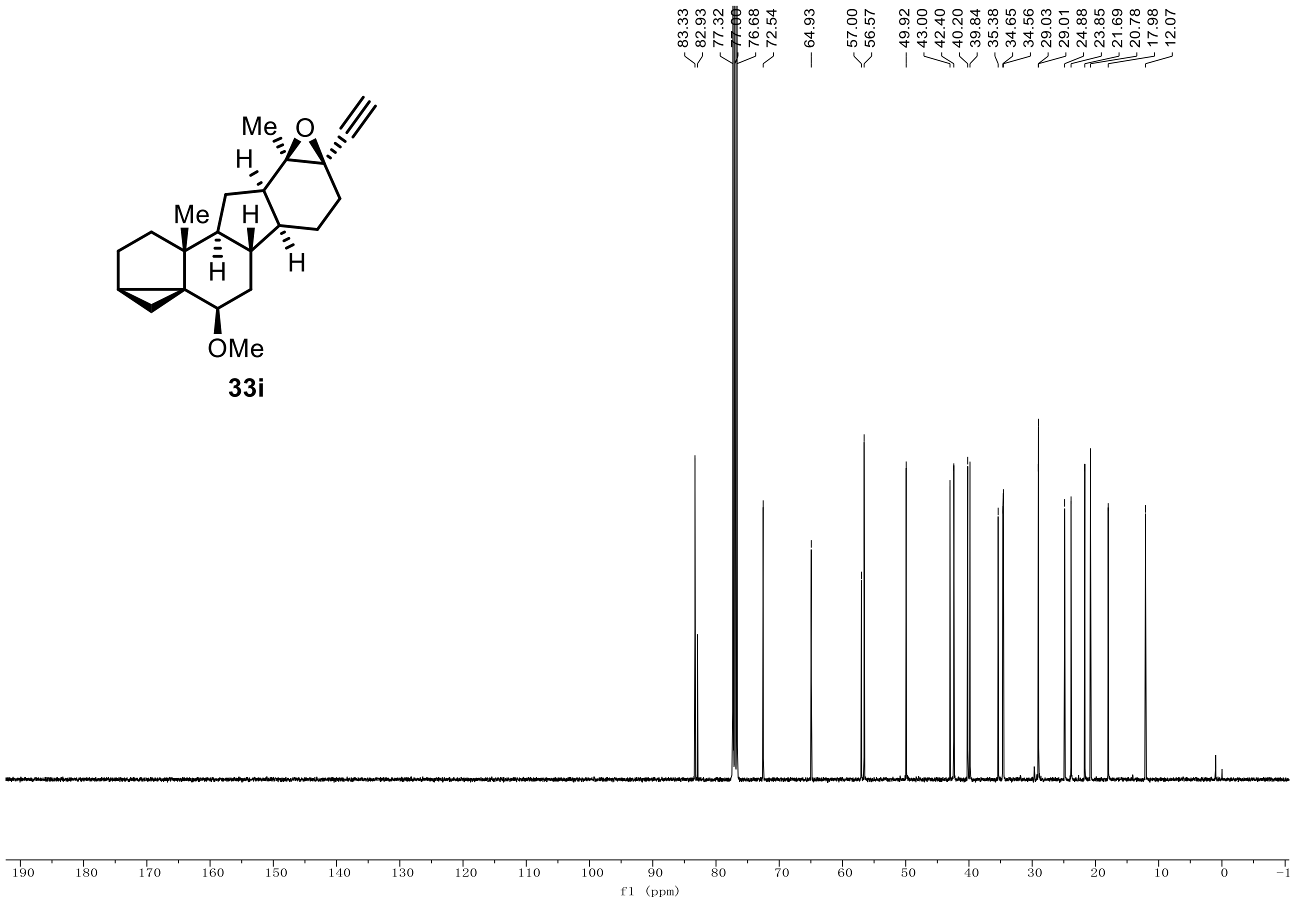

^{1}H NMR of **33j** (600 MHz, $CDCl_3$)

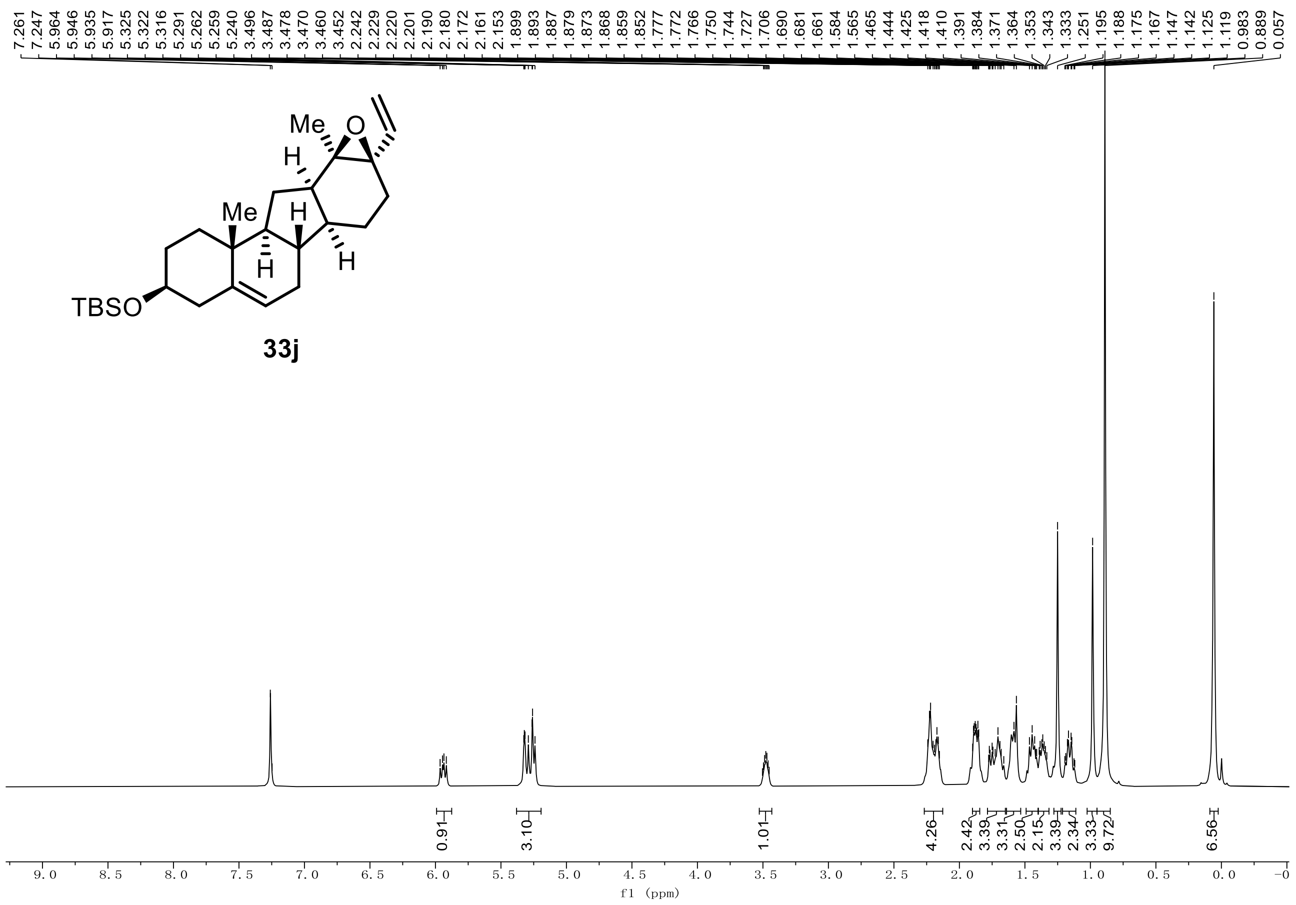

^{13}C NMR of **33j** (100 MHz, $CDCl_3$)

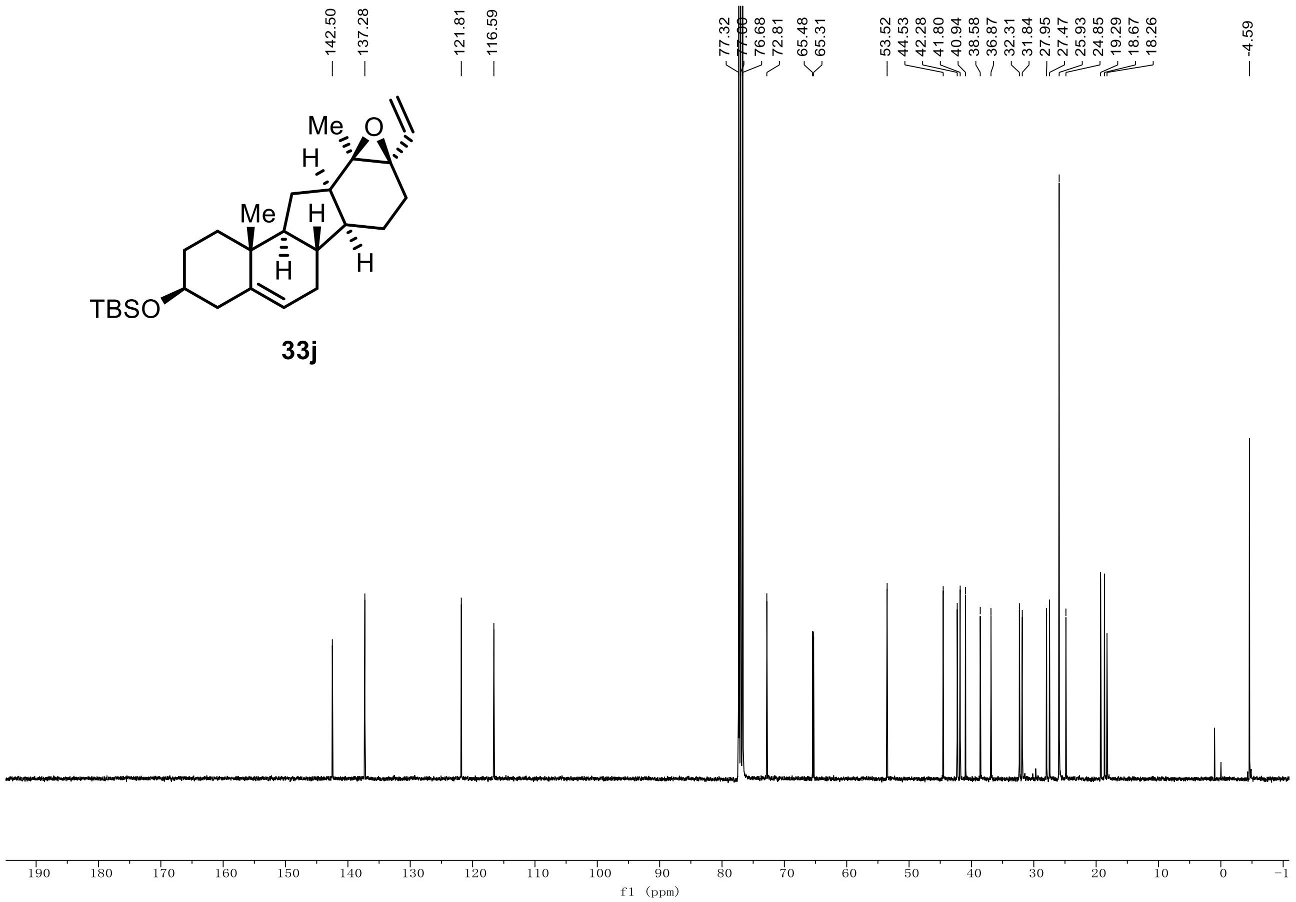

^{1}H NMR of **33k** (400 MHz, $CDCl_3$)

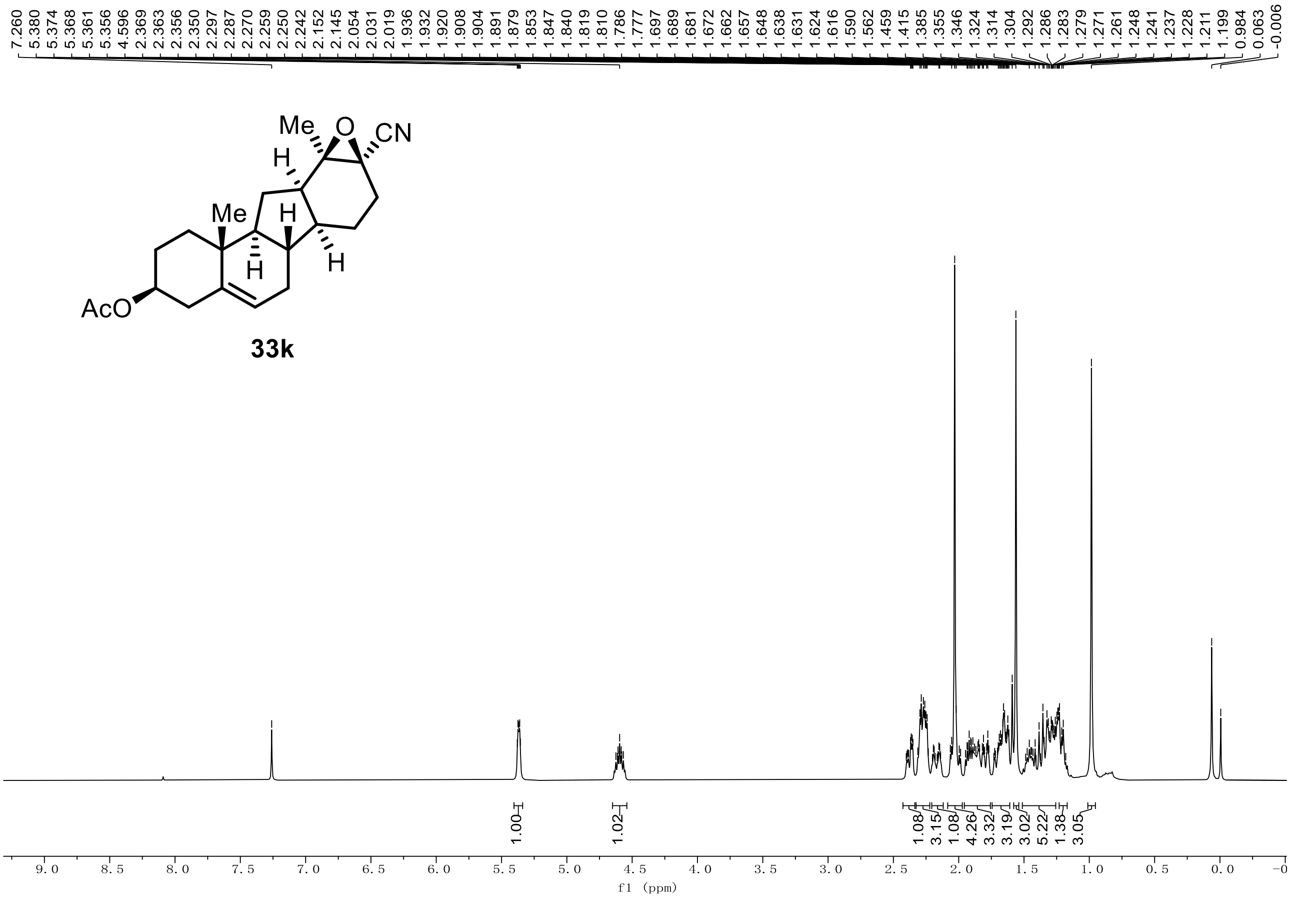

^{13}C NMR of **33k** (100 MHz, $CDCl_3$)

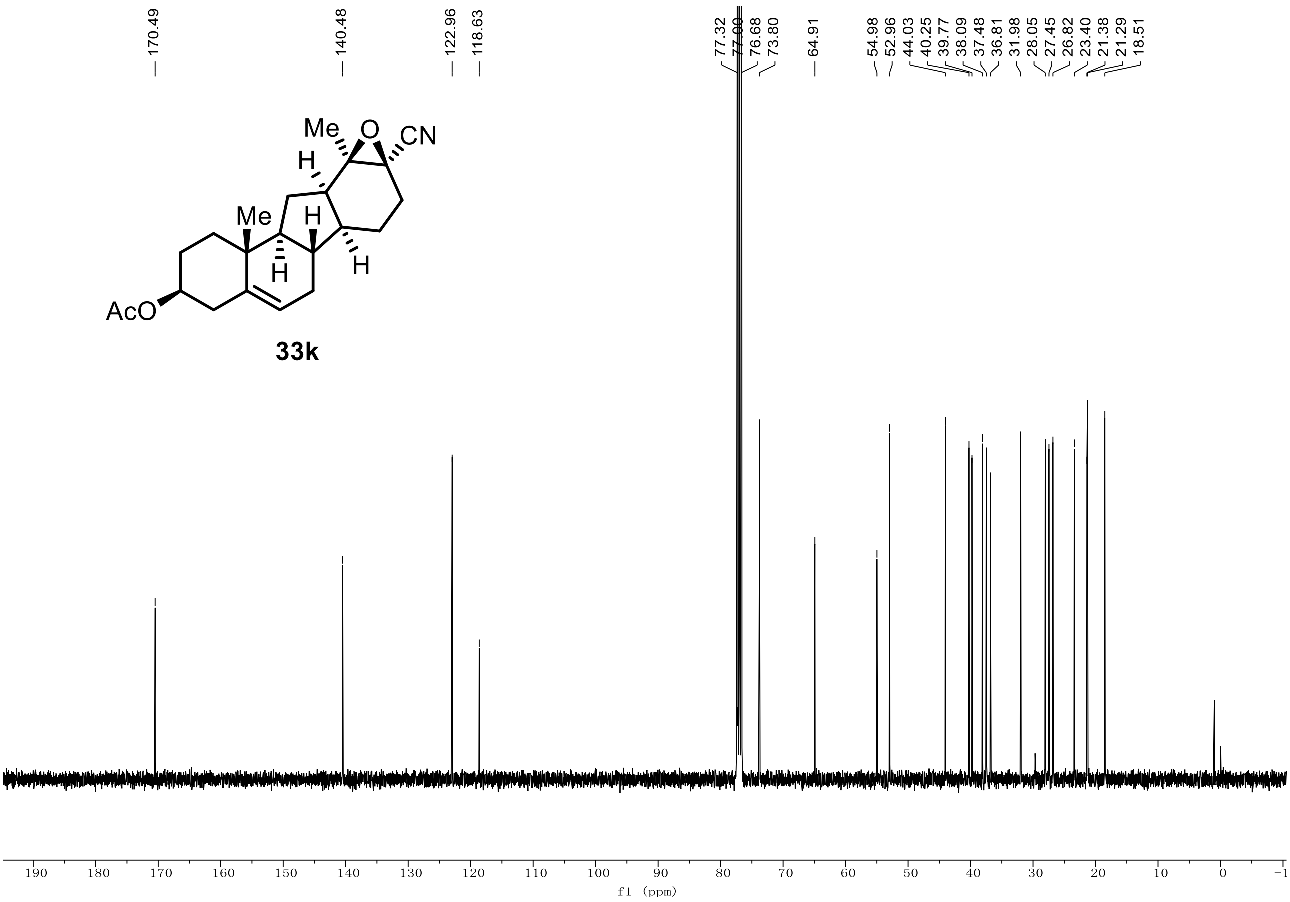

^{1}H NMR of **33l** (400 MHz, $CDCl_3$)

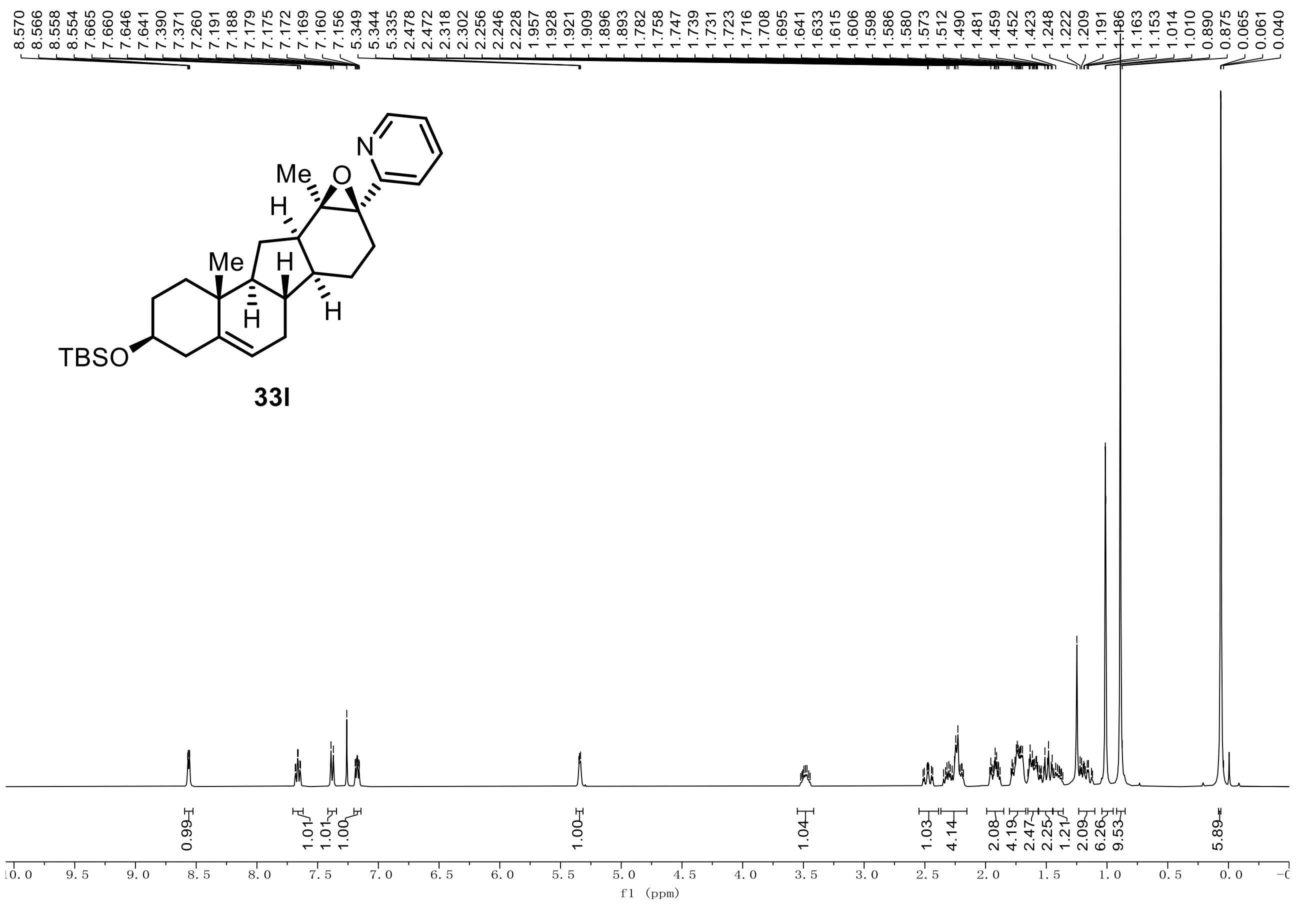

^{13}C NMR of **33l** (100 MHz, $CDCl_3$)

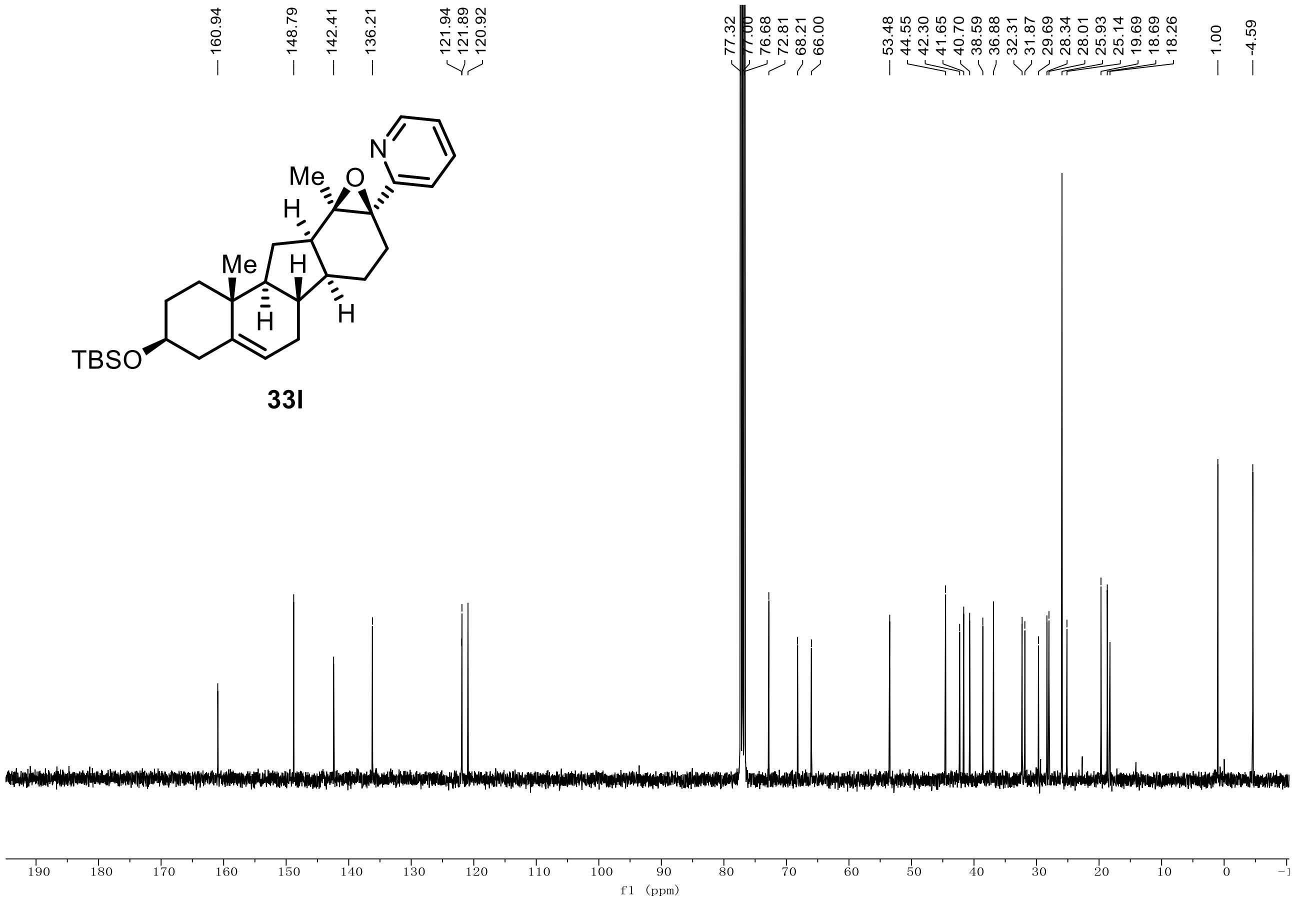

^{1}H NMR of **S18** (400 MHz, $CDCl_3$)

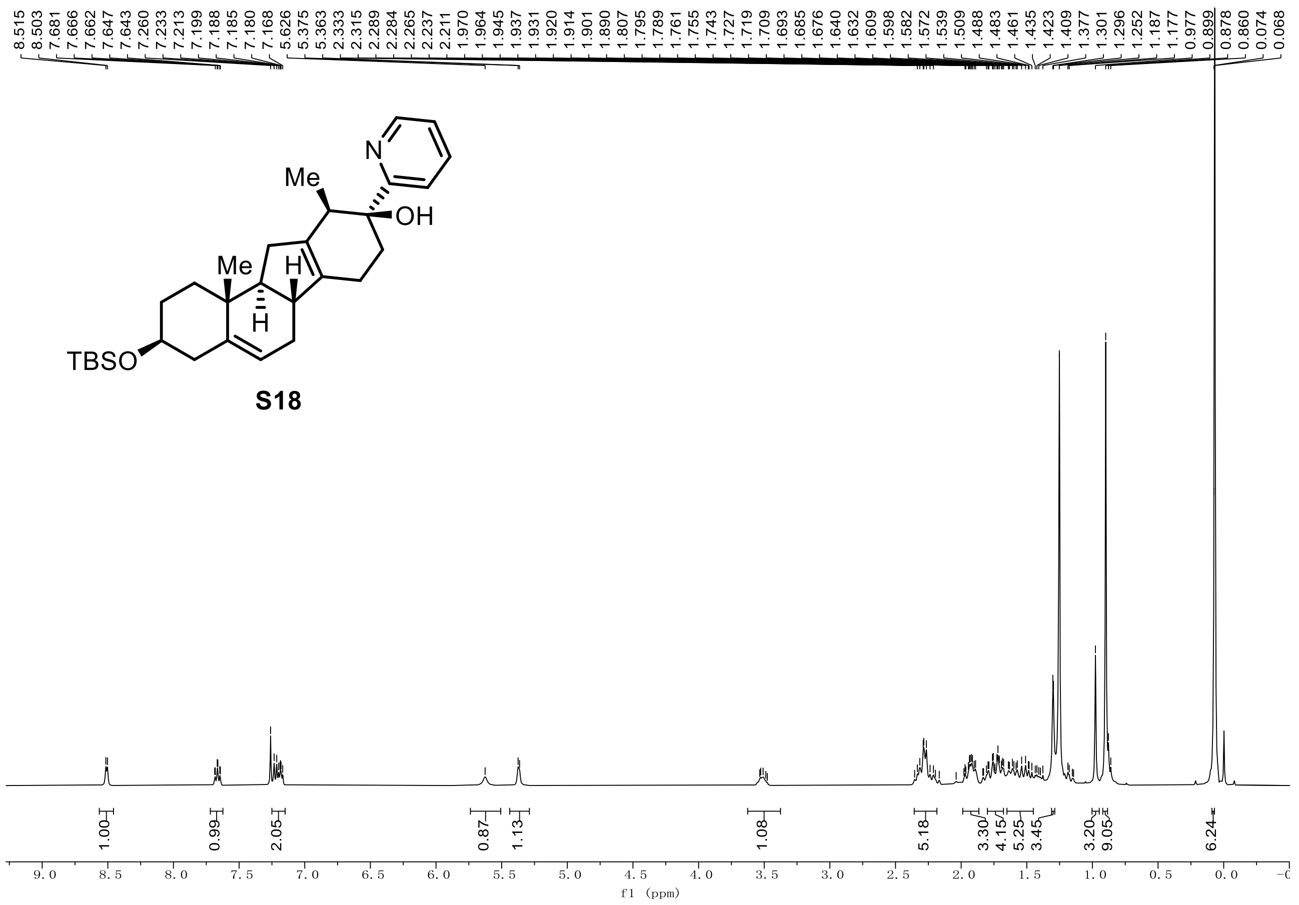

^{13}C NMR of **S18** (100 MHz, $CDCl_3$)

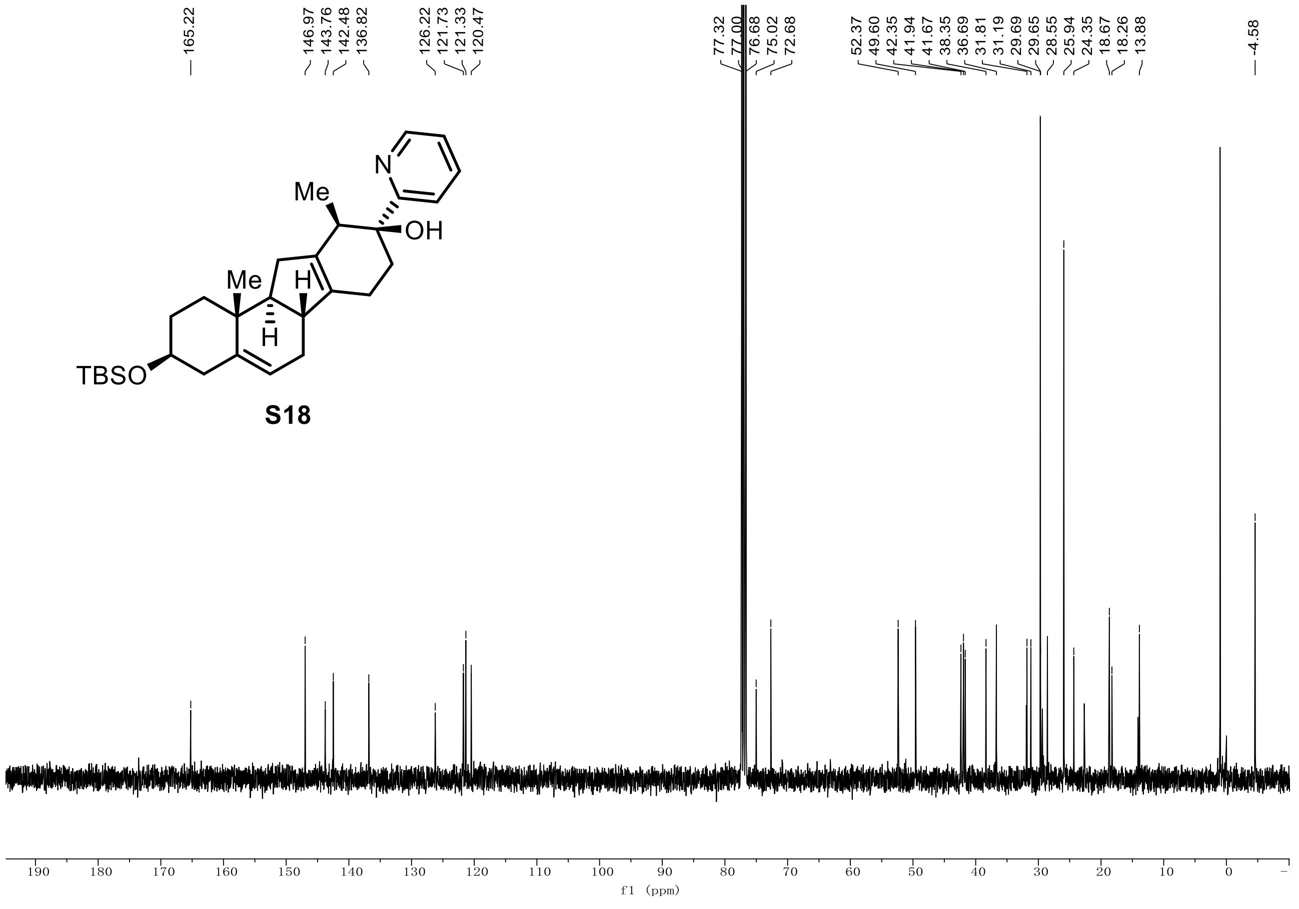

5. References

1) See, Y. Y., Herrmann, A. T., Aihara, Y. & Baran, P. S. Scalable C–H oxidation with copper: synthesis of polyoxypregnanes. *J. Am. Chem. Soc.* **137**, 13776–13779 (2015).
2) Meiries, S., Bartoli, A., Decostanzi, M., Parrain, J.-L. & Commeiras, L. Directed studies towards the total synthesis of (+)-13-deoxytedanolide: simple and convenient synthesis of the C8–C16 fragment. *Org. Biomol. Chem.* **11**, 4882–4890 (2013).
3) Li, S. et al. Rhodium-catalyzed enantioselective anti-Markovnikov hydroformylation of α-substituted acryl acid derivatives. *Org. Lett.* **22**, 1108–1112 (2020).
4) Imamoto, T. & Ono, M. The reaction of samarium(III) iodide with samarium metal in tetrahydrofuran. A new method for the preparation of samarium(II) iodide. *Chem. Lett.* 501–502 (1987).
5) Shao, H. et al. Asymmetric synthesis of cyclopamine, a hedgehog (Hh) signaling pathway inhibitor. *J. Am. Chem. Soc.* **145**, 25086–25092 (2023).
6) Tezuka, Y., Kikuchi, T., Zhao, W., Chen, J. & Guo, Y. Two new steroidal alkaloids, 20-isoveratramine and verapatuline, from the roots and rhizomes of *Veratrum patulum*. *J. Nat. Prod.* **61**, 1078–1081 (1998).
7) Sofiadis, M. et al. Convergent total synthesis of (–)-cyclopamine. *J. Am. Chem. Soc.* **145**, 21760–21765 (2023).
